# Cognitive Behavioral Interventions for Children and Adolescents With Overweight or Obesity: A Systematic Review and Component Network Meta‐Analysis

**DOI:** 10.1111/obr.70118

**Published:** 2026-03-06

**Authors:** Xinran Xie, Yunhe Mao, Minghui Sun, Xinyu Zou, Yan Yang, Yiyuan Gao, Wei Xu, Lan Zhang, Leticia Kawano‐Dourado, Arnav Agarwal, Jiafeng Li, Xia Hong, Yufang Bi, Ying Liu, Jing An, Jiahui Ma, Yuzi Cao, Kailei Nong, Jiyun Guo, Jing Zeng, Mengnan Zhao, Yibin Zhang, Qinbo Yang, Baihai Su, Changzheng Yuan, Changjian Qiu, Gordon Guyatt, Sheyu Li

**Affiliations:** ^1^ Department of Endocrinology and Metabolism, West China Hospital Sichuan University Chengdu China; ^2^ Department of Guideline and Rapid Recommendation, Cochrane China Centre, MAGIC China Centre, Chinese Evidence‐Based Medicine Centre, West China Hospital Sichuan University Chengdu China; ^3^ Sports Medicine Centre, West China Hospital Sichuan University Chengdu China; ^4^ Department of Orthopaedics and Orthopaedic Research Institute, West China Hospital Sichuan University Chengdu China; ^5^ Department of Endocrinology, Tongji Hospital, Tongji Medical College Huazhong University of Science and Technology Wuhan China; ^6^ Faculty of Psychology Beijing Normal University Beijing China; ^7^ Mental Health Centre, West China Hospital Sichuan University Chengdu China; ^8^ Hcor Research Institute Hcor Hospital Sao Paulo Brazil; ^9^ Pulmonary Division, Heart Institute (InCor) University of Sao Paulo Brazil; ^10^ MAGIC Evidence Ecosystem Foundation Oslo Norway; ^11^ Department of Health Research Methods, Evidence and Impact McMaster University Hamilton Ontario Canada; ^12^ Division of General Internal Medicine McMaster University Hamilton Ontario Canada; ^13^ Department of Psychological Medicine Peking Union Medical College Hospital Beijing China; ^14^ Department of Psychiatry, Ruijin Hospital Shanghai Jiao Tong University School of Medicine Shanghai China; ^15^ Department of Paediatric Genetic, Metabolic, and Endocrine Disorders, West China Second University Hospital Sichuan University Chengdu China; ^16^ Department of Obstetrics and Gynaecology, West China Second University Hospital Sichuan University Chengdu China; ^17^ Key Laboratory of Birth Defects and Related Diseases of Women and Children (Ministry of Education), West China Second University Hospital Sichuan University Chengdu China; ^18^ Department of Nephrology, West China Hospital Sichuan University Chengdu China; ^19^ School of Public Health Zhejiang University Hangzhou China

**Keywords:** adolescents, children, cognitive behavioral therapy, obesity

## Abstract

**Background:**

The efficacy of individual cognitive behavioral therapy (CBT) components for managing pediatric obesity remains unclear. This study systematically evaluated the impacts of CBT and its constituent techniques in this population.

**Method:**

We searched PubMed, Embase, and Cochrane CENTRAL from inception to July 17, 2024, for randomized controlled trials comparing CBT techniques or usual care targeting obesity management in children and adolescents with overweight or obesity. Component network meta‐analyses provided estimates of effects of each component on obesity‐related outcomes. We rated the certainty of evidence using modified GRADE approaches.

**Results:**

We included 125 trials with 16,513 children and adolescents. For conceptual level components, compared with minimal education, behavioral therapy probably reduces body fat percentage (MD, −1.16%; 95% CI, −1.68% to −0.64%), waist circumference (MD, −1.70 cm; 95% CI, −2.74 to −0.67 cm), and improves quality of life (SMD, 0.16; 95% CI, 0.03–0.30). For technical‐level components, when compared with minimal education, parental involvement (MD, −0.09; 95% CI, −0.16 to −0.03) and stimulus control (MD, −0.07; 95% CI, −0.12 to −0.01) probably reduce body mass index (BMI) z‐score. Preplanning (MD, −3.05%; 95% CI, −5.82% to −0.28%) and feedback (MD, −2.73%; 95% CI, −5.31% to −0.14%) probably reduce body fat percentage, whereas device monitoring, problem‐solving, rule‐setting, and relaxation training might increase body fat percentage.

**Interpretation:**

Behavioral therapy alone is likely effective for pediatric obesity management, irrespective of cognitive therapy integration. Techniques such as parental involvement, stimulus control, preplanning, and feedback should be prioritized in CBT.

**Abbreviations**
BMI: body mass index
CBT: cognitive behavioral therapy
MD: mean difference
MID: minimal important difference
SMD: standardized mean difference

## 1 Introduction

Obesity affects approximately 160 million children and adolescents worldwide [1] and is anticipated to affect over 250 million by 2030 [2]. Adolescents with obesity are at an elevated risk of developing Type 2 diabetes, hypertension, nonalcoholic fatty liver disease, and cardiovascular diseases, all of which are associated with increased morbidity and mortality [3, 4, 5]. Approximately 80% of adolescents with overweight or obesity continue to live with the conditions and their complications into adulthood, exacerbating downstream and long‐term health issues [6].

Health behavior and lifestyle counseling is the cornerstone of overweight or obesity management [7, 8]. However, when unaccompanied by structured treatment strategies to maintain healthy behaviors, most individuals are likely to relapse and regain weight [9]. Cognitive behavioral therapy (CBT) has emerged as a complementary therapy to promote healthy behaviors and cognitive patterns while supporting sustainable weight loss in children and adolescents with overweight or obesity [10, 11, 12]. It typically combines cognitive and behavioral therapy components and psychoeducation [13, 14]. Cognitive strategies focus on encouraging healthy thoughts and challenging and restructuring negative beliefs that result in harmful behaviors or emotional distress [15]. Behavioral strategies focus on identifying and modifying maladaptive behaviors (e.g., diet and physical activity) [16]. Psychoeducation promotes knowledge and understanding regarding how thoughts and behaviors affect weight and to support cognitive and behavioral changes [17].

Supported by multiple randomized trials and systematic reviews [10, 11, 12, 18], obesity management guidelines may consider CBT in the treatment of obesity among children and adolescents [19]. Nevertheless, significant evidence gaps persist regarding the specific therapeutic value of individual CBT components. Existing studies demonstrate substantial heterogeneity in CBT techniques across trials and clinical settings, with limited understanding about which specific components contribute most effectively to weight management outcomes [18, 19]. To address this critical knowledge gap, we developed a standardized taxonomy of CBT components through a multidisciplinary consensus process. This study aimed to assess the comparative effects of different treatment components of CBT and control of weight‐related outcomes, quality of life, and mental health in children and adolescents with overweight or obesity. We thus conducted a systematic review and component network meta‐analysis and rated the certainty of evidence using a modified Grading of Recommendations, Assessment, Development, and Evaluation (GRADE) approach. This study differentiated core active components from incidental components in CBT interventions while systematically evaluating the certainty of evidence for each therapeutic component across obesity‐related outcomes.

## 2 Methods

This study followed PRISMA 2020 and PRISMA‐NMA statements [20] and was registered in the Prospective Register of Systematic Reviews (ID: CRD42025632579).

### 2.1 Data Sources and Searches

We searched MEDLINE, Embase, and Cochrane CENTRAL via Ovid from inception to July 17, 2024, using a predefined search strategy using keywords and MeSH terms including adolescent, children, obesity, overweight, and CBT (Appendix S1).

### 2.2 Eligibility Criteria

Randomized controlled trials (RCTs) enrolling children and adolescents with overweight or obesity, using the age definition specified by authors of included trials, were eligible if they either directly compared two distinct forms of CBT or evaluated a CBT intervention against a control group receiving only minimal education. For this review, minimal education was defined as basic lifestyle advice delivered without structured behavioral modification components, ensuring that observed effects could be attributed to the CBT‐specific elements of the intervention (as defined in our “Taxonomy of CBT” below and Table 2). Eligible treatment programs were at least 12 weeks in duration. The definitions of overweight or obesity followed the trial definitions with overweight typically as BMI in the 85th percentile or higher and obesity typically as BMI in the 95th percentile or higher. We excluded trials focused on psychiatric or psychological conditions (e.g., eating disorders, depression, psychosis, and suicidality), those that randomized individuals during the weight maintenance period, and those that were published in a language other than English.

### 2.3 Study Selection

After completing pilot testing, paired reviewers (X.X., M.S., Y.Z., Y.G., Y.C., J.G., and J.M.) used standardized forms to independently screen identified hits at the title and abstract and full‐text levels. Discrepancies were resolved through discussion or by a senior reviewer (S.L.).

### 2.4 Data Extraction and Data Items

Paired reviewers used standardized forms to independently extract the following data from eligible trials:

- Study characteristics: Year, country, setting, funding, and length of follow‐up.
- Baseline patient characteristics: Demographic characteristics (age and proportion of girls), number of patients, and baseline obesity‐related complications or relevant comorbidities.
- Content of different CBT interventions. These intervention details will subsequently be classified according to the “Taxonomy of CBT” described in the following section.
- Outcomes: Trial‐specific outcome definitions, magnitude of effect sizes and corresponding sample sizes

All extracted data underwent cross‐checking, with discrepancies resolved by a senior reviewer (S.L.). We prioritized results from intention‐to‐treat (ITT) or modified ITT analyses over per‐protocol results. When both adjusted end‐of‐treatment and change‐from‐baseline values were reported, we prioritized change‐from‐baseline values. If only end‐of‐treatment values were available, the adjusted end‐of‐treatment values were extracted next, followed by unadjusted ones.

### 2.5 Outcomes and Effect Measures

In evaluating patient‐important outcomes, we prioritized BMI z‐score as the primary outcome, as this standardized measure inherently accounts for age‐ and sex‐specific growth patterns critical in pediatric populations, which was calculated using WHO growth curves with Lambda–Mu–Sigma normalization. Although absolute BMI values were also assessed, they were interpreted separately as a secondary outcome, given their limited capacity to disentangle physiological growth from true weight changes in developing youth.

Other anthropometric outcomes included change in height, waist circumference, and body fat percentage (measured through any validated method). For psychosocial impacts, quality‐of‐life and mental health scores were analyzed.

Absolute changes of BMI z‐score, height, waist circumference, and body fat mass were reported as mean differences (MD); the absolute change from baseline in scores for quality of life and mental health was reported as standardized mean differences (SMD).

### 2.6 Taxonomy of CBT

Through interview and discussion among a multidisciplinary team including endocrinologists, psychologists, psychiatrists, and pediatricians, we drafted a taxonomy for the components of CBT treatment for children and adolescents with overweight or obesity. The CBT component taxonomy involved classification at technical and conceptual levels. We considered technical‐level classifications as specific interventional techniques, which can be directly applied to the design of a CBT intervention, for example, cognitive restructuring. We considered conceptual‐level classifications as groups of these techniques in a concept level, for example, cognitive and behavioral interventions. Specifically, at the conceptual level, the taxonomy included cognitive therapy components (at technical level including cognitive restructuring, third‐wave components and self‐concept improvement), behavioral therapy components (at technical level including functional behavioral analysis, motivation, goal‐setting, task‐setting, rule‐setting, self‐monitoring, device‐monitoring, reminders, feedback, problem‐solving, preplanning, reinforcement, contracting, modeling, stimulus control, relaxation training, inhibition training, social support, stress management, role playing), and educational components (at technical level including psychoeducation and stoplight approach). Interventions were also categorized based on whether they targeted a group or an individual; were delivered remotely or face‐to‐face; whether they involved serious games or not; and whether they involved parental involvement or not. One reviewer (X.X.) categorized the treatment components of each included study into the taxonomy, which were then checked by another reviewer (M.S.). Any uncertainty or disagreement was discussed with the experts from our multidisciplinary team. Minimal education was used as control and defined as no treatment, wait‐list, minimal care, or usual primary care.

### 2.7 Geometry of the Network

We summarized the network structure by tabulating the number of trials and participants contributing to each pairwise comparison. The network plot (Appendix S4) depicts the nodes (treatments) and edges (direct comparisons); the thickness of each edge is proportional to the number of trials.

### 2.8 Risk of Bias Assessment

Paired reviewers (X.Z. and Y.G.) independently assessed the risk of bias for included trials at the outcome level using the Cochrane Risk of Bias Tool 2.0 for Randomized Trials (ROB‐2) [21], which involves five domains including the randomization process, deviations from intended interventions, missing outcome data, measurement of the outcome, and selective reporting. Discrepancies were resolved through discussion or by a senior reviewer (S.L.).

### 2.9 Data Synthesis and Analysis

Using the *netmeta* package in R, we conducted a random‐effect frequentist NMA at the conceptual level. Estimations were based on weighted least‐square regression with the Moore–Penrose pseudoinverse method [22]. Indirect estimates were obtained from the network through node splitting and back calculation methods within the network loops. MD, SMD, and corresponding 95% confidence intervals were estimated for continuous outcomes. Using extension functions from *netmeta* package, we further implemented an additive component NMA model under a random‐effect frequentist framework to estimate the component‐specific effect sizes for each component. To quantify the contribution of evidence from constituent comparisons to the component effect estimates, we applied a leave‐one‐out algorithm that assesses precision leverage upon exclusion of each direct comparison [23, 24]. Component analyses were performed at both conceptual and technical levels [25] with minimal education as the inactive component. Component‐specific effects ( $\hat{\beta }$) were derived from $\hat{\beta }=\left(X^{T},\text{WX}\right)⁺X^{T}\text{Wd}$, and treatment‐level effects (θ) were subsequently computed as θ̂ $=C\;\hat{\beta }$ with their covariance matrix $C\;\left(X^{T},\text{WX}\right)⁺C^{T}$ [26] (see Appendix S1.3 for full details). All pairwise contrasts were then calculated from θ̂ together with 95% confidence intervals obtained from the estimated covariance matrix. Contribution matrices were generated to assess the contribution of each direct comparison.

Heterogeneity was assessed using the generalized Cochran's *Q* test and *I* ^2^ statistics, where *I* ^2^ values exceeding 50% indicated substantial heterogeneity [27] (see Appendix S4.5 for full details). Direct evidence for the comparisons in each outcome was further rated down for high heterogeneity in the evidence certainty rating phase. For outcomes involving nine or more trials, we assessed publication bias and small study effects through visual inspection of comparison‐adjusted funnel plots and as well as using Begg's rank correlation test and Egger's regression test. Intransitivity was judged based on distribution comparisons of potential effect modifiers (i.e., baseline age, sex, BMI z‐score, and length of follow‐up) for each direct comparison and outcome.

### 2.10 Subgroup and Sensitivity Analysis

We conducted three subgroup analyses/meta‐regressions: (1) people with overweight versus obesity (hypothesizing larger effect sizes across outcomes in obesity); (2) children versus adolescents (hypothesizing larger effect sizes across outcomes in adolescents); and (3) setting (involving schools versus not involving schools, hypothesizing larger effect sizes across outcomes in trials involving schools). The credibility of any apparent subgroup effect (regression coefficient's interval excludes null effect) was assessed using the ICEMAN tool [28].

To test the robustness of our results, we conducted four sensitivity analyses: (1) using end‐of‐follow‐up data instead of end‐of‐treatment data for analyses if different; (2) excluding trials with treatment duration of less than 24 weeks; (3) excluding trials with high risk of bias; and (4) splitting waiting list from minimal education as a separate treatment component. Furthermore, we also analyzed absolute change of BMI as a different measure of the same result with BMI z‐score.

### 2.11 Evidence Certainty Rating

We employed a modified GRADE framework to assess the certainty of evidence from the component NMA [29, 30], a methodologically intricate system that will be elaborated in forthcoming methodology‐focused publications. At its core, our approach utilized a contribution proportion‐anchored strategy to evaluate certainty for component estimates. Direct evidence was initially assigned high certainty but could be downgraded based on risk of bias, inconsistency, indirectness, or publication bias [31]. Additive estimates from multicomponent trials faced potential additional downgrades for incoherence.

This uniquely developed methodological framework involved quantifying the proportional contribution of each direct evidence source to specific component estimates [22]. The initial certainty of component estimates was weighted proportionally based on these contributions. For estimates informed by mixed direct and additive evidence, we applied a “proportional dominance rule” to categorize certainty:

- High certainty: If high certainty evidence contributed 50% or more to the estimate
- Moderate certainty: If a combination of high and moderate certainty evidence contributed 50% or more, but high certainty evidence alone contributed less than 50%
- Low certainty: If high and moderate certainty together contributed less than 50%, and very low certainty evidence contributed less than 50%
- Very low certainty: If very low certainty evidence contributed 50% or more to the estimate

We assessed incoherence by calculating the Cochran's *Q* test across the integrated direct, additive indirect, and component estimates. If the corresponding *p* value < 0.1 or *I* ^2^ > 50%, we considered severe statistical incoherence. For comparisons with severe statistical incoherence, we applied “best evidence” consideration aligned with standard NMA GRADE practice. Appendix S1.4 presents more details of the GRADE assessment methodology. All such decisions were made case by case. For the target component or combination of components potentially used for practice directly, we rated imprecision using the minimal important difference (MID) as the decision threshold; for those not directly used for practice, we rated the imprecision using the null effect as the decision threshold. We adopted the MIDs from the literature as follows [32, 33, 34, 35]: (1) 0.1 change for BMI z‐score; (2) 1.0 kg/m^2^ change for BMI; (3) 5% for fat percentage change; (4) 2 cm change for waist circumference; (5) 0.12 SMD for quality‐of‐life score; (6) 0.12 SMD change for mental health score; and (7) null effect for height.

### 2.12 Protocol Deviations

The registered protocol stated “PubMed”; however, we searched MEDLINE via Ovid to ensure reproducibility through controlled vocabulary (MeSH) and consistent syntax. In the original protocol, children and adolescents under 19 were the targeted population, yet considering the varying definition of children and adolescents across countries and regions, we followed the criteria from each included trial. These changes do not affect the scope of the search.

## 3 Results

### 3.1 Study Selection

**FIGURE 1:**
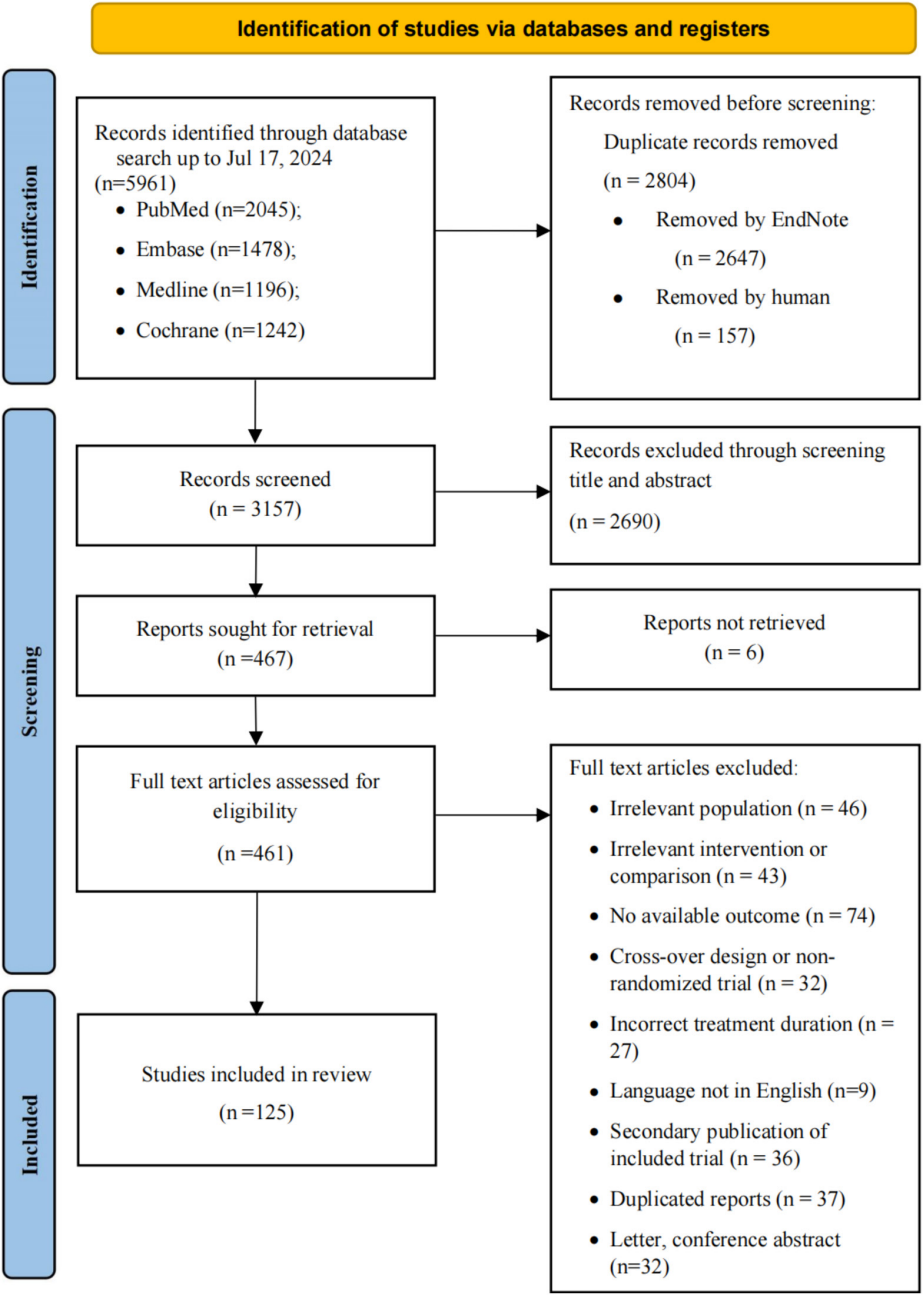
Flow diagram for trial screen and selection.

Of 5961 citations identified, our team assessed 461 full manuscripts for eligibility, of which 125 RCTs proved eligible (Figure 1).

### 3.2 Study Characteristics

The 125 eligible trials enrolled 16,513 children and adolescents with overweight or obesity (Figure 1). Table 1 shows the characteristics of eligible trials with additional details in Appendix S2. The included trials were conducted across 29 countries/regions, with the United States as the most common country (58 trials, 46%). Fourteen trials (11.4%) were conducted in developing countries, including Brazil, China, Indonesia, Iran, Malaysia, Mexico, and Turkey. Median treatment duration was 26 weeks. The mean age of the participants was 11.12 years old, with 56.83% girls. Mean baseline BMI was 27.68 kg/m^2^ with a mean z‐score of 2.32. The reviewers identified 32 high risk of bias domains in 24 of 125 (19.20%) trials. Appendix S3 presents the details of assessments of risk of bias. The most common high risk of bias domain was bias due to missing outcome data (62.5%). Appendix S4 presents heterogeneity, intransitivity, and inconsistency evaluations. The included trials formed connected networks at the conceptual level and disconnected ones at the technical level. It is worth noting that due to the limited trials included and excessive components as well as treatment combinations, the network at technical level is sparse. Two outcomes, including BMI z‐score and BMI, were considered with statistically significant heterogeneity in between designs (Appendixes S4.5 and S8.3, respectively). The evidence did not suggest intransitivity for any outcomes (Appendixes S4.6 and S8.4, respectively).

**TABLE 1:** Summary of characteristics of included studies.

Study characteristics	No. %/mean	95% confidence interval
Eligible studies		
Total no. of trials	125	
No. of participants	16,513	
Studies in LMICs	14 (11.2%)	
Most common countries	USA: 58 (46.4%)	
Most common settings	Clinic and hospital: 48 (38.4%)	Primary care setting: 26 (20.8%)
Median treatment duration (weeks)	26	16–52 (IQR)
Median follow‐up (weeks)	52	26–52 (IQR)
Participants		
No. of females (%)	56.83	
Age (years)	11.12	10.59–11.65
Body mass index (kg/m^2^)	27.68	26.74–28.61
BMI z‐score	2.32	2.22–2.42

Abbreviations: IQR, interquartile range; LMIC, low‐ or middle‐income country.

### 3.3 Taxonomy of CBT

Table 2 illustrates the taxonomy of CBT components including conceptual and technical levels. CBT was composed of psychoeducation, cognitive, and behavioral therapy at the conceptual level. The technical‐level taxonomy shown in Table 2 consisted of 26 interventional components and three special delivery strategy components: group versus individual, remote versus face‐to‐face, and using versus not using serious games. According to the definitions in Table 2, we identified the components in all included intervention arms. Among the 29 components, psychoeducation (*n* = 121), parental involvement (*n* = 111), and goal‐setting (*n* = 91) were the most frequently used (Table 2).

**TABLE 2:** Taxonomy of cognitive behavioral therapy.

Components	No. of studies	Definition
Psychoeducation	**/**	Treatment that aims to provide information about the cause and nature of a specific health condition to enhance understanding and also provides general coping skills
Psychoeducation	121	Provision of information about the cause and nature of obesity and general recommendations about lifestyle (e.g., diet, exercise, and substance use) to lose weight. Advice about lifestyle modification (e.g., exercise and food as opposed to cognitive behavioral therapy) was regarded as a form of psychoeducation
Stoplight approach	27	Traffic light diet training, a nutritional approach that categorizes foods into green, yellow, and red based on their calorie and fat content, helping individuals make healthier choices by limiting red foods, moderating yellow foods, and promoting green foods for balanced nutrition and weight management
Cognitive therapy	/	Treatments that aim to challenge negative thought patterns that result in harmful behaviors or emotional distress, based on the premise that thoughts and behaviors are interconnected and that changing one can affect the others
Cognitive restructuring	13	Skills to identify, challenge, and modify mal‐adaptive or distorted thoughts and beliefs about overeating, high‐calorie foods, and sedentary lifestyle using strategies such as Socratic questioning and guided imagery. Sometimes simply called cognitive therapy. This may include behavioral experiments
Third‐wave components	5	Mindfulness: A form of meditation emphasizing a nonjudgmental state of heightened or complete awareness of one's thoughts, emotions, or experiences on a moment‐to‐moment basis Acceptance and commitment therapy: Focuses on accepting the feelings and thoughts associated with obesity through value‐based behaviors Dialectical behavior therapy: Emphasizes balancing acceptance and change strategies to help individuals manage intense emotions and develop coping skills for undesired behaviors and other mental health challenges
Self‐concept improvement	41	Improvement of beliefs, attitudes, and perceptions about the participant's identity, abilities, and worth, that is, the enhancement of self‐efficacy, self‐esteem and body image
Behavioral therapy	/	Treatment focusing on identifying and modifying maladaptive behaviors directly. It is grounded in the principles of learning theory that behaviors are learned through processes such as modeling and reinforcement
Functional behavioral analysis	19	A process used to identify and understand the relationship between specific behaviors and their antecedents and consequences, with the goal of developing effective interventions to modify mal‐adaptive behaviors, including the management of emotion, hunger and food cravings
Motivation	55	Motivational interviews or motivational statements to increase individual's willingness and commitment to engage in therapeutic activities and make positive changes in their thoughts, emotions, and behaviors
Goal‐setting	91	The development of an action plan designed to guide the individual or group toward specific, measurable, achievable and time‐bound objectives Specific behavioral change targets and short‐term goals are specifically categorized as goal‐setting
Task‐setting	27	Regular homework assignments outside of therapy sessions, either checked by humans or mandated by a computer program. It is designed to help individuals apply the learned CBT or other behavioral skills. It requires some active participation from the participant, thereby reinforcing therapeutic progress and fostering greater self‐efficacy. Simple reviewing of the materials or further reading were not regarded as homework
Rule‐setting	15	Establishment of a set of clear behavioral regulations to help children change unhealthy eating habits and increase physical activity. These rules are typically family rules developed collaboratively by the therapist, the child, and their family
Self‐monitoring	58	Self‐observation and self‐recording, for example, diet diary The behaviors, thoughts, and feelings that occur before, during, and after attempts at prudent eating and exercise behaviors are recorded to increase awareness and facilitate better self‐regulation
Device‐monitoring	21	Technological devices to track and analyze various physiological or behavioral metrics, providing objective data to help better self‐monitoring or to inform treatment decisions, e.g., device‐monitored smaller portion sizes or reduction in the rate of eating, pedometers helping to monitor the daily footsteps.
Reminders	18	Cues or prompts used to help individuals remember to engage in specific therapeutic activities, practice new skills, or adhere to treatment recommendations. It can be in person, via telephone or email or by other means
Feedback	55	Information provided by an agent (e.g., therapist, peer, book, parent, self, and experience) regarding aspects of one's performance
Problem‐solving	64	Identifying personal problems/barriers that prevent goal attainment, analyzing and resolving specific issues or challenges, empowering individuals to develop effective solutions, and enhancing their coping skills
Preplanning	31	Development of plans in advance for high‐risk situations (e.g., parties) to decrease likelihood of unhealthful eating or lack of physical activity
Reinforcement	56	Rewards (verbal praises, objects, events, situations, or activities as motivational rewards, e.g., a voucher) are provided contingent on objective evidence of weight loss progress or behavior changes (appropriate healthful behavioral patterns related to weight loss or maintenance). Punishment may also be included
Contracting	16	Establishment of a specific, measurable, achievable, and time‐bound goals and agreements between the therapist (or the parents) and participants
Modeling	37	Parents or peers' model healthy lifestyle for participants. By watching and emulating positive role models, children can develop essential skills for behavior modification and weight management
Stimulus control	50	Modification of environmental cues that lead to inappropriate behaviors to make healthful choices more available For example, bedtime routine, eating at the dinner table, and availability of energy‐dense foods and beverages
Relaxation training	4	Induction of a relaxed body state to reduce general tension using techniques such as deep breathing, progressive muscle relaxation, and guided imagery
Inhibition training	6	Skills to improve behavior by strengthen executive control to stimulus, such as delay discounting
Social support	38	Mainly indicates peer support and cooperation; enhancement of participants' abilities to interact effectively with others, including communication, assertiveness, and conflict resolution skills, to improve social functioning and overall well‐being
Stress management	16	Coping strategies and relaxation techniques to effectively handle stress and emotions, thereby preventing unhealthy eating behaviors triggered by stress
Role‐playing	6	Simulation of real‐life situations to practice and reinforce healthy behaviors and responses, helping children build confidence in managing challenges related to weight management
Delivery strategies		
Group	74	Interaction with therapists as a member of a group, whether in person or remote
Remote	37	Interactive remote/digital techniques, including online courses, interactive website platform, or telephone coaching
Serious games	24	Interactive games designed for purposes beyond mere entertainment, often used for education, training, simulation, and social change
Parental involvement	111	Parents involved or family‐based interventions: general education for parents, in the hope for supportive family environment; parent‐monitoring of target behaviors; parents play an active role in the intervention Interventions targeted at parents only are also included
Control condition		
Minimal education	81	Minimal intervention provided for ethical reasons; general education about obesity without adequate counseling or any CBT contents; prompts or encouragements prepared and provided by human beings or automated, to proceed with the treatment programme via telephone or email; usual care; no treatment delivered in the intervention period; watchful waiting or follow‐up by researchers

### 3.4 Effect Estimates for Conceptual‐Level Components

At conceptual level, we identified 93 trials with 196 arms: nine arms with psychoeducation only, two with cognitive therapy only, 60 with behavioral therapy only, and 40 CBT.

**FIGURE 2:**
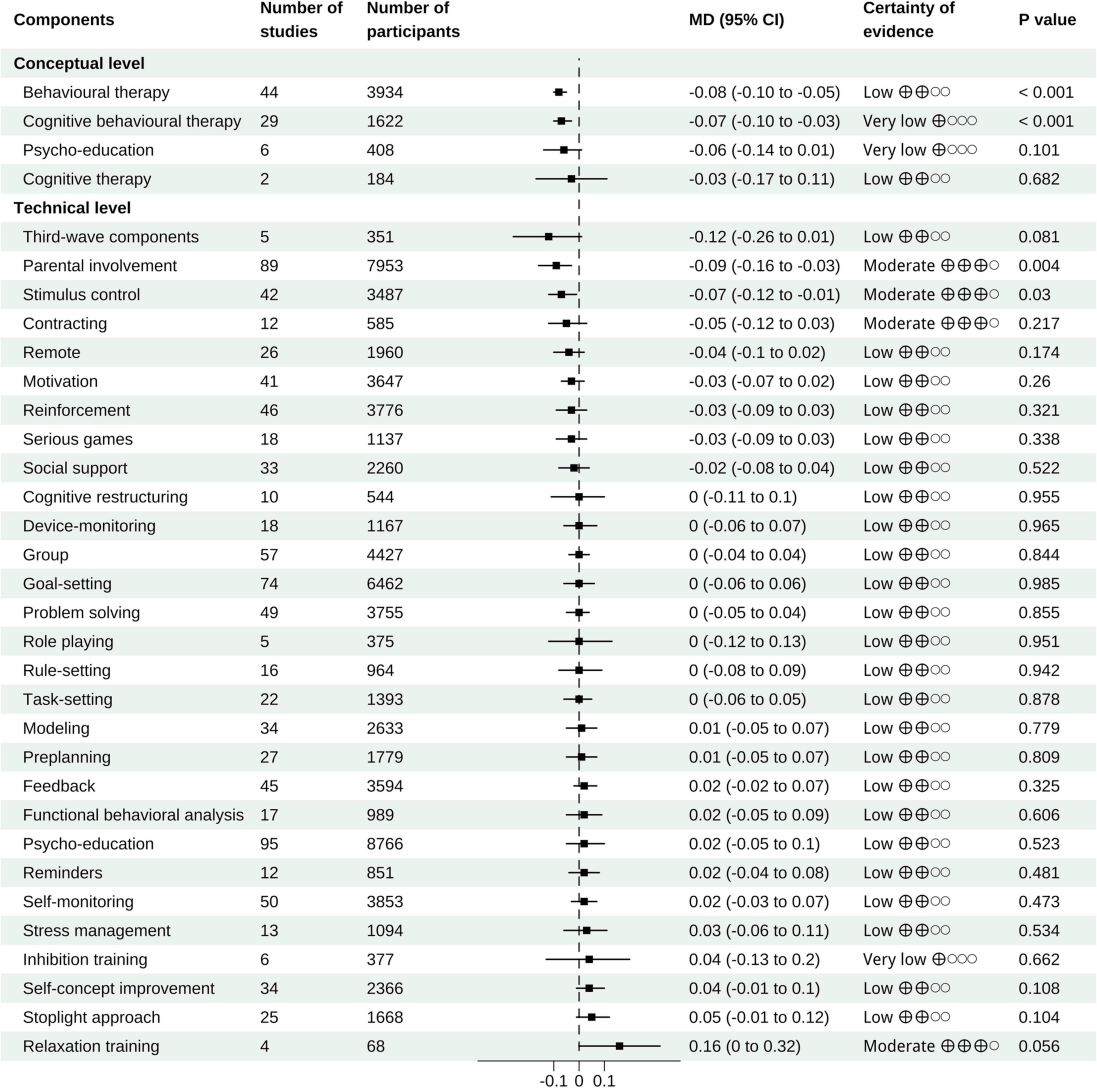
Effect estimates in BMI z‐score change for conceptual‐level and technical‐level components. 95% CI, 95% confidence interval. MD, mean difference. An MD of less than 0 shows that the component is favored.

**FIGURE 3:**
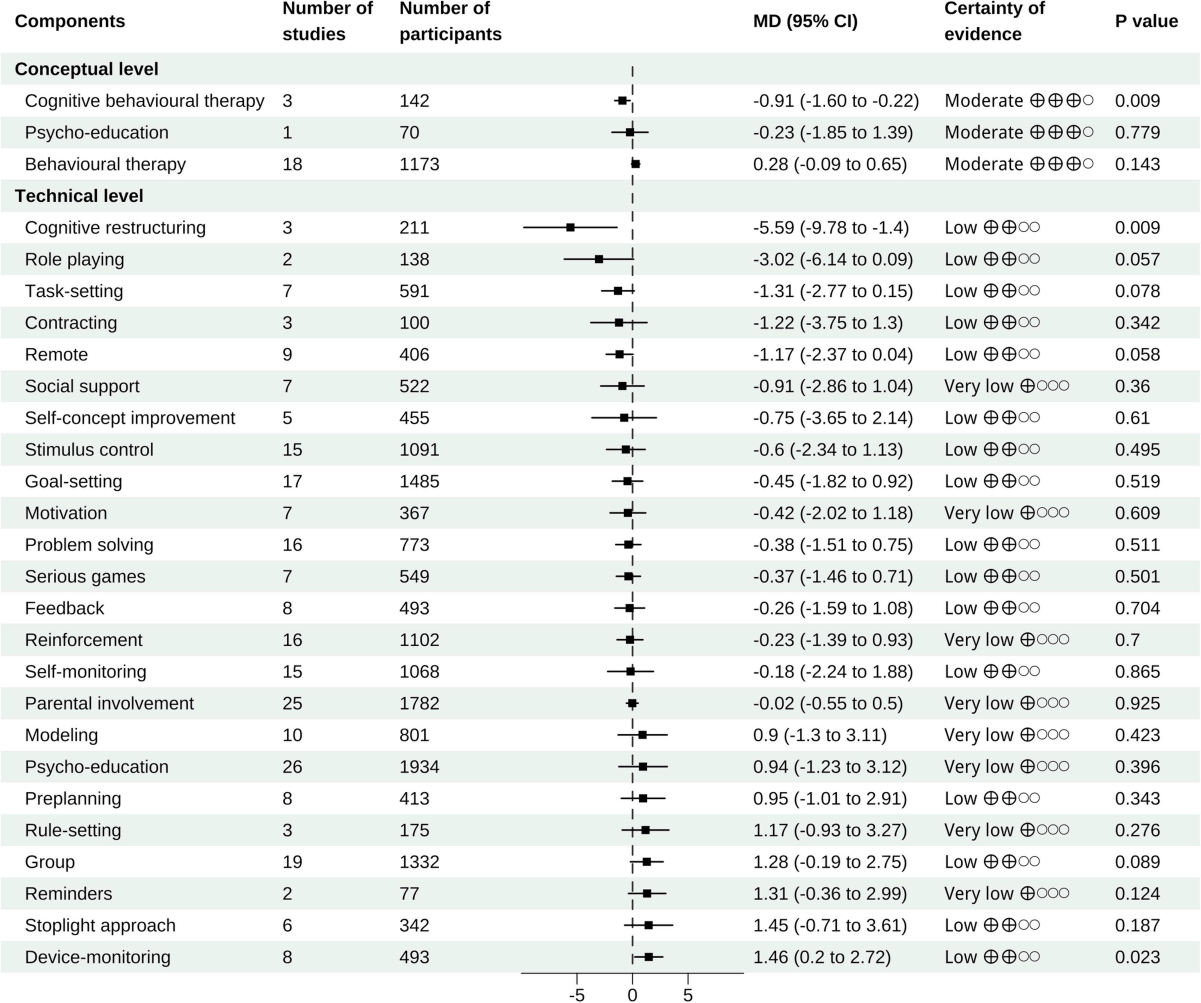
Effect estimates in height change for conceptual‐level and technical‐level components. 95% CI, 95% confidence interval. MD, mean difference. An MD of more than 0 shows that the component is favored.

**FIGURE 4:**
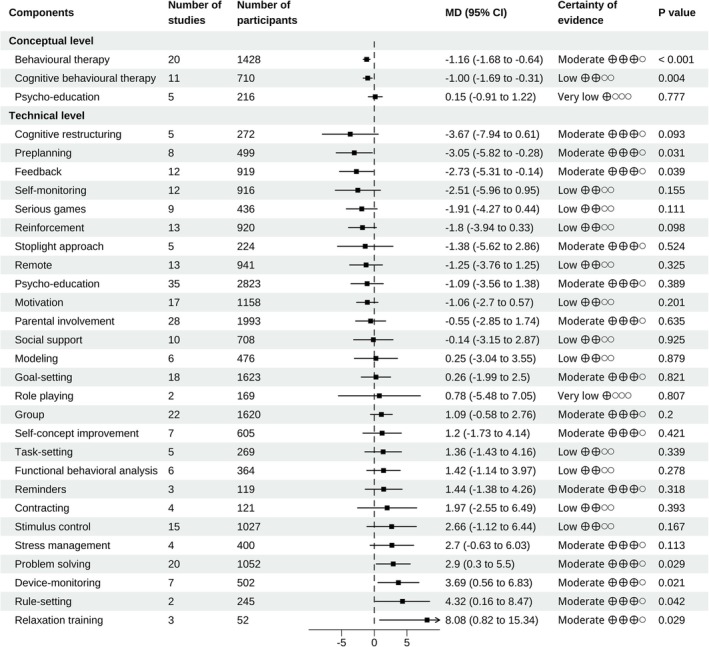
Effect estimates in body fat percentage (%) change for conceptual‐level and technical‐level components. 95% CI, 95% confidence interval. MD, mean difference. An MD of less than 0 shows that the component is favored.

**FIGURE 5:**
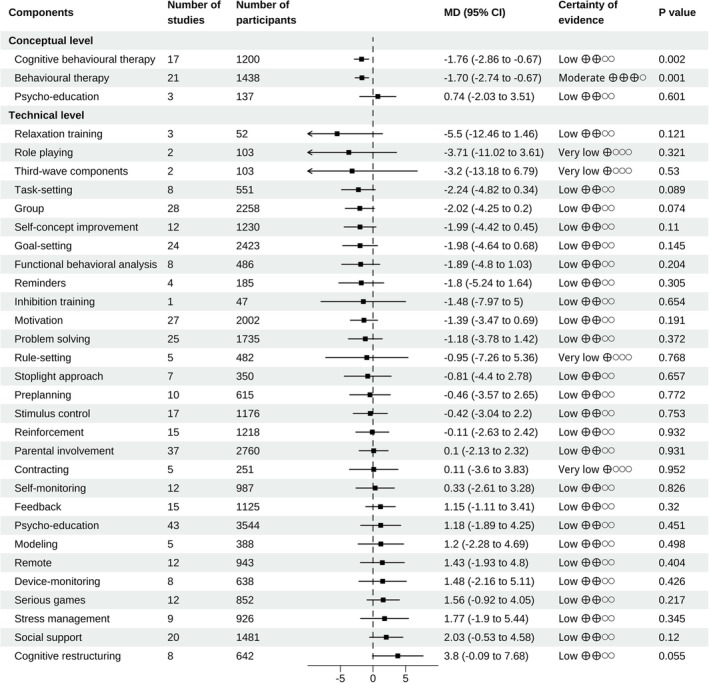
Effect estimates in waist circumference change for conceptual‐level and technical‐level components. 95% CI, 95% confidence interval. MD, mean difference. An MD of less than 0 shows that the component is favored.

**FIGURE 6:**
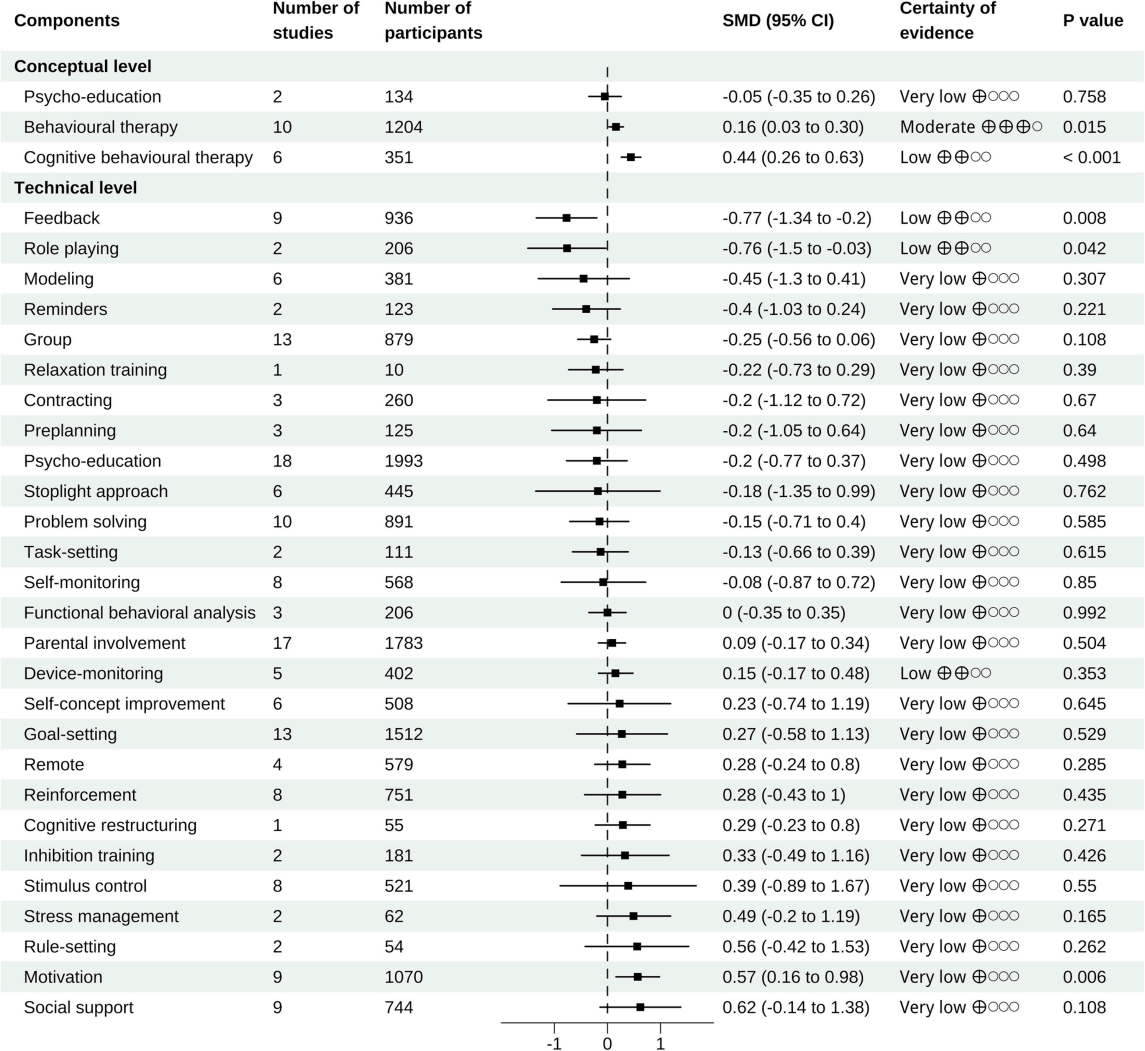
Effect estimates in quality‐of‐life score change for conceptual‐level and technical‐level components. 95% CI, 95% confidence interval. SMD, standardized mean difference. An SMD of more than 0 shows that the component is favored.

**FIGURE 7:**
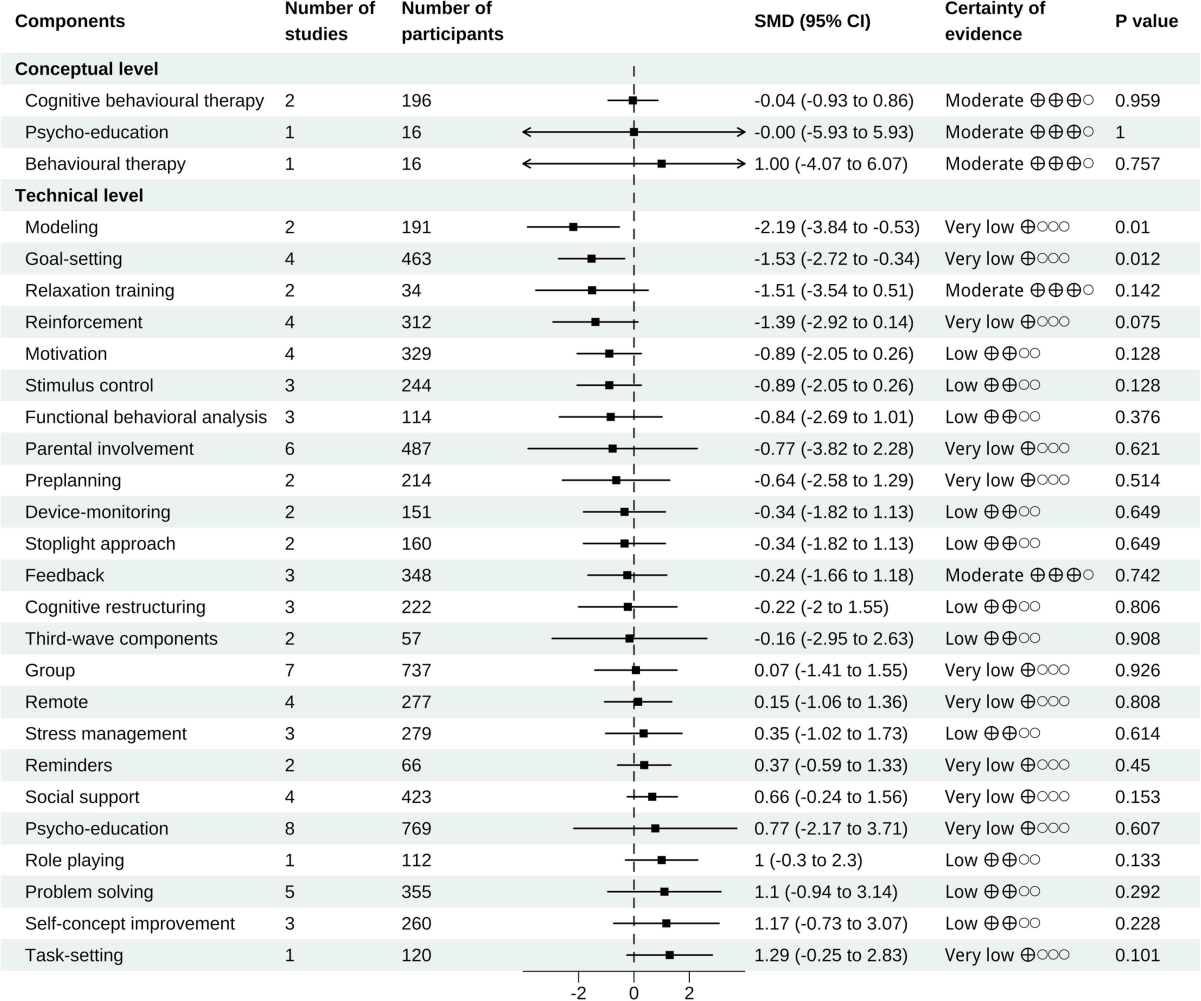
Effect estimates in mental health score change for conceptual‐level and technical‐level components. 95% CI, 95% confidence interval. SMD, standardized mean difference. An SMD of less than 0 shows that the component is favored.

Figures 2, 3, 4, 5, 6, 7 show the estimates of components at the conceptual level compared to minimal education.

All estimates compared each conceptual‐level intervention with minimal education. CBT (MD, −0.91 cm; 95% CI, −1.60 to −0.22 cm; moderate certainty; MID, 0 cm) probably suppresses the increase of height. Behavioral therapy probably reduces waist circumference (MD, −1.70 cm; 95% CI, −2.74 to −0.67 cm; moderate certainty; MID, 2 cm) and body fat percentage (MD, −1.16%; 95% CI, −1.68% to −0.64%; moderate certainty; MID, 5%) and improves quality of life with important difference (SMD, 0.16; 95% CI, 0.03–0.30; moderate certainty; MID, 0.12). Behavioral therapy (MD, 0.28 cm; 95% CI, −0.09–0.65 cm; moderate certainty) and psychoeducation (MD, −0.23 cm; 95% CI, −1.85–1.39 cm; moderate certainty) probably have no effect on height. CBT (SMD, −0.04; 95% CI, −0.93–0.86; moderate certainty; MID, 0.12), behavioral therapy (SMD, 1.00; 95% CI, −4.07–6.07; moderate certainty), and psychoeducation (SMD, 0.00; 95% CI, −5.93–5.93; moderate certainty) probably have no effect on mental health. Behavioral therapy possibly reduces BMI z‐score (MD, −0.08; 95% CI, −0.10 to −0.05; low certainty; MID, 0.1). CBT possibly reduces waist circumference (MD, −1.76 cm; 95% CI, −2.86 to −0.67 cm; low certainty) and body fat percentage (MD, −1.0%; 95% CI, −1.69% to −0.31%; low certainty) and improves quality of life with important difference (SMD, 0.44; 95% CI, 0.26–0.63; low certainty). Appendix S4 presents further details, and Appendix S5 presents all results together with their certainty‐of‐evidence assessments.

### 3.5 Effect Estimates for Technical‐Level Components

Appendix S5 shows GRADE certainty of evidence assessments. Figures 2, 3, 4, 5, 6, 7 show the estimates of components at technical level compared to minimal education. All estimates compared each technical‐level component with minimal education. Parental involvement (MD, −0.09; 95% CI, −0.16 to −0.03; moderate certainty) and stimulus control (MD, −0.07; 95% CI, −0.12 to −0.01; moderate certainty) probably reduce BMI z‐score, whereas relaxation training (MD, 0.16; 95% CI, 0–0.32; moderate certainty) and contracting (MD, −0.05; 95% CI, −0.12–0.03; moderate certainty) probably have no effect on BMI z‐score. Device monitoring (MD, 1.46 cm; 95% CI, 0.2–2.72 cm; low certainty) possibly promotes height increase, whereas cognitive restructuring (MD, −5.59 cm; 95% CI, −9.78 to −1.4 cm; low certainty) possibly suppresses height increase.

In terms of body fat percentage, preplanning (MD, −3.05%; 95% CI, −5.82% to −0.28%; moderate certainty) and feedback (MD, −2.73%; 95% CI, −5.31% to −0.14%; moderate certainty) probably reduce body fat percentage. Device monitoring (MD, 3.69%; 95% CI, 0.56%–6.83%; moderate certainty), problem‐solving (MD, 2.9%; 95% CI, 0.3%–5.5%; moderate certainty), and rule‐setting (MD, 4.32%; 95% CI, 0.16%–8.47%; moderate certainty) probably increase body fat percentage. Although it also suggests that relaxation training (MD, 8.08%; 95% CI, 0.82%–15.34%; moderate certainty) probably increases body fat percentage with important difference, this should be taken cautiously due to the limited evidence from only two trials [36, 37]. Cognitive restructuring (MD, −3.67%; 95% CI, −7.94%–0.61%; moderate certainty), stoplight approach (MD, −1.38%; 95% CI, −5.62%–2.86%; moderate certainty), psychoeducation (MD, −1.09%; 95% CI, −3.56%–1.38%; moderate certainty), parental involvement (MD, −0.55%; 95% CI, −2.85%–1.74%; moderate certainty), goal‐setting (MD, 0.26%; 95% CI, −1.99%–2.5%; moderate certainty), group intervention (MD, −0.58%; 95% CI, −0.58%–2.76%; moderate certainty), self‐concept improvement (MD, 1.2%; 95% CI, −1.73%–4.14%; moderate certainty), reminders (MD, 1.44%; 95% CI, −1.38%–4.26%; moderate certainty), and stress management (MD, 2.7%; 95% CI, −0.63%–6.03%; moderate certainty) probably have no effect on body fat percentage. Feedback (SMD, −0.77; 95% CI, −1.34 to −0.2; low certainty) and role‐playing (SMD, −0.76; 95% CI, −1.5 to −0.03; low certainty) possibly impair quality of life with important difference. Relaxation training (SMD, −1.51; 95% CI, −3.54–0.51; moderate certainty) and feedback (SMD, −0.24; 95% CI, −1.66–1.18; moderate certainty) probably have no effect on mental health. Appendix S4 provides additional details. However, many treatment components and their combinations were underpowered in this analysis showing low to very low certainty, thus calling for further trustworthy studies in the future.

### 3.6 Effect Estimates for Treatment Combination

**FIGURE 8:**
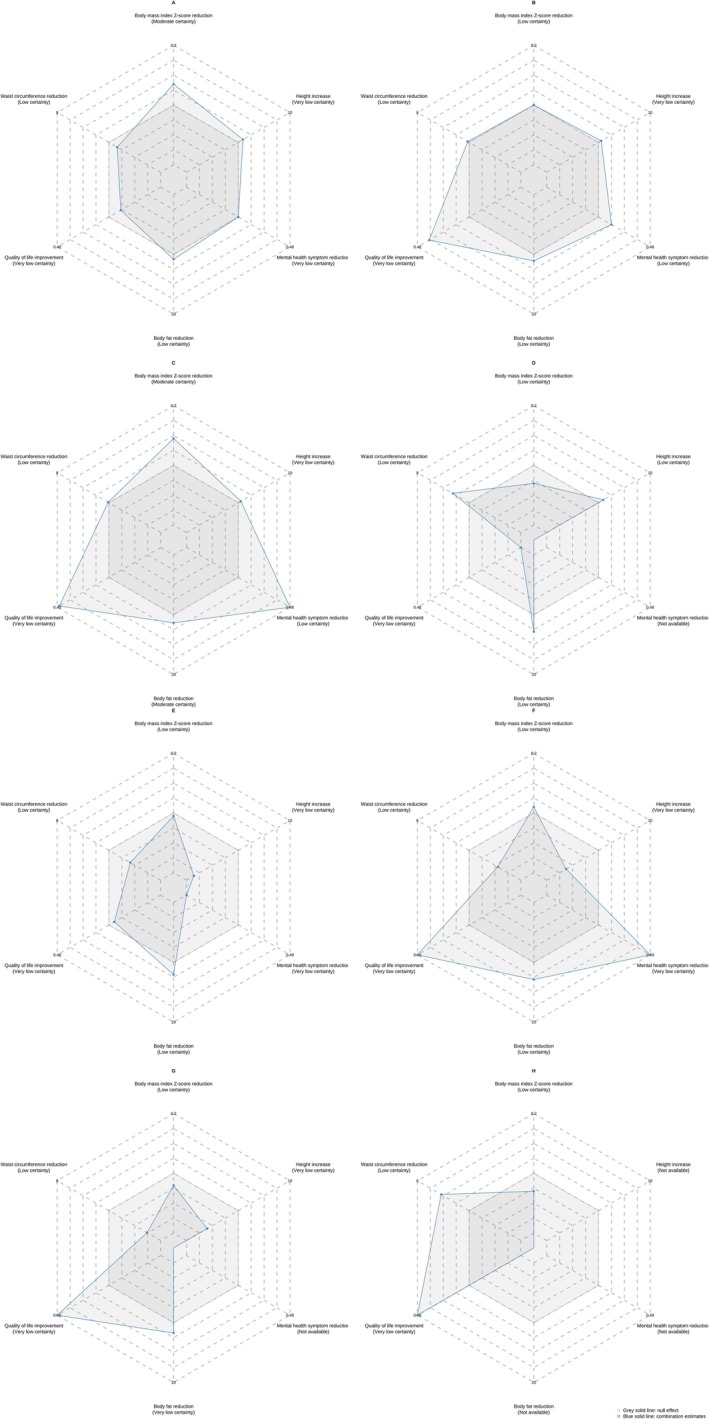
Radar chart of effect estimates for combinations. *Note:* In BMI z‐score, fat percentage, waist circumference, and mental health, an effect size of less than 0 shows that the component is favored. In height and quality of life, an effect size of more than 0 shows that the component is favored.

**FIGURE 9:**
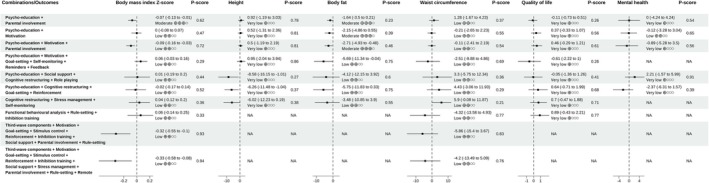
Forest plot of effect estimates for combinations. *Note:* In BMI z‐score, fat percentage, waist circumference, and mental health, an effect size of less than 0 shows that the component is favored. In height and quality of life, an effect size of more than 0 shows that the component is favored.

Considering the varying combinations of intervention components in clinical practice, there may be recommended interventions according to the following three conditions: (1) combinations most frequently used in included studies; (2) expert‐recommended; (3) data driven, with concerns of the estimates of the components in the seven targeted outcomes. We conducted efficacy estimations at the intervention level according to the aforementioned component analysis results (Figures 2, 3, 4, 5, 6, 7). Figures 8 and 9 show the effect estimates of the 10 combinations on each outcome. The most frequently used combination (i.e., psychoeducation + parental involvement) probably reduces BMI z‐score (MD, −0.07; 95% CI, −0.13 to −0.01; moderate certainty) and has no effect on body fat percentage (MD, −1.64%; 95% CI, −3.5%–0.21%; moderate certainty). Another one (i.e., psychoeducation + motivation + parental involvement) possibly reduces BMI z‐score (MD, −0.09; 95% CI, −0.16 to −0.03; low certainty) and probably reduces body fat percentage (MD, −2.71%; 95% CI, −4.93% to −0.48%; moderate certainty). The two data‐driven combinations formulated in our study (i.e., third‐wave components + motivation + goal‐setting + stimulus control + reinforcement + inhibition training + social support + parental involvement + rule‐setting, third‐wave components + motivation + goal‐setting + stimulus control + reinforcement + inhibition training + social support + stress management + parental involvement + rule‐setting + remote) achieve favorable results in reducing BMI z‐score. Other interventions demonstrate uncertain or little effects on weight‐related outcomes.

### 3.7 Sensitivity and Subgroup Analysis

We did not identify any credible subgroup effects (Appendix S6). The results of the four sensitivity analyses were consistent with those of our primary analyses, suggesting the robustness of our primary results (Appendix S7). However, in terms of absolute change of BMI, the effective components seemed to differ with those in BMI z‐score. Preplanning (MD, −1.06; 95% CI, −1.98 to −0.14; moderate certainty) and self‐monitoring (MD, −0.59; 95% CI, −1.15 to −0.03; moderate certainty) probably reduce BMI. Remote technique probably has little or no effect on BMI (MD, 0.07; 95% CI, −0.51–0.65; moderate certainty), whereas device monitoring (MD, 1.04; 95% CI, 0.41–1.67; moderate certainty) probably increases BMI. Appendix S8 provides further details.

## 4 Discussion

This component NMA retrieved 125 RCTs addressing CBT for obesity management that included 16,513 children and adolescents with overweight or obesity. This is the most comprehensive evidence synthesis on the topic. Incorporating a novel approach of meta‐analysis and a multidisciplinary team informing the treatment component taxonomy, this study for the first time estimated outcome values for each component of CBT for obesity management separately. In general, behavioral therapy and CBT probably have small or very small effects in reducing BMI z‐score, waist circumference, and body fat percentage and improving quality of life, whereas psychoeducation and cognitive therapy alone may not. For specific CBT techniques, parental involvement, stimulus control, preplanning, and feedback probably reduce BMI z‐score and body fat percentage.

CBT focuses on behavioral training by shaping daily habits. Children and adolescents undergo rapid physical and mental development. Imbalanced growth of body fat mass is usually a bidirectional cause of mental health issues [38]. It means that routine dietary or exercise prescription may not result in lifestyle changes in these young people. CBT has long been investigated and implemented for obesity management [39, 40], although its implementation seldom considers an anatomy of detailed treatment components. For the conceptual‐level components, our results identified effectiveness in behavioral therapy, CBT, and psychoeducation but not in cognitive therapy alone, which is consistent with previous findings in adults [11, 32, 41, 42, 43]. Previous reviews suggested the effectiveness of CBT in weight management in adult participants [11, 43], yet our study did not come up with similar findings in children and adolescents due to limited evidence. CBT was found to have a promising effect in immersion treatment where young people were placed in a therapeutic and educational environment for an extended period of time (i.e., camps and residential programs) [44]. However, in our review, all the included studies were mostly conducted in outpatient settings; little effect of CBT was found in weight control.

Our study found that CBT probably results in growth suppression compared to minimal education, which represents a potential harm for weight‐lowering therapy for children and adolescents. Like other weight‐lowering treatments, CBT reduces body weight by restricting calorie intake, when protein and other essential nutrients may be insufficient for children and adolescents with rapid growth during prepuberty or puberty period. This fact once again highlights the importance of dietary consultation during and after the weight‐lowering program.

Unlike with adults, the developing central neural system and limited social experience restricted self‐control among children and adolescents during weight loss. Although young people are in general quick to acquire new knowledge and skills, those at rebellious phases may have more difficulties following cognitive reconstruction compared to adults. In our systematic review, behavioral therapy rather than cognitive therapy showed benefits for children and adolescents with overweight or obesity. This finding is in line with previous analyses [18, 45] and confirms that clinicians and psychologists may consider behavioral therapy rather than cognitive therapy when treating children and adolescents with overweight or obesity.

In our analysis, parental involvement was one of the most effective components for obesity management, which highlights the important role parents play in obesity management. They foster changes in lifestyles and facilitate CBT delivery [46]. Some trials showed other benefits, including improved health outcomes in parents [47, 48]. Stimulus control, feedback, and preplanning also showed effectiveness in reducing BMI z‐score or body fat mass in different magnitudes, which is consistent with previous studies [49, 50, 51]. These techniques are easy to implement and can be delivered by trained psychotherapists.

We observed four technical‐level components (problem‐solving, rule‐setting, device monitoring, and relaxation training) probably increased body fat percentage with moderate certainty. Although the effect sizes for problem‐solving, rule‐setting, and device monitoring did not exceed the MID of 5%, clinicians should be cautious in considering these techniques in practice. Improper problem‐solving and rule‐setting may compromise treatment and eliminate motivation for obesity treatment. Observational studies are necessary for validation in certain contexts. The underlying mechanism can be complicated. For example, relaxation training (e.g., meditation and deep breathing) help reduce basal metabolic rates when releasing anxiety.

Device monitoring, typically step counters using accelerometers, is emerging technology with suggested benefits for weight loss in adults with obesity [52, 53]. As a surrogate parameter for physical activity, step counting may be helpful in people with strong motivation; otherwise, it is easily misused by some cheating devices (adding step counts without real exercise). The accuracy of monitoring reflecting real lifestyle is important for the success of weight management of children and adolescents. Improper use of step counts may mislead the behavioral feedback and potential reward in the CBT. Clinicians and parents need to be cautious with the information obtained from monitoring devices.

With complementary interaction among cognitive therapy and behavioral therapy, CBT has emerged as an effective strategy for managing obesity. One primary mechanism is the modification of maladaptive cognitions related to eating and weight by modifying the antecedent stimuli and consequent outcomes of behaviors, thereby breaking the vicious cycle of “environmental triggers‐automated behaviours‐negative reinforcement” [54]. By correcting patients' excessive cravings for food, overemphasis on weight, and negative cognitions regarding weight loss failure, CBT helps individuals develop healthier thought patterns and reduce emotional eating [55]. This cognitive modification is complemented by the enhancement of self‐regulation skills, such as self‐monitoring and goal‐setting, which empower individuals to take control of their eating behaviors and physical activity levels [56]. Additionally, CBT focuses on improving self‐efficacy and intrinsic motivation, which are crucial for long‐term adherence to weight management strategies [57]. What is more, according to the neurobehavioral mechanisms of temptation, it is possible that conditional reinforcement, stimulus control and exposure therapy together with inhibition training help to handle the reward system and adopt a healthier lifestyle habit [11, 58]. Our research suggested that stimulus control, reinforcement, preplanning, and cognitive restructuring may serve as effective CBT techniques. For instance, stimulus control directly targets antecedents by reducing environmental cues that trigger maladaptive behaviors (e.g., removing snacks and staying away from smoking areas) and increasing cues for target behaviors (e.g., placing workout clothes beside the bed). This approach reduces the cognitive load required to initiate behaviors (minimizing “willpower expenditure”) and creates space for conscious choices (e.g., “I can choose to drink water now”) by diminishing automated responses (e.g., “eating snacks upon seeing them”), thereby reinforcing self‐efficacy. Contingency management utilizes immediate external reinforcement (e.g., reward tokens and check‐ins) or intrinsic reinforcement (e.g., the sense of pleasure after exercising) to modify the consequent outcomes (consequences) of behaviors.

Previous reviews pointed out that cognitive processes play a crucial role in weight control and dysregulated eating habits, in which autonomous motivation, self‐efficacy/barriers, self‐regulation skills (such as self‐monitoring), flexible eating restraint, and positive body image proved to be psychological or behavioral mediators for positive weight control [56, 59, 60]. By setting achievable goals and celebrating small successes, individuals gain confidence in their ability to maintain healthy behaviors. CBT also addresses psychological barriers such as anxiety, depression, and negative body image, which can undermine efforts to adopt healthier lifestyles and to lose weight [55, 61]. CBT helps individuals develop a more resilient mindset toward obesity management by fostering a positive body image and reducing the psychological distress associated with the disease. The comprehensive cognitive behavioral approaches not only promote weight loss but also enhance the overall psychological well‐being of individuals, making CBT a potent choice in the multidisciplinary management of obesity.

Our study has limitations. First, both waiting list and non‐waiting list minimal education may differ in effect size but were considered as the same control group in the analyses to avoid information overload. A sensitivity analysis confirmed the robustness of study findings by splitting them into two groups. Second, some included trials are at high risk of bias; the sensitivity analysis excluding them confirmed the results of these trials were similar to those at low risk of bias. Third, with many treatment components and their combinations, the NMA proved underpowered for many comparisons. Therefore, there is a need for further trials with different combinations of treatment components selected based on existing evidence. Fourth, most trials were conducted in high‐income countries, which limits the applicability of our findings in low‐middle‐income countries (LMICs). Qualitative and health systems reviews indicate that trained CBT providers are scarce and that routine reimbursement is uncommon in these settings [62, 63]. Consequently, the pooled effect sizes may not reflect the potential impact in LMICs where CBT is not yet embedded in primary care. Nevertheless, novel techniques such as remote approaches may improve equity by raising the feasibility and applicability of CBTs across societies with health delivery capacities disparity [64].

In conclusion, behavioral therapy and CBT are possibly effective for obesity management in children and adolescents by reducing BMI z‐score and improving clinically important quality of life. Some specific techniques such as parental involvement, stimulus control, preplanning, and feedback showed potential benefits and should be prioritized in CBT studies and interventions. Other treatment components such as device monitoring and relaxation training provided no suggestion of potential benefit. Low‐cost techniques such as self‐monitoring and stimulus control showing beneficial are worthy of spreading, especially in low‐resource settings. Although this study suggested some treatment combinations based on the anticipation using the data, further study in comparative trials is necessary to inform clinical practice.

## Author Contributions

S.L. and X.X. conceived and designed the study. X.X., S.L., W.X., L.Z., J.L., and X.H. devised the taxonomy of CBT. X.X., M.S., Y.Z., and J.M. screened and selected the articles. X.X., M.S., Y.G., JM, YC, YM, J.G., J.Z., and M.Z. extracted the data. X.Z. and Y.G. assessed the risk of bias. X.X., Y.M., and X.Z. analyzed the data. S.L. supervised the data analyses. Y.M. and Q.Y. rated the certainty of evidence. X.X. and S.L. interpreted the data and drafted the manuscript. Y.M., X.Z., M.S., Y.Y., L.K.‐D., A.A., Y.B., J.A., K.N., B.S., C.Y., C.Q., and G.G. contributed to revising the manuscript. All authors had full access to all the data in the study and had final responsibility for the decision to submit for publication.

## Funding

The Department of Nutrition and Food Safety at the World Health Organization (WHO) commissioned and provided financial support for this work.

## Conflicts of Interest

The authors declare no conflicts of interest.

## Supporting information


**Appendix S1:** Supplement to methods.
**Appendix S2:** List of included studies.
**Appendix S3:** Risk of bias assessments.
**Appendix S4:** Supplement to results.
**Appendix S5:** GRADE certainty of evidence assessments.
**Appendix S6:** Subgroup analyses.
**Appendix S7:** Sensitivity analyses.
**Appendix S8:** Results of BMI.
**Appendix S9:** Reference list for included studies.

## Data Availability

Data and the analytic code are available on reasonable request from the corresponding author after completion of a data‐use agreement.
