# Supplementary material for: Cognitive Behavioral Interventions for Children and Adolescents With Overweight or Obesity: A Systematic Review and Component Network Meta‐Analysis
Source: Obes Rev. 2026 Mar 6;27(8):e70118. doi: 10.1111/obr.70118 (PMC13371829; doi:10.1111/obr.70118)
Supplement: Supplementary file 1 — Appendix S1: Supplement to methods. Appendix S2: List of included studies. Appendix S3: Risk of bias assessments. Appendix S4: Supplement to results. Appendix S5: GRADE certainty of evidence assessments. Appendix S6: Subgroup analyses. Appendix S7: Sensitivity analyses. Appendix S8: Results of BMI. Appendix S9: Reference list for included studies. [file OBR-27-e70118-s001.pdf]

Supplementary appendix

Title: Cognitive behavioural interventions for children and adolescents with overweight or obesity: a systematic review and component network meta-analysis

Authors: Xinran Xie[#1,2], Yunhe Mao[#3,4], Minghui Sun[#1,2], Xinyu Zou[#1,2], Yan Yang[#5], Yiyuan Gao[1,2], Wei Xu[6], Lan Zhang[7], Leticia Kawano-Dourado[8,9,10], Arnav Agarwal[10,11,12], Jiafeng Li[7], Xia Hong[13], Yufang Bi[14], Ying Liu[15], Jing An[1,2], Jiahui Ma[1,2], Yuzi Cao[1,2], Kailei Nong[1,2], Jiyun Guo[1,2], Jing Zeng[16,17], Mengnan Zhao[1,2], Yibin Zhang[5], Qinbo Yang[18], Baihai Su[18], Changzheng Yuan[19], Changjian Qiu[7], Gordon Guyatt[11], Sheyu Li[*1,2]

# co-first authors

1 Department of Endocrinology and Metabolism, West China Hospital, Sichuan University, Chengdu, China.

2 Department of Guideline and Rapid Recommendation, Cochrane China Centre, MAGIC China Centre, Chinese Evidence-Based Medicine Centre, West China Hospital, Sichuan University, Chengdu, China.

3 Sports Medicine Centre, West China Hospital, Sichuan University, Chengdu, China.

4 Department of Orthopaedics and Orthopaedic Research Institute, West China Hospital, Sichuan University, Chengdu, China.

5 Department of Endocrinology, Tongji Hospital, Tongji Medical College, Huazhong University of Science and Technology, Wuhan, China.

6 Faculty of Psychology, Beijing Normal University, Beijing, China.

7 Mental Health Centre, West China Hospital, Sichuan University, Chengdu, China.

8 Hcor Research Institute, Hcor Hospital, Sao Paulo, Brazil.

9 Pulmonary Division, Heart Institute (InCor), University of Sao Paulo, Brazil.

10 MAGIC Evidence Ecosystem Foundation, Oslo, Norway.

11 Department of Health Research Methods, Evidence and Impact, McMaster University, Hamilton, Ontario, Canada.

12 Division of General Internal Medicine, McMaster University, Hamilton, Ontario, Canada;

13 Department of Psychological Medicine, Peking Union Medical College Hospital, Beijing, China

14 Department of Psychiatry, Ruijin Hospital, Shanghai Jiao Tong University School of Medicine, Shanghai, China

15 Department of Paediatric Genetic, Metabolic, and Endocrine Disorders, West China Second University Hospital, Sichuan University, Chengdu, China.

16 Department of Obstetrics and Gynaecology, West China Second University Hospital, Sichuan University, Chengdu, China.

17 Key laboratory of Birth Defects and Related Diseases of Women and Children (Ministry of Education), West China Second University Hospital, Sichuan University, Chengdu, China.

18 Department of Nephrology, West China Hospital, Sichuan University, Chengdu, China.

19 School of Public Health, Zhejiang University, Hangzhou, China.

Corresponding author and e-mail address:

Sheyu Li, M.D.

Department of Endocrinology and Metabolism, MAGIC China Centre, West China Hospital of Sichuan University, Chengdu, China.

Email: lisheyu@gmail.com; lisheyu@scu.edu.cn

Phone: +86-13194874843

Fax: +86-28-85422982

Content

Appendix 1 Supplement to methods **5**
1.1 Search strategy 5
1.2 Definition of outcomes 8
1.3 Detailed methodology of handling multi-arm trials, variance structure and model fit assessment 9
1.4 Details of GRADE assessment methodology 11
Appendix 2 List of included studies **13**
Appendix 3 Risk of bias assessments **30**
Appendix 4 Supplement to results **39**
4.1 Network plots for the outcomes in conceptual level NMA 39
4.2 Network plots for the outcomes in technical level CNMA 46
4.3 Forest plots for the outcomes in conceptual level NMA 53
4.4 Forest plots for the outcomes in technical level CNMA 59
4.5 Pairwise forest plots for the outcomes in conceptual level NMA 69
4.6 Pairwise forest plots for the outcomes in technical level CNMA 76
4.7 Heterogeneity (Inconsistency) assessed by pairwise meta-analyses 89
4.8 Publication bias assessments 92
4.9 Intransitivity assessments 96
Appendix 5 GRADE certainty of evidence assessments **121**
5.1 Summary of findings table in conceptual level NMA 121
5.2 Summary of findings table in technical level CNMA 124
5.3 Percentage contribution matrix for conceptual level NMA 139
5.4 Percentage contribution matrix for technical level CNMA 145
Appendix 6 Subgroup analyses **151**
6.1 Subgroup analyses for the conceptual level NMA 151
6.2 Subgroup analyses for the technical level CNMA 155
Appendix 7 Sensitivity analyses **176**
7.1 Sensitivity analyses for conceptual level NMA 177
7.2 Sensitivity analyses for technical level CNMA 178
Appendix 8 Results of BMI **186**
8.1 Network plots 186
8.2 Forest plots 188
8.3 Heterogeneity (Inconsistency) assessed by pairwise meta-analyses 197
8.4 Publication bias assessments 199
8.5 Intransitivity assessments 200
8.6 GRADE certainty of evidence assessments 204
8.7 Subgroup analyses 209
8.8 Sensitivity analyses 215
Appendix 9 Reference list for included studies **217**

Appendix 1 Supplement to methods

1.1 Search strategy

Embase <1974 to 2024 July 16>

1 exp Obesity/ 706632

2 exp Pediatric Obesity/ or exp Obesity Management/ 24786

3 exp Overweight/ 706632

4 exp Weight Loss/ 104376

5 (obes* or "body mass ind*" or adiposity or overweight or "over weight" or "overload syndrome*" or "over eat*" or overfeed* or "over feed*" or overfed or "over fed" or "weight cycling" or "skinfold thickness" or antiobesity or "anti obesity" or obesitas or bodyweight or "body weight").mp. [mp=title, abstract, heading word, drug trade name, original title, device manufacturer, drug manufacturer, device trade name, keyword heading word, floating subheading word, candidate term word] 1562352

6 1 or 2 or 3 or 4 or 5 1610104

7 exp Child, Preschool/ 641944

8 exp Child/ 3219719

9 exp Adolescent/ 1854216

10 (adolescen* or youth or youths or teen or teens or teenage* or children).mp. [mp=title, abstract, heading word, drug trade name, original title, device manufacturer, drug manufacturer, device trade name, keyword heading word, floating subheading word, candidate term word] 3167679

11 7 or 8 or 9 or 10 4506336

12 exp Randomized Controlled Trial/ 833494

13 exp Double-Blind Method/ 221104

14 ("randomized controlled study" or "randomized controlled trial" or "randomized study" or "randomized trial" or "randomized pl acebo controlled study" or "randomized placebo controlled trial" or "randomized placebo controlled" or "randomized placebo controlled" or "randomized double blin*" or "randomized double blin*").mp. [mp=title, abstract, heading word, drug trade name, original title, device manufacturer, drug manufacturer, device trade name, keyword heading word, floating subheading word, candidate term word] 1202809

15 12 or 13 or 14 1240761

16 exp Psychotherapy/ 310534

17 exp Behavior Therapy/ 81129

18 exp Cognitive Behavioral Therapy/ 32203

19 (psychotherap* or CBT or CBTI or CBT-I or "behavio?r* therap*" or "behavio?r* treatment*" or "cogniti* therap*" or "cogniti* treatment*" or "behavio?r* modification*" or "behavior change technique*" or "psychodynamic" or "psychoanalysis" or "psychoanalytic*" or "counsel?ing" or "problem-solving" or "mindfulness" or "assertiveness training" or "behavior* activation" or "acceptance and commitment therapy" or "cognitive restructuring" or "metacognitive therap*" or "metacognitive treatment*" or "meta-cognitive therap*" or "solution focused therap*" or "solution-focused therap*" or "self-control" or "self control" or "goal setting" or reinforcement or preplanning).mp. [mp=title, abstract, heading word, drug trade name, original title, device manufacturer, drug manufacturer, device trade name, keyword heading word, floating subheading word, candidate term word] 676777

20 16 or 17 or 18 or 19 755747

21 6 and 11 and 15 and 201478

EBM Reviews - Cochrane Central Register of Controlled Trials <June 2024>

1 exp Obesity/ 21491

2 exp Pediatric Obesity/ or exp Obesity Management/ 4044

3 exp Overweight/ 25242

4 exp Weight Loss/9103

5 (obes* or "body mass ind*" or adiposity or overweight or "over weight" or "overload syndrome*" or "over eat*" or overfeed* or "over feed*" or overfed or "over fed" or "weight cycling" or "skinfold thickness" or antiobesity or "anti obesity" or obesitas or bodyweight or "body weight").mp. [mp=title, original title, abstract, floating sub-heading word, mesh headings, heading words, keyword] 142757

6 1 or 2 or 3 or 4 or 5 144097

7 exp Child, Preschool/ 39275

8 exp Child/ 81000

9 exp Adolescent/ 136705

10 (adolescen* or youth or youths or teen or teens or teenage* or children).mp. [mp=title, original title, abstract, floating sub-heading word, mesh headings, heading words, keyword] 275484

11 7 or 8 or 9 or 10 284054

12 exp Randomized Controlled Trial/ 37

13 exp Double-Blind Method/ 171391

14 ("randomized controlled study" or "randomized controlled trial" or "randomized study" or "randomized trial" or "randomized pl acebo controlled study" or "randomized placebo controlled trial" or "randomized placebo controlled" or "randomized placebo controlled" or "randomized double blin*" or "randomized double blin*").mp. [mp=title, original title, abstract, floating sub-heading word, mesh headings, heading words, keyword] 867404

15 12 or 13 or 14 946876

16 exp Psychotherapy/ 35253

17 exp Behavior Therapy/ 24748

18 exp Cognitive Behavioral Therapy/ 14011

19 (psychotherap* or CBT or CBTI or CBT-I or "behavio?r* therap*" or "behavio?r* treatment*" or "cogniti* therap*" or "cogniti* treatment*" or "behavio?r* modification*" or "behavior change technique*" or "psychodynamic" or "psychoanalysis" or "psychoanalytic*" or "counsel?ing" or "problem-solving" or "mindfulness" or "assertiveness training" or "behavior* activation" or "acceptance and commitment therapy" or "cognitive restructuring" or "metacognitive therap*" or "metacognitive treatment*" or "meta-cognitive therap*" or "solution focused therap*" or "solution-focused therap*" or "self-control" or "self control" or "goal setting" or reinforcement or preplanning).mp. [mp=title, original title, abstract, floating sub-heading word, mesh headings, heading words, keyword] 96409

20 16 or 17 or 18 or 19 104967

21 6 and 11 and 15 and 201242

Ovid MEDLINE(R) and Epub Ahead of Print, In-Process, In-Data-Review & Other Non-Indexed Citations, Daily and Versions <1946 to July 16, 2024>

1 exp Obesity/ 273416

2 exp Pediatric Obesity/ or exp Obesity Management/ 50590

3 exp Overweight/ 285148

4 exp Weight Loss/51712

5 (obes* or "body mass ind*" or adiposity or overweight or "over weight" or "overload syndrome*" or "over eat*" or overfeed* or "over feed*" or overfed or "over fed" or "weight cycling" or "skinfold thickness" or antiobesity or "anti obesity" or obesitas or bodyweight or "body weight").mp. [mp=title, book title, abstract, original title, name of substance word, subject heading word, floating sub-heading word, keyword heading word, organism supplementary concept word, protocol supplementary concept word, rare disease supplementary concept word, unique identifier, synonyms, population supplementary concept word, anatomy supplementary concept word] 1004960

6 1 or 2 or 3 or 4 or 5 1031033

7 exp Child, Preschool/ 1006629

8 exp Child/ 2215533

9 exp Adolescent/ 2260371

10 (adolescen* or youth or youths or teen or teens or teenage* or children).mp. [mp=title, book title, abstract, original title, name of substance word, subject heading word, floating sub-heading word, keyword heading word, organism supplementary concept word, protocol supplementary concept word, rare disease supplementary concept word, unique identifier, synonyms, population supplementary concept word, anatomy supplementary concept word] 3214716

11 7 or 8 or 9 or 10 3854168

12 exp Randomized Controlled Trial/ 619262

13 exp Double-Blind Method/ 179545

14 ("randomized controlled study" or "randomized controlled trial" or "randomized study" or "randomized trial" or "randomized pl acebo controlled study" or "randomized placebo controlled trial" or "randomized placebo controlled" or "randomized placebo controlled" or "randomized double blin*" or "randomized double blin*").mp. [mp=title, book title, abstract, original title, name of substance word, subject heading word, floating sub-heading word, keyword heading word, organism supplementary concept word, protocol supplementary concept word, rare disease supplementary concept word, unique identifier, synonyms, population supplementary concept word, anatomy supplementary concept word] 715311

15 12 or 13 or 14 748448

16 exp Psychotherapy/ 224984

17 exp Behavior Therapy/ 93273

18 exp Cognitive Behavioral Therapy/ 38731

19 (psychotherap* or CBT or CBTI or CBT-I or "behavio?r* therap*" or "behavio?r* treatment*" or "cogniti* therap*" or "cogniti* treatment*" or "behavio?r* modification*" or "behavior change technique*" or "psychodynamic" or "psychoanalysis" or "psychoanalytic*" or "counsel?ing" or "problem-solving" or "mindfulness" or "assertiveness training" or "behavior* activation" or "acceptance and commitment therapy" or "cognitive restructuring" or "metacognitive therap*" or "metacognitive treatment*" or "meta-cognitive therap*" or "solution focused therap*" or "solution-focused therap*" or "self-control" or "self control" or "goal setting" or reinforcement or preplanning).mp. [mp=title, book title, abstract, original title, name of substance word, subject heading word, floating sub-heading word, keyword heading word, organism supplementary concept word, protocol supplementary concept word, rare disease supplementary concept word, unique identifier, synonyms, population supplementary concept word, anatomy supplementary concept word] 476816

20 16 or 17 or 18 or 19 542460

21 6 and 11 and 15 and 201196

1.2 Definition of outcomes

Outcome	Definition	Effect measure
BMI change from baseline	Treatment-end BMI minus baseline BMI	Mean difference
BMI z-score change from baseline	Treatment-end BMI z-score minus baseline BMI z-score	Mean difference
Height change from baseline	Treatment-end height minus baseline height	Mean difference
Body fat percentage (%) change from baseline	Treatment-end body fat percentage minus baseline body fat percentage	Mean difference
Waist circumference change from baseline	Treatment-end waist circumference minus baseline waist circumference	Mean difference
Quality of life	Treatment-end quality-of-life score minus baseline quality-of-life score<br>Quality-of-life score could be measured by any type of scales	Standardised mean difference
Mental health	Treatment-end mental health score minus baseline mental health score<br>Mental health score could be measured by any type of scales	Standardised mean difference

1.3 Detailed methodology of handling multi-arm trials, variance structure and model fit assessment

Handling multi-arm trials, variance structure, and model fit assessment follows the frequentist framework for CNMA[1] implemented in the R package *netmeta*. Below we clarify each aspect:

1. Multi-arm trials:

Multi-arm trials are incorporated via the weight matrix W in the weighted least squares regression. Each multi-arm study with p arms contributes $\frac{\mathrm{p(p-1)}}{2)}$ treatment comparisons to the design matrix X. To avoid double-counting, the weights w_j in the diagonal matrix W are adjusted such that:

For two-arm studies: $w_j = \frac{1}{Var(d_j)}$

For multi-arm studies: weights are scaled to account for correlated comparisons within the same trial, as per Rücker & Schwarzer (2014). This ensures valid covariance estimation: $\boldsymbol{\hat{\delta}} = \boldsymbol{X}(\boldsymbol{X^T W X})^{+}\boldsymbol{X^T W d}$

2. Variance structure:

The variance-covariance matrix $\boldsymbol{\Sigma}$ of residuals ϵ is derived from $\mathbf{W^{-1}}$, assuming known sampling variances. For random-effects CNMA, the between-study variance τ^2 is estimated via a multivariate method of moments (Jackson et al., 2012) as:

$$\hat{\tau}^2 = \max\left(\frac{Q_a - df_a}{\mathrm{tr}\left((\boldsymbol{I} - \boldsymbol{H_a})\boldsymbol{UW}\right)}, 0\right)$$

where $\boldsymbol{H_a}$ is the hat matrix, $\boldsymbol{U}$ is a block-diagonal matrix based on $0.5\boldsymbol{BB}^{\top}$ (adjusted per multi-arm study), and Q_a is the heterogeneity statistic. This $\hat{\tau}^2$ is added to observed sampling variances before model refitting.

3. Model fit assessment:

Heterogeneity: Quantified by Cochran's Q statistic for the standard NMA model and CNMA model $Q_a = (\boldsymbol{d} - \widehat{\boldsymbol{\delta}_a})^{\top}\boldsymbol{W}(\boldsymbol{d} - \widehat{\boldsymbol{\delta}_a})$.

Additivity test: The assumption of no component interactions is tested via the difference $Q_a - Q = \left(\widehat{\boldsymbol{\delta}_a} - \widehat{\boldsymbol{\delta}^{\mathrm{nma}}}\right)^{\top} W\left(\widehat{\boldsymbol{\delta}_a} - \widehat{\boldsymbol{\delta}^{\mathrm{nma}}}\right)$, which follows a χ^2 distribution with $n - r - 1$ degrees of freedom under $\boldsymbol{H}_0$.

Model comparison: For interaction CNMA models, nested Q-tests (e.g., additive vs. interaction model) evaluate whether added interaction terms improve fit, $Q_{\mathrm{int}} - Q_a \sim \chi^2(\Delta\mathrm{df})$

Reference

1. Rücker G, Petropoulou M, Schwarzer G. Network meta-analysis of multicomponent interventions. *Biometrical journal Biometrische Zeitschrift* 2020;62(3):808-21. doi: 10.1002/bimj.201800167 [published Online First: 2019/04/26]

1.4 Details of GRADE assessment methodology

The study adapted the GRADE framework to systematically assess the certainty of evidence of the component network meta-analysis (CNMA), prioritising a stepwise evaluation that began with direct evidence and integrated additive model assumptions. The process starts by assessing direct pairwise comparisons using the same GRADE approaches in standard network meta-analysis[1 2]. Specifically, (1) each study contributing to direct evidence is evaluated using the Cochrane ROB 2.0 tool, with downgrades applied for risk of bias; (2) comparisons require Q-test $p > 0.1$ and $I^2 < 50\%$ to avoid downgrading in heterogeneity; (3) indirectness judged through boxplots to detect basic characters mismatches relative to the target PICO; (4) publication is tested via funnel plot asymmetry (Egger's test) or trim-and-fill analysis for directional changes.

Once direct evidence is rated, the evidence contribution matrix was applied to quantify the influence of each direct comparison on component effect estimates. This matrix was constructed through a 'leave-one-out' algorithm: individual component effects $\boldsymbol{\beta}_{\text{full}}$ and their standard deviations $\boldsymbol{sd}_{\text{full}}$ were first obtained using the '*netcomb*' function in R package *netmeta*, in which $\boldsymbol{\beta}_{\text{full}}$ are estimated using all available direct comparisons and weighted by inverse variance[3]. Each direct comparison is then iteratively excluded, and the component effects $\boldsymbol{\beta}_{\boldsymbol{new}}^{(\boldsymbol{i})}$ and their standard deviations $\boldsymbol{sd}_{\boldsymbol{new}}^{(\boldsymbol{i})}$ are recalculated. The absolute difference ($\boldsymbol{\Delta}_{\text{abs}}[j, i]$) between full and leave-one-out estimates for each component j was computed as $\boldsymbol{\Delta}_{\text{abs}}[j, i] = \left|\mathbf{sd}_{\text{full}}[j] - \mathbf{sd}_{\text{new}}^{(i)}[j]\right|$, and these differences were normalised to percentage contributions.

The initial GRADE rating followed a 50% majority rule, where rating is determined based on the combined contributions of evidence certainty levels: if the combined contribution from high- and moderate-certainty evidence exceeds 50%, the rating is high if high-certainty evidence alone accounts for > 50% of contributions, or moderate if high- certainty evidence is ≤50 %. Conversely, if low- or very low-certainty contributions dominate (≥50%), the rating is low if very low-certainty evidence is <50%, or very low if very low-certainty evidence accounts for ≥50%.

The validity of additive CNMA estimates was validated through in-additivity and intransitivity checks[4 5]. A Q-test compares additive estimates against standard NMA results; significant discrepancies ($p < 0.05$) indicate violations of the additivity assumption (e.g., unmodeled component interactions) and trigger a one-level downgrade. Transitivity was assessed by examining the distribution of effect modifiers and methodological heterogeneity across comparisons. Components with sparse direct evidence rely more on additive network estimates, which are further decomposed into direct $\boldsymbol{\theta}_{direct}^{(j)}$ and additive $\boldsymbol{\theta}_{add}^{(j)}$ contributions using the equation $\boldsymbol{\beta}_{\text{full}}^{(j)} =$

$\phi^{(j)}_{\text{direct}}\theta^{(j)}_{\text{direct}} + \phi^{(j)}_{\text{add}}\theta^{(j)}_{\text{add}}$, where $\phi^{(j)}_{\text{direct}}$ and $\phi^{(j)}_{\text{add}}$ reflects the proportional weight of direct estimate and additive estimate respectively.

The final certainty rating incorporated adjustments for incoherence and imprecision[6]. Incoherence was evaluated through standardised difference tests between direct and additive estimates ($p < 0.05$ triggering a one-level downgrade), such as a direct estimate of 0.8 (95% CI: 0.6~1.0) conflicting with an additive estimate of -0.5 (-0.7~-0.3). Imprecision was assessed differently based on clinical utility: standalone components use minimal important differences (MID), we rated down for imprecision by two levels when 95% CI crossed more than one threshold of importance[7]. Combination-dependent components relied on the null effect line (effect crossing zero).

Reference

1. Izcovich A, Chu DK, Mustafa RA, et al. A guide and pragmatic considerations for applying GRADE to network meta-analysis. *BMJ (Clinical research ed)* 2023;381:e074495. doi: 10.1136/bmj-2022-074495 [published Online First: 2023/06/28]
2. Balshem H, Helfand M, Schünemann HJ, et al. GRADE guidelines: 3. Rating the quality of evidence. *Journal of clinical epidemiology* 2011;64(4):401-6. doi: 10.1016/j.jclinepi.2010.07.015 [published Online First: 2011/01/07]
3. Rücker G, Petropoulou M, Schwarzer G. Network meta-analysis of multicomponent interventions. *Biometrical journal Biometrische Zeitschrift* 2020;62(3):808-21. doi: 10.1002/bimj.201800167 [published Online First: 2019/04/26]
4. Rücker G, Schwarzer G. Reduce dimension or reduce weights? Comparing two approaches to multi-arm studies in network meta-analysis. *Statistics in medicine* 2014;33(25):4353-69. doi: 10.1002/sim.6236 [published Online First: 2014/06/20]
5. Zeng L, Brignardello-Petersen R, Hultcrantz M, et al. GRADE guidelines 32: GRADE offers guidance on choosing targets of GRADE certainty of evidence ratings. *Journal of clinical epidemiology* 2021;137:163-75. doi: 10.1016/j.jclinepi.2021.03.026 [published Online First: 2021/04/16]
6. Puhan MA, Schünemann HJ, Murad MH, et al. A GRADE Working Group approach for rating the quality of treatment effect estimates from network meta-analysis. *BMJ (Clinical research ed)* 2014;349:g5630. doi: 10.1136/bmj.g5630 [published Online First: 2014/09/26]
7. Zeng L, Brignardello-Petersen R, Hultcrantz M, et al. GRADE Guidance 34: update on rating imprecision using a minimally contextualized approach. *Journal of clinical epidemiology* 2022;150:216-24. doi: 10.1016/j.jclinepi.2022.07.014 [published Online First: 2022/08/08]

Appendix 2 List of included studies

Study	Treatment duration (week)	Settings	Country/ region	Intervention components	Sample size	Proportion of girls	Age (year)	Baseline BMI (Kg/m^2)
Sánchez-López A M_2020	52	Medical centre	Spain	PSYE+PI+G	49	0.47	10.47	27.54
Sánchez-López A M_2020	52	Medical centre	Spain	PSYE+SG+PI+G	49	0.47	10.39	29.73
Sen_2018	12	Medical centre	Turkey	STOPLIGHT+PSYE+PS+GS+SM+RIF+PI+MODELING+RULE+G	12	0.42	9.91	25.36
Sen_2018	12	Medical centre	Turkey	STOPLIGHT+PSYE+PS+GS+RIF+SG+PI+MODELING+RULE+G	12	0.33	10.75	26.81
Cohen_2016	26	Research centre	Canada	STOPLIGHT+PSYE+MTV+PS+GS+CONTRACTING+SG+PI	50	0.58	7.9	24.65
Cohen_2016	26	Research centre	Canada	ME	28	0.57	7.7	24.1
Willeboordse_2016	78	Medical centre	the Netherlands	PSYE+CR+SCI+FBA+MTV+PS+GS+TASK+RMD+FB+RIF+SC+PRP+SS+SG+PI+MODELING+G	43	0.39	12.3	25
Willeboordse_2016	78	Medical centre	the Netherlands	ME	44	0.39	11.9	25.2
Abraham_2015	24	Medical centre	China	ME	16	0.38	14.53	30.27
Abraham_2015	24	Medical centre	China	PSYE+RMD+PRP+STRESS+RD	16	0.44	14.93	28.97
Abraham_2015	24	Medical centre	China	PSYE+PI	16	0.38	14.6	31.67
Berry_2014	78	At school	USA	PSYE+CR+SCI+MTV+PS+GS+DM+SS+SG+PI+G	184	0.55	9.2	
Berry_2014	78	At school	USA	ME	162	0.56	9	
Bohlin_2017	78	Medical centre	Sweden	PSYE+PS+RIF+PI+MODELING+RULE	19	0.47	9	25.7
Bohlin_2017	78	Medical centre	Sweden	PSYE+RIF+PI+MODELING+RULE+G	18	0.22	8.5	24.3
Boutelle_2017	26	Medical centre	USA	STOPLIGHT+PSYE+PS+GS+TASK+FB+RIF+SC+PRP+PI+MODELING	75	0.67	10.43	26.56
Boutelle_2017	26	Medical	USA	STOPLIGHT+PSYE+PS+	75	0.67	10.39	26.13

Study	Treatment duration (week)	Settings	Country/ region	Intervention components	Sample size	Proportion of girls	Age (year)	Baseline BMI (Kg/m^2)
		centre		GS+TASK+SM+FB+RIF+SC+PRP+PI+MODELING+G				
Broccoli_2016	52	Primary care setting	Italy	PSYE+MTV+GS+FB+PI	187			18.27
Broccoli_2016	52	Primary care setting	Italy	ME	185			18.21
Davis_2024	34	At school	USA	STOPLIGHT+PSYE+SCI+PS+GS+TASK+SM+RMD+RIF+PRP+PI+MODELING+G+RD	59			
Davis_2024	34	At school	USA	ME	83			
Davis_2013	34	At school	USA	STOPLIGHT+PSYE+SCI+GS+TASK+SM+RIF+ROLE+PI+MODELING+G+RD	31	0.29	8.48	
Davis_2013	34	At school	USA	ME	27	0.3	8.69	
Díaz_2010	52	Primary care setting	Mexico	STOPLIGHT+PSYE+SCI+FBA+GS+SS+PI+G	21	0.52	11.6	30.2
Díaz_2010	52	Primary care setting	Mexico	ME	22	0.5	11.7	29.1
Doyle_2008	16	Remote	USA	PSYE+CR+TWC+SCI+PS+GS+SM+DM+RMD+FB+SS+PI+RD	40	0.65	14.9	34.64
Doyle_2008	16	Remote	USA	ME	40	0.6	14.1	33.86
Epstein_2004	26	Research centre	USA	STOPLIGHT+PSYE+GS+SM+FB+CONTRACTING+SC+PRP+PI+RULE+G	30	0.6	9.9	
Epstein_2004	26	Research centre	USA	STOPLIGHT+PSYE+GS+SM+FB+RIF+CONTRACTING+PRP+PI+G	32	0.66	9.8	
Fedele_2018	42	Medical centre	USA	STOPLIGHT+PSYE+SCI+FBA+GS+SM+DM+FB+SC+SS+PRP+PI+MODELING+RULE+G	14	0.57	8.64	
Fedele_2018	42	Medical centre	USA	PSYE+RT+STRESS+G	10	0.5	8.7	
Freira_2018	26	At school	Portugal	PSYE+MTV	46	0.67	16.15	
Freira_2018	26	At school	Portugal	PSYE	51	0.73	15.77	
Garipağaoğlu_20	13	Medical	Turkey	PSYE+SCI+MTV+SC+PR	40	0.53	10.3	27.4

Study	Treatment duration (week)	Settings	Country/ region	Intervention components	Sample size	Proportion of girls	Age (year)	Baseline BMI (Kg/m²)
09		centre		P+PI				
Garipağaoğlu_2009	13	Medical centre	Turkey	PSYE+SCI+MTV+SC+PRP+PI+G	40	0.5	10.3	27.7
Golley_2007	26	Medical centre	Australia	PSYE+SCI+PS+GS+RIF+PRP+SS+PI+MODELING+RULE	38	0.63	8.2	
Golley_2007	26	Medical centre	Australia	PSYE+SCI+PS+GS+RIF+PRP+PI+MODELING+RULE	37	0.65	8.2	
Golley_2007	26	Medical centre	Australia	ME	36	0.64	8.2	
Hadley_2015	16	Medical centre	USA	PSYE+CR+GS+FB+PI+G	19	0.68	14.9	31.6
Hadley_2015	16	Medical centre	USA	PSYE+CR+GS+FB+PI+MODELING+G	19	0.84	15.2	32.9
Hidayanty_2016	12	At school	USA	PSYE+SCI+MTV+GS+FB+SG+PI+STRESS+G	84	0.63	13	
Hidayanty_2016	12	At school	USA	ME	88	0.66	13	
Jelalian_2011	16	Medical centre	Indonesia	PSYE+SCI+FBA+MTV+PS+GS+SM+RIF+CONTRACTING+SC+SS+SG+PI+G	45	0.66	14.45	31.49
Jelalian_2011	16	Medical centre	Indonesia	PSYE+FBA+MTV+GS+SM+RIF+CONTRACTING+SC+PI+G	44	0.69	14.19	31.33
Looney_2014	26	Primary care setting	USA	ME	8	37.5	7.3	
Looney_2014	26	Primary care setting	USA	PSYE+SM+FB+PI+RD	7	85.7	8.6	
Looney_2014	26	Primary care setting	USA	PSYE+GS+TASK+SM+FB+RIF+SC+PI+MODELING+RD	7	85.7	8.2	
Maddison_2014	24	At home	New Zealand	PSYE+SM+DM+RIF+SC+SG+PI+MODELING	127	0.43	11.2	26.51
Maddison_2014	24	At home	New Zealand	ME	124	0.44	11.3	26.62
Magarey_2011	26	Medical centre	Australia	PSYE+SCI+PS+GS+RIF+PRP+SS+PI+MODELING+RULE	85	0.55	8.2	
Magarey_2011	26	Medical	Australia	PSYE+SCI+SS+PI	84	0.56	8.2	

Study	Treatment duration (week)	Settings	Country/ region	Intervention components	Sample size	Proportion of girls	Age (year)	Baseline BMI (Kg/m^2)
		centre						
Marques_2023	12	Primary care setting	Brazil	PSYE+SM+SC+PRP+PI+G	27	0.43	14.24	28.89
Marques_2023	12	Primary care setting	Brazil	PSYE+SM+SC+PRP+G	29	0.64	13.23	30.77
Miri_2019	26	Medical centre	Iran	PSYE+CR+FBA+MTV+GS+TASK+SM+SC+PI+G	55	0.49	14.64	
Miri_2019	26	Medical centre	Iran	ME	55	0.45	14.88	
Nemet_2005	12	Medical centre	Israel	PSYE+MTV+TASK+SC+PRP+SG+PI+G	24	0.45	11.3	
Nemet_2005	12	Medical centre	Israel	ME	22	0.42	10.9	
Norman_2016_1	16	Primary care setting	USA	ME	25	1	11.8	28.9
Norman_2016_1	16	Primary care setting	USA	PSYE+SCI+PS+GS+SM+DM+FB+SS+PI+RD	29	1	12	29.8
Norman_2016_2	16	Primary care setting	USA	ME	28	0	11.7	28.9
Norman_2016_2	16	Primary care setting	USA	PSYE+SCI+PS+GS+SM+DM+FB+SS+PI+RD	24	0	12	29.4
O'Connor_2013	34	Primary care setting	USA	PSYE+PS+GS+SM+CONTRACTING+PI+RD	20	0.9	7.1	
O'Connor_2013	34	Primary care setting	USA	ME	20	0.7	6.6	
Patrick_2013	52	Remote	USA	STOPLIGHT+PSYE+SCI+GS+DM+RMD+FB+RIF+SS+PI+RD	26	0.62	14.1	
Patrick_2013	52	Remote	USA	STOPLIGHT+PSYE+SCI+PS+GS+DM+RMD+FB+RIF+SS+PI+RD	24	0.5	14.3	
Patrick_2013	52	Remote	USA	STOPLIGHT+PSYE+SCI+PS+GS+DM+FB+RIF+SS+PI+G+RD	26	0.69	14.3	
Patrick_2013	52	Remote	USA	ME	25	0.72	14.5	
Pbert_2016	34	At school	USA	ME	57	0.61	16.3	31.65
Pbert_2016	34	At school	USA	PSYE+CR+MTV+PS+GS+SM+DM+SC+PRP+SS+SG+G	54	0.63	16.5	30.66
Quattrin_2014	52	Medical	USA	PSYE+GS+FB+RIF+CON	46	0.67	4.6	20.4

Study	Treatment duration (week)	Settings	Country/ region	Intervention components	Sample size	Proportion of girls	Age (year)	Baseline BMI (Kg/m²)
		centre		TRACTING+SC+PRP+PI +MODELING				
Quattrin_2014	52	Medical centre	USA	PSYE+GS+FB+PI	50	0.66	4.4	20.1
Stark_2019	26	Medical centre, at home	USA	PSYE+PS+GS+DM+FB+ RIF+SC+ROLE+PI+MOD ELING+RULE+G	47	0.53	4.59	
Stark_2019	26	Medical centre, at home	USA	PSYE+PS+GS+PI	50	0.58	4.58	
Stark_2019	26	Medical centre, at home	USA	ME	54	0.59	4.61	
Taveras_2015	52	Primary care setting	USA	PSYE+MTV+PI	194	0.48	9.8	25.6
Taveras_2015	52	Primary care setting	USA	PSYE+MTV+GS+SM+R MD+PI+RD+RD	171	0.47	9.8	26
Taveras_2015	52	Primary care setting	USA	ME	184	0.46	9.8	25.7
Tucker_2013	26	Primary care setting	USA	ME	57	0.44	9.5	21
Tucker_2013	26	Primary care setting	USA	PSYE+MTV+PS+GS+PI+ RD+RD	68	0.55	10	21.6
Wake_2009	26	Primary care setting	Australia	PSYE+GS+RIF+CONTRA CTING+PI	139	0.43	7.4	20.2
Wake_2009	26	Primary care setting	Australia	ME	119	0.51	7.6	20.3
Wake_2013	52	Primary care setting, tertiary care settings	Australia	PSYE+PS+GS+FB+PI	62	0.5	7.2	22.3
Wake_2013	52	Primary care setting, tertiary care settings	Australia	ME	56	0.41	7.4	22.8
Waling_2012	52	Research centre	Sweden	PSYE+GS+TASK+SS+PI +G+RD	48	0.44	10.5	23.4
Waling_2012	52	Research centre	Sweden	ME	45	0.58	10.5	22.6
Warschburger_20	12	Medical	Germany	PSYE+GS+SM+FB+RIF+	249	0.53	11.3	

Study	Treatment duration (week)	Settings	Country/ region	Intervention components	Sample size	Proportion of girls	Age (year)	Baseline BMI (Kg/m²)
16		centre		SC+PI+MODELING+G				
Warschburger_2016	12	Medical centre	Germany	PSYE+GS+SM+RIF+SC+G	274	0.52	11.3	
Wright_2013	12	Primary care setting, remote	USA	STOPLIGHT+PSYE+PS+GS+SM+RMD+FB+RIF+CONTRACTING+SC+PI+MODELING+RULE+RD	24	0.38	10.9	26
Wright_2013	12	Primary care setting, remote	USA	ME	26	0.46	10.5	25.3
Boutelle_2014	16	Medical centre	USA	PSYE+TWC+MTV+SM+RIF+SC+RT+IT+PI+MODELING+G	22	0.46	10.5	28
Boutelle_2014	16	Medical centre	USA	ME	22	0.55	9.9	26.5
Christie_2017	26	Community setting	UK	ME	87	0.63	15.33	31.93
Christie_2017	26	Community setting	UK	PSYE+FBA+MTV+PI	87	0.62	15	32.07
Collin_2011	26	Research centre	Australia	PSYE+PS+GS+TASK+FB+RIF+PI+MODELING	42	0.62	8.2	24.6
Collin_2011	26	Research centre	Australia	GS+TASK+FB+PI+G	63	0.6	8.3	25.2
Collin_2011	26	Research centre	Australia	PSYE+PS+GS+TASK+FB+RIF+PI+MODELING+G	60	0.55	8.1	24.4
Hinton_2018	26	Medical centre, at home	UK	DM+FB	10	0.6	13	
Hinton_2018	26	Medical centre, at home	UK	ME	9	0.67	13	
Trost_2014	16	At school	USA	PSYE+GS+SM+FB+RIF+SC+SG+PI+MODELING+G	34	0.56	10.1	28.4
Trost_2014	16	At school	USA	PSYE+GS+SM+FB+RIF+SC+PI+MODELING+G	41	0.54	9.9	27.3
Ahmad_2018	16	Research centre, remote	Malaysia	PSYE+SCI+PS+GS+SM+FB+SC+PI	67	0.6	9.6	25.2
Ahmad_2018	16		Malaysia	ME	67	0.57	9.6	25.7

Study	Treatment duration (week)	Settings	Country/ region	Intervention components	Sample size	Proportion of girls	Age (year)	Baseline BMI (Kg/m²)
Alustiza_2021	104	Primary care setting	Spain	PSYE+SCI+MTV+SC+IT+SS+PI+G	47	0.6	13	26.4
Alustiza_2021	104	Primary care setting	Spain	ME	45	0.53	13.1	26.6
Anderson_2018	52	Medical centre	New Zealand	PSYE+PI	97	0.6	10.5	
Anderson_2018	52	Medical centre	New Zealand	PSYE+SCI+PI+G	100	0.5	10.8	
Arauz_2013	26	Primary care setting	USA	ME	12	0.58	10.4	
Arauz_2013	26	Primary care setting	USA	PSYE+FBA+PS+GS+TASK+SG+PI+STRESS	14	0.64	10.2	
Arlinghaus_2019	52	At school	USA	ME	63	0.59	12.05	27.43
Arlinghaus_2019	52	At school	USA	STOPLIGHT+PSYE+GS+TASK+SM+RIF+SC+PI+MODELING+G	180	0.48	12.06	26.91
Arlinghaus_2021_1	12	At school	USA	PSYE+SCI+GS+TASK+SM+DM+FB+RIF+PI+G	58	0.58	13.64	
Arlinghaus_2021_1	12	At school	USA	ME	54	0.6	13.62	
Arlinghaus_2021_2	12	At school	USA	PSYE+SCI+GS+TASK+SM+DM+FB+RIF+PI+G	27	0.58	13.64	
Arlinghaus_2021_2	12	At school	USA	ME	32	0.6	13.62	
Bagherniya_2018	31	At school	Iran	PSYE+SCI+PS+GS+RMD+RIF+SS+PI+G+RD	87	1	13.53	29.2
Bagherniya_2018	31	At school	Iran	ME	85	1	13.35	27.2
Ball_2011	16	Medical centre	Canada	PSYE+SCI+GS+SM+DM+PI	15	0.73	16.2	36.6
Ball_2011	16	Medical centre	Canada	PSYE+SCI+MTV+GS+SM+DM+PI	17	0.59	14.6	34.8
Ball_2011	16	Medical centre	Canada	ME	14	0.5	14.8	37.1
Bean, M. K._2018	26	Medical centre	USA	PSYE+SCI+MTV+PI+G	58	0.76	13.6	36.8
Bean, M. K._2018	26	Medical centre	USA	PSYE+PI+G	41	0.71	14.1	36.6
Berkowitz, R. I._2013	52	Primary care setting	USA	PSYE+CR+PS+GS+SM+FB+RIF+SC+SS+PI+STRESS+G	81	0.77	14.6	36.7

Study	Treatment duration (week)	Settings	Country/ region	Intervention components	Sample size	Proportion of girls	Age (year)	Baseline BMI (Kg/m^2)
Berkowitz, R. I. _2013	52	Primary care setting	USA	PSYE+CR+PS+GS+SM+RIF+SC+PI+STRESS	88	0.77	14.6	36.7
Bocca, G._2014	16	Medical centre	the Netherlands	PSYE+FBA+MTV+GS+DM+FB+RIF+SC+PI+MODELING+G	40	0.7	4.6	21.2
Bocca, G._2014	16	Medical centre	the Netherlands	PSYE+DM+G	35	0.74	4.7	21
Chew, C. S. E._2021	26	Medical centre	Singapore	ME	30	0.4	12.6	30.11
Chew, C. S. E._2021	26	Medical centre	Singapore	PSYE+SCI+FBA+GS+RIF+PRP+SS+PI+STRESS+G	31	0.39	13	29.93
Cohen, T. R. _2023	26	Research centre	Canada	ME	30	0.5	11.1	27.8
Cohen, T. R. _2023	26	Research centre	Canada	STOPLIGHT+PSYE+MTV+PS+GS+CONTRACTING+SG+PI	30	0.57	11.1	27.7
Crespo, N. C._2018	52	Primary care setting	USA	STOPLIGHT+PSYE+PS+GS+RMD+SC+PI+MODELING+G	149	0.46	8.17	20.7
Crespo, N. C._2018	52	Primary care setting	USA	ME	148	0.53	8.03	20.5
de Niet, J._2012	52	Remote	the Netherlands	PSYE+SCI+GS+RIF+IT+SS+SG+PI+G	68	0.66	9.8	
de Niet, J._2012	52	Remote	the Netherlands	PSYE+SCI+MTV+GS+SM+RMD+FB+RIF+IT+SS+SG+PI+G+RD	73	0.62	10	
Epstein, L. H._2000	52	Research centre	USA	STOPLIGHT+PSYE+PS+TASK+SM+RIF+SC+PRP+PI+G	35	0.51	10.49	
Epstein, L. H._2000	52	Research centre	USA	STOPLIGHT+PSYE+TASK+SM+RIF+SC+PRP+PI+G	17	0.53	10	
Faith, M. S._2001	12	At home	USA	RIF	6	0.33	10.2	30.2
Faith, M. S._2001	12	At home	USA	ME	4	0.25	10	26
Ford, A. L._2009	52	Medical centre	UK	ME	52	0.56	12.5	33.1
Ford, A. L._2009	52	Medical centre	UK	PSYE+DM+FB+PI+RD	54	0.56	12.7	34.4

Study	Treatment duration (week)	Settings	Country/ region	Intervention components	Sample size	Proportion of girls	Age (year)	Baseline BMI (Kg/m^2)
Fullerton, G._2007	26	At school	USA	PSYE+GS+SM+SC+PI+G	40		12.1	
Fullerton, G._2007	26	At school	USA	ME	40		12.1	
Gerards, S. M. P. L._2015	16	Primary care setting, remote	the Netherlands	PSYE+SCI+PS+GS+FB+RIF+PRP+PI+MODELING+RULE	44	0.57	7.14	20.37
Gerards, S. M. P. L._2015	16	Primary care setting, remote	the Netherlands	ME	42	0.55	7.29	20.67
Gillis, D._2007	26	Primary care setting	Jerusalem	SM+FB+RD	14		11.2	
Gillis, D._2007	26	Primary care setting	Jerusalem	ME	13		9.9	
Golan, M._2006	26	Medical centre	Israel	PSYE+CR+PS+TASK+SM+SC+IT+PI+MODELING	17	0.59	8.75	24.2
Golan, M._2006	26	Medical centre	Israel	PSYE+CR+PS+TASK+SM+SC+IT+PI+MODELING+G	20	0.5	8.7	24.3
Gourlan, M._2013	26	Medical centre	France	PSYE+GS+SM+FB	34			29.59
Gourlan, M._2013	26	Medical centre	France	PSYE+MTV+PS+GS+SM+FB	28			29.56
Grey, M._2009	16	At school	USA	PSYE+PS+GS+RIF+FB+PRP+SS+ROLE+STRESS+G+RD	112	0.63	12.8	30.5
Grey, M._2009	16	At school	USA	PSYE+PRP+G	86	0.42	12.6	30.3
Ham, O. K._2016	26	At school	South Korea	PSYE+SCI+MTV+PS+GS+TASK+RMD+FB+CONTRACTING+PI	48	0.44	10.77	24.35
Ham, O. K._2016	26	At school	South Korea	ME	27	0.44	10.26	24.22
Hofsteenge, G. H._2014	26	Medical centre	the Netherlands	PSYE+PS+GS+TASK+SM+DM+PRP+SS+PI+G	71	0.54	14.5	33.3
Hofsteenge, G. H._2014	26	Medical centre	the Netherlands	ME	51	0.59	14.4	33.6
Hughes, A. R._2008	26	Medical centre	UK	ME	65	0.55	8.5	
Hughes, A. R._2008	26	Medical centre	UK	STOPLIGHT+PSYE+MTV+PS+GS+SM+RIF+SS+	69	0.57	9.1	

Study	Treatment duration (week)	Settings	Country/ region	Intervention components	Sample size	Proportion of girls	Age (year)	Baseline BMI (Kg/m²)
				PI				
Hystad, H. T._2013	26	Medical centre	Norway	PSYE+SCI+MTV+GS+TASK+PRP+SS+PI+G	46	0.62	9.9	28.4
Hystad, H. T._2013	26	Medical centre	Norway	PSYE+PI+G	50	0.57	10.5	28.7
Janicke, D. M._2019	52	Community setting	USA	PSYE+PI+G	83	0.53	10.4	
Janicke, D. M._2019	52	Community setting	USA	STOPLIGHT+PSYE+MTV+PS+GS+SM+DM+RIF+FB+SC+SS+ROLE+PI+MODELING	78	0.49	10.3	
Janicke, D. M._2019	52	Community setting	USA	STOPLIGHT+PSYE+MTV+PS+GS+SM+DM+RIF+FB+SC+SS+ROLE+PI+MODELING+G	88	0.61	10.4	
Janicke, D. M._2008_1	16	Community setting	USA	STOPLIGHT+PSYE+SCI+FBA+MTV+PS+GS+SM+DM+RIF+CONTRACTING+SC+PRP+SS+PI+MODELING+G	24	0.63	11.4	
Janicke, D. M._2008_1	16	Community setting	USA	STOPLIGHT+PSYE+SCI+FBA+MTV+PS+GS+SM+DM+RIF+CONTRACTING+SC+PRP+SS+PI+MODELING	26	0.46	11	
Janicke, D. M._2008_1	16	Community setting	USA	ME	21	0.76	11	
Jelalian_2006	16	Medical centre	USA	PSYE+MTV+GS+SM+RIF+CONTRACTING+SC+PI+G	39	0.71	14.7	32.79
Jelalian_2006	16	Medical centre	USA	PSYE+SCI+MTV+GS+SM+RIF+CONTRACTING+SC+SS+SG+PI+G	37	0.71	14.31	32.15
Jiang_2005	104	At school, at home	China	ME	35	0.6	13.2	26.1
Jiang_2005	104	At school, at home	China	STOPLIGHT+PSYE+GS+SM+FB+RIF+PI	33	0.61	13.3	26.6
Johnston_2013	26	At school	USA	PSYE+GS+SM+RIF+SC+SG+PI+MODELING+G	46	0.52	12.2	27.7
Johnston_2013	26	At school	USA	ME	25	0.6	12.2	25.6

Study	Treatment duration (week)	Settings	Country/ region	Intervention components	Sample size	Proportion of girls	Age (year)	Baseline BMI (Kg/m^2)
Johnston_2010	26	At school	USA	PSYE+GS+SM+RIF+SC+SG+PI+MODELING+G	40	0.48	12.3	25.2
Johnston_2010	26	At school	USA	ME	20	0.4	12.5	26.7
Kalarchian_2009	26	Medical centre	USA	STOPLIGHT+PSYE+SCI+FBA+GS+SM+FB+RIF+SC+SS+PI+G	97	0.56	10.07	31.71
Kalarchian_2009	26	Medical centre	USA	ME	95	0.58	10.3	32.54
Kalavainen_2007	52	Medical centre	Finland	ME	35	0.66	8	22.9
Kalavainen_2007	52	Medical centre	Finland	PSYE+FBA+GS+TASK+PRP+SS+SG+PI+STRESS+MODELING+G	35	0.54	8.1	23.4
Kitzman-Ulrich_2009	16	Medical centre	USA	PSYE+SCI+MTV+PS+GS+TASK+SM+FB+RIF+PRP+SS+PI+STRESS+G	15			
Kitzman-Ulrich_2009	16	Medical centre	USA	PSYE+SCI+MTV+PS+GS+TASK+SM+RIF+PRP+SS+SG+PI+STRESS+G	16			
Kitzman-Ulrich_2009	16	Medical centre	USA	ME	11			
Kulendran_2016	12	Remote	UK	PSYE+PS+RMD+FB+RD	13	0.62	13.7	32.2
Kulendran_2016	12	Remote	UK	PSYE+PS+TASK+RMD+FB+RD	14	0.71	13.8	31.3
Love-Osborne_2014_1	26	At school	USA	PSYE+MTV+GS+SM+RMD+FB+RD	20	0.58	15.7	31.9
Love-Osborne_2014_1	26	At school	USA	ME	23	0.46	16	31.6
Love-Osborne_2014_2	26	At school	USA	PSYE+MTV+GS+SM+RMD+FB+RD	40	0.58	15.7	31.9
Love-Osborne_2014_2	26	At school	USA	ME	35	0.46	16	31.6
Love-Osborne_2014_3	26	At school	USA	PSYE+MTV+GS+SM+RMD+FB+RD	17	0.58	15.7	31.9
Love-Osborne_2014_3	26	At school	USA	ME	14	0.46	16	31.6
Luque_2024	52	Primary care setting	Spain	ME	95	0.38	10.58	25.7
Luque_2024	52	Primary care setting	Spain	PSYE+MTV+GS+TASK+DM+FB+SC+PI	106	0.51	10.83	25.75

Study	Treatment duration (week)	Settings	Country/ region	Intervention components	Sample size	Proportion of girls	Age (year)	Baseline BMI (Kg/m²)
MacDonell_2012	12	Medical centre	Michigan	PSYE+MTV+PS+PI	22	0.82	15.05	37.6
MacDonell_2012	12	Medical centre	Michigan	PSYE+PS+PI	22	0.77	15.18	34.92
Markert_2014	52	Remote	Germany	PSYE+FBA+PS+GS+PI+STRESS+RD	145	0.51	9.7	24.1
Markert_2014	52	Remote	Germany	ME	144	0.5	9.8	24.2
McCallum_2007	39	Primary care setting	Australia	PSYE+PS+GS+PI	82	0.49	7.5	20.5
McCallum_2007	39	Primary care setting	Australia	ME	81	0.54	7.4	20
Moore_2019	156	At home	USA	PSYE+SCI+MTV+PS+GS+SM+RIF+SS+STRESS+MODELING+G+RD	118	0.56	11.6	27.3
Moore_2019	156	At home	USA	PSYE+GS+SM+SC+PI+STRESS+G+RD	123	0.59	11.5	27.4
Moore_2019	156	At home	Cleveland	ME	119	0.58	11.6	26.8
Nguyen_2012	104	Primary care setting, tertiary care settings	Australia	PSYE+SCI+MTV+GS+SG+PI+STRESS+MODELING+G	78	0.54	14.2	30.8
Nguyen_2012	104	Primary care setting, tertiary care settings	Australia	PSYE+SCI+MTV+PS+GS+RMD+RIF+SG+PI+STRESS+MODELING+G+RD	73	0.49	14	30.8
Njardvik_2018	18	Medical centre	Iceland	STOPLIGHT+PSYE+CR+GS+SM+RIF+SC+SS+PI+G	43	0.49	10.9	
Njardvik_2018	18	Medical centre	Iceland	STOPLIGHT+PSYE+CR+TWC+FBA+GS+SM+RIF+SC+SS+PI+G	41	0.41	11.1	
Pakpour_2015	52	Medical centre	Iran	PSYE+MTV+G	119	0.54	15.59	33.07
Pakpour_2015	52	Medical centre	Iran	PSYE+MTV+PI+G	119	0.36	15.57	33.09
Pakpour_2015	52	Medical centre	Iran	ME	119	0.45	15.78	32.92
Patsopoulou_2017	12	Community setting	Greece	ME	61	0.53	14.04	33.4
Patsopoulou_201	12	Community	Greece	TASK+SG+G	60	0.48	14.04	32.6

Study	Treatment duration (week)	Settings	Country/ region	Intervention components	Sample size	Proportion of girls	Age (year)	Baseline BMI (Kg/m^2)
7		setting						
Patsopoulou_2017	12	Community setting	Greece	PSYE+MTV+TASK+SC+SG+PI+G	60	0.53	14.01	32.3
Resnicow_2005	26	Church	USA	PSYE+G	70	1	13.6	33.2
Resnicow_2005	26	Church	USA	PSYE+MTV+RMD+PI+G+RD	53	1	13.6	32
Rhee_2022	26	Primary care setting, tertiary care settings	USA	PSYE+SCI+FBA+MTV+PS+RIF+SC+PRP+SS+PI+G	79	0.52	9.6	26.9
Rhee_2022	26	Primary care setting, tertiary care settings	USA	PSYE+SCI+FBA+MTV+PS+GS+SM+FB+RIF+CONTRACTING+SC+PRP+SS+PI+MODELING	80	0.46	8.27	26.67
Robinson_2021	156	Primary care setting, community setting, at home	USA	PSYE+CR+MTV+PS+TASK+FB+SC+PI+G	120	0.58	9.5	25.26
Robinson_2021	156	Primary care setting, community setting, at home	USA	ME	121	0.54	9.5	24.86
Ruotsalainen_2015	12	Remote	Finland	PSYE+MTV+PS+DM+RIF+SS+PI+G+RD	15	0.67	14.8	29.8
Ruotsalainen_2015	12	Remote	Finland	PSYE+MTV+PS+SS+PI+G+RD	16	0.69	14.8	27.5
Ruotsalainen_2015	12	Remote	Finland	ME	15	0.73	14.7	27
Saelens_2013	20	Research centre	USA	STOPLIGHT+PSYE+PS+GS+SM+FB+RIF+SC+PI+G	37	0.68	9.8	27
Saelens_2013	20	Research centre	USA	STOPLIGHT+PSYE+MTV+GS+SM+FB+RIF+SC+PI+G	35	0.66	9.7	25.9
Saelens_2002	30	Primary care setting	USA	STOPLIGHT+PSYE+PS+GS+SM+FB+RIF+SC+PRP+PI+RD	23			31
Saelens_2002	30	Primary care	USA	ME	21			30.7

Study	Treatment duration (week)	Settings	Country/ region	Intervention components	Sample size	Proportion of girls	Age (year)	Baseline BMI (Kg/m^2)
		setting						
Sauder_2018	34	Research centre	USA	STOPLIGHT+RMD+FB+ SG+PI+G	29	0.55	9.3	26.4
Sauder_2018	34	Research centre	USA	ME	33	0.42	9.1	26.1
Savoye_2011	52	Medical centre	USA	PSYE+GS+SM+RIF+SC+ PRP+SS+PI+MODELING +G	105	0.55	12	35.7
Savoye_2011	52	Medical centre	USA	ME	69	0.68	12.5	36.2
Serra Paya_2015	34	Primary care setting	Spain	STOPLIGHT+PSYE+MT V+PS+GS+SM+FB+PRP+ SS+SG+PI+G	54	0.5	10.1	25.22
Serra Paya_2015	34	Primary care setting	Spain	ME	59	0.44	9.13	24.65
Small-2014	16	Primary care setting	USA	PSYE+MTV+PS+GS+FB+ PI	33	0.52	5.73	21.93
Small-2014	16	Primary care setting	USA	ME	27	0.7	5.41	20.36
Smith-2021	26	Medical centre, at home, community setting	USA	PSYE+MTV+PS+GS+FB+ PI+RULE+RD	141	0.48	9.5	25.77
Smith-2021	26	Medical centre, at home, community setting	USA	ME	99	0.51	9.5	25.31
Spence-2023	16	Medical centre	Canada	PSYE+CR+SCI+FBA+MT V+PS+GS+SM+SC+SS+P I+RULE	27	0.44	9.6	
Spence-2023	16	Medical centre	Canada	PSYE+SCI+GS+SM+SC+ SS+PI+RULE	25	0.6	9.9	
Stasinaki-2021	52	Medical centre	Switzerland	PSYE+TASK+SM+FB+RI F+RT+RD	18	0.39	13.38	28.98
Stasinaki-2021	52	Medical centre	Switzerland	PSYE+MTV+GS+TASK+ SM+CONTRACTING+SC	13	0.46	13.78	28.9
Taveras_2017	52	Primary care setting	USA	ME	361	0.52	8	22.8

Study	Treatment duration (week)	Settings	Country/ region	Intervention components	Sample size	Proportion of girls	Age (year)	Baseline BMI (Kg/m^2)
Taveras_2017	52	Primary care setting	USA	PSYE+MTV+PS+GS+FB+PI+RD	360	0.5	8.1	23
Taylor-2015	52	Research centre	New Zealand	ME	102	0.55	6.4	19
Taylor-2015	52	Medical centre	New Zealand	PSYE+PS+GS+FB+RIF+PI+RULE+RD	104	0.56	6.5	19.8
Tsai-2024	52	At school, at home	China	PSYE+MTV+PS+GS+SM+RMD+FB+PI	82	0.49	7.56	20.75
Tsai-2024	52	At school, at home	China	ME	82	0.46	7.57	20.86
Tsiros-2008	20	Medical centre	Australia	PSYE+CR+SCI+MTV+PS+GS+SM+DM+SC+PRP+PI+G+RD	25	0.64	14.6	30.6
Tsiros-2008	20	Medical centre	Australia	ME	22	0.68	14.6	31.1
Varagiannis-2021	26	Research centre	Greece	PSYE+SCI+PS+GS+DM+SC+ROLE+PI+G	36	0.58	10.2	26.2
Varagiannis-2021	26	Remote	Greece	PSYE+SCI+PS+GS+DM+SC+ROLE+PI	30	0.53	10	26
Varagiannis-2021	26	Remote	Greece	PSYE+SCI+PS+GS+DM+SC+ROLE+PI+RD	25	0.6	10.62	26
Vermeiren-2021	26	Remote	Belgium	PSYE+IT+PI+G+RD	90	0.59	13.2	
Vermeiren-2021	26	Remote	Belgium	PSYE+PI+G	86	0.62	13	
Vidmar-2021	12	Medical centre, at home	USA	PSYE+TASK+SM+RD	19	0.68	16.16	
Vidmar-2021	12	Medical centre, at home	USA	PSYE+TASK+SM+DM+FB+RD	16	0.69	16.8	
Vidmar-2021	12	Medical centre, at home	USA	PSYE+SM+RD	15	0.8	16.38	
Vidmar-2023	24	Remote	USA	STOPLIGHT+PSYE+TWC+FBA+MTV+GS+TASK+SM+RIF+SC+STRESS+RD	53	0.59	15.75	
Vidmar-2023	24	Remote	USA	STOPLIGHT+PSYE+TWC+FBA+MTV+PS+GS+TASK+SM+RMD+FB+RIF+SC+STRESS+RD	54	0.64	15.25	

Study	Treatment duration (week)	Settings	Country/ region	Intervention components	Sample size	Proportion of girls	Age (year)	Baseline BMI (Kg/m^2)
Vidmar-2023	24	Remote	USA	PSYE+TWC+GS+SM+PRP+PI+RULE	54	0.72	15.75	
Vos-2012	12	Medical centre	the Netherlands	STOPLIGHT+PSYE+SCI+MTV+PS+GS+TASK+FB+RIF+SC+IT+PRP+SS+ROLE+PI+MODELING+RULE+G	40	0.55	13.3	
Vos-2012	12	Medical centre	the Netherlands	ME	39	0.51	13.1	
Wafa-2011	26	Medical centre	Malaysia	STOPLIGHT+PSYE+MTV+PS+GS+SM+RIF+CONTRACTING+PI+STRESS+MODELING	52	0.46	9.7	27.6
Wafa-2011	26	Medical centre	Malaysia	ME	55	0.53	9.9	28
Walpole-2013	26	Medical centre	Canada	PSYE+MTV	20	0.7	14.1	30.3
Walpole-2013	26	Medical centre	Canada	ME	20	0.45	13.7	29.7
Weigensberg-2014	12	Research centre	USA	PSYE+CR+RT+G	18	0.53	15.5	36.2
Weigensberg-2014	12	Research centre	USA	PSYE+SCI+FBA+G	15	0.5	16.1	35.1
Williamson-2005	26	Remote	USA	PSYE+PS+GS+SM+FB+CONTRACTING+G+RD	28		13.14	35.31
Williamson-2005	26	Remote	USA	PSYE	29		13.23	37.34
Wylie-Rosett-2018	52	Primary care setting	USA	ME	182	0.48	9.3	
Wylie-Rosett-2018	52	Primary care setting	USA	PSYE+SCI+MTV+PS+GS+SM+FB+SG+PI+STRESS+MODELING+G	178	0.55	9.3	
Yackobovitch-Gavan-2018_1	12	Medical centre	Israel	PSYE+PRP+PI	89			
Yackobovitch-Gavan-2018_1	12	Medical centre	Israel	PSYE+PRP+PI+G	84			
Yackobovitch-Gavan-2018_1	12	Medical centre	Israel	ME	74			
Zoellner-2022	26	Community setting	USA	PSYE+PS+GS+FB+SS+PI+G+RD	70	0.66	10.1	
Zoellner-2022	26	Community	USA	PSYE+GS+SC+PI+MODE	69	0.49	10	

Study	Treatment duration (week)	Settings	Country/ region	Intervention components	Sample size	Proportion of girls	Age (year)	Baseline BMI (Kg/m^2)
		setting		LING				
Anna Ek_2019	52	Primary care setting, tertiary care settings	Sweden	PSYE+TWC+FBA+PS+RIF+SC+PRP+SS+PI+STRESS+RULE	44	0.43	5.2	21.4
Anna Ek_2019	52	Primary care setting, tertiary care settings	Sweden	PSYE+TWC+FBA+RIF+SC+PRP+SS+PI+STRESS+RULE	43	0.53	5.2	21.9
Anna Ek_2019	52	Primary care setting, tertiary care settings	Sweden	ME	87	0.64	5.3	21.3
Anderson_2017	52	Medical centre, at home, community setting	New Zealand	ME	69	0.58	10.3	28.9
Anderson_2017	52	Medical centre, at home, community setting	New Zealand	PSYE+SCI+CONTRACTING+SS+PI+G	69	0.49	10.7	29.6
Taveras_2011	52	Primary care setting	USA	PSYE+SCI+MTV+GS+RIF+PI+RD	253	0.48	4.8	19.2
Taveras_2011	52	Primary care setting	USA	ME	192	0.49	5.2	19.1

Notes: ME, minimal education; PSYE, psychoeducation; CR, cognitive restructuring; TWC, third-wave components; SCI, self-concept improvement; FBA, functional behavioural analysis; MTV, motivation; GS, goal-setting; TASK, task-setting; RULE, rule-setting; SM, self-monitoring; DM, device-monitoring; RMD, reminders; FB, feedback; PS, problem solving; PRP, preplanning; RIF, reinforcement; CONTRACTING, contracting; MODELING, modelling; SC, stimulus control; RT, relaxation training; IT, inhibition training; SS, social support; STRESS, stress management; STOPLIGHT, stoplight approach; ROLE, role playing; G, group; RD, remote; SG, serious games; PI, parental involvement

Appendix 3 Risk of bias assessments

Pairs of reviewers independently assessed the risk of bias of each study using the revised Cochrane risk-of-bias tool for randomized trials (RoB 2) (Sterne, 2019).

It involves judgement in five domains including randomisation process, deviations from intended interventions, missing outcome data, outcome measurement, and selection of reported results. Each domain contains several questions which leads to a final judgement: “low risk of bias”, “some concerns risk of bias” or “high risk of bias”. The domain-specific judgments informed an overall risk of bias assessment for each trial. A third reviewer checked the paired assessments and summarized the final results with any discrepancies were resolved by a senior reviewer.

Reference:

Sterne JAC, Savović J, Page MJ, et al. RoB 2: a revised tool for assessing risk of bias in randomised trials. BMJ. 2019;366:l4898. Published 2019 Aug 28.
doi:10.1136/bmj.l4898

Study	Domain1	Domain2	Domain3	Domain4	Domain5
**Absolute BMI z-score change from baseline**					
Sen_2018	Low	High	High	Low	Some concerns
Cohen_2016	Low	Low	Low	Low	Low
Willeboordse_2016	Low	Low	Some concerns	Low	Low
Bohlin_2017	Low	Some concerns	Low	Low	Low
Boutelle_2017	Low	Low	Low	Low	Low
Broccoli_2016	Low	Some concerns	Some concerns	Low	Low
Davis_2024	Low	Low	Low	Low	Low
Davis_2013	Low	Some concerns	Some concerns	Low	Low
Díaz_2010	Low	Low	Some concerns	Low	Some concerns
Doyle_2008	Low	Low	Low	Low	Low
Epstein_2004	Low	High	Some concerns	Low	Some concerns
Fedele_2018	Low	High	High	Low	Some concerns
Freira_2018	Some concerns	High	High	Low	Low
Garipağaoğlu_2009	Low	Some concerns	Low	Low	Some concerns
Golley_2007	Low	Low	Some concerns	Low	Low
Hidayanty_2016	Some concerns	Low	Some concerns	Low	Low
Jelalian_2011	Low	High	High	Low	Low
Looney_2014	Low	Low	Low	Low	Low
Maddison_2014	Low	Some concerns	Low	Low	Low
Magarey_2011	Some concerns	Some concerns	Some concerns	Low	Low
Marques_2023	Low	Some concerns	Some concerns	Low	Some concerns
Miri_2019	Low	Some concerns	Low	Low	Low
Norman_2016	Some concerns	Some concerns	Some concerns	Low	Low
O'Connor_2013	Low	Some concerns	Some concerns	Low	Some concerns
Patrick_2013	Some concerns	Low	Some concerns	Low	Low
Pbert_2016	Low	Low	Some concerns	Low	Low

Study	Domain1	Domain2	Domain3	Domain4	Domain5
Quattrin_2014	Low	Low	Low	Low	Low
Stark_2019	Some concerns	Some concerns	Low	Low	Low
Taveras_2015	Low	Low	Some concerns	Low	Low
Wake_2013	Low	Some concerns	Some concerns	Low	Low
Waling_2012	Low	Low	Some concerns	Low	Some concerns
Warschburger_2016	Low	Low	High	Low	Low
Wright_2013	Low	Low	Low	Low	Low
Boutelle_2014	Low	Some concerns	Low	Low	Some concerns
Christie_2017	Low	Low	Some concerns	Low	Low
Collin_2011	Low	Low	High	Low	Low
Hinton_2018	Some concerns	Low	Some concerns	Low	Low
Trost_2014	Low	Low	Some concerns	Low	Low
Ahmad_2018	Low	Low	Low	Low	Low
Alustiza_2021	Some concerns	Some concerns	Some concerns	Low	Low
Anderson_2018	Low	High	Some concerns	Low	Low
Arauz_2013	Low	Some concerns	High	Low	Low
Arlinghaus_2019	Low	Low	Some concerns	Low	Low
Arlinghaus_2021	Low	Some concerns	Low	Low	Low
Ball_2011	Some concerns	Some concerns	Low	Low	Low
Bean, M. K._2018	Low	Low	Low	Low	Low
Berkowitz, R. I. _2013	Low	Some concerns	Some concerns	Low	Low
Bocca, G._2014	Some concerns	Some concerns	Some concerns	Low	Low
Chew, C. S. E._2021	Low	High	High	Low	Low
Cohen, T. R. _2023	Low	Some concerns	Low	Low	Low
Crespo, N. C._2018	Low	Some concerns	Low	Low	Low
de Niet, J._2012	Low	Low	High	Low	Low
Epstein, L. H._2000	Some concerns	Some concerns	Some concerns	Low	Low
Ford, A. L._2009	Low	Low	Some concerns	Low	Low
Fullerton, G._2007	Some concerns	Some concerns	Some concerns	Low	Low
Gerards, S. M. P. L._2015	Low	Low	Some concerns	Low	Low
Gillis, D._2007	Low	High	High	Low	Some concerns
Golan, M._2006	Low	Low	Low	Low	Some concerns
Hofsteenge, G. H._2014	Low	Some concerns	Some concerns	Low	Low
Hughes, A. R._2008	Low	Low	Low	Low	Low
Hystad, H. T._2013	Some concerns	Some concerns	Some concerns	Low	Low
Janicke, D. M._2019	Low	Low	Some concerns	Low	Low
Janicke, D. M._2008	Low	High	High	Low	Low
Johnston_2010	Low	Low	Low	Low	Low
Kalavainen_2007	Low	Low	Low	Low	Some concerns
Kitzman-Ulrich_2009	Some concerns	High	High	Low	Some concerns
Love-Osborne_2014	Low	Some concerns	Some concerns	Low	Low

Study	Domain1	Domain2	Domain3	Domain4	Domain5
Luque_2024	Low	Low	Some concerns	Low	Low
Markert_2014	Low	High	Some concerns	Low	Low
McCallum_2007	Some concerns	Some concerns	Some concerns	Low	Low
Nguyen_2012	Some concerns	Some concerns	Some concerns	Low	Low
Njardvik_2018	Low	Low	Some concerns	Low	Some concerns
Pakpour_2015	Some concerns	Some concerns	Some concerns	Low	Low
Rhee_2022	Low	Low	Low	Low	Low
Saelens_2013	Some concerns	Some concerns	Low	Low	Low
Saelens_2002	Low	Low	Some concerns	Low	Some concerns
Sauder_2018	Low	Some concerns	Low	Low	Low
Savoye_2011	Low	Some concerns	Low	Low	Low
Serra Paya_2015	Low	Low	Low	Low	Low
Smith_2021	Low	Low	High	Low	Low
Spence_2023	Low	Low	Some concerns	Low	Low
Stasinaki_2021	Low	Low	Low	Low	Low
Taveras_2017	Low	Low	Some concerns	Low	Low
Taylor_2015	Some concerns	Some concerns	Some concerns	Low	Low
Tsai_2024	Low	Low	Some concerns	Low	Low
Varagiannis_2021	Low	High	Some concerns	Low	Some concerns
Vermeiren_2021	Low	Some concerns	High	Low	Low
Vidmar_2021	Some concerns	Low	Low	Low	Low
Vidmar_2023	Low	Low	High	Low	Low
Vos_2012	Low	Low	Some concerns	Low	Low
Wafa_2011	Low	Some concerns	Some concerns	Low	Low
Walpole_2013	Low	Low	Some concerns	Low	Low
Weigensberg_2014	Low	Some concerns	Some concerns	Low	Low
Wylie Rosett_2018	Low	Low	Some concerns	Low	Low
Yackobovitch Gavan_2018	Low	Some concerns	High	Low	Some concerns
Zoellner_2022	Low	Low	High	Low	Low
Anna Ek_2019	Low	Low	Some concerns	Low	Low
Anderson_2017	Low	High	High	Low	Low
Absolute BMI change from baseline					
Sánchez-López A M_2020	Low	Low	Low	Low	Low
Sen_2018	Low	High	High	Low	Some concerns
Abraham_2015	Low	Some concerns	Low	Low	Low
Broccoli_2016	Low	Some concerns	Some concerns	Low	Low
Díaz_2010	Low	Low	Some concerns	Low	Some concerns
Doyle_2008	Low	Low	Low	Low	Low
Garipağaoğlu_2009	Low	Some concerns	Low	Low	Some concerns
Hadley_2015	Some concerns	Some concerns	Low	Low	Low
Jelalian_2011	Low	High	High	Low	Low

Study	Domain1	Domain2	Domain3	Domain4	Domain5
Maddison_2014	Low	Some concerns	Low	Low	Low
Marques_2023	Low	Some concerns	Some concerns	Low	Some concerns
Nemet_2005	Low	Some concerns	Some concerns	Low	Low
Pbert_2016	Low	Low	Some concerns	Low	Low
Taveras_2015	Low	Low	Some concerns	Low	Low
Tucker_2013	Some concerns	Low	Some concerns	Low	Low
Wake_2009	Low	Some concerns	Some concerns	Low	Low
Wake_2013	Low	Some concerns	Some concerns	Low	Low
Waling_2012	Low	Low	Some concerns	Low	Some concerns
Wright_2013	Low	Low	Low	Low	Low
Boutelle_2014	Low	Some concerns	Low	Low	Some concerns
Christie_2017	Low	Low	Some concerns	Low	Low
Alustiza_2021	Some concerns	Some concerns	Some concerns	Low	Low
Arlinghaus_2019	Low	Low	Some concerns	Low	Low
Arlinghaus_2021	Low	Some concerns	Low	Low	Low
Bagherniya_2018	Some concerns	Some concerns	High	Low	Low
Ball_2011	Some concerns	Some concerns	Low	Low	Low
Berkowitz, R. I. _2013	Low	Some concerns	Some concerns	Low	Low
Bocca, G._2014	Some concerns	Some concerns	Some concerns	Low	Low
Cohen, T. R. _2023	Low	Some concerns	Low	Low	Low
Crespo, N. C._2018	Low	Some concerns	Low	Low	Low
Faith, M. S._2001	Low	Low	Some concerns	Low	Some concerns
Golan, M._2006	Low	Low	Low	Low	Some concerns
Gourlan, M._2013	Some concerns	Some concerns	Low	Low	Low
Grey, M._2009	Some concerns	Some concerns	Low	Low	Low
Ham, O. K._2016	Low	Some concerns	Low	Low	Low
Hofsteenge, G. H._2014	Low	Some concerns	Some concerns	Low	Low
Janicke, D. M._2019	Low	Low	Some concerns	Low	Low
Jelalian_2006	Low	Low	Low	Low	Some concerns
Jiang_2005	Low	Some concerns	Some concerns	Low	Low
Johnston_2013	Low	Low	Low	Low	Some concerns
Johnston_2010	Low	Low	Low	Low	Low
Kalarchian_2009	Low	Low	High	Low	Low
Kalavainen_2007	Low	Low	Low	Low	Some concerns
Kulendran_2016	Some concerns	Low	Some concerns	Low	Low
Luque_2024	Low	Low	Some concerns	Low	Low
MacDonell_2012	Some concerns	Some concerns	Some concerns	Low	Low
McCallum_2007	Some concerns	Some concerns	Some concerns	Low	Low
Moore_2019	Low	Some concerns	Low	Low	Low
Nguyen_2012	Some concerns	Some concerns	Some concerns	Low	Low
Pakpour_2015	Some concerns	Some concerns	Some concerns	Low	Low

Study	Domain1	Domain2	Domain3	Domain4	Domain5
Patsopoulou_2017	Low	Low	Low	Low	Low
Resnicow_2005	Low	Low	High	Low	Some concerns
Robinson_2021	Low	Low	Low	Low	Low
Ruotsalainen_2015	Low	Some concerns	Low	Low	Low
Saelens_2002	Low	Low	Some concerns	Low	Some concerns
Sauder_2018	Low	Some concerns	Low	Low	Low
Savoye_2011	Low	Some concerns	Low	Low	Low
Smith_2021	Low	Low	High	Low	Low
Taylor_2015	Some concerns	Some concerns	Some concerns	Low	Low
Tsai_2024	Low	Low	Some concerns	Low	Low
Tsiros_2008	Some concerns	Low	Some concerns	Low	Some concerns
Varagiannis_2021	Low	High	Some concerns	Low	Some concerns
Walpole_2013	Low	Low	Some concerns	Low	Low
Weigensberg_2014	Low	Some concerns	Some concerns	Low	Low
Williamson_2005	Low	Some concerns	Some concerns	Low	Some concerns
Anna Ek_2019	Low	Low	Some concerns	Low	Low
Taveras_2011	Low	Low	Low	Low	Some concerns
**Absolute weight change from baseline**					
Sánchez-López A M_2020	Low	Low	Low	Low	Low
Díaz_2010	Low	Low	Some concerns	Low	Some concerns
Doyle_2008	Low	Low	Low	Low	Low
Jelalian_2011	Low	High	High	Low	Low
Maddison_2014	Low	Some concerns	Low	Low	Low
Marques_2023	Low	Some concerns	Some concerns	Low	Some concerns
Nemet_2005	Low	Some concerns	Some concerns	Low	Low
Quattrin_2014	Low	Low	Low	Low	Low
Stark_2019	Some concerns	Some concerns	Low	Low	Low
Waling_2012	Low	Low	Some concerns	Low	Some concerns
Wright_2013	Low	Low	Low	Low	Low
Christie_2017	Low	Low	Some concerns	Low	Low
Arlinghaus_2019	Low	Low	Some concerns	Low	Low
Arlinghaus_2021	Low	Some concerns	Low	Low	Low
Ball_2011	Some concerns	Some concerns	Low	Low	Low
Berkowitz, R. I. _2013	Low	Some concerns	Some concerns	Low	Low
Bocca, G._2014	Some concerns	Some concerns	Some concerns	Low	Low
Chew, C. S. E._2021	Low	High	High	Low	Low
Cohen, T. R. _2023	Low	Some concerns	Low	Low	Low
Epstein, L. H._2000	Some concerns	Some concerns	Some concerns	Low	Low
Faith, M. S._2001	Low	Low	Some concerns	Low	Some concerns
Hofsteenge, G. H._2014	Low	Some concerns	Some concerns	Low	Low
Hughes, A. R._2008	Low	Low	Low	Low	Low

Study	Domain1	Domain2	Domain3	Domain4	Domain5
Jelalian_2006	Low	Low	Low	Low	Some concerns
Jiang_2005	Low	Some concerns	Some concerns	Low	Low
Johnston_2013	Low	Low	Low	Low	Some concerns
Johnston_2010	Low	Low	Low	Low	Low
Kalarchian_2009	Low	Low	High	Low	Low
Kalavainen_2007	Low	Low	Low	Low	Some concerns
Nguyen_2012	Some concerns	Some concerns	Some concerns	Low	Low
Patsopoulou_2017	Low	Low	Low	Low	Low
Resnicow_2005	Low	Low	High	Low	Some concerns
Ruotsalainen_2015	Low	Some concerns	Low	Low	Low
Saelens_2002	Low	Low	Some concerns	Low	Some concerns
Savoye_2011	Low	Some concerns	Low	Low	Low
Taylor_2015	Some concerns	Some concerns	Some concerns	Low	Low
Tsiros_2008	Some concerns	Low	Some concerns	Low	Some concerns
Varagiannis_2021	Low	High	Some concerns	Low	Some concerns
Wafa_2011	Low	Some concerns	Some concerns	Low	Low
Williamson_2005	Low	Some concerns	Some concerns	Low	Some concerns
**Absolute height change from baseline**					
Maddison_2014	Low	Some concerns	Low	Low	Low
Marques_2023	Low	Some concerns	Some concerns	Low	Some concerns
Quattrin_2014	Low	Low	Low	Low	Low
Stark_2019	Some concerns	Some concerns	Low	Low	Low
Waling_2012	Low	Low	Some concerns	Low	Some concerns
Wright_2013	Low	Low	Low	Low	Low
Arlinghaus_2019	Low	Low	Some concerns	Low	Low
Arlinghaus_2021	Low	Some concerns	Low	Low	Low
Ball_2011	Some concerns	Some concerns	Low	Low	Low
Berkowitz, R. I. _2013	Low	Some concerns	Some concerns	Low	Low
Cohen, T. R. _2023	Low	Some concerns	Low	Low	Low
Epstein, L. H._2000	Some concerns	Some concerns	Some concerns	Low	Low
Faith, M. S._2001	Low	Low	Some concerns	Low	Some concerns
Hofsteenge, G. H._2014	Low	Some concerns	Some concerns	Low	Low
Jiang_2005	Low	Some concerns	Some concerns	Low	Low
Johnston_2013	Low	Low	Low	Low	Some concerns
Johnston_2010	Low	Low	Low	Low	Low
Kalavainen_2007	Low	Low	Low	Low	Some concerns
Nguyen_2012	Some concerns	Some concerns	Some concerns	Low	Low
Patsopoulou_2017	Low	Low	Low	Low	Low
Resnicow_2005	Low	Low	High	Low	Some concerns
Ruotsalainen_2015	Low	Some concerns	Low	Low	Low
Saelens_2002	Low	Low	Some concerns	Low	Some concerns

Study	Domain1	Domain2	Domain3	Domain4	Domain5
Savoye_2011	Low	Some concerns	Low	Low	Low
Taylor_2015	Some concerns	Some concerns	Some concerns	Low	Low
Tsiros_2008	Some concerns	Low	Some concerns	Low	Some concerns
Varagiannis_2021	Low	High	Some concerns	Low	Some concerns
**Absolute body fat percentage change from baseline**					
Sánchez-López A M_2020	Low	Low	Low	Low	Low
Cohen_2016	Low	Low	Low	Low	Low
Abraham_2015	Low	Some concerns	Low	Low	Low
Díaz_2010	Low	Low	Some concerns	Low	Some concerns
Freira_2018	Some concerns	High	High	Low	Low
Maddison_2014	Low	Some concerns	Low	Low	Low
Miri_2019	Low	Some concerns	Low	Low	Low
Nemet_2005	Low	Some concerns	Some concerns	Low	Low
Patrick_2013	Some concerns	Low	Some concerns	Low	Low
Pbert_2016	Low	Low	Some concerns	Low	Low
Wake_2013	Low	Some concerns	Some concerns	Low	Low
Waling_2012	Low	Low	Some concerns	Low	Some concerns
Christie_2017	Low	Low	Some concerns	Low	Low
Ahmad_2018	Low	Low	Low	Low	Low
Bocca, G._2014	Some concerns	Some concerns	Some concerns	Low	Low
Chew, C. S. E._2021	Low	High	High	Low	Low
Cohen, T. R. _2023	Low	Some concerns	Low	Low	Low
Faith, M. S._2001	Low	Low	Some concerns	Low	Some concerns
Ford, A. L._2009	Low	Low	Some concerns	Low	Low
Grey, M._2009	Some concerns	Some concerns	Low	Low	Low
Hystad, H. T._2013	Some concerns	Some concerns	Some concerns	Low	Low
Johnston_2013	Low	Low	Low	Low	Some concerns
Johnston_2010	Low	Low	Low	Low	Low
Kalarchian_2009	Low	Low	High	Low	Low
Moore_2019	Low	Some concerns	Low	Low	Low
Pakpour_2015	Some concerns	Some concerns	Some concerns	Low	Low
Resnicow_2005	Low	Low	High	Low	Some concerns
Robinson_2021	Low	Low	Low	Low	Low
Savoye_2011	Low	Some concerns	Low	Low	Low
Smith_2021	Low	Low	High	Low	Low
Stasinaki_2021	Low	Low	Low	Low	Low
Taylor_2015	Some concerns	Some concerns	Some concerns	Low	Low
Tsiros_2008	Some concerns	Low	Some concerns	Low	Some concerns
Varagiannis_2021	Low	High	Some concerns	Low	Some concerns
Weigensberg_2014	Low	Some concerns	Some concerns	Low	Low
Williamson_2005	Low	Some concerns	Some concerns	Low	Some concerns

Study	Domain1	Domain2	Domain3	Domain4	Domain5
**Absolute waist circumference change from baseline**					
Cohen_2016	Low	Low	Low	Low	Low
Abraham_2015	Low	Some concerns	Low	Low	Low
Berry_2014	Low	Low	Low	Low	Low
Díaz_2010	Low	Low	Some concerns	Low	Some concerns
Freira_2018	Some concerns	High	High	Low	Low
Hidayanty_2016	Some concerns	Low	Some concerns	Low	Low
Jelalian_2011	Low	High	High	Low	Low
Miri_2019	Low	Some concerns	Low	Low	Low
Pbert_2016	Low	Low	Some concerns	Low	Low
Wake_2013	Low	Some concerns	Some concerns	Low	Low
Waling_2012	Low	Low	Some concerns	Low	Some concerns
Christie_2017	Low	Low	Some concerns	Low	Low
Alustiza_2021	Some concerns	Some concerns	Some concerns	Low	Low
Bagherniya_2018	Some concerns	Some concerns	High	Low	Low
Ball_2011	Some concerns	Some concerns	Low	Low	Low
Berkowitz, R. I. _2013	Low	Some concerns	Some concerns	Low	Low
Bocca, G._2014	Some concerns	Some concerns	Some concerns	Low	Low
Chew, C. S. E._2021	Low	High	High	Low	Low
Cohen, T. R. _2023	Low	Some concerns	Low	Low	Low
Gerards, S. M. P. L._2015	Low	Low	Some concerns	Low	Low
Grey, M._2009	Some concerns	Some concerns	Low	Low	Low
Hofsteenge, G. H._2014	Low	Some concerns	Some concerns	Low	Low
Hughes, A. R._2008	Low	Low	Low	Low	Low
Kalarchian_2009	Low	Low	High	Low	Low
Kalavainen_2007	Low	Low	Low	Low	Some concerns
Luque_2024	Low	Low	Some concerns	Low	Low
Moore_2019	Low	Some concerns	Low	Low	Low
Nguyen_2012	Some concerns	Some concerns	Some concerns	Low	Low
Pakpour_2015	Some concerns	Some concerns	Some concerns	Low	Low
Patsopoulou_2017	Low	Low	Low	Low	Low
Resnicow_2005	Low	Low	High	Low	Some concerns
Robinson_2021	Low	Low	Low	Low	Low
Sauder_2018	Low	Some concerns	Low	Low	Low
Serra Paya_2015	Low	Low	Low	Low	Low
Small_2014	Some concerns	Some concerns	Low	Low	Low
Stasinaki_2021	Low	Low	Low	Low	Low
Taylor_2015	Some concerns	Some concerns	Some concerns	Low	Low
Tsiros_2008	Some concerns	Low	Some concerns	Low	Some concerns
Varagiannis_2021	Low	High	Some concerns	Low	Some concerns
Walpole_2013	Low	Low	Some concerns	Low	Low

Study	Domain1	Domain2	Domain3	Domain4	Domain5
Weigensberg_2014	Low	Some concerns	Some concerns	Low	Low
Anna Ek_2019	Low	Low	Some concerns	Low	Low
Anderson_2017	Low	High	High	Low	Low
**Quality-of-life score change**					
Fedele_2018	Low	High	High	Some concerns	Some concerns
Freira_2018	Some concerns	High	High	Some concerns	Low
Miri_2019	Low	Some concerns	Low	Some concerns	Low
Patrick_2013	Some concerns	Low	Some concerns	Some concerns	Low
Wake_2009	Low	Some concerns	Some concerns	Some concerns	Low
Bocca, G._2014	Some concerns	Some concerns	Some concerns	Some concerns	Low
de Niet, J._2012	Low	Low	High	Some concerns	Low
Fullerton, G._2007	Some concerns	Some concerns	Some concerns	Some concerns	Low
Hofsteenge, G. H._2014	Low	Some concerns	Some concerns	Some concerns	Low
Janicke, D. M._2019	Low	Low	Some concerns	Some concerns	Low
Kalarchian_2009	Low	Low	High	Some concerns	Low
McCallum_2007	Some concerns	Some concerns	Some concerns	Some concerns	Low
Pakpour_2015	Some concerns	Some concerns	Some concerns	Some concerns	Low
Taveras_2017	Low	Low	Some concerns	Some concerns	Low
Vos_2012	Low	Low	Some concerns	Some concerns	Low
Wafa_2011	Low	Some concerns	Some concerns	Some concerns	Low
Zoellner_2022	Low	Low	High	Some concerns	Low
Anderson_2017	Low	High	High	Some concerns	Low
**Mental health score change**					
Abraham_2015	Low	Some concerns	Low	Some concerns	Low
Patrick_2013	Some concerns	Low	Some concerns	Some concerns	Low
Bocca, G._2014	Some concerns	Some concerns	Some concerns	Some concerns	Low
Grey, M._2009	Some concerns	Some concerns	Low	Some concerns	Low
Nguyen_2012	Some concerns	Some concerns	Some concerns	Some concerns	Low
Njardvik_2018	Low	Low	Some concerns	Some concerns	Some concerns
Robinson_2021	Low	Low	Low	Some concerns	Low
Weigensberg_2014	Low	Some concerns	Some concerns	Some concerns	Low

Appendix 4 Supplement to results

4.1 Network plots for the outcomes in conceptual level NMA

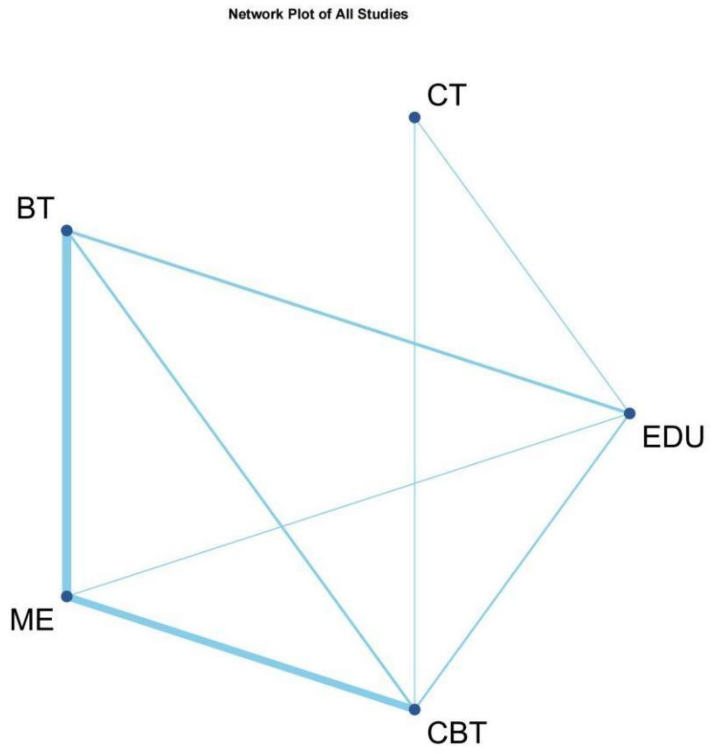

Notes: BT, behavioural therapy; CBT, cognitive behavioural therapy; CT, cognitive therapy; EDU, psychoeducation; ME, minimal education

4.1.1 BMI z-score

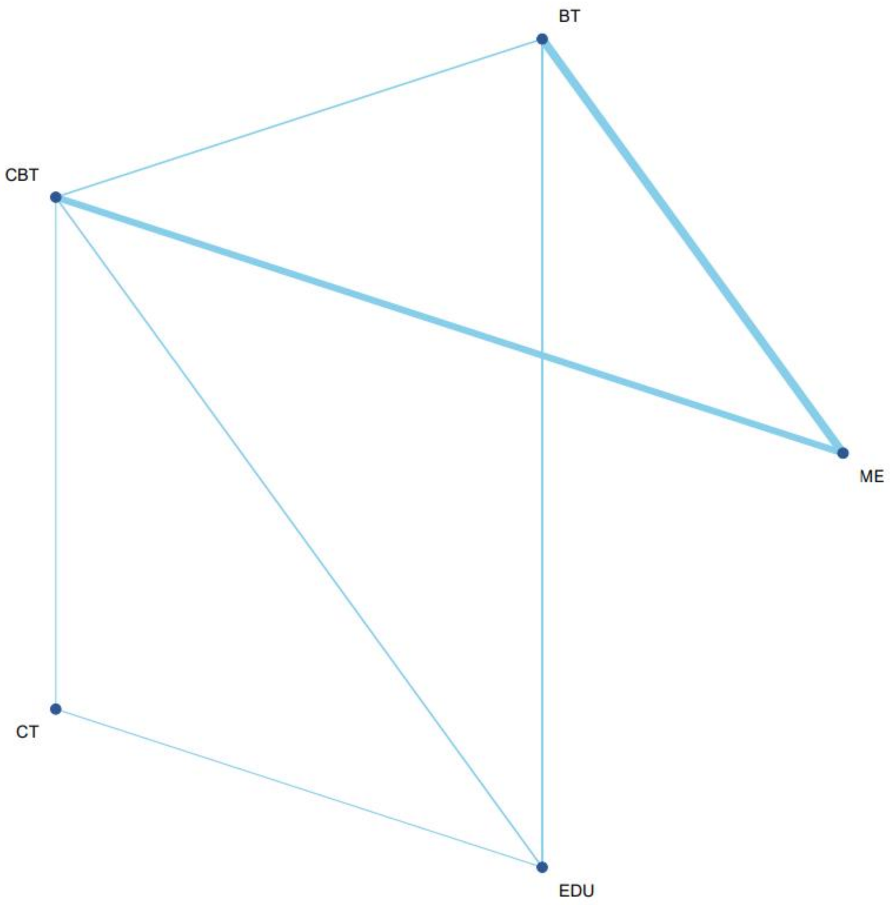

Notes: BT, behavioural therapy; CBT, cognitive behavioural therapy; CT, cognitive therapy; EDU, psychoeducation; ME, minimal education

4.1.2 Body fat

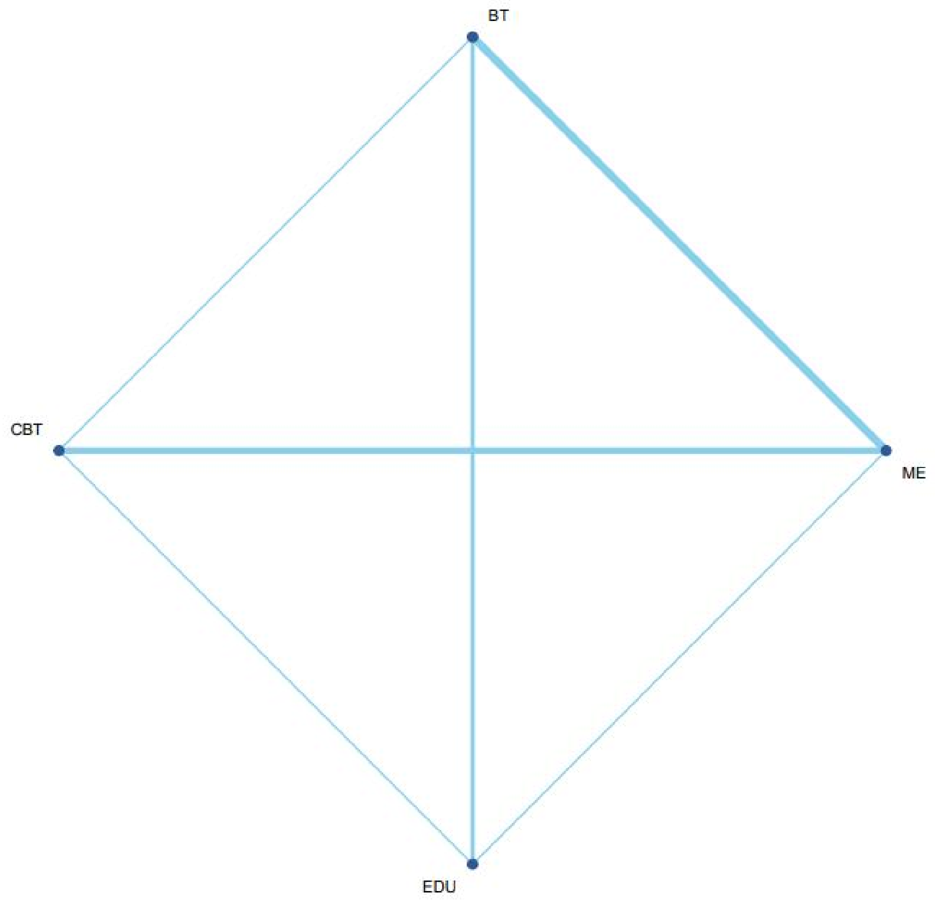

Notes: BT, behavioural therapy; CBT, cognitive behavioural therapy; CT, cognitive therapy; EDU, psychoeducation; ME, minimal education

4.1.3 Waist circumference

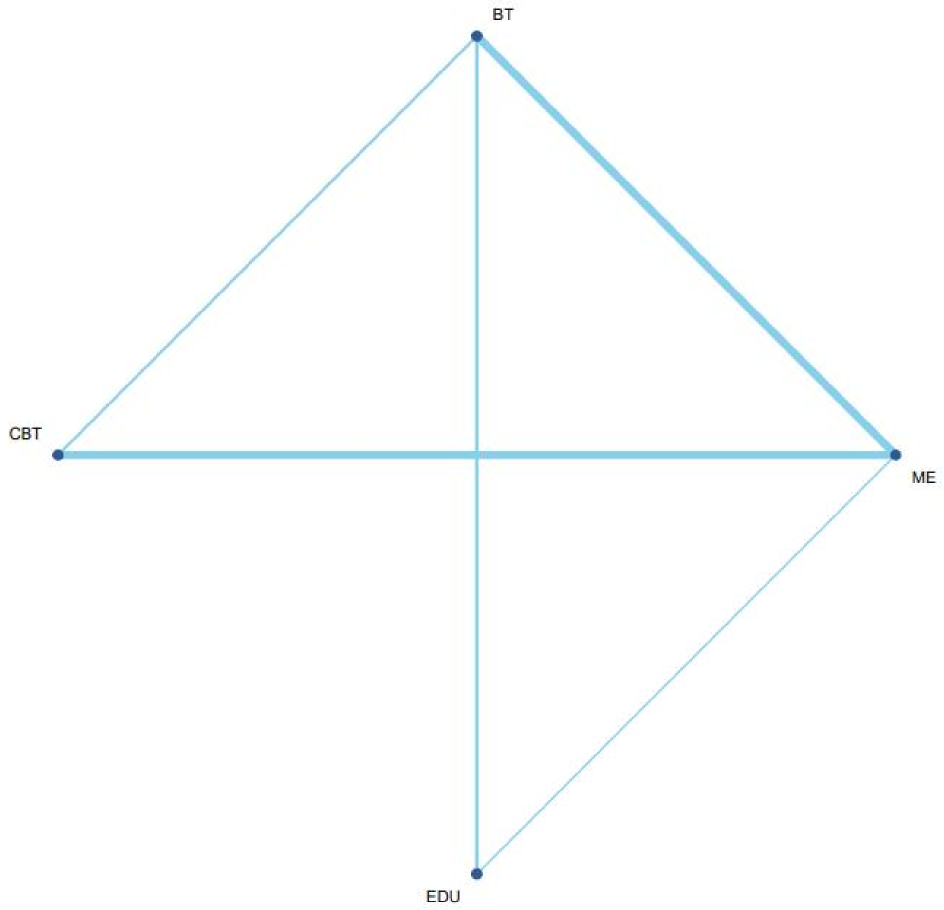

Notes: BT, behavioural therapy; CBT, cognitive behavioural therapy; CT, cognitive therapy; EDU, psychoeducation; ME, minimal education

4.1.4 Height

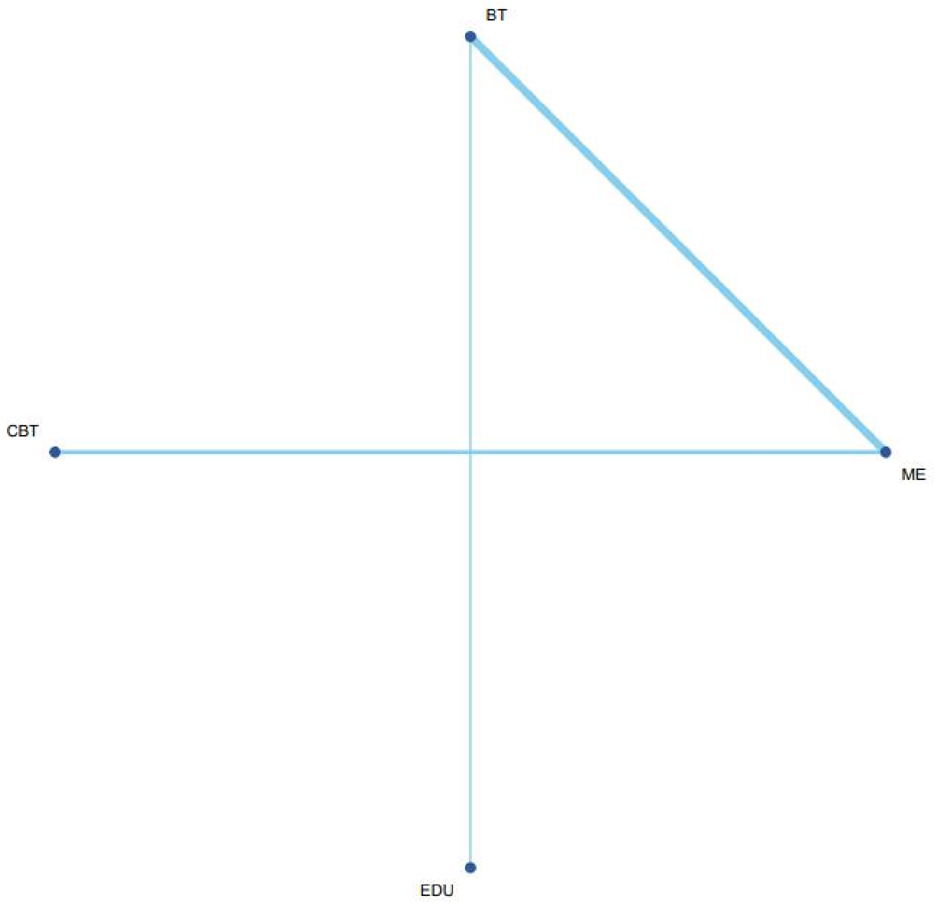

Notes: BT, behavioural therapy; CBT, cognitive behavioural therapy; CT, cognitive therapy; EDU, psychoeducation; ME, minimal education

4.1.5 Quality of life

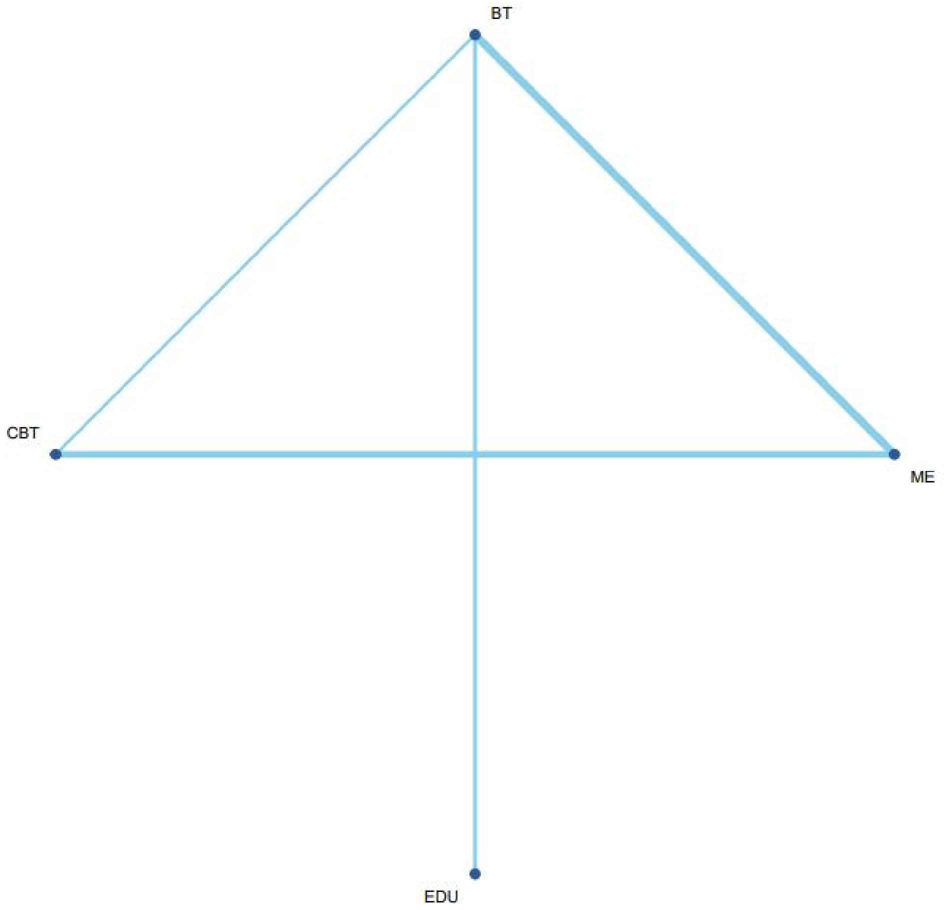

Notes: BT, behavioural therapy; CBT, cognitive behavioural therapy; CT, cognitive therapy; EDU, psychoeducation; ME, minimal education

4.1.6 Mental health

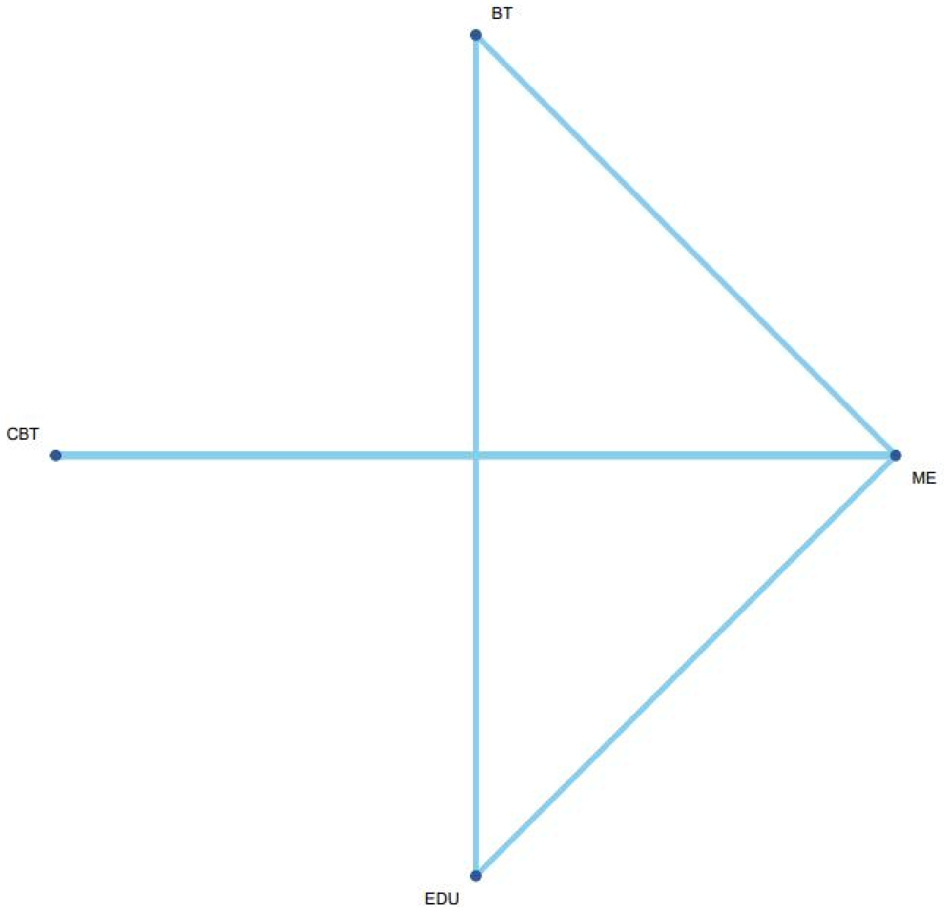

Notes: BT, behavioural therapy; CBT, cognitive behavioural therapy; CT, cognitive therapy; EDU, psychoeducation; ME, minimal education

4.2 Network plots for the outcomes in technical level CNMA

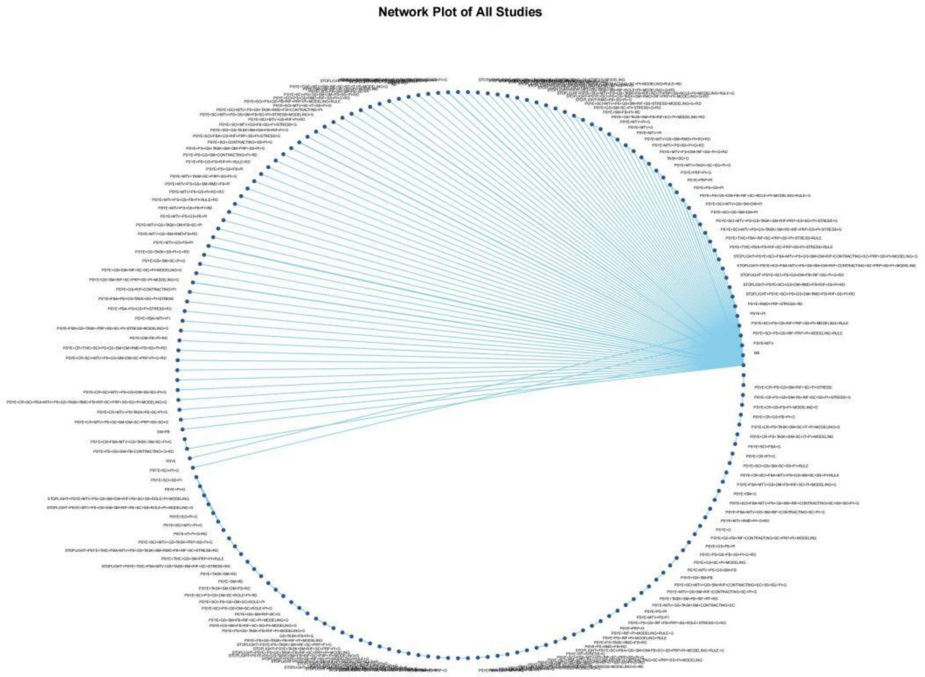

Notes: ME, minimal education; PSYE, psychoeducation; CR, cognitive restructuring; TWC, third-wave components; SCI, self-concept improvement; FBA, functional behavioural analysis; MTV, motivation; GS, goal-setting; TASK, task-setting; RULE, rule-setting; SM, self-monitoring; DM, device-monitoring; RMD, reminders; FB, feedback; PS, problem solving; PRP, preplanning; RIF, reinforcement; CONTRACTING, contracting; MODELING, modelling; SC, stimulus control; RT, relaxation training; IT, inhibition training; SS, social support; STRESS, stress management; STOPLIGHT, stoplight approach; ROLE, role playing; G, group; RD, remote; SG, serious games; PI, parental involvement

4.2.1 BMI z-score

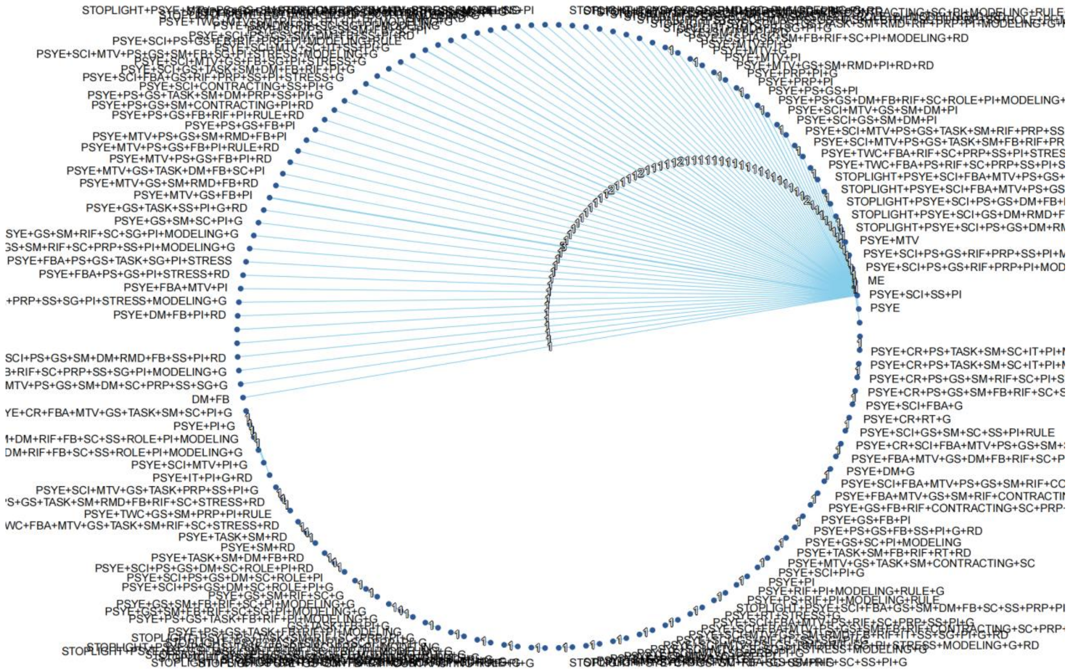

Notes: ME, minimal education; PSYE, psychoeducation; CR, cognitive restructuring; TWC, third-wave components; SCI, self-concept improvement; FBA, functional behavioural analysis; MTV, motivation; GS, goal-setting; TASK, task-setting; RULE, rule-setting; SM, self-monitoring; DM, device-monitoring; RMD, reminders; FB, feedback; PS, problem solving; PRP, preplanning; RIF, reinforcement; CONTRACTING, contracting; MODELING, modelling; SC, stimulus control; RT, relaxation training; IT, inhibition training; SS, social support; STRESS, stress management; STOPLIGHT, stoplight approach; ROLE, role playing; G, group; RD, remote; SG, serious games; PI, parental involvement

4.2.2 Body fat

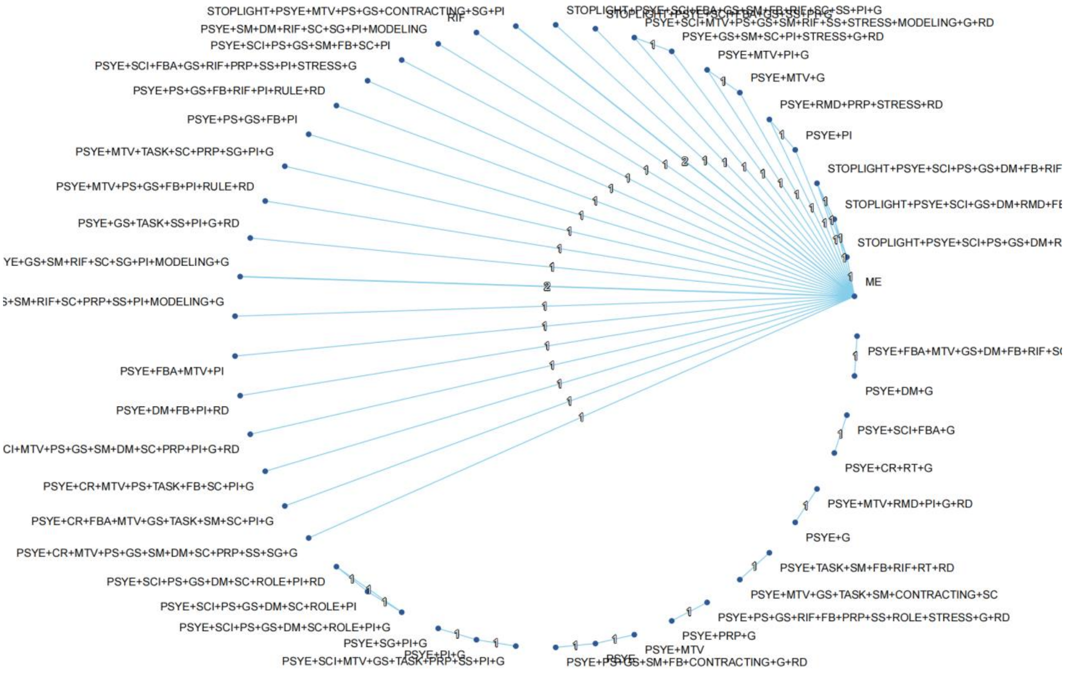

Notes: ME, minimal education; PSYE, psychoeducation; CR, cognitive restructuring; TWC, third-wave components; SCI, self-concept improvement; FBA, functional behavioural analysis; MTV, motivation; GS, goal-setting; TASK, task-setting; RULE, rule-setting; SM, self-monitoring; DM, device-monitoring; RMD, reminders; FB, feedback; PS, problem solving; PRP, preplanning; RIF, reinforcement; CONTRACTING, contracting; MODELING, modelling; SC, stimulus control; RT, relaxation training; IT, inhibition training; SS, social support; STRESS, stress management; STOPLIGHT, stoplight approach; ROLE, role playing; G, group; RD, remote; SG, serious games; PI, parental

4.2.3 Waist circumference

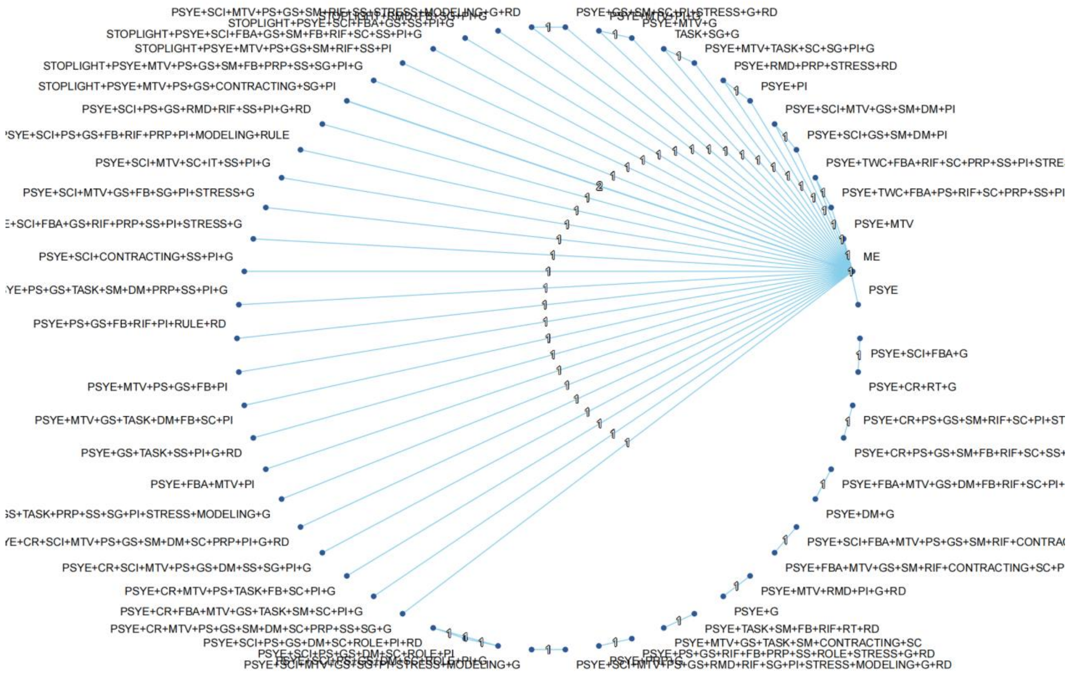

Notes: ME, minimal education; PSYE, psychoeducation; CR, cognitive restructuring; TWC, third-wave components; SCI, self-concept improvement; FBA, functional behavioural analysis; MTV, motivation; GS, goal-setting; TASK, task-setting; RULE, rule-setting; SM, self-monitoring; DM, device-monitoring; RMD, reminders; FB, feedback; PS, problem solving; PRP, preplanning; RIF, reinforcement; CONTRACTING, contracting; MODELING, modelling; SC, stimulus control; RT, relaxation training; IT, inhibition training; SS, social support; STRESS, stress management; STOPLIGHT, stoplight approach; ROLE, role playing; G, group; RD, remote; SG, serious games; PI, parental involvement

4.2.4 Height

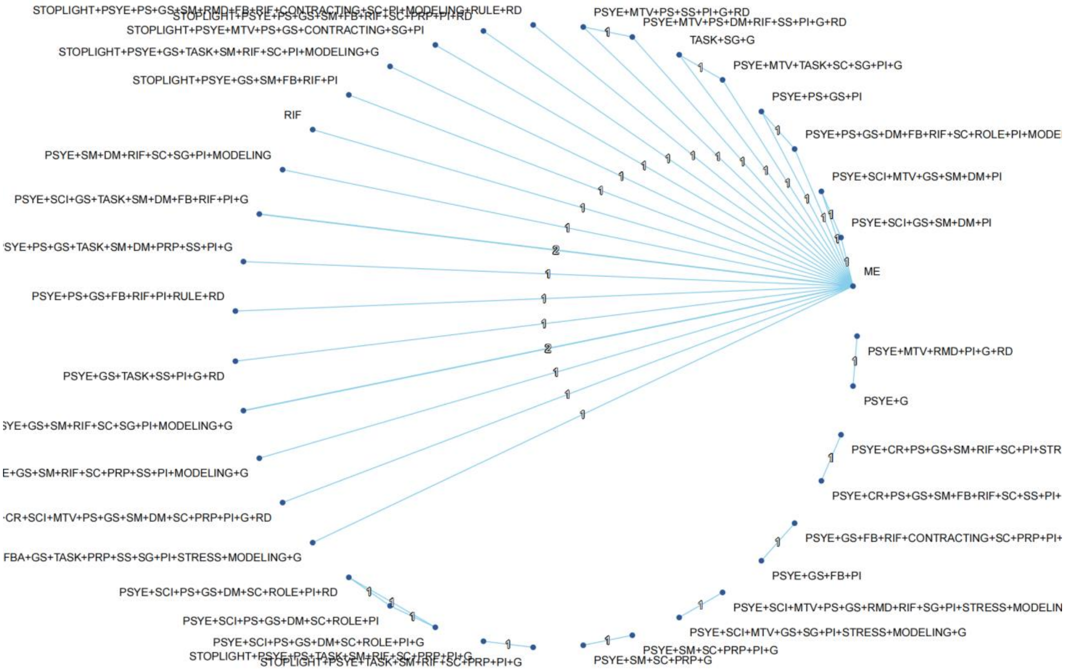

Notes: ME, minimal education; PSYE, psychoeducation; CR, cognitive restructuring; TWC, third-wave components; SCI, self-concept improvement; FBA, functional behavioural analysis; MTV, motivation; GS, goal-setting; TASK, task-setting; RULE, rule-setting; SM, self-monitoring; DM, device-monitoring; RMD, reminders; FB, feedback; PS, problem solving; PRP, preplanning; RIF, reinforcement; CONTRACTING, contracting; MODELING, modelling; SC, stimulus control; RT, relaxation training; IT, inhibition training; SS, social support; STRESS, stress management; STOPLIGHT, stoplight approach; ROLE, role playing; G, group; RD, remote; SG, serious games; PI, parental involvement

4.2.5 Quality of life

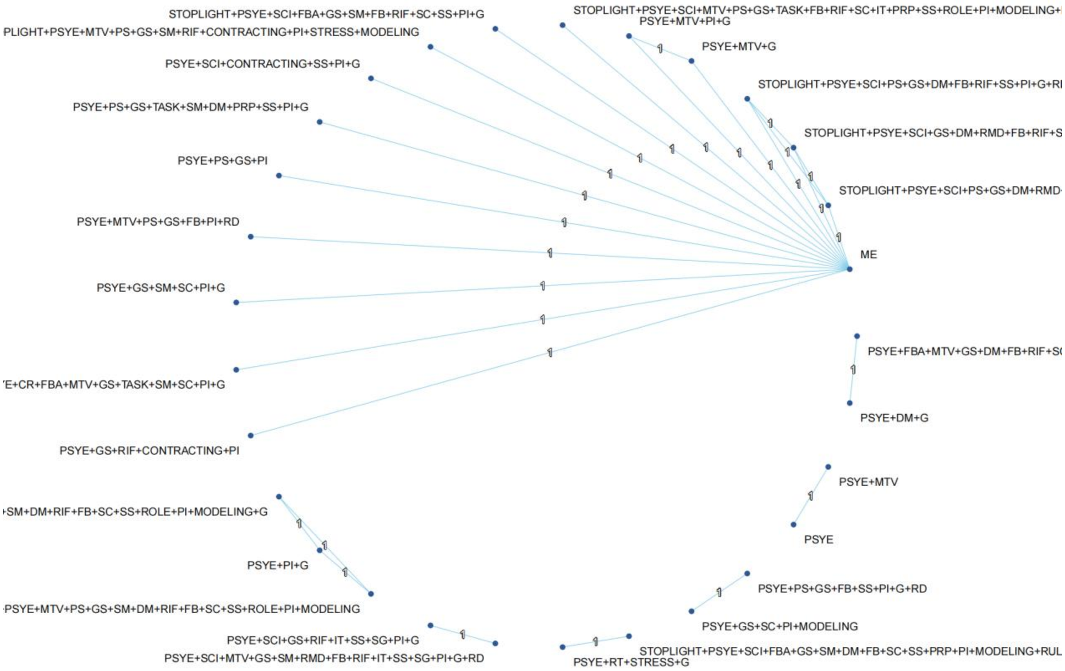

Notes: ME, minimal education; PSYE, psychoeducation; CR, cognitive restructuring; TWC, third-wave components; SCI, self-concept improvement; FBA, functional behavioural analysis; MTV, motivation; GS, goal-setting; TASK, task-setting; RULE, rule-setting; SM, self-monitoring; DM, device-monitoring; RMD, reminders; FB, feedback; PS, problem solving; PRP, preplanning; RIF, reinforcement; CONTRACTING, contracting; MODELING, modelling; SC, stimulus control; RT, relaxation training; IT, inhibition training; SS, social support; STRESS, stress management; STOPLIGHT, stoplight approach; ROLE, role playing; G, group; RD, remote; SG, serious games; PI, parental involvement

4.2.6 Mental health

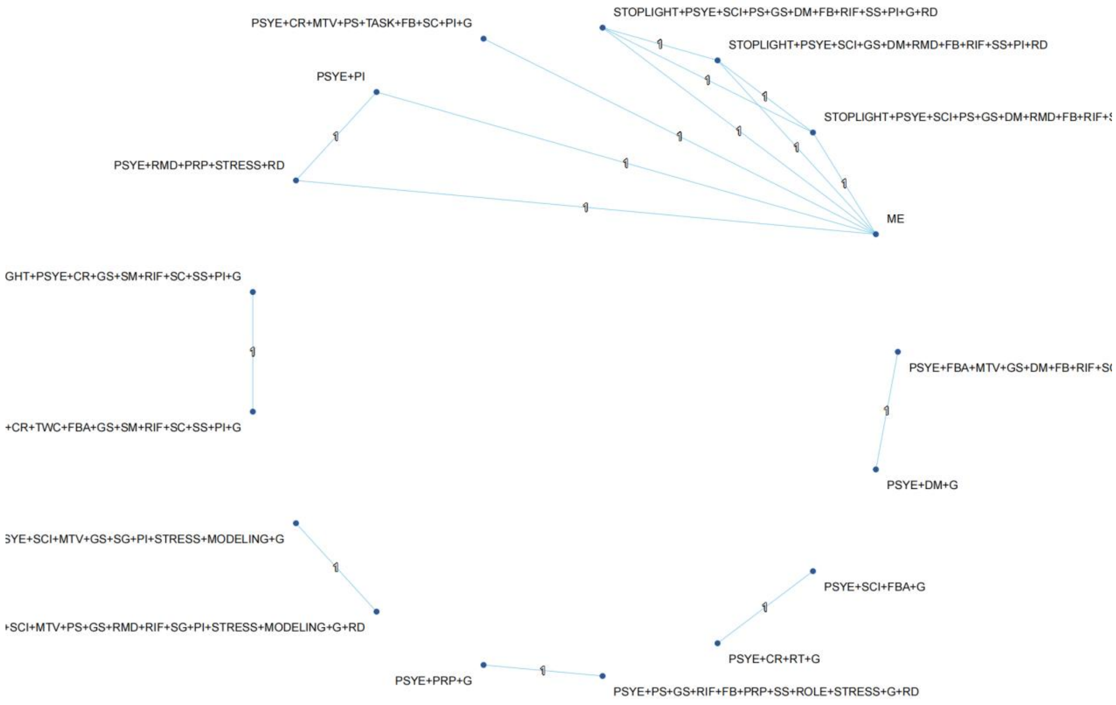

Notes: ME, minimal education; PSYE, psychoeducation; CR, cognitive restructuring; TWC, third-wave components; SCI, self-concept improvement; FBA, functional behavioural analysis; MTV, motivation; GS, goal-setting; TASK, task-setting; RULE, rule-setting; SM, self-monitoring; DM, device-monitoring; RMD, reminders; FB, feedback; PS, problem solving; PRP, preplanning; RIF, reinforcement; CONTRACTING, contracting; MODELING, modelling; SC, stimulus control; RT, relaxation training; IT, inhibition training; SS, social support; STRESS, stress management; STOPLIGHT, stoplight approach; ROLE, role playing; G, group; RD, remote; SG, serious games; PI, parental involvement

4.3 Forest plots for the outcomes in conceptual level NMA

4.3.1 BMI z-score

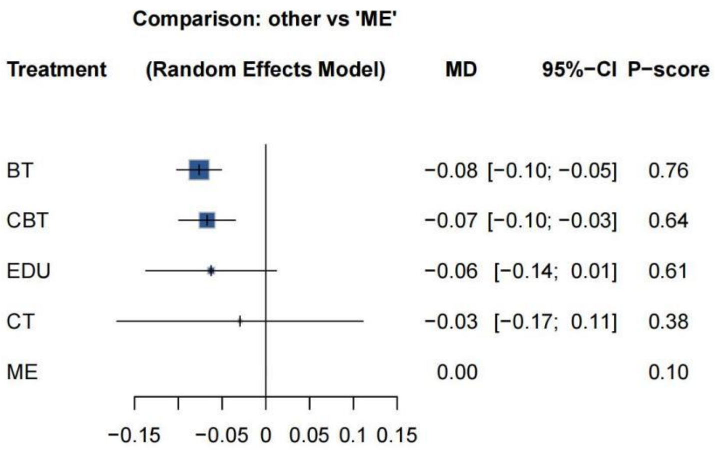

Notes: BT, behavioural therapy; CBT, cognitive behavioural therapy; CT, cognitive therapy; EDU, psychoeducation; ME, minimal education

4.3.2 Body fat

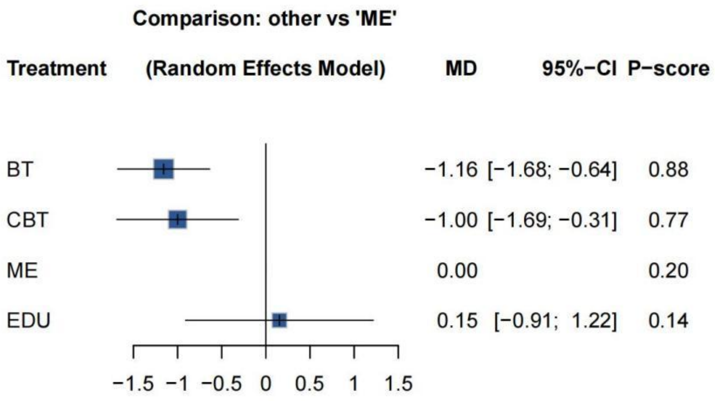

Notes: BT, behavioural therapy; CBT, cognitive behavioural therapy; CT, cognitive therapy; EDU, psychoeducation; ME, minimal education

4.3.3 Waist circumference

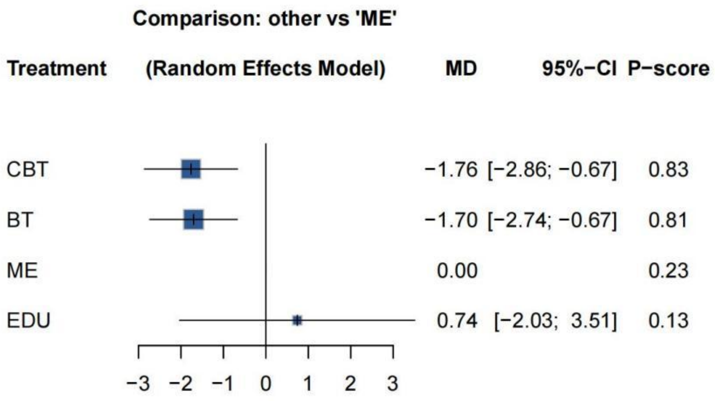

Notes: BT, behavioural therapy; CBT, cognitive behavioural therapy; CT, cognitive therapy; EDU, psychoeducation; ME, minimal education

4.3.4 Height

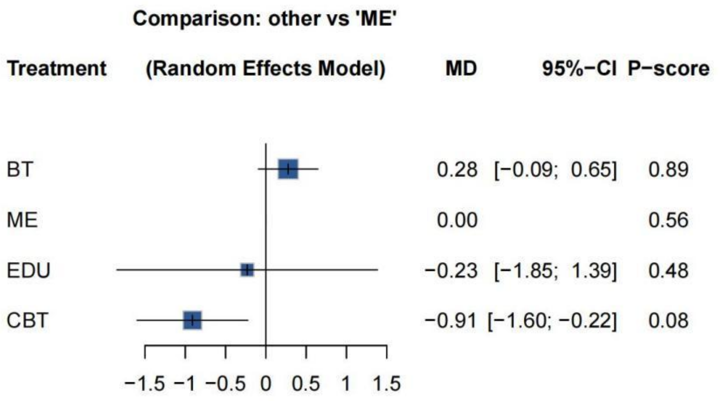

Notes: BT, behavioural therapy; CBT, cognitive behavioural therapy; CT, cognitive therapy; EDU, psychoeducation; ME, minimal education

4.3.5 Quality of life

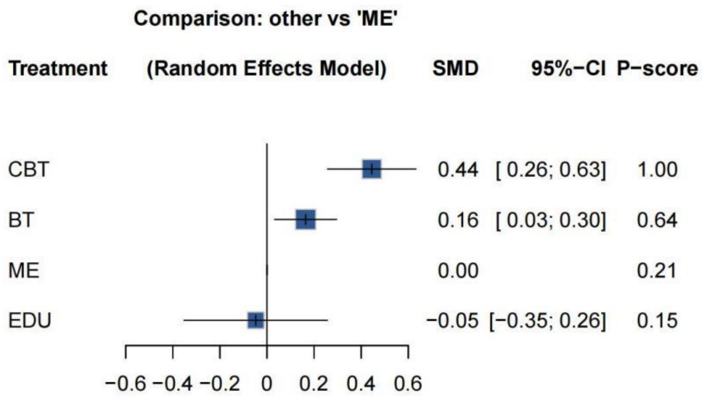

Notes: BT, behavioural therapy; CBT, cognitive behavioural therapy; CT, cognitive therapy; EDU, psychoeducation; ME, minimal education

4.3.6 Mental health

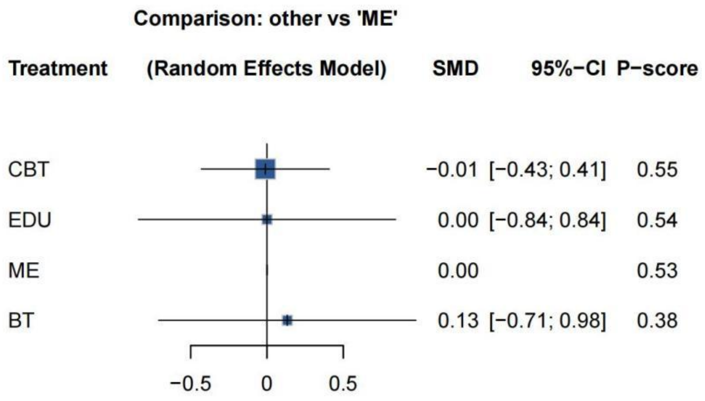

Notes: BT, behavioural therapy; CBT, cognitive behavioural therapy; CT, cognitive therapy; EDU, psychoeducation; ME, minimal education

4.4 Forest plots for the outcomes in technical level CNMA

4.4.1 BMI z-score

Comparison: other vs 'DM+FB'

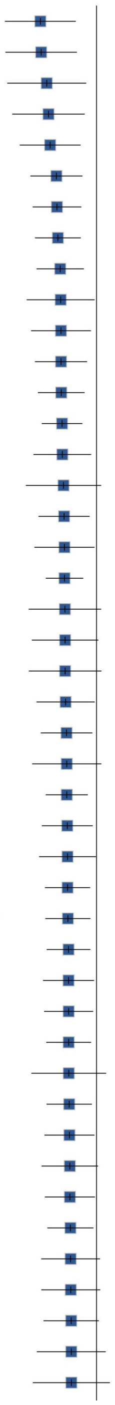

Treatment	(Random Effects Model)	MD	95%−CI
PSYE+TWC+FBA+PS+RIF+SC+PRP+SS+PI+STRESS+RULE		−0.29	[−0.47; −0.11]
PSYE+TWC+FBA+RIF+SC+PRP+SS+PI+STRESS+RULE		−0.28	[−0.47; −0.10]
STOPLIGHT+PSYE+CR+TWC+FBA+GS+SM+RIF+SC+SS+PI+G		−0.26	[−0.46; −0.05]
PSYE+SCI+FBA+MTV+PS+GS+SM+RIF+CONTRACTING+SC+SS+SG+PI+G		−0.25	[−0.43; −0.06]
PSYE+FBA+MTV+GS+SM+RIF+CONTRACTING+SC+PI+G		−0.24	[−0.39; −0.08]
PSYE+GS+FB+RIF+CONTRACTING+SC+PRP+PI+MODELING		−0.21	[−0.34; −0.07]
PSYE+GS+SM+RIF+SC+SG+PI+MODELING+G		−0.20	[−0.33; −0.08]
PSYE+SM+DM+RIF+SC+SG+PI+MODELING		−0.20	[−0.31; −0.08]
PSYE+GS+SM+RIF+SC+PRP+SS+PI+MODELING+G		−0.19	[−0.31; −0.07]
PSYE+TWC+GS+SM+PRP+PI+RULE		−0.18	[−0.36; −0.01]
PSYE+SCI+FBA+MTV+PS+RIF+SC+PRP+SS+PI+G		−0.18	[−0.33; −0.03]
PSYE+GS+TASK+SM+FB+RIF+SC+PI+MODELING+RD		−0.18	[−0.31; −0.05]
PSYE+GS+SM+FB+RIF+SC+SG+PI+MODELING+G		−0.18	[−0.30; −0.06]
PSYE+FBA+MTV+GS+DM+FB+RIF+SC+PI+MODELING+G		−0.18	[−0.28; −0.07]
PSYE+SCI+FBA+MTV+PS+GS+SM+FB+RIF+CONTRACTING+SC+PRP+SS+PI+MODELING		−0.18	[−0.32; −0.03]
STOPLIGHT+PSYE+TWC+FBA+MTV+GS+TASK+SM+RIF+SC+STRESS+RD		−0.17	[−0.36; 0.02]
PSYE+PS+GS+DM+FB+RIF+SC+ROLE+PI+MODELING+RULE+G		−0.17	[−0.30; −0.04]
PSYE+CR+SCI+FBA+MTV+PS+GS+TASK+RMD+FB+RIF+SC+PRP+SS+SG+PI+MODELING+G		−0.16	[−0.32; −0.01]
PSYE+MTV+GS+TASK+DM+FB+SC+PI		−0.16	[−0.26; −0.07]
PSYE+CR+PS+GS+SM+FB+RIF+SC+SS+PI+STRESS+G		−0.16	[−0.35; 0.02]
PSYE+CR+TWC+SCI+PS+GS+SM+DM+RMD+FB+SS+PI+RD		−0.16	[−0.33; 0.01]
PSYE+CR+PS+GS+SM+RIF+SC+PI+STRESS		−0.16	[−0.35; 0.02]
PSYE+CR+FBA+MTV+GS+TASK+SM+SC+PI+G		−0.16	[−0.31; −0.01]
PSYE+PS+GS+SM+CONTRACTING+PI+RD		−0.15	[−0.29; −0.02]
STOPLIGHT+PSYE+CR+GS+SM+RIF+SC+SS+PI+G		−0.15	[−0.33; 0.02]
PSYE+GS+SM+FB+RIF+SC+PI+MODELING+G		−0.15	[−0.26; −0.05]
STOPLIGHT+PSYE+PS+GS+SM+RMD+FB+RIF+CONTRACTING+SC+PI+MODELING+RULE+RD		−0.15	[−0.28; −0.02]
PSYE+GS+TASK+SS+PI+G+RD		−0.15	[−0.29; −0.00]
PSYE+GS+SC+PI+MODELING		−0.15	[−0.26; −0.03]
STOPLIGHT+PSYE+SCI+FBA+MTV+PS+GS+SM+DM+RIF+CONTRACTING+SC+PRP+SS+PI+MODELING+G		−0.15	[−0.26; −0.03]
PSYE+GS+SM+SC+PI+G		−0.14	[−0.25; −0.03]
STOPLIGHT+PSYE+MTV+PS+GS+CONTRACTING+SG+PI		−0.14	[−0.27; −0.01]
STOPLIGHT+PSYE+MTV+PS+GS+SM+DM+RIF+FB+SC+SS+ROLE+PI+MODELING+G		−0.14	[−0.27; −0.02]
STOPLIGHT+PSYE+SCI+FBA+MTV+PS+GS+SM+DM+RIF+CONTRACTING+SC+PRP+SS+PI+MODELING		−0.14	[−0.26; −0.03]
PSYE+SCI+PS+GS+DM+SC+ROLE+PI+RD		−0.14	[−0.33; 0.05]
PSYE+PS+GS+FB+RIF+PI+RULE+RD		−0.14	[−0.26; −0.03]
STOPLIGHT+PSYE+MTV+PS+GS+SM+DM+RIF+FB+SC+SS+ROLE+PI+MODELING		−0.14	[−0.27; −0.01]
PSYE+SCI+MTV+SC+PRP+PI+G		−0.14	[−0.28; 0.01]
PSYE+SM+SC+PRP+PI+G		−0.14	[−0.26; −0.01]
STOPLIGHT+PSYE+MTV+GS+SM+FB+RIF+SC+PI+G		−0.13	[−0.25; −0.02]
PSYE+SCI+MTV+SC+PRP+PI		−0.13	[−0.28; 0.02]
STOPLIGHT+PSYE+PS+TASK+SM+RIF+SC+PRP+PI+G		−0.13	[−0.28; 0.02]
STOPLIGHT+PSYE+PS+GS+SM+FB+RIF+SC+PRP+PI+RD		−0.13	[−0.27; 0.01]
STOPLIGHT+PSYE+TWC+FBA+MTV+PS+GS+TASK+SM+RMD+FB+RIF+SC+STRESS+RD		−0.13	[−0.31; 0.05]
PSYE+TWC+MTV+SM+RIF+SC+RT+IT+PI+MODELING+G		−0.13	[−0.33; 0.07]

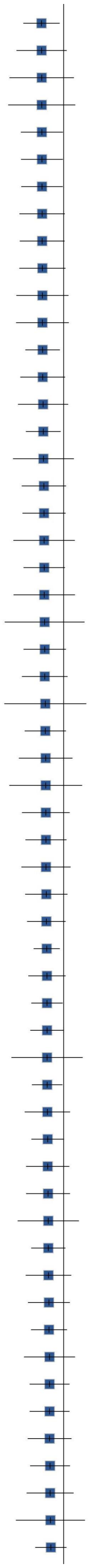

PSYE+MTV+PS+GS+FB+PI+RD	−0.13	[−0.23; −0.02]
STOPLIGHT+PSYE+TASK+SM+RIF+SC+PRP+PI+G	−0.13	[−0.27; 0.02]
PSYE+SCI+MTV+SC+IT+SS+PI+G	−0.13	[−0.31; 0.06]
PSYE+CR+SCI+FBA+MTV+PS+GS+SM+SC+SS+PI+RULE	−0.13	[−0.32; 0.07]
PSYE+PS+GS+FB+SS+PI+G+RD	−0.13	[−0.25; −0.01]
STOPLIGHT+PSYE+GS+TASK+SM+RIF+SC+PI+MODELING+G	−0.13	[−0.25; −0.01]
PSYE+MTV+PS+GS+FB+PI+RULE+RD	−0.13	[−0.25; −0.01]
PSYE+PS+RIF+PI+MODELING+RULE	−0.12	[−0.26; 0.01]
PSYE+RIF+PI+MODELING+RULE+G	−0.12	[−0.25; 0.00]
PSYE+SCI+MTV+PS+GS+TASK+SM+RIF+PRP+SS+SG+PI+STRESS+G	−0.12	[−0.26; 0.01]
PSYE+MTV+GS+TASK+SM+CONTRACTING+SC	−0.12	[−0.27; 0.03]
PSYE+SCI+MTV+PS+GS+RMD+RIF+SG+PI+STRESS+MODELING+G+RD	−0.12	[−0.28; 0.03]
PSYE+MTV+PI+G	−0.12	[−0.22; −0.02]
PSYE+CR+MTV+PS+GS+SM+DM+SC+PRP+SS+SG+G	−0.12	[−0.25; 0.01]
PSYE+SCI+CONTRACTING+SS+PI+G	−0.12	[−0.26; 0.03]
PSYE+MTV+PI	−0.12	[−0.22; −0.02]
PSYE+SCI+PS+GS+DM+SC+ROLE+PI+G	−0.12	[−0.29; 0.06]
STOPLIGHT+PSYE+PS+GS+TASK+FB+RIF+SC+PRP+PI+MODELING	−0.11	[−0.24; 0.01]
PSYE+PS+GS+TASK+FB+RIF+PI+MODELING+G	−0.11	[−0.24; 0.01]
PSYE+SCI+PS+GS+DM+SC+ROLE+PI	−0.11	[−0.29; 0.06]
STOPLIGHT+PSYE+PS+GS+SM+FB+RIF+SC+PI+G	−0.11	[−0.23; 0.01]
PSYE+SCI+GS+SM+SC+SS+PI+RULE	−0.11	[−0.29; 0.06]
PSYE+CR+PS+TASK+SM+SC+IT+PI+MODELING+G	−0.11	[−0.34; 0.12]
PSYE+PS+GS+TASK+FB+RIF+PI+MODELING	−0.11	[−0.23; 0.01]
STOPLIGHT+PSYE+MTV+PS+GS+SM+RIF+SS+PI	−0.11	[−0.24; 0.02]
PSYE+CR+PS+TASK+SM+SC+IT+PI+MODELING	−0.11	[−0.34; 0.13]
PSYE+MTV+GS+SM+RMD+PI+RD+RD	−0.11	[−0.23; 0.01]
STOPLIGHT+PSYE+GS+SM+FB+CONTRACTING+SC+PRP+PI+RULE+G	−0.10	[−0.26; 0.05]
PSYE+SCI+GS+RIF+IT+SS+SG+PI+G	−0.10	[−0.31; 0.11]
STOPLIGHT+PSYE+PS+GS+RIF+SG+PI+MODELING+RULE+G	−0.10	[−0.24; 0.03]
GS+TASK+FB+PI+G	−0.10	[−0.22; 0.02]
STOPLIGHT+PSYE+MTV+PS+GS+SM+RIF+CONTRACTING+PI+STRESS+MODELING	−0.10	[−0.24; 0.04]
STOPLIGHT+PSYE+PS+GS+TASK+SM+FB+RIF+SC+PRP+PI+MODELING+G	−0.10	[−0.22; 0.02]
PSYE+FBA+MTV+PI	−0.10	[−0.21; 0.01]
PSYE+DM+FB+PI+RD	−0.10	[−0.17; −0.02]
PSYE+PS+GS+TASK+SM+DM+PRP+SS+PI+G	−0.10	[−0.20; 0.01]
PSYE+PI+G	−0.10	[−0.19; −0.01]
PSYE+PS+GS+PI	−0.10	[−0.19; 0.00]
PSYE+SCI+MTV+GS+SM+RMD+FB+RIF+IT+SS+SG+PI+G+RD	−0.10	[−0.30; 0.11]
PSYE+MTV+GS+FB+PI	−0.10	[−0.18; −0.01]
PSYE+SCI+MTV+GS+TASK+PRP+SS+PI+G	−0.09	[−0.23; 0.04]
PSYE+PI	−0.09	[−0.19; 0.00]
PSYE+SCI+PS+GS+RIF+PRP+SS+PI+MODELING+RULE	−0.09	[−0.22; 0.03]
PSYE+GS+SM+RIF+SC+G	−0.09	[−0.22; 0.04]
PSYE+IT+PI+G+RD	−0.09	[−0.27; 0.09]
PSYE+PRP+PI+G	−0.09	[−0.19; 0.01]
PSYE+FBA+GS+TASK+PRP+SS+SG+PI+STRESS+MODELING+G	−0.09	[−0.22; 0.04]
PSYE+FBA+PS+GS+TASK+SG+PI+STRESS	−0.09	[−0.21; 0.04]
PSYE+PRP+PI	−0.09	[−0.19; 0.02]
STOPLIGHT+PSYE+GS+SM+FB+RIF+CONTRACTING+PRP+PI+G	−0.08	[−0.23; 0.07]
PSYE+FBA+PS+GS+PI+STRESS+RD	−0.08	[−0.20; 0.03]
PSYE+SM+FB+PI+RD	−0.08	[−0.20; 0.03]
STOPLIGHT+PSYE+PS+GS+RMD+SC+PI+MODELING+G	−0.08	[−0.21; 0.04]
PSYE+SCI+MTV+PI+G	−0.08	[−0.19; 0.04]
PSYE+SCI+PS+GS+SM+FB+SC+PI	−0.08	[−0.21; 0.06]
STOPLIGHT+PSYE+SCI+MTV+PS+GS+TASK+FB+RIF+SC+IT+PRP+SS+ROLE+PI+MODELING+RULE+G	−0.08	[−0.28; 0.12]
PSYE+PS+GS+FB+PI	−0.07	[−0.16; 0.02]

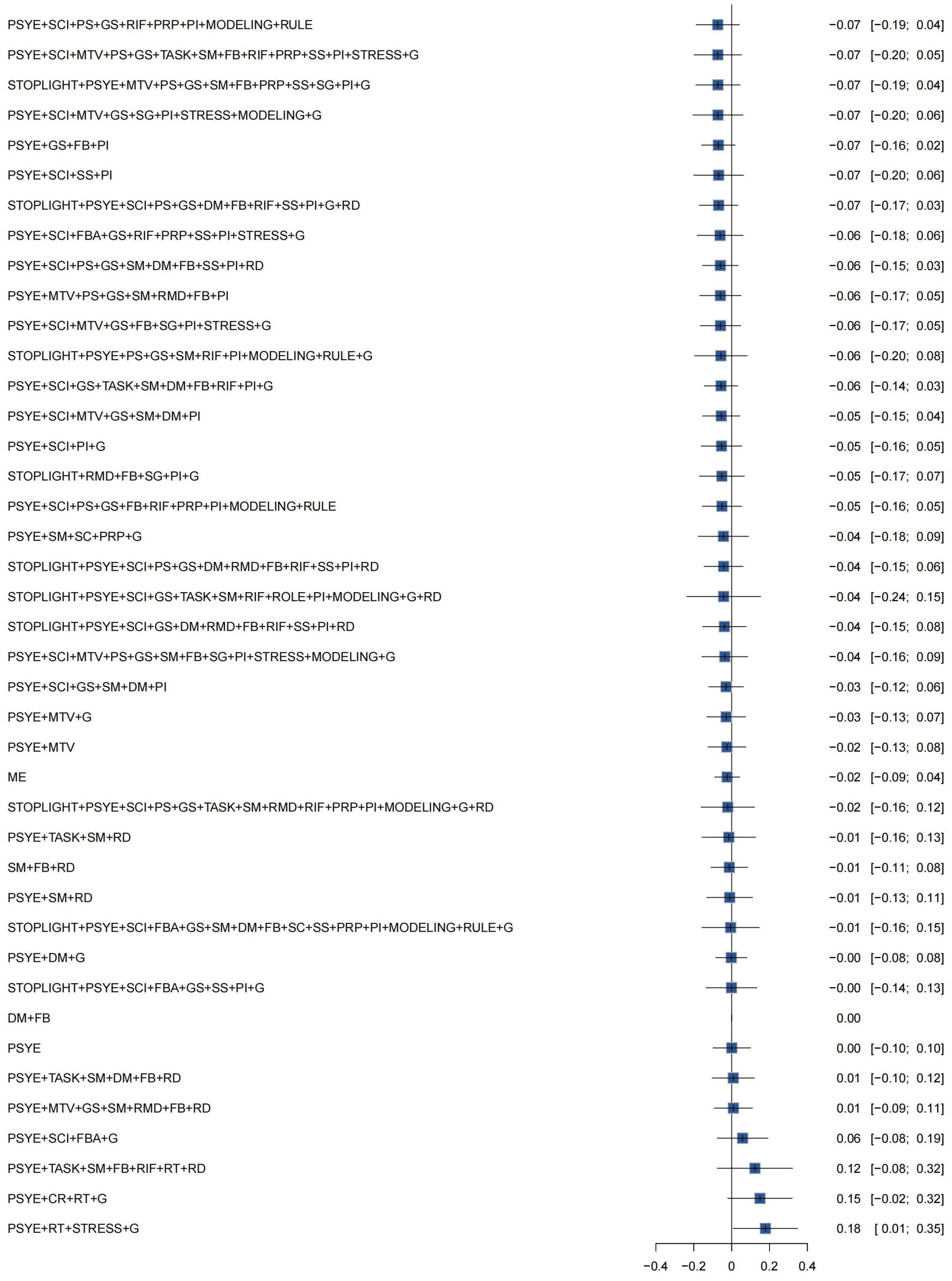

Notes: ME, minimal education; PSYE, psychoeducation; CR, cognitive restructuring; TWC, third-wave components; SCI, self-concept improvement; FBA, functional behavioural analysis; MTV, motivation; GS, goal-setting; TASK, task-setting; RULE, rule-setting; SM, self-monitoring; DM, device-monitoring; RMD, reminders; FB, feedback; PS, problem solving; PRP, preplanning; RIF, reinforcement; CONTRACTING, contracting; MODELING, modelling; SC, stimulus control; RT, relaxation training; IT, inhibition training; SS, social support; STRESS, stress management; STOPLIGHT, stoplight approach; ROLE, role playing; G, group; RD, remote; SG, serious games; PI, parental involvement

4.4.2 Body fat

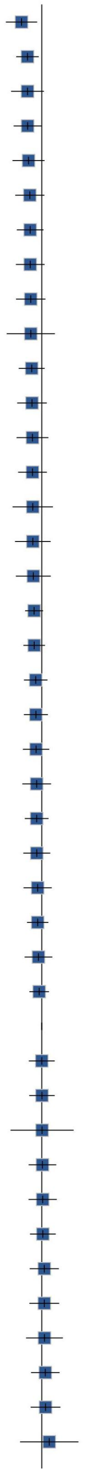

Comparison: other vs 'ME' (Random Effects Model)

Treatment	MD	95%−CI
PSYE+GS+SM+RIF+SC+PRP+SS+PI+MODELING+G	−4.33	[−7.61; −1.05]
PSYE+GS+SM+RIF+SC+SG+PI+MODELING+G	−3.05	[−5.39; −0.71]
PSYE+PRP+G	−3.04	[−6.49; 0.40]
STOPLIGHT+PSYE+SCI+FBA+GS+SM+FB+RIF+SC+SS+PI+G	−3.02	[−5.95; −0.10]
PSYE+CR+MTV+PS+GS+SM+DM+SC+PRP+SS+SG+G	−2.83	[−6.19; 0.53]
PSYE+MTV+TASK+SC+PRP+SG+PI+G	−2.55	[−5.60; 0.50]
PSYE+DM+FB+PI+RD	−2.48	[−5.27; 0.31]
PSYE+SG+PI+G	−2.47	[−5.47; 0.54]
STOPLIGHT+PSYE+SCI+GS+DM+RMD+FB+RIF+SS+PI+RD	−2.36	[−5.43; 0.72]
PSYE+PS+GS+RIF+FB+PRP+SS+ROLE+STRESS+G+RD	−2.34	[−7.43; 2.74]
PSYE+MTV	−2.15	[−4.86; 0.55]
PSYE+CR+FBA+MTV+GS+TASK+SM+SC+PI+G	−2.09	[−5.19; 1.01]
PSYE+MTV+RMD+PI+G+RD	−1.98	[−5.32; 1.35]
PSYE+SCI+MTV+GS+TASK+PRP+SS+PI+G	−1.98	[−4.98; 1.02]
PSYE+CR+SCI+MTV+PS+GS+SM+DM+SC+PRP+PI+G+RD	−1.92	[−6.17; 2.32]
PSYE+PS+GS+SM+FB+CONTRACTING+G+RD	−1.91	[−5.66; 1.83]
PSYE+RMD+PRP+STRESS+RD	−1.81	[−5.47; 1.86]
PSYE+PI	−1.64	[−3.50; 0.21]
PSYE+MTV+PI+G	−1.62	[−3.86; 0.62]
PSYE+FBA+MTV+PI	−1.29	[−3.74; 1.16]
RIF	−1.25	[−3.76; 1.25]
PSYE+PS+GS+FB+PI	−1.21	[−3.97; 1.54]
PSYE+CR+MTV+PS+TASK+FB+SC+PI+G	−1.09	[−4.09; 1.91]
PSYE	−1.09	[−3.56; 1.38]
PSYE+MTV+G	−1.06	[−3.87; 1.75]
PSYE+GS+TASK+SS+PI+G+RD	−0.88	[−3.83; 2.07]
STOPLIGHT+PSYE+MTV+PS+GS+CONTRACTING+SG+PI	−0.87	[−3.09; 1.35]
PSYE+SM+DM+RIF+SC+SG+PI+MODELING	−0.70	[−3.60; 2.20]
PSYE+PI+G	−0.55	[−2.58; 1.47]
ME	0.00	
PSYE+G	0.00	[−2.70; 2.70]
PSYE+PS+GS+FB+RIF+PI+RULE+RD	0.04	[−2.62; 2.70]
PSYE+TASK+SM+FB+RIF+RT+RD	0.06	[−6.61; 6.74]
PSYE+SCI+PS+GS+SM+FB+SC+PI	0.15	[−2.74; 3.03]
STOPLIGHT+PSYE+SCI+PS+GS+DM+FB+RIF+SS+PI+G+RD	0.19	[−2.76; 3.14]
PSYE+MTV+PS+GS+FB+PI+RULE+RD	0.23	[−2.45; 2.92]
PSYE+SCI+MTV+PS+GS+SM+RIF+SS+STRESS+MODELING+G+RD	0.54	[−2.51; 3.59]
STOPLIGHT+PSYE+SCI+PS+GS+DM+RMD+FB+RIF+SS+PI+RD	0.54	[−2.58; 3.66]
PSYE+SCI+FBA+GS+RIF+PRP+SS+PI+STRESS+G	0.58	[−3.30; 4.45]
PSYE+GS+SM+SC+PI+STRESS+G+RD	0.75	[−2.23; 3.74]
STOPLIGHT+PSYE+SCI+FBA+GS+SS+PI+G	0.80	[−2.31; 3.92]
PSYE+MTV+GS+TASK+SM+CONTRACTING+SC	1.60	[−4.57; 7.77]

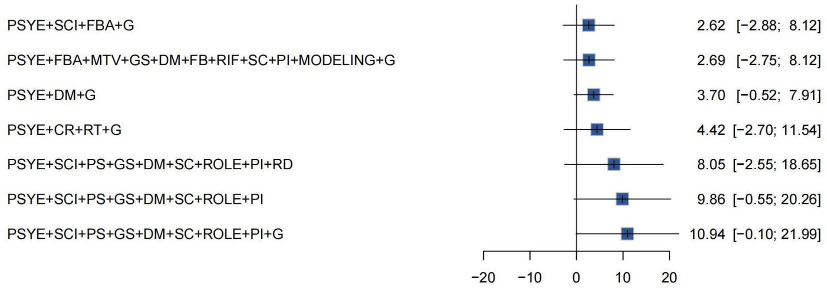

Notes: ME, minimal education; PSYE, psychoeducation; CR, cognitive restructuring; TWC, third-wave components; SCI, self-concept improvement; FBA, functional behavioural analysis; MTV, motivation; GS, goal-setting; TASK, task-setting; RULE, rule-setting; SM, self-monitoring; DM, device-monitoring; RMD, reminders; FB, feedback; PS, problem solving; PRP, preplanning; RIF, reinforcement; CONTRACTING, contracting; MODELING, modelling; SC, stimulus control; RT, relaxation training; IT, inhibition training; SS, social support; STRESS, stress management; STOPLIGHT, stoplight approach; ROLE, role playing; G, group; RD, remote; SG, serious games; PI, parental involvement

4.4.3 Waist circumference

Comparison: other vs 'ME' (Random Effects Model)

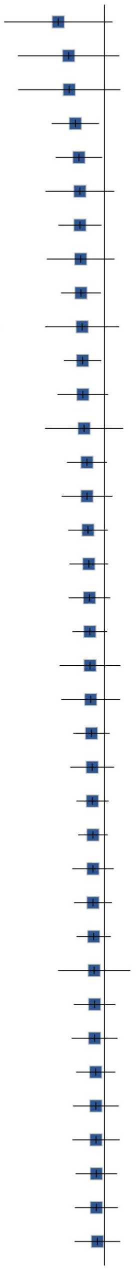

Treatment	MD	95%−CI
PSYE+SCI+PS+GS+DM+SC+ROLE+PI+G	−8.54	[−18.48; 1.41]
PSYE+SCI+PS+GS+DM+SC+ROLE+PI+RD	−6.62	[−15.94; 2.69]
PSYE+SCI+PS+GS+DM+SC+ROLE+PI	−6.52	[−15.90; 2.87]
STOPLIGHT+PSYE+SCI+FBA+GS+SS+PI+G	−5.38	[−9.69; −1.07]
PSYE+SCI+FBA+G	−4.72	[−8.95; −0.48]
PSYE+FBA+MTV+GS+SM+RIF+CONTRACTING+SC+PI+G	−4.54	[−10.84; 1.76]
PSYE+CR+FBA+MTV+GS+TASK+SM+SC+PI+G	−4.53	[−8.47; −0.59]
PSYE+MTV+GS+TASK+SM+CONTRACTING+SC	−4.40	[−10.60; 1.80]
PSYE+SCI+PS+GS+RMD+RIF+SS+PI+G+RD	−4.34	[−7.96; −0.72]
PSYE+SCI+FBA+MTV+PS+GS+SM+RIF+CONTRACTING+SC+SS+SG+PI+G	−4.12	[−10.89; 2.65]
PSYE+MTV+RMD+PI+G+RD	−4.05	[−7.44; −0.65]
PSYE+SCI+MTV+SC+IT+SS+PI+G	−4.00	[−8.62; 0.62]
PSYE+TASK+SM+FB+RIF+RT+RD	−3.76	[−10.92; 3.39]
PSYE+MTV+TASK+SC+SG+PI+G	−3.23	[−6.87; 0.40]
PSYE+SCI+MTV+PS+GS+RMD+RIF+SG+PI+STRESS+MODELING+G+RD	−3.22	[−7.85; 1.41]
PSYE+GS+TASK+SS+PI+G+RD	−3.05	[−6.65; 0.56]
STOPLIGHT+PSYE+SCI+FBA+GS+SM+FB+RIF+SC+SS+PI+G	−2.89	[−6.41; 0.63]
PSYE+PS+GS+TASK+SM+DM+PRP+SS+PI+G	−2.77	[−6.56; 1.01]
TASK+SG+G	−2.70	[−5.86; 0.46]
PSYE+CR+SCI+MTV+PS+GS+SM+DM+SC+PRP+PI+G+RD	−2.66	[−8.23; 2.90]
PSYE+CR+RT+G	−2.55	[−7.94; 2.84]
STOPLIGHT+PSYE+MTV+PS+GS+CONTRACTING+SG+PI	−2.40	[−5.71; 0.91]
PSYE+SCI+MTV+GS+SM+DM+PI	−2.27	[−6.25; 1.72]
PSYE+MTV+G	−2.23	[−5.15; 0.69]
PSYE+MTV+PI+G	−2.13	[−4.80; 0.53]
PSYE+MTV+GS+TASK+DM+FB+SC+PI	−2.13	[−5.91; 1.66]
PSYE+MTV+PS+GS+FB+PI	−2.12	[−5.57; 1.32]
PSYE+FBA+MTV+PI	−2.00	[−5.10; 1.11]
PSYE+PS+GS+RIF+FB+PRP+SS+ROLE+STRESS+G+RD	−1.90	[−8.52; 4.72]
STOPLIGHT+RMD+FB+SG+PI+G	−1.83	[−5.62; 1.96]
PSYE+SCI+FBA+GS+RIF+PRP+SS+PI+STRESS+G	−1.83	[−6.01; 2.35]
PSYE+SCI+MTV+GS+FB+SG+PI+STRESS+G	−1.62	[−5.18; 1.95]
PSYE+TWC+FBA+PS+RIF+SC+PRP+SS+PI+STRESS+RULE	−1.59	[−5.76; 2.58]
PSYE+SCI+MTV+GS+SG+PI+STRESS+MODELING+G	−1.56	[−5.88; 2.76]
STOPLIGHT+PSYE+MTV+PS+GS+SM+FB+PRP+SS+SG+PI+G	−1.50	[−5.27; 2.28]
PSYE+SCI+PS+GS+FB+RIF+PRP+PI+MODELING+RULE	−1.49	[−5.41; 2.43]
PSYE+PRP+G	−1.30	[−5.46; 2.85]

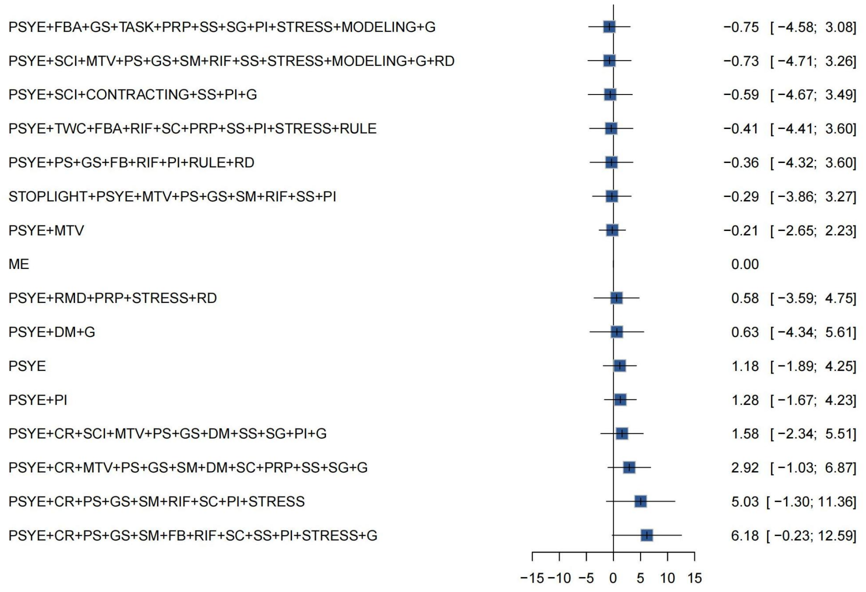

Notes: ME, minimal education; PSYE, psychoeducation; CR, cognitive restructuring; TWC, third-wave components; SCI, self-concept improvement; FBA, functional behavioural analysis; MTV, motivation; GS, goal-setting; TASK, task-setting; RULE, rule-setting; SM, self-monitoring; DM, device-monitoring; RMD, reminders; FB, feedback; PS, problem solving; PRP, preplanning; RIF, reinforcement; CONTRACTING, contracting; MODELING, modelling; SC, stimulus control; RT, relaxation training; IT, inhibition training; SS, social support; STRESS, stress management; STOPLIGHT, stoplight approach; ROLE, role playing; G, group; RD, remote; SG, serious games; PI, parental involvement

4.4.4 Height

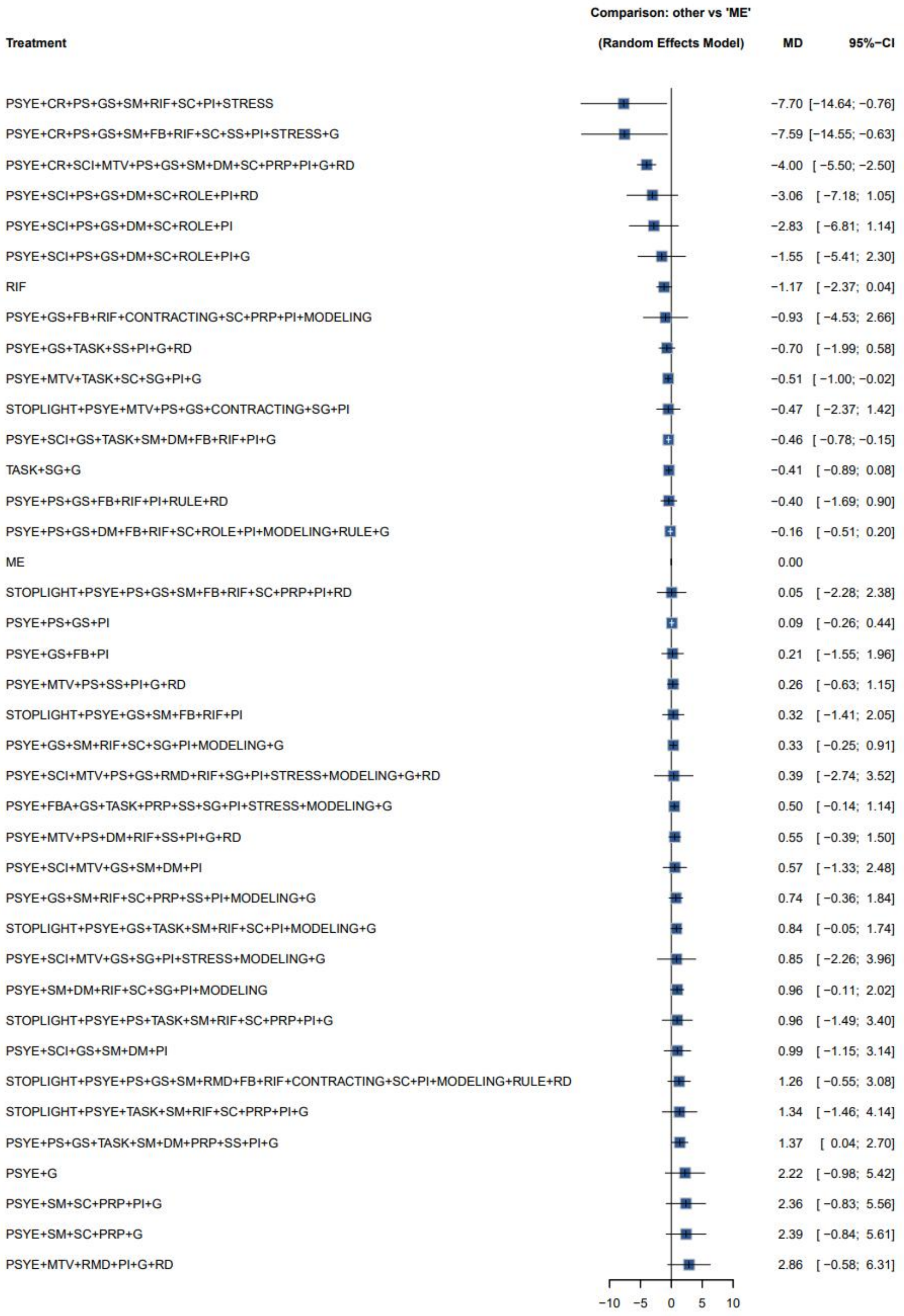

Notes: ME, minimal education; PSYE, psychoeducation; CR, cognitive restructuring; TWC, third-wave components; SCI, self-concept improvement; FBA, functional behavioural analysis; MTV, motivation; GS, goal-setting; TASK, task-setting; RULE, rule-setting; SM, self-monitoring; DM, device-monitoring; RMD, reminders; FB, feedback; PS, problem solving; PRP, preplanning; RIF, reinforcement; CONTRACTING, contracting; MODELING, modelling; SC, stimulus control; RT, relaxation training; IT, inhibition training; SS, social support; STRESS, stress management; STOPLIGHT, stoplight approach; ROLE, role playing; G, group; RD, remote; SG, serious games; PI, parental involvement

4.4.5 Quality of life

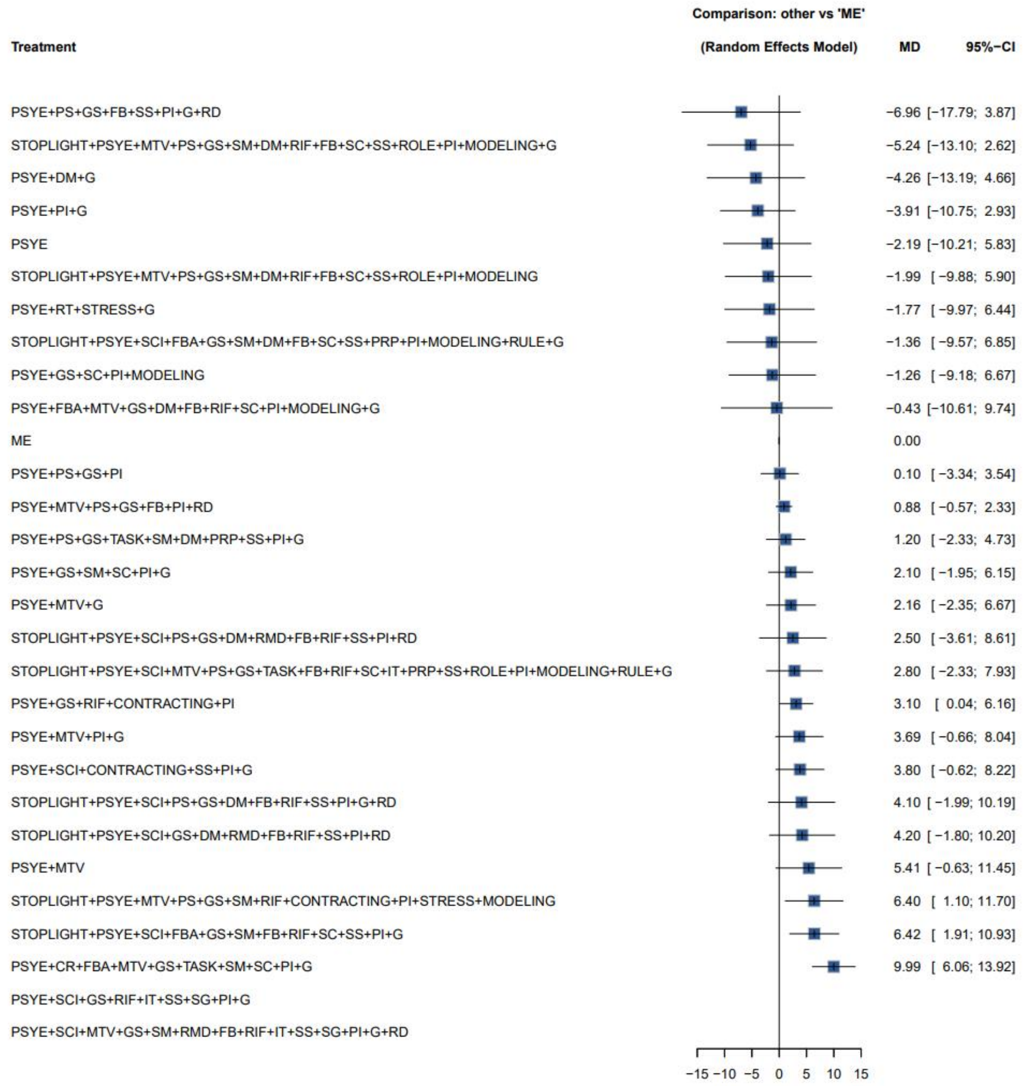

Notes: ME, minimal education; PSYE, psychoeducation; CR, cognitive restructuring; TWC, third-wave components; SCI, self-concept improvement; FBA, functional behavioural analysis; MTV, motivation; GS, goal-setting; TASK, task-setting; RULE, rule-setting; SM, self-monitoring; DM, device-monitoring; RMD, reminders; FB, feedback; PS, problem solving; PRP, preplanning; RIF, reinforcement; CONTRACTING, contracting; MODELING, modelling; SC, stimulus control; RT, relaxation training; IT, inhibition training; SS, social support; STRESS, stress management; STOPLIGHT, stoplight approach; ROLE, role playing; G, group; RD, remote; SG, serious games; PI, parental involvement

4.4.6 Mental health

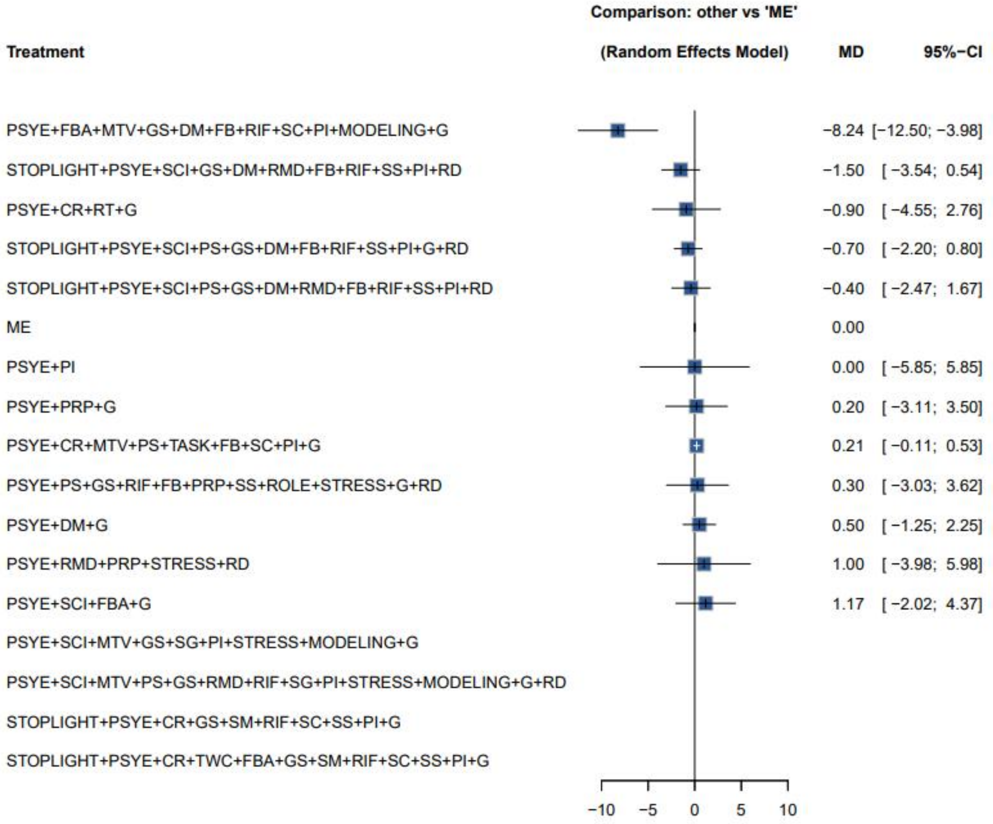

Notes: ME, minimal education; PSYE, psychoeducation; CR, cognitive restructuring; TWC, third-wave components; SCI, self-concept improvement; FBA, functional behavioural analysis; MTV, motivation; GS, goal-setting; TASK, task-setting; RULE, rule-setting; SM, self-monitoring; DM, device-monitoring; RMD, reminders; FB, feedback; PS, problem solving; PRP, preplanning; RIF, reinforcement; CONTRACTING, contracting; MODELING, modelling; SC, stimulus control; RT, relaxation training; IT, inhibition training; SS, social support; STRESS, stress management; STOPLIGHT, stoplight approach; ROLE, role playing; G, group; RD, remote; SG, serious games; PI, parental involvement.

4.5 Pairwise forest plots for the outcomes in conceptual level NMA

4.5.1 BMI z-score

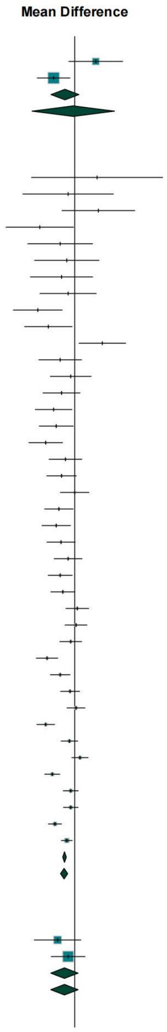

Study	Experimental Total	Experimental Mean	Experimental SD	Control Total	Control Mean	Control SD	MD [95%−CI]	Weight (common)	Weight (random)
**BT:EDU**									
Vermeiren−2021	90	−0.40	0.7018	86	−0.56	0.6833	0.16 [−0.04; 0.36]	26.9%	46.6%
Freira_2018	46	−0.12	0.3650	51	0.04	0.2383	−0.16 [−0.28; −0.04]	73.1%	53.4%
**Common effect model**	**136**			**137**			**−0.07 [−0.18; 0.03]**	**100.0%**	.
**Random effects model**							**−0.01 [−0.32; 0.30]**	.	**100.0%**
Heterogeneity: I^2 = 85.4%, τ^2 = 0.0437, p = 0.0088									
**BT:ME**									
Hinton_2018	10	0.07	0.7174	9	−0.10	0.3422	0.17 [−0.33; 0.67]	0.1%	0.3%
Looney_2014	14	−0.12	0.3070	8	−0.07	0.4389	−0.05 [−0.39; 0.29]	0.2%	0.5%
Walpole−2013	20	−0.05	0.3323	20	−0.23	0.5359	0.18 [−0.10; 0.46]	0.3%	0.8%
Cohen_2016	50	−0.34	0.6380	28	−0.07	0.5000	−0.26 [−0.52; −0.01]	0.3%	0.9%
Waling_2012	48	−0.29	0.6200	45	−0.18	0.5900	−0.11 [−0.36; 0.14]	0.3%	1.0%
Smith−2021	141	−0.06	1.0679	99	0.00	0.8607	−0.06 [−0.30; 0.18]	0.3%	1.0%
Wafa−2011	52	0.00	0.7200	55	0.10	0.5000	−0.10 [−0.34; 0.14]	0.3%	1.0%
Cohen, T. R. _2023	30	−0.10	0.3321	30	−0.05	0.4968	−0.05 [−0.26; 0.16]	0.4%	1.2%
Tsai−2024	82	−0.08	0.6057	82	0.20	0.6069	−0.28 [−0.47; −0.09]	0.6%	1.5%
O'Connor_2013	20	−0.10	0.2700	20	0.10	0.3100	−0.20 [−0.38; −0.02]	0.6%	1.5%
Love−Osborne_2014_1	20	0.14	0.3000	23	−0.07	0.2900	0.21 [0.03; 0.39]	0.6%	1.5%
Saelens_2002	23	−0.05	0.3181	21	0.06	0.2263	−0.11 [−0.27; 0.05]	0.7%	1.7%
Maddison_2014	127	0.01	0.5896	124	0.04	0.6612	−0.03 [−0.19; 0.13]	0.8%	1.8%
Kalavainen_2007	35	−0.20	0.3000	35	−0.10	0.3000	−0.10 [−0.24; 0.04]	1.0%	2.0%
Sauder_2018	29	−0.17	0.2700	33	−0.01	0.2900	−0.16 [−0.30; −0.02]	1.0%	2.1%
Hofsteenge, G. H._2014	71	−0.12	0.3326	51	0.02	0.3766	−0.14 [−0.27; −0.01]	1.2%	2.2%
Ford, A. L._2009	54	−0.36	0.3600	52	−0.14	0.3100	−0.22 [−0.35; −0.09]	1.2%	2.3%
McCallum_2007	82	−0.04	0.4238	81	0.03	0.3839	−0.07 [−0.19; 0.05]	1.3%	2.3%
Wake−−2013	62	−0.20	0.3536	56	−0.10	0.2646	−0.10 [−0.21; 0.01]	1.5%	2.5%
Love−Osborne_2014_2	40	−0.04	0.2600	35	−0.04	0.2200	0.00 [−0.11; 0.11]	1.6%	2.6%
Gillis, D._2007	14	−0.04	0.1900	13	0.07	0.0800	−0.12 [−0.23; −0.01]	1.6%	2.6%
Pakpour_2015	238	−0.13	0.5070	119	0.01	0.4852	−0.14 [−0.25; −0.03]	1.7%	2.6%
Markert_2014	145	−0.09	0.5800	144	0.02	0.3100	−0.10 [−0.21; 0.00]	1.7%	2.6%
Stark_2019	97	−0.18	0.3300	54	−0.13	0.3100	−0.05 [−0.16; 0.06]	1.7%	2.7%
Taylor−2015	104	−0.19	0.3652	102	−0.08	0.3007	−0.11 [−0.20; −0.02]	2.3%	3.0%
Luque_2024	106	−0.36	0.3500	95	−0.27	0.3100	−0.09 [−0.18; 0.00]	2.3%	3.0%
Arauz_2013	14	−0.03	0.1400	12	−0.05	0.0800	0.02 [−0.07; 0.11]	2.6%	3.1%
Christie_2017	87	−0.02	0.2800	87	−0.03	0.2800	0.01 [−0.07; 0.09]	2.8%	3.2%
Serra Paya_2015	54	−0.12	0.2200	59	−0.09	0.2300	−0.03 [−0.11; 0.05]	2.8%	3.2%
Johnston_2010	40	−0.16	0.1900	20	0.05	0.1300	−0.21 [−0.29; −0.13]	2.9%	3.2%
Broccoli_2016	187	−0.12	0.3800	185	−0.01	0.3500	−0.11 [−0.18; −0.04]	3.5%	3.4%
Yackobovitch−Gavan−2018_1	173	−0.06	0.2750	74	−0.03	0.2600	−0.04 [−0.11; 0.04]	3.7%	3.4%
Crespo, N. C._2018	149	−0.07	0.2938	148	−0.08	0.3046	0.01 [−0.06; 0.08]	4.2%	3.5%
Savoye_2011	105	−0.21	0.2100	69	0.01	0.2300	−0.22 [−0.29; −0.15]	4.3%	3.5%
Hughes, A. R._2008	69	−0.12	0.1700	65	−0.08	0.2000	−0.04 [−0.10; 0.02]	4.9%	3.6%
Love−Osborne_2014_3	17	0.02	0.0700	14	−0.02	0.1000	0.04 [−0.02; 0.10]	5.0%	3.6%
Fullerton, G._2007	40	−0.13	0.1400	40	0.04	0.1200	−0.17 [−0.23; −0.11]	5.9%	3.7%
Taveras_2017	360	−0.09	0.3900	361	−0.06	0.3900	−0.03 [−0.09; 0.03]	6.0%	3.7%
Wright_2013	24	−0.06	0.1000	26	−0.03	0.1000	−0.03 [−0.09; 0.03]	6.3%	3.8%
Arlinghaus_2019	180	−0.16	0.2300	63	−0.01	0.1500	−0.15 [−0.20; −0.10]	7.8%	3.9%
Taveras_2015	365	−0.10	0.2470	184	−0.04	0.2245	−0.06 [−0.10; −0.02]	11.5%	4.1%
**Common effect model**	**3578**			**2841**			**−0.08 [−0.09; −0.06]**	**100.0%**	.
**Random effects model**							**−0.08 [−0.11; −0.05]**	.	**100.0%**
Heterogeneity: I^2 = 67%, τ^2 = 0.0043, p < 0.0001									
**CBT:BT**									
Fedele_2018	14	−0.12	0.2400	10	0.01	0.2000	−0.13 [−0.31; 0.05]	34.6%	34.6%
Jelalian_2011	45	−0.21	0.3145	44	−0.16	0.3043	−0.05 [−0.18; 0.08]	65.4%	65.4%
**Common effect model**	**59**			**54**			**−0.08 [−0.18; 0.03]**	**100.0%**	.
**Random effects model**							**−0.08 [−0.18; 0.03]**	.	**100.0%**
Heterogeneity: I^2 = 0%, τ^2 = 0, p = 0.4728									

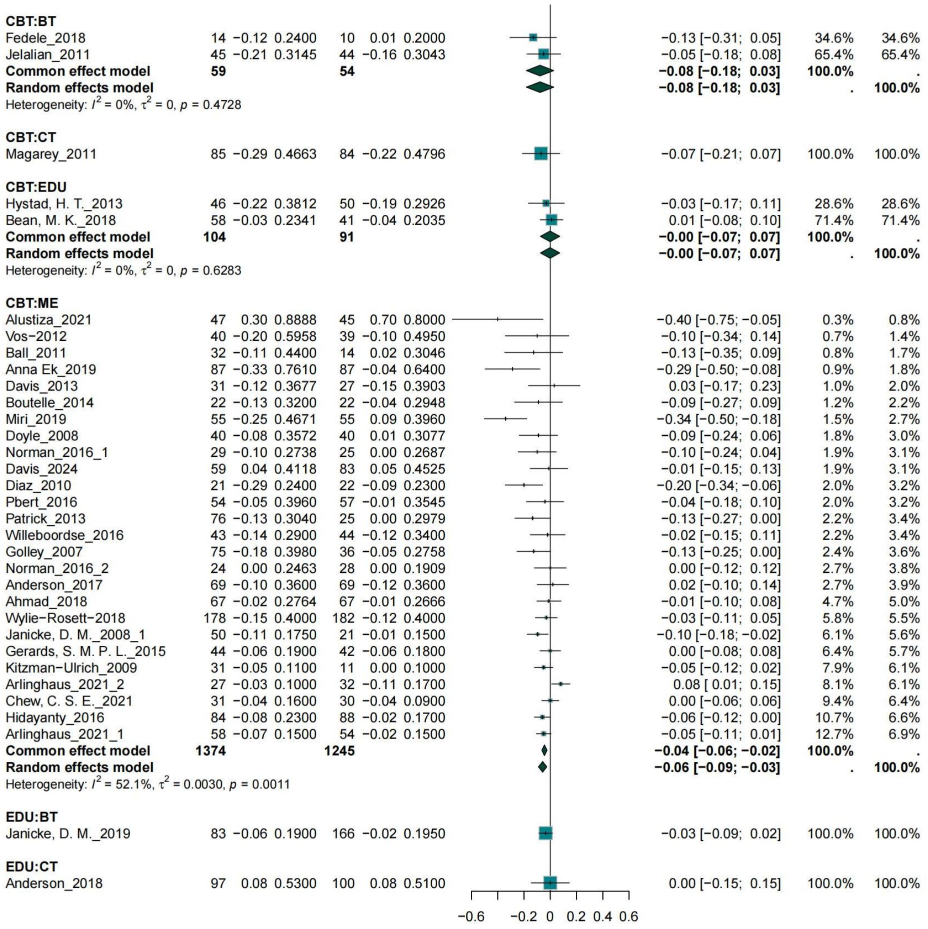

Notes: BT, behavioural therapy; CBT, cognitive behavioural therapy; CT, cognitive therapy; EDU, psychoeducation; ME, minimal education

4.5.2 Body fat

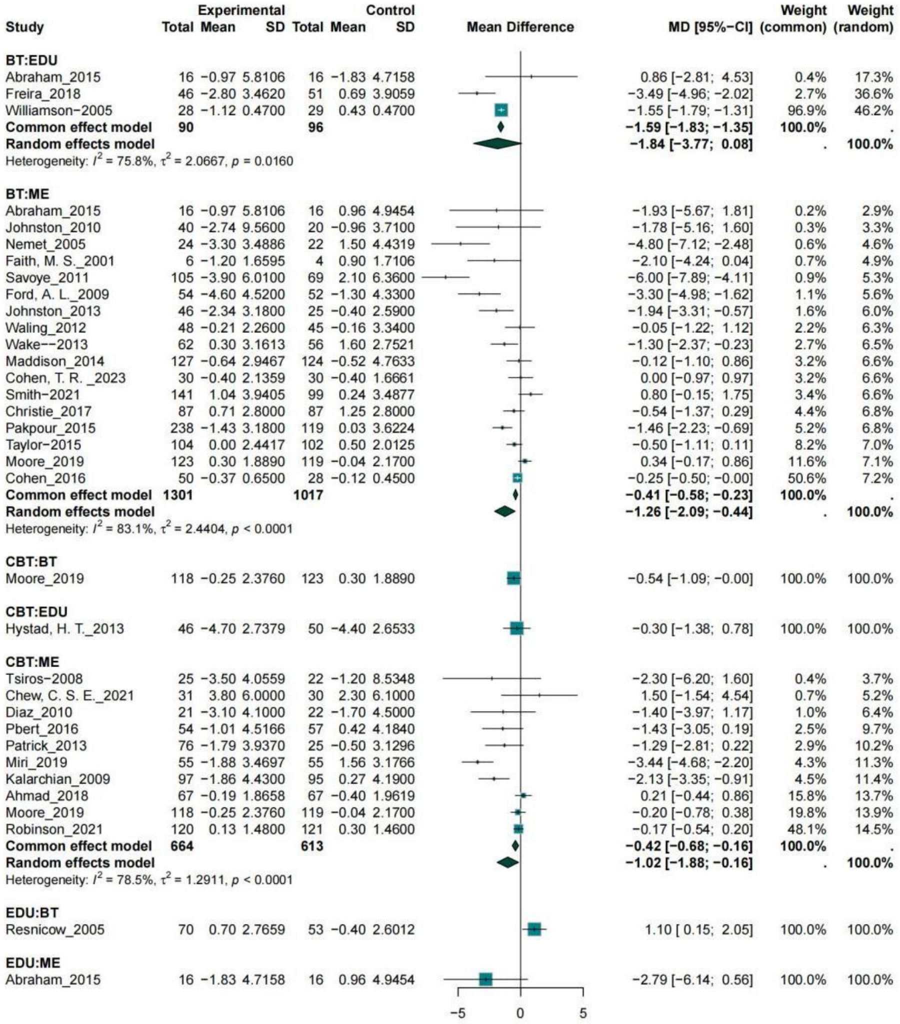

Notes: BT, behavioural therapy; CBT, cognitive behavioural therapy; CT, cognitive therapy; EDU, psychoeducation; ME, minimal education

4.5.3 Waist circumference

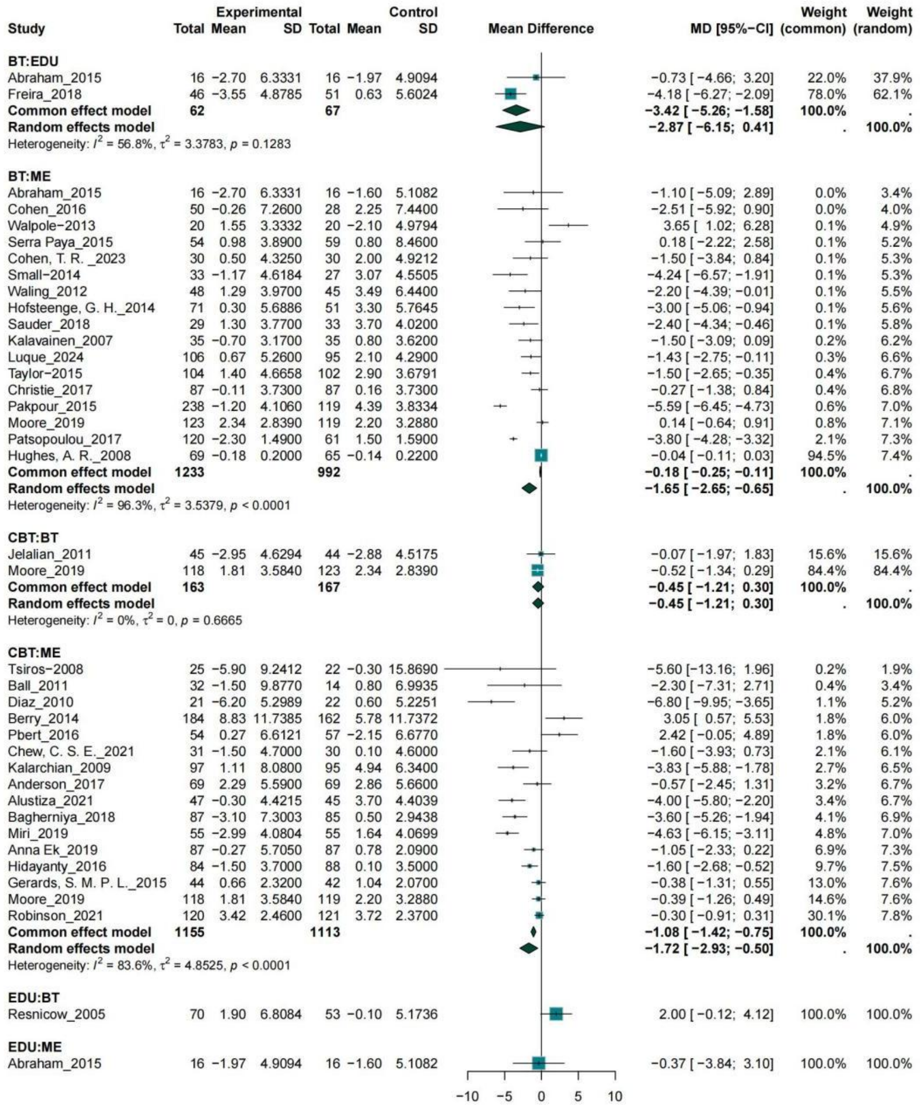

Notes: BT, behavioural therapy; CBT, cognitive behavioural therapy; CT, cognitive therapy; EDU, psychoeducation; ME, minimal education

4.5.4 Height

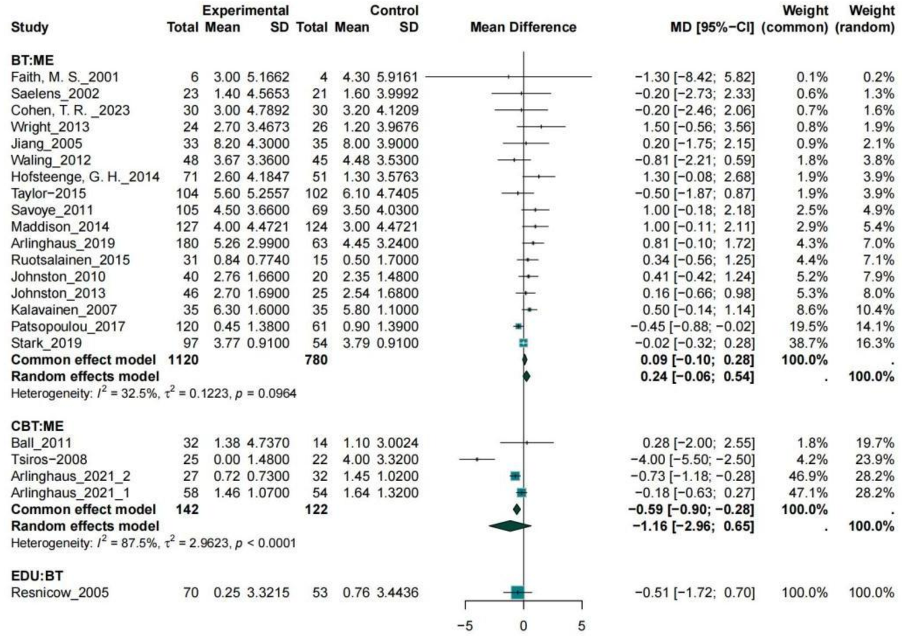

Notes: BT, behavioural therapy; CBT, cognitive behavioural therapy; CT, cognitive therapy; EDU, psychoeducation; ME, minimal education

4.5.5 Quality of life

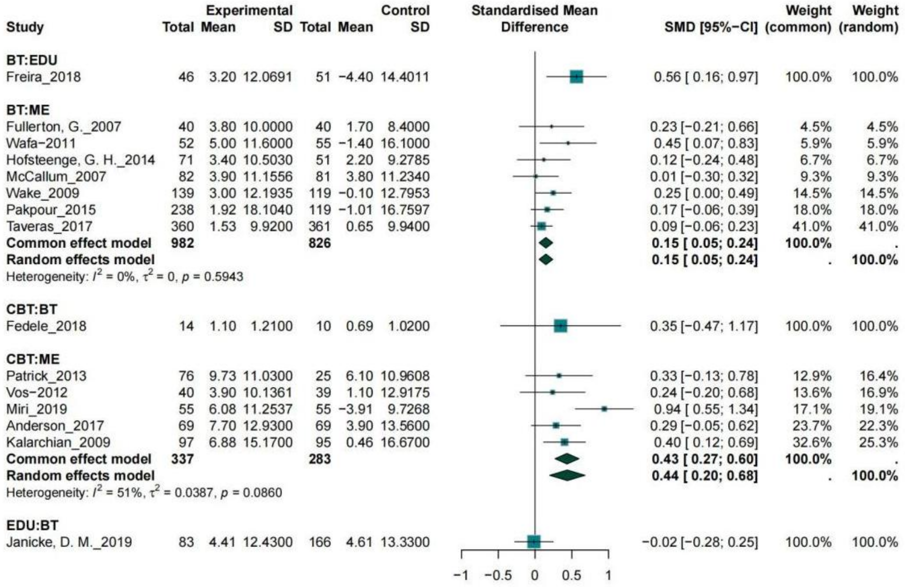

Notes: BT, behavioural therapy; CBT, cognitive behavioural therapy; CT, cognitive therapy; EDU, psychoeducation; ME, minimal education

4.5.6 Mental health

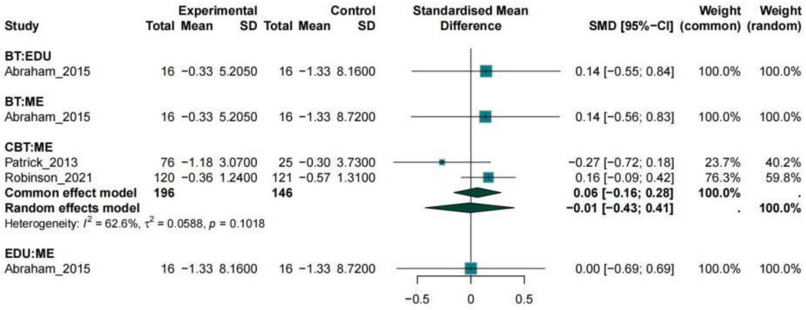

Notes: BT, behavioural therapy; CBT, cognitive behavioural therapy; CT, cognitive therapy; EDU, psychoeducation; ME, minimal education

4.6 Pairwise forest plots for the outcomes in technical level CNMA

4.6.1 BMI z-score

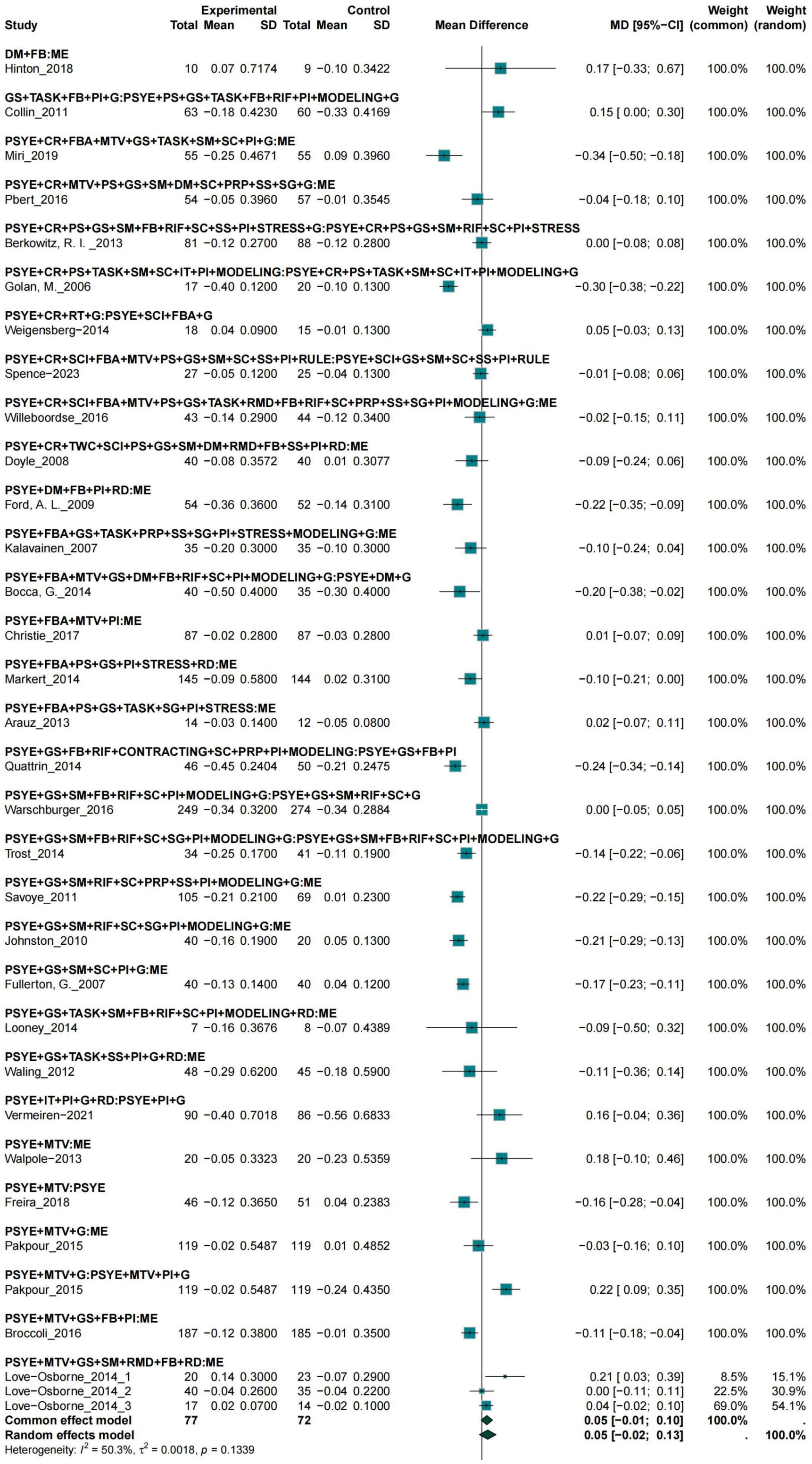

**PSYE+MTV+GS+SM+RMD+PI+RD+RD:ME**									
Taveras_2015	171	−0.09	0.2345	184	−0.04	0.2245	−0.05 [−0.10; −0.00]	100.0%	100.0%
**PSYE+MTV+GS+TASK+DM+FB+SC+PI:ME**									
Luque_2024	106	−0.36	0.3500	95	−0.27	0.3100	−0.09 [−0.18; 0.00]	100.0%	100.0%
**PSYE+MTV+PI:ME**									
Taveras_2015	194	−0.11	0.2581	184	−0.04	0.2245	−0.07 [−0.12; −0.02]	100.0%	100.0%
**PSYE+MTV+PI:PSYE+MTV+GS+SM+RMD+PI+RD+RD**									
Taveras_2015	194	−0.11	0.2581	171	−0.09	0.2345	−0.02 [−0.07; 0.03]	100.0%	100.0%
**PSYE+MTV+PI+G:ME**									
Pakpour_2015	119	−0.24	0.4350	119	0.01	0.4852	−0.25 [−0.37; −0.13]	100.0%	100.0%
**PSYE+MTV+PS+GS+FB+PI+RD:ME**									
Taveras_2017	360	−0.09	0.3900	361	−0.06	0.3900	−0.03 [−0.09; 0.03]	100.0%	100.0%
**PSYE+MTV+PS+GS+FB+PI+RULE+RD:ME**									
Smith−2021	141	−0.06	1.0679	99	0.00	0.8607	−0.06 [−0.30; 0.18]	100.0%	100.0%
**PSYE+MTV+PS+GS+SM+RMD+FB+PI:ME**									
Tsai−2024	82	−0.08	0.6057	82	0.20	0.6069	−0.28 [−0.47; −0.09]	100.0%	100.0%
**PSYE+PI:PSYE+SCI+PI+G**									
Anderson_2018	97	0.08	0.5300	100	0.08	0.5100	0.00 [−0.15; 0.15]	100.0%	100.0%
**PSYE+PI+G:STOPLIGHT+PSYE+MTV+PS+GS+SM+DM+RIF+FB+SC+SS+ROLE+PI+MODELING**									
Janicke, D. M._2019	83	−0.06	0.1900	78	−0.03	0.2000	−0.03 [−0.09; 0.03]	100.0%	100.0%
**PSYE+PI+G:STOPLIGHT+PSYE+MTV+PS+GS+SM+DM+RIF+FB+SC+SS+ROLE+PI+MODELING+G**									
Janicke, D. M._2019	83	−0.06	0.1900	88	−0.02	0.1900	−0.04 [−0.10; 0.02]	100.0%	100.0%
**PSYE+PRP+PI:ME**									
Yackobovitch−Gavan−2018_1	89	−0.08	0.2300	74	−0.03	0.2600	−0.05 [−0.13; 0.03]	100.0%	100.0%
**PSYE+PRP+PI:PSYE+PRP+PI+G**									
Yackobovitch−Gavan−2018_1	89	−0.08	0.2300	84	−0.05	0.2300	−0.03 [−0.10; 0.04]	100.0%	100.0%
**PSYE+PRP+PI+G:ME**									
Yackobovitch−Gavan−2018_1	84	−0.05	0.2300	74	−0.03	0.2600	−0.02 [−0.10; 0.06]	100.0%	100.0%
**PSYE+PS+GS+DM+FB+RIF+SC+ROLE+PI+MODELING+RULE+G:ME**									
Stark_2019	47	−0.32	0.3300	54	−0.13	0.3100	−0.19 [−0.32; −0.06]	100.0%	100.0%
**PSYE+PS+GS+DM+FB+RIF+SC+ROLE+PI+MODELING+RULE+G:PSYE+PS+GS+PI**									
Stark_2019	47	−0.32	0.3300	50	−0.05	0.2700	−0.27 [−0.39; −0.15]	100.0%	100.0%
**PSYE+PS+GS+FB+PI:ME**									
Wake−−2013	62	−0.20	0.3536	56	−0.10	0.2646	−0.10 [−0.21; 0.01]	100.0%	100.0%
**PSYE+PS+GS+FB+RIF+PI+RULE+RD:ME**									
Taylor−2015	104	−0.19	0.3652	102	−0.08	0.3007	−0.11 [−0.20; −0.02]	100.0%	100.0%
**PSYE+PS+GS+FB+SS+PI+G+RD:PSYE+GS+SC+PI+MODELING**									
Zoellner−2022	70	0.03	0.6800	69	0.00	0.6800	0.03 [−0.20; 0.26]	100.0%	100.0%
**PSYE+PS+GS+PI:ME**									
McCallum_2007	82	−0.04	0.4238	81	0.03	0.3839	−0.07 [−0.19; 0.05]	44.7%	48.3%
Stark_2019	50	−0.05	0.2700	54	−0.13	0.3100	0.08 [−0.03; 0.19]	55.3%	51.7%
**Common effect model**	**132**			**135**			**0.01 [−0.07; 0.10]**	**100.0%**	**.**
**Random effects model**							**0.01 [−0.14; 0.15]**	**.**	**100.0%**
Heterogeneity: I^2 = 67.8%, τ^2 = 0.0076, p = 0.0781									
**PSYE+PS+GS+SM+CONTRACTING+PI+RD:ME**									
O'Connor_2013	20	−0.10	0.2700	20	0.10	0.3100	−0.20 [−0.38; −0.02]	100.0%	100.0%
**PSYE+PS+GS+TASK+FB+RIF+PI+MODELING:GS+TASK+FB+PI+G**									
Collin_2011	42	−0.32	0.3022	63	−0.18	0.4230	−0.14 [−0.28; −0.00]	100.0%	100.0%
**PSYE+PS+GS+TASK+FB+RIF+PI+MODELING:PSYE+PS+GS+TASK+FB+RIF+PI+MODELING+G**									
Collin_2011	42	−0.32	0.3022	60	−0.33	0.4169	0.01 [−0.13; 0.15]	100.0%	100.0%
**PSYE+PS+GS+TASK+SM+DM+PRP+SS+PI+G:ME**									
Hofsteenge, G. H._2014	71	−0.12	0.3326	51	0.02	0.3766	−0.14 [−0.27; −0.01]	100.0%	100.0%
**PSYE+PS+RIF+PI+MODELING+RULE:PSYE+RIF+PI+MODELING+RULE+G**									
Bohlin_2017	19	−0.16	0.3900	18	−0.12	0.4300	−0.04 [−0.30; 0.22]	100.0%	100.0%
**PSYE+SCI+CONTRACTING+SS+PI+G:ME**									
Anderson_2017	69	−0.10	0.3600	69	−0.12	0.3600	0.02 [−0.10; 0.14]	100.0%	100.0%
**PSYE+SCI+FBA+GS+RIF+PRP+SS+PI+STRESS+G:ME**									
Chew, C. S. E._2021	31	−0.04	0.1600	30	−0.04	0.0900	0.00 [−0.06; 0.06]	100.0%	100.0%
**PSYE+SCI+FBA+MTV+PS+GS+SM+RIF+CONTRACTING+SC+SS+SG+PI+G:PSYE+FBA+MTV+GS+SM+RIF+CONTRACTING+SC+PI+G**									
Jelalian_2011	45	−0.21	0.3145	44	−0.16	0.3043	−0.05 [−0.18; 0.08]	100.0%	100.0%
**PSYE+SCI+FBA+MTV+PS+RIF+SC+PRP+SS+PI+G:PSYE+SCI+FBA+MTV+PS+GS+SM+FB+RIF+CONTRACTING+SC+PRP+SS+PI+MODELING**									
Rhee_2022	79	−0.07	0.1700	80	−0.08	0.1700	0.01 [−0.05; 0.06]	100.0%	100.0%
**PSYE+SCI+GS+RIF+IT+SS+SG+PI+G:PSYE+SCI+MTV+GS+SM+RMD+FB+RIF+IT+SS+SG+PI+G+RD**									
de Niet, J._2012	68	−0.20	0.3772	73	−0.25	0.3903	0.05 [−0.08; 0.18]	100.0%	100.0%
**PSYE+SCI+GS+SM+DM+PI:ME**									
Ball_2011	15	−0.09	0.3580	14	0.02	0.3046	−0.11 [−0.35; 0.13]	100.0%	100.0%
**PSYE+SCI+GS+SM+DM+PI:PSYE+SCI+MTV+GS+SM+DM+PI**									
Ball_2011	15	−0.09	0.3580	17	−0.12	0.5043	0.03 [−0.27; 0.33]	100.0%	100.0%

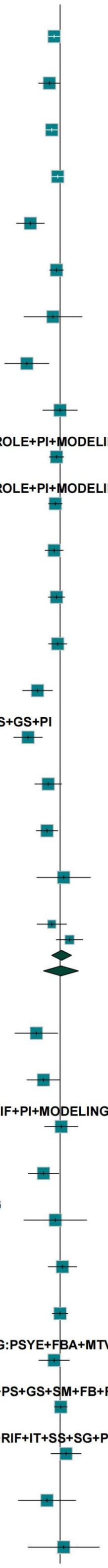

**PSYE+SCI+GS+TASK+SM+DM+FB+RIF+PI+G:ME**									
Arlinghaus_2021_2	27	−0.03	0.1000	32	−0.11	0.1700	0.08 [0.01; 0.15]	38.7%	48.6%
Arlinghaus_2021_1	58	−0.07	0.1500	54	−0.02	0.1500	−0.05 [−0.11; 0.01]	61.3%	51.4%
**Common effect model**	**85**			**86**			**0.00 [−0.04; 0.04]**	**100.0%**	**.**
**Random effects model**							**0.01 [−0.11; 0.14]**	**.**	**100.0%**
Heterogeneity: I^2 = 87.7%, τ^2 = 0.0074, p = 0.0043									
**PSYE+SCI+MTV+GS+FB+SG+PI+STRESS+G:ME**									
Hidayanty_2016	84	−0.08	0.2300	88	−0.02	0.1700	−0.06 [−0.12; 0.00]	100.0%	100.0%
**PSYE+SCI+MTV+GS+SG+PI+STRESS+MODELING+G:PSYE+SCI+MTV+PS+GS+RMD+RIF+SG+PI+STRESS+MODELING+G+RD**									
Nguyen_2012	78	−0.09	0.2580	73	−0.20	0.3376	0.11 [0.01; 0.21]	100.0%	100.0%
**PSYE+SCI+MTV+GS+SM+DM+PI:ME**									
Ball_2011	17	−0.12	0.5043	14	0.02	0.3046	−0.14 [−0.43; 0.15]	100.0%	100.0%
**PSYE+SCI+MTV+GS+TASK+PRP+SS+PI+G:PSYE+PI+G**									
Hystad, H. T._2013	46	−0.22	0.3812	50	−0.19	0.2926	−0.03 [−0.17; 0.11]	100.0%	100.0%
**PSYE+SCI+MTV+PI+G:PSYE+PI+G**									
Bean, M. K._2018	58	−0.03	0.2341	41	−0.04	0.2035	0.01 [−0.08; 0.10]	100.0%	100.0%
**PSYE+SCI+MTV+PS+GS+SM+FB+SG+PI+STRESS+MODELING+G:ME**									
Wylie−Rosett−2018	178	−0.15	0.4000	182	−0.12	0.4000	−0.03 [−0.11; 0.05]	100.0%	100.0%
**PSYE+SCI+MTV+PS+GS+TASK+SM+FB+RIF+PRP+SS+PI+STRESS+G:ME**									
Kitzman−Ulrich_2009	15	0.00	0.1000	11	0.00	0.1000	0.00 [−0.08; 0.08]	100.0%	100.0%
**PSYE+SCI+MTV+PS+GS+TASK+SM+FB+RIF+PRP+SS+PI+STRESS+G:PSYE+SCI+MTV+PS+GS+TASK+SM+RIF+PRP+SS+SG+PI+STRESS+G**									
Kitzman−Ulrich_2009	15	0.00	0.1000	16	−0.10	0.1000	0.10 [0.03; 0.17]	100.0%	100.0%
**PSYE+SCI+MTV+PS+GS+TASK+SM+RIF+PRP+SS+SG+PI+STRESS+G:ME**									
Kitzman−Ulrich_2009	16	−0.10	0.1000	11	0.00	0.1000	−0.10 [−0.18; −0.02]	100.0%	100.0%
**PSYE+SCI+MTV+SC+IT+SS+PI+G:ME**									
Alustiza_2021	47	0.30	0.8888	45	0.70	0.8000	−0.40 [−0.75; −0.05]	100.0%	100.0%
**PSYE+SCI+MTV+SC+PRP+PI:PSYE+SCI+MTV+SC+PRP+PI+G**									
Garipagaoglu_2009	40	−0.11	0.3501	40	−0.10	0.3501	−0.01 [−0.16; 0.14]	100.0%	100.0%
**PSYE+SCI+PS+GS+DM+SC+ROLE+PI:PSYE+SCI+PS+GS+DM+SC+ROLE+PI+RD**									
Varagiannis−2021	30	−0.28	0.4637	25	−0.24	0.5385	−0.04 [−0.31; 0.23]	100.0%	100.0%
**PSYE+SCI+PS+GS+DM+SC+ROLE+PI+G:PSYE+SCI+PS+GS+DM+SC+ROLE+PI**									
Varagiannis−2021	36	−0.13	0.4243	30	−0.28	0.4637	0.15 [−0.07; 0.37]	100.0%	100.0%
**PSYE+SCI+PS+GS+DM+SC+ROLE+PI+G:PSYE+SCI+PS+GS+DM+SC+ROLE+PI+RD**									
Varagiannis−2021	36	−0.13	0.4243	25	−0.24	0.5385	0.11 [−0.14; 0.36]	100.0%	100.0%
**PSYE+SCI+PS+GS+FB+RIF+PRP+PI+MODELING+RULE:ME**									
Gerards, S. M. P. L._2015	44	−0.06	0.1900	42	−0.06	0.1800	0.00 [−0.08; 0.08]	100.0%	100.0%
**PSYE+SCI+PS+GS+RIF+PRP+PI+MODELING+RULE:ME**									
Golley_2007	37	−0.13	0.3952	36	−0.05	0.2758	−0.08 [−0.24; 0.08]	100.0%	100.0%
**PSYE+SCI+PS+GS+RIF+PRP+SS+PI+MODELING+RULE:ME**									
Golley_2007	38	−0.22	0.3952	36	−0.05	0.2758	−0.17 [−0.32; −0.02]	100.0%	100.0%
**PSYE+SCI+PS+GS+RIF+PRP+SS+PI+MODELING+RULE:PSYE+SCI+PS+GS+RIF+PRP+PI+MODELING+RULE**									
Golley_2007	38	−0.22	0.3952	37	−0.13	0.3952	−0.09 [−0.27; 0.09]	100.0%	100.0%
**PSYE+SCI+PS+GS+RIF+PRP+SS+PI+MODELING+RULE:PSYE+SCI+SS+PI**									
Magarey_2011	85	−0.29	0.4663	84	−0.22	0.4796	−0.07 [−0.21; 0.07]	100.0%	100.0%
**PSYE+SCI+PS+GS+SM+DM+FB+SS+PI+RD:ME**									
Norman_2016_1	29	−0.10	0.2738	25	0.00	0.2687	−0.10 [−0.24; 0.04]	41.2%	41.8%
Norman_2016_2	24	0.00	0.2463	28	0.00	0.1909	0.00 [−0.12; 0.12]	58.8%	58.2%
**Common effect model**	**53**			**53**			**−0.04 [−0.13; 0.05]**	**100.0%**	**.**
**Random effects model**							**−0.04 [−0.14; 0.05]**	**.**	**100.0%**
Heterogeneity: I^2 = 7%, τ^2 = 0.0003, p = 0.2998									
**PSYE+SCI+PS+GS+SM+FB+SC+PI:ME**									
Ahmad_2018	67	−0.02	0.2764	67	−0.01	0.2666	−0.01 [−0.10; 0.08]	100.0%	100.0%
**PSYE+SM+DM+RIF+SC+SG+PI+MODELING:ME**									
Maddison_2014	127	0.01	0.5896	124	0.04	0.6612	−0.03 [−0.19; 0.13]	100.0%	100.0%
**PSYE+SM+FB+PI+RD:ME**									
Looney_2014	7	−0.08	0.2249	8	−0.07	0.4389	−0.01 [−0.36; 0.34]	100.0%	100.0%
**PSYE+SM+FB+PI+RD:PSYE+GS+TASK+SM+FB+RIF+SC+PI+MODELING+RD**									
Looney_2014	7	−0.08	0.2249	7	−0.16	0.3676	0.08 [−0.24; 0.40]	100.0%	100.0%
**PSYE+SM+SC+PRP+PI+G:PSYE+SM+SC+PRP+G**									
Marques_2023	27	−0.05	0.2600	29	−0.07	0.2000	0.02 [−0.10; 0.14]	100.0%	100.0%
**PSYE+TASK+SM+DM+FB+RD:PSYE+SM+RD**									
Vidmar−2021	16	−0.11	0.1900	15	−0.05	0.0900	−0.06 [−0.16; 0.04]	100.0%	100.0%
**PSYE+TASK+SM+FB+RIF+RT+RD:PSYE+MTV+GS+TASK+SM+CONTRACTING+SC**									
Stasinaki−2021	18	−0.06	0.1900	13	−0.55	0.5100	0.48 [0.19; 0.78]	100.0%	100.0%
**PSYE+TASK+SM+RD:PSYE+SM+RD**									
Vidmar−2021	19	−0.09	0.1400	15	−0.05	0.0900	−0.04 [−0.12; 0.04]	100.0%	100.0%
**PSYE+TASK+SM+RD:PSYE+TASK+SM+DM+FB+RD**									
Vidmar−2021	19	−0.09	0.1400	16	−0.11	0.1900	0.02 [−0.09; 0.13]	100.0%	100.0%
**PSYE+TWC+FBA+PS+RIF+SC+PRP+SS+PI+STRESS+RULE:ME**									
Anna Ek_2019	44	−0.54	0.8000	87	−0.04	0.6400	−0.50 [−0.77; −0.23]	100.0%	100.0%

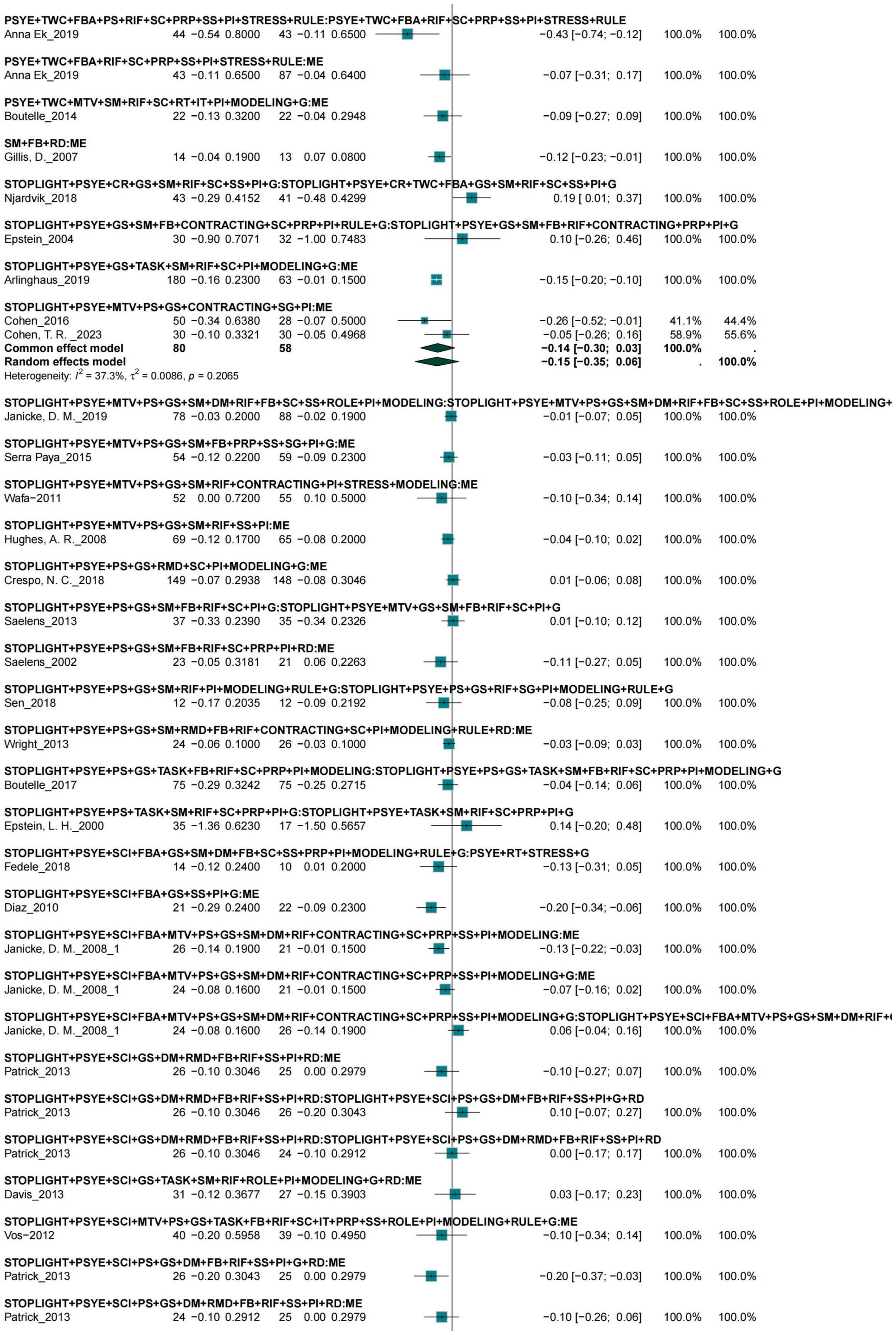

PSYE+TWC+FBA+PS+RIF+SC+PRP+SS+PI+STRESS+RULE:PSYE+TWC+FBA+RIF+SC+PRP+SS+PI+STRESS+RULE
Anna Ek_2019 44 −0.54 0.8000 43 −0.11 0.6500 −0.43 [−0.74; −0.12] 100.0% 100.0%
PSYE+TWC+FBA+RIF+SC+PRP+SS+PI+STRESS+RULE:ME
Anna Ek_2019 43 −0.11 0.6500 87 −0.04 0.6400 −0.07 [−0.31; 0.17] 100.0% 100.0%
PSYE+TWC+MTV+SM+RIF+SC+RT+IT+PI+MODELING+G:ME
Boutelle_2014 22 −0.13 0.3200 22 −0.04 0.2948 −0.09 [−0.27; 0.09] 100.0% 100.0%
SM+FB+RD:ME
Gillis, D._2007 14 −0.04 0.1900 13 0.07 0.0800 −0.12 [−0.23; −0.01] 100.0% 100.0%
STOPLIGHT+PSYE+CR+GS+SM+RIF+SC+SS+PI+G:STOPLIGHT+PSYE+CR+TWC+FBA+GS+SM+RIF+SC+SS+PI+G
Njardvik_2018 43 −0.29 0.4152 41 −0.48 0.4299 0.19 [0.01; 0.37] 100.0% 100.0%
STOPLIGHT+PSYE+GS+SM+FB+CONTRACTING+SC+PRP+PI+RULE+G:STOPLIGHT+PSYE+GS+SM+FB+RIF+CONTRACTING+PRP+PI+G
Epstein_2004 30 −0.90 0.7071 32 −1.00 0.7483 0.10 [−0.26; 0.46] 100.0% 100.0%
STOPLIGHT+PSYE+GS+TASK+SM+RIF+SC+PI+MODELING+G:ME
Arlinghaus_2019 180 −0.16 0.2300 63 −0.01 0.1500 −0.15 [−0.20; −0.10] 100.0% 100.0%
STOPLIGHT+PSYE+MTV+PS+GS+CONTRACTING+SG+PI:ME
Cohen_2016 50 −0.34 0.6380 28 −0.07 0.5000 −0.26 [−0.52; −0.01] 41.1% 44.4%
Cohen, T. R. _2023 30 −0.10 0.3321 30 −0.05 0.4968 −0.05 [−0.26; 0.16] 58.9% 55.6%
Common effect model 80 58 −0.14 [−0.30; 0.03] 100.0% .
Random effects model −0.15 [−0.35; 0.06] . 100.0%
Heterogeneity: I^2 = 37.3%, τ^2 = 0.0086, p = 0.2065
STOPLIGHT+PSYE+MTV+PS+GS+SM+DM+RIF+FB+SC+SS+ROLE+PI+MODELING:STOPLIGHT+PSYE+MTV+PS+GS+SM+DM+RIF+FB+SC+SS+ROLE+PI+MODELING+
Janicke, D. M._2019 78 −0.03 0.2000 88 −0.02 0.1900 −0.01 [−0.07; 0.05] 100.0% 100.0%
STOPLIGHT+PSYE+MTV+PS+GS+SM+FB+PRP+SS+SG+PI+G:ME
Serra Paya_2015 54 −0.12 0.2200 59 −0.09 0.2300 −0.03 [−0.11; 0.05] 100.0% 100.0%
STOPLIGHT+PSYE+MTV+PS+GS+SM+RIF+CONTRACTING+PI+STRESS+MODELING:ME
Wafa−2011 52 0.00 0.7200 55 0.10 0.5000 −0.10 [−0.34; 0.14] 100.0% 100.0%
STOPLIGHT+PSYE+MTV+PS+GS+SM+RIF+SS+PI:ME
Hughes, A. R._2008 69 −0.12 0.1700 65 −0.08 0.2000 −0.04 [−0.10; 0.02] 100.0% 100.0%
STOPLIGHT+PSYE+PS+GS+RMD+SC+PI+MODELING+G:ME
Crespo, N. C._2018 149 −0.07 0.2938 148 −0.08 0.3046 0.01 [−0.06; 0.08] 100.0% 100.0%
STOPLIGHT+PSYE+PS+GS+SM+FB+RIF+SC+PI+G:STOPLIGHT+PSYE+MTV+GS+SM+FB+RIF+SC+PI+G
Saelens_2013 37 −0.33 0.2390 35 −0.34 0.2326 0.01 [−0.10; 0.12] 100.0% 100.0%
STOPLIGHT+PSYE+PS+GS+SM+FB+RIF+SC+PRP+PI+RD:ME
Saelens_2002 23 −0.05 0.3181 21 0.06 0.2263 −0.11 [−0.27; 0.05] 100.0% 100.0%
STOPLIGHT+PSYE+PS+GS+SM+RIF+PI+MODELING+RULE+G:STOPLIGHT+PSYE+PS+GS+RIF+SG+PI+MODELING+RULE+G
Sen_2018 12 −0.17 0.2035 12 −0.09 0.2192 −0.08 [−0.25; 0.09] 100.0% 100.0%
STOPLIGHT+PSYE+PS+GS+SM+RMD+FB+RIF+CONTRACTING+SC+PI+MODELING+RULE+RD:ME
Wright_2013 24 −0.06 0.1000 26 −0.03 0.1000 −0.03 [−0.09; 0.03] 100.0% 100.0%
STOPLIGHT+PSYE+PS+GS+TASK+FB+RIF+SC+PRP+PI+MODELING:STOPLIGHT+PSYE+PS+GS+TASK+SM+FB+RIF+SC+PRP+PI+MODELING+G
Boutelle_2017 75 −0.29 0.3242 75 −0.25 0.2715 −0.04 [−0.14; 0.06] 100.0% 100.0%
STOPLIGHT+PSYE+PS+TASK+SM+RIF+SC+PRP+PI+G:STOPLIGHT+PSYE+TASK+SM+RIF+SC+PRP+PI+G
Epstein, L. H._2000 35 −1.36 0.6230 17 −1.50 0.5657 0.14 [−0.20; 0.48] 100.0% 100.0%
STOPLIGHT+PSYE+SCI+FBA+GS+SM+DM+FB+SC+SS+PRP+PI+MODELING+RULE+G:PSYE+RT+STRESS+G
Fedele_2018 14 −0.12 0.2400 10 0.01 0.2000 −0.13 [−0.31; 0.05] 100.0% 100.0%
STOPLIGHT+PSYE+SCI+FBA+GS+SS+PI+G:ME
Diaz_2010 21 −0.29 0.2400 22 −0.09 0.2300 −0.20 [−0.34; −0.06] 100.0% 100.0%
STOPLIGHT+PSYE+SCI+FBA+MTV+PS+GS+SM+DM+RIF+CONTRACTING+SC+PRP+SS+PI+MODELING:ME
Janicke, D. M._2008_1 26 −0.14 0.1900 21 −0.01 0.1500 −0.13 [−0.22; −0.03] 100.0% 100.0%
STOPLIGHT+PSYE+SCI+FBA+MTV+PS+GS+SM+DM+RIF+CONTRACTING+SC+PRP+SS+PI+MODELING+G:ME
Janicke, D. M._2008_1 24 −0.08 0.1600 21 −0.01 0.1500 −0.07 [−0.16; 0.02] 100.0% 100.0%
STOPLIGHT+PSYE+SCI+FBA+MTV+PS+GS+SM+DM+RIF+CONTRACTING+SC+PRP+SS+PI+MODELING+G:STOPLIGHT+PSYE+SCI+FBA+MTV+PS+GS+SM+DM+RIF+
Janicke, D. M._2008_1 24 −0.08 0.1600 26 −0.14 0.1900 0.06 [−0.04; 0.16] 100.0% 100.0%
STOPLIGHT+PSYE+SCI+GS+DM+RMD+FB+RIF+SS+PI+RD:ME
Patrick_2013 26 −0.10 0.3046 25 0.00 0.2979 −0.10 [−0.27; 0.07] 100.0% 100.0%
STOPLIGHT+PSYE+SCI+GS+DM+RMD+FB+RIF+SS+PI+RD:STOPLIGHT+PSYE+SCI+PS+GS+DM+FB+RIF+SS+PI+G+RD
Patrick_2013 26 −0.10 0.3046 26 −0.20 0.3043 0.10 [−0.07; 0.27] 100.0% 100.0%
STOPLIGHT+PSYE+SCI+GS+DM+RMD+FB+RIF+SS+PI+RD:STOPLIGHT+PSYE+SCI+PS+GS+DM+RMD+FB+RIF+SS+PI+RD
Patrick_2013 26 −0.10 0.3046 24 −0.10 0.2912 0.00 [−0.17; 0.17] 100.0% 100.0%
STOPLIGHT+PSYE+SCI+GS+TASK+SM+RIF+ROLE+PI+MODELING+G+RD:ME
Davis_2013 31 −0.12 0.3677 27 −0.15 0.3903 0.03 [−0.17; 0.23] 100.0% 100.0%
STOPLIGHT+PSYE+SCI+MTV+PS+GS+TASK+FB+RIF+SC+IT+PRP+SS+ROLE+PI+MODELING+RULE+G:ME
Vos−2012 40 −0.20 0.5958 39 −0.10 0.4950 −0.10 [−0.34; 0.14] 100.0% 100.0%
STOPLIGHT+PSYE+SCI+PS+GS+DM+FB+RIF+SS+PI+G+RD:ME
Patrick_2013 26 −0.20 0.3043 25 0.00 0.2979 −0.20 [−0.37; −0.03] 100.0% 100.0%
STOPLIGHT+PSYE+SCI+PS+GS+DM+RMD+FB+RIF+SS+PI+RD:ME
Patrick_2013 24 −0.10 0.2912 25 0.00 0.2979 −0.10 [−0.26; 0.06] 100.0% 100.0%

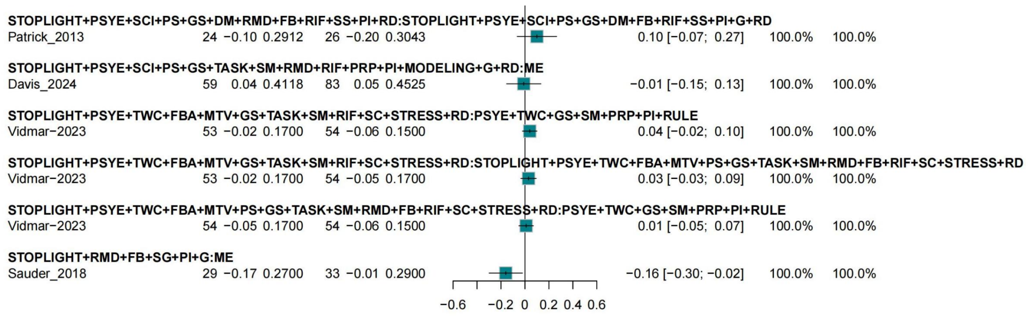

Notes: ME, minimal education; PSYE, psychoeducation; CR, cognitive restructuring; TWC, third-wave components; SCI, self-concept improvement; FBA, functional behavioural analysis; MTV, motivation; GS, goal-setting; TASK, task-setting; RULE, rule-setting; SM, self-monitoring; DM, device-monitoring; RMD, reminders; FB, feedback; PS, problem solving; PRP, preplanning; RIF, reinforcement; CONTRACTING, contracting; MODELING, modelling; SC, stimulus control; RT, relaxation training; IT, inhibition training; SS, social support; STRESS, stress management; STOPLIGHT, stoplight approach; ROLE, role playing; G, group; RD, remote; SG, serious games; PI, parental involvement

4.6.2 Body fat

Study	Experimental Total	Experimental Mean	Experimental SD	Control Total	Control Mean	Control SD	MD [95%−CI]	Weight (common)	Weight (random)
**PSYE+CR+FBA+MTV+GS+TASK+SM+SC+PI+G:ME**									
Miri_2019	55	−1.88	3.4697	55	1.56	3.1766	−3.44 [−4.68; −2.20]	100.0%	100.0%
**PSYE+CR+MTV+PS+GS+SM+DM+SC+PRP+SS+SG+G:ME**									
Pbert_2016	54	−1.01	4.5166	57	0.42	4.1840	−1.43 [−3.05; 0.19]	100.0%	100.0%
**PSYE+CR+MTV+PS+TASK+FB+SC+PI+G:ME**									
Robinson_2021	120	0.13	1.4800	121	0.30	1.4600	−0.17 [−0.54; 0.20]	100.0%	100.0%
**PSYE+CR+RT+G:PSYE+SCI+FBA+G**									
Weigensberg−2014	18	0.78	4.0000	15	0.39	5.3000	0.39 [−2.87; 3.65]	100.0%	100.0%
**PSYE+CR+SCI+MTV+PS+GS+SM+DM+SC+PRP+PI+G+RD:ME**									
Tsiros−2008	25	−3.50	4.0559	22	−1.20	8.5348	−2.30 [−6.20; 1.60]	100.0%	100.0%
**PSYE+DM+FB+PI+RD:ME**									
Ford, A. L._2009	54	−4.60	4.5200	52	−1.30	4.3300	−3.30 [−4.98; −1.62]	100.0%	100.0%
**PSYE+FBA+MTV+GS+DM+FB+RIF+SC+PI+MODELING+G:PSYE+DM+G**									
Bocca, G._2014	40	−1.50	3.4000	35	−0.30	4.0000	−1.20 [−2.89; 0.49]	100.0%	100.0%
**PSYE+FBA+MTV+PI:ME**									
Christie_2017	87	0.71	2.8000	87	1.25	2.8000	−0.54 [−1.37; 0.29]	100.0%	100.0%
**PSYE+G:PSYE+MTV+RMD+PI+G+RD**									
Resnicow_2005	70	0.70	2.7659	53	−0.40	2.6012	1.10 [0.15; 2.05]	100.0%	100.0%
**PSYE+GS+SM+RIF+SC+PRP+SS+PI+MODELING+G:ME**									
Savoye_2011	105	−3.90	6.0100	69	2.10	6.3600	−6.00 [−7.89; −4.11]	100.0%	100.0%
**PSYE+GS+SM+RIF+SC+SG+PI+MODELING+G:ME**									
Johnston_2010	40	−2.74	9.5600	20	−0.96	3.7100	−1.78 [−5.16; 1.60]	14.1%	14.1%
Johnston_2013	46	−2.34	3.1800	25	−0.40	2.5900	−1.94 [−3.31; −0.57]	85.9%	85.9%
**Common effect model**	**86**			**45**			**−1.92 [−3.19; −0.65]**	**100.0%**	.
**Random effects model**							**−1.92 [−3.19; −0.65]**	.	**100.0%**
Heterogeneity: I^2 = 0%, τ^2 = 0, p = 0.9315									
**PSYE+GS+SM+SC+PI+STRESS+G+RD:ME**									
Moore_2019	123	0.30	1.8890	119	−0.04	2.1700	0.34 [−0.17; 0.86]	100.0%	100.0%
**PSYE+GS+TASK+SS+PI+G+RD:ME**									
Waling_2012	48	−0.21	2.2600	45	−0.16	3.3400	−0.05 [−1.22; 1.12]	100.0%	100.0%
**PSYE+MTV:PSYE**									
Freira_2018	46	−2.80	3.4620	51	0.69	3.9059	−3.49 [−4.96; −2.02]	100.0%	100.0%
**PSYE+MTV+G:ME**									
Pakpour_2015	119	−1.12	3.4610	119	0.03	3.6224	−1.15 [−2.05; −0.25]	100.0%	100.0%
**PSYE+MTV+G:PSYE+MTV+PI+G**									
Pakpour_2015	119	−1.12	3.4610	119	−1.74	2.8376	0.62 [−0.18; 1.42]	100.0%	100.0%
**PSYE+MTV+PI+G:ME**									
Pakpour_2015	119	−1.74	2.8376	119	0.03	3.6224	−1.77 [−2.60; −0.94]	100.0%	100.0%
**PSYE+MTV+PS+GS+FB+PI+RULE+RD:ME**									
Smith−2021	141	1.04	3.9405	99	0.24	3.4877	0.80 [−0.15; 1.75]	100.0%	100.0%
**PSYE+MTV+TASK+SC+PRP+SG+PI+G:ME**									
Nemet_2005	24	−3.30	3.4886	22	1.50	4.4319	−4.80 [−7.12; −2.48]	100.0%	100.0%
**PSYE+PI:ME**									
Abraham_2015	16	−1.83	4.7158	16	0.96	4.9454	−2.79 [−6.14; 0.56]	100.0%	100.0%
**PSYE+PI+G:PSYE+SG+PI+G**									
Sanchez−Lopez A M_2020	49	2.57	2.5580	49	−2.84	2.9836	5.41 [4.31; 6.51]	100.0%	100.0%
**PSYE+PS+GS+FB+PI:ME**									
Wake−−2013	62	0.30	3.1613	56	1.60	2.7521	−1.30 [−2.37; −0.23]	100.0%	100.0%
**PSYE+PS+GS+FB+RIF+PI+RULE+RD:ME**									
Taylor−2015	104	0.00	2.4417	102	0.50	2.0125	−0.50 [−1.11; 0.11]	100.0%	100.0%
**PSYE+PS+GS+RIF+FB+PRP+SS+ROLE+STRESS+G+RD:PSYE+PRP+G**									
Grey, M._2009	112	−1.70	7.0200	86	−2.40	6.6200	0.70 [−1.21; 2.61]	100.0%	100.0%
**PSYE+PS+GS+SM+FB+CONTRACTING+G+RD:PSYE**									
Williamson−2005	28	−1.12	0.4700	29	0.43	0.4700	−1.55 [−1.79; −1.31]	100.0%	100.0%
**PSYE+RMD+PRP+STRESS+RD:ME**									
Abraham_2015	16	−0.97	5.8106	16	0.96	4.9454	−1.93 [−5.67; 1.81]	100.0%	100.0%
**PSYE+RMD+PRP+STRESS+RD:PSYE+PI**									
Abraham_2015	16	−0.97	5.8106	16	−1.83	4.7158	0.86 [−2.81; 4.53]	100.0%	100.0%

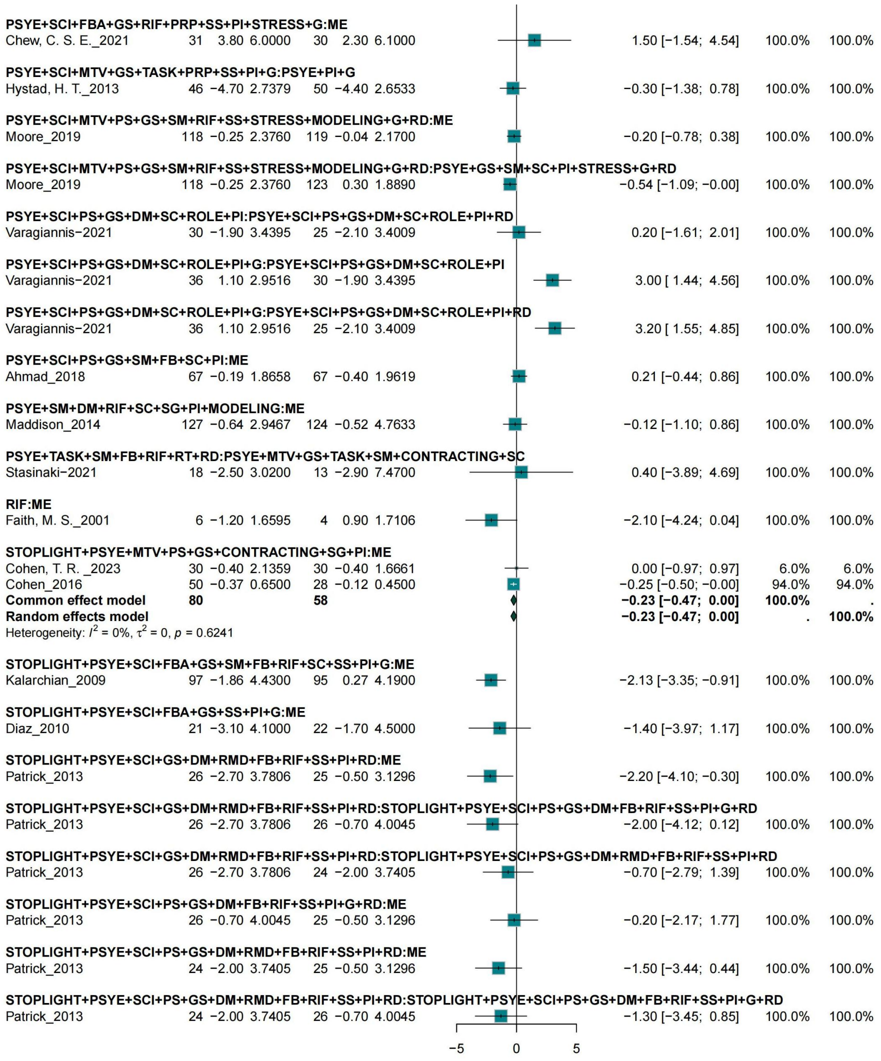

Notes: ME, minimal education; PSYE, psychoeducation; CR, cognitive restructuring; TWC, third-wave components; SCI, self-concept improvement; FBA, functional behavioural analysis; MTV, motivation; GS, goal-setting; TASK, task-setting; RULE, rule-setting; SM, self-monitoring; DM, device-monitoring; RMD, reminders; FB, feedback; PS, problem solving; PRP, preplanning; RIF, reinforcement; CONTRACTING, contracting; MODELING, modelling; SC, stimulus control; RT, relaxation training; IT, inhibition training; SS, social support; STRESS, stress management; STOPLIGHT, stoplight approach; ROLE, role playing; G, group; RD, remote; SG, serious games; PI, parental involvement

4.6.3 Waist circumference

Study	Experimental Total	Experimental Mean	Experimental SD	Control Total	Control Mean	Control SD	MD [95%−CI]	Weight (common)	Weight (random)
**PSYE+CR+FBA+MTV+GS+TASK+SM+SC+PI+G:ME**									
Miri_2019	55	−2.99	4.0804	55	1.64	4.0699	−4.63 [−6.15; −3.11]	100.0%	100.0%
**PSYE+CR+MTV+PS+GS+SM+DM+SC+PRP+SS+SG+G:ME**									
Pbert_2016	54	0.27	6.6121	57	−2.15	6.6770	2.42 [−0.05; 4.89]	100.0%	100.0%
**PSYE+CR+MTV+PS+TASK+FB+SC+PI+G:ME**									
Robinson_2021	120	3.42	2.4600	121	3.72	2.3700	−0.30 [−0.91; 0.31]	100.0%	100.0%
**PSYE+CR+PS+GS+SM+FB+RIF+SC+SS+PI+STRESS+G:PSYE+CR+PS+GS+SM+RIF+SC+PI+STRESS**									
Berkowitz, R. I. _2013	81	−2.87	9.0900	88	−3.41	9.8500	0.54 [−2.32; 3.40]	100.0%	100.0%
**PSYE+CR+RT+G:PSYE+SCI+FBA+G**									
Weigensberg−2014	18	0.64	1.6000	15	−0.77	3.2000	1.41 [−0.37; 3.19]	100.0%	100.0%
**PSYE+CR+SCI+MTV+PS+GS+DM+SS+SG+PI+G:ME**									
Berry_2014	184	8.83	11.7385	162	5.78	11.7372	3.05 [0.57; 5.53]	100.0%	100.0%
**PSYE+CR+SCI+MTV+PS+GS+SM+DM+SC+PRP+PI+G+RD:ME**									
Tsiros−2008	25	−5.90	9.2412	22	−0.30	15.8690	−5.60 [−13.16; 1.96]	100.0%	100.0%
**PSYE+FBA+GS+TASK+PRP+SS+SG+PI+STRESS+MODELING+G:ME**									
Kalavainen_2007	35	−0.70	3.1700	35	0.80	3.6200	−1.50 [−3.09; 0.09]	100.0%	100.0%
**PSYE+FBA+MTV+GS+DM+FB+RIF+SC+PI+MODELING+G:PSYE+DM+G**									
Bocca, G._2014	40	−0.90	3.2000	35	0.90	5.2000	−1.80 [−3.79; 0.19]	100.0%	100.0%
**PSYE+FBA+MTV+PI:ME**									
Christie_2017	87	−0.11	3.7300	87	0.16	3.7300	−0.27 [−1.38; 0.84]	100.0%	100.0%
**PSYE+G:PSYE+MTV+RMD+PI+G+RD**									
Resnicow_2005	70	1.90	6.8084	53	−0.10	5.1736	2.00 [−0.12; 4.12]	100.0%	100.0%
**PSYE+GS+SM+SC+PI+STRESS+G+RD:ME**									
Moore_2019	123	2.34	2.8390	119	2.20	3.2880	0.14 [−0.64; 0.91]	100.0%	100.0%
**PSYE+GS+TASK+SS+PI+G+RD:ME**									
Waling_2012	48	1.29	3.9700	45	3.49	6.4400	−2.20 [−4.39; −0.01]	100.0%	100.0%
**PSYE+MTV:ME**									
Walpole−2013	20	1.55	3.3332	20	−2.10	4.9794	3.65 [1.02; 6.28]	100.0%	100.0%
**PSYE+MTV:PSYE**									
Freira_2018	46	−3.55	4.8785	51	0.63	5.6024	−4.18 [−6.27; −2.09]	100.0%	100.0%
**PSYE+MTV+G:ME**									
Pakpour_2015	119	−0.62	4.1129	119	4.39	3.8334	−5.01 [−6.02; −4.00]	100.0%	100.0%
**PSYE+MTV+G:PSYE+MTV+PI+G**									
Pakpour_2015	119	−0.62	4.1129	119	−1.78	4.0158	1.16 [0.13; 2.19]	100.0%	100.0%
**PSYE+MTV+GS+TASK+DM+FB+SC+PI:ME**									
Luque_2024	106	0.67	5.2600	95	2.10	4.2900	−1.43 [−2.75; −0.11]	100.0%	100.0%
**PSYE+MTV+PI+G:ME**									
Pakpour_2015	119	−1.78	4.0158	119	4.39	3.8334	−6.17 [−7.17; −5.17]	100.0%	100.0%
**PSYE+MTV+PS+GS+FB+PI:ME**									
Small−2014	33	−1.17	4.6184	27	3.07	4.5505	−4.24 [−6.57; −1.91]	100.0%	100.0%
**PSYE+MTV+TASK+SC+SG+PI+G:ME**									
Patsopoulou_2017	60	−2.40	1.5800	61	1.50	1.5900	−3.90 [−4.46; −3.34]	100.0%	100.0%
**PSYE+PI:ME**									
Abraham_2015	16	−1.97	4.9094	16	−1.60	5.1082	−0.37 [−3.84; 3.10]	100.0%	100.0%
**PSYE+PS+GS+FB+RIF+PI+RULE+RD:ME**									
Taylor−2015	104	1.40	4.6658	102	2.90	3.6791	−1.50 [−2.65; −0.35]	100.0%	100.0%
**PSYE+PS+GS+RIF+FB+PRP+SS+ROLE+STRESS+G+RD:PSYE+PRP+G**									
Grey, M._2009	112	−3.80	10.8000	86	−3.20	9.9400	−0.60 [−3.50; 2.30]	100.0%	100.0%
**PSYE+PS+GS+TASK+SM+DM+PRP+SS+PI+G:ME**									
Hofsteenge, G. H._2014	71	0.30	5.6886	51	3.30	5.7645	−3.00 [−5.06; −0.94]	100.0%	100.0%
**PSYE+RMD+PRP+STRESS+RD:ME**									
Abraham_2015	16	−2.70	6.3331	16	−1.60	5.1082	−1.10 [−5.09; 2.89]	100.0%	100.0%
**PSYE+RMD+PRP+STRESS+RD:PSYE+PI**									
Abraham_2015	16	−2.70	6.3331	16	−1.97	4.9094	−0.73 [−4.66; 3.20]	100.0%	100.0%
**PSYE+SCI+CONTRACTING+SS+PI+G:ME**									
Anderson_2017	69	2.29	5.5900	69	2.86	5.6600	−0.57 [−2.45; 1.31]	100.0%	100.0%
**PSYE+SCI+FBA+GS+RIF+PRP+SS+PI+STRESS+G:ME**									
Chew, C. S. E._2021	31	−1.50	4.7000	30	0.10	4.6000	−1.60 [−3.93; 0.73]	100.0%	100.0%
**PSYE+SCI+FBA+MTV+PS+GS+SM+RIF+CONTRACTING+SC+SS+SG+PI+G:PSYE+FBA+MTV+GS+SM+RIF+CONTRACTING+SC+PI+G**									
Jelalian_2011	45	−2.95	4.6294	44	−2.88	4.5175	−0.07 [−1.97; 1.83]	100.0%	100.0%
**PSYE+SCI+GS+SM+DM+PI:ME**									
Ball_2011	15	−2.30	8.4926	14	0.80	6.9935	−3.10 [−8.75; 2.55]	100.0%	100.0%
**PSYE+SCI+GS+SM+DM+PI:PSYE+SCI+MTV+GS+SM+DM+PI**									
Ball_2011	15	−2.30	8.4926	17	−0.80	10.9062	−1.50 [−8.23; 5.23]	100.0%	100.0%

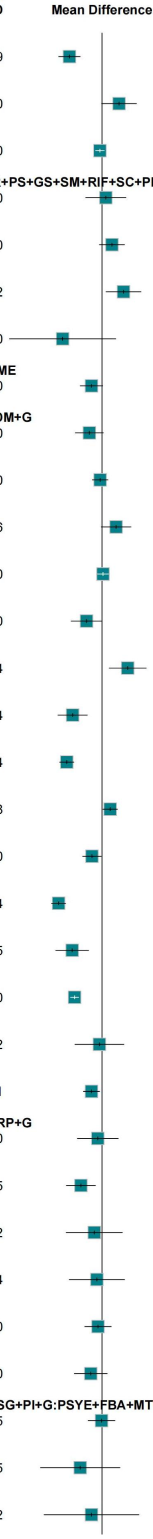

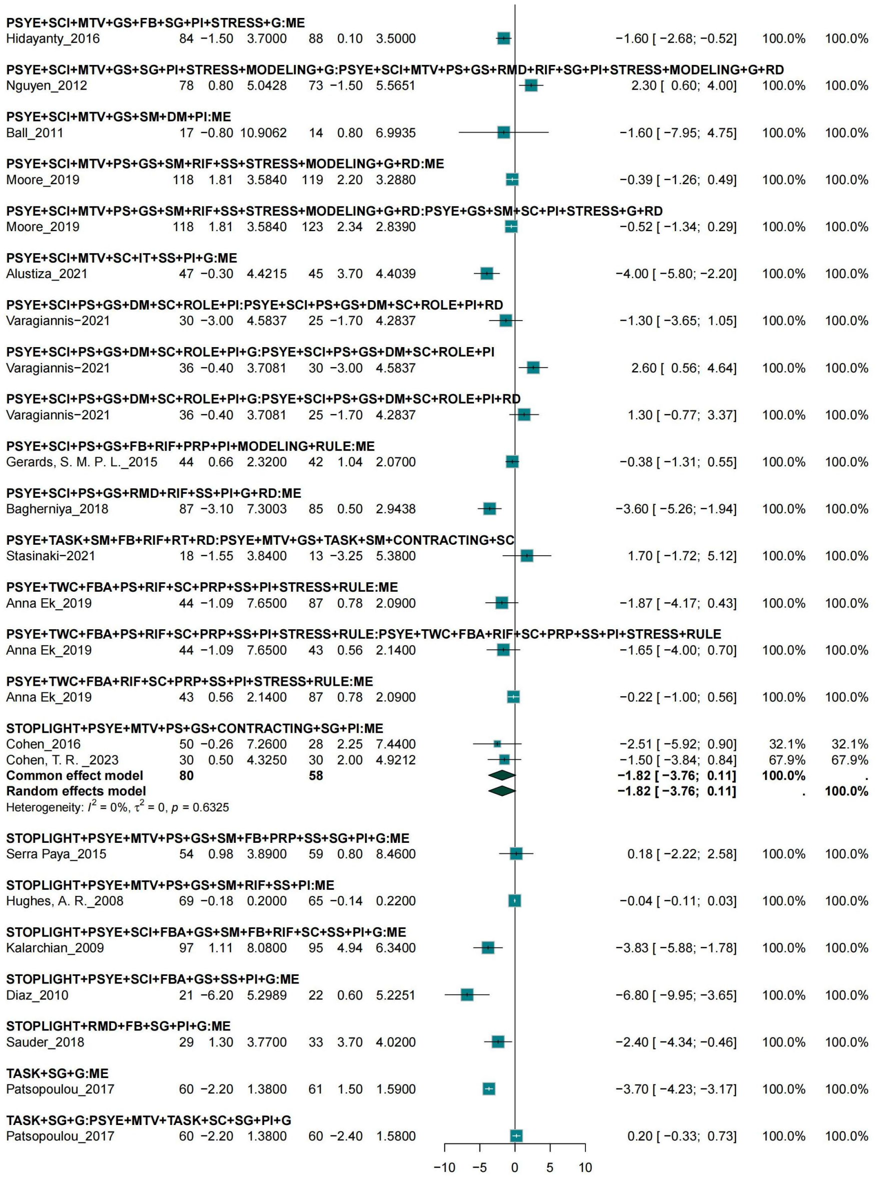

Notes: ME, minimal education; PSYE, psychoeducation; CR, cognitive restructuring; TWC, third-wave components; SCI, self-concept improvement; FBA, functional behavioural analysis; MTV, motivation; GS, goal-setting; TASK, task-setting; RULE, rule-setting; SM, self-monitoring; DM, device-monitoring; RMD, reminders; FB, feedback; PS, problem solving; PRP, preplanning; RIF, reinforcement; CONTRACTING, contracting; MODELING, modelling; SC, stimulus control; RT, relaxation training; IT, inhibition training; SS, social support; STRESS, stress management; STOPLIGHT, stoplight approach; ROLE, role playing; G, group; RD, remote; SG, serious games; PI, parental involvement

4.6.4 Height

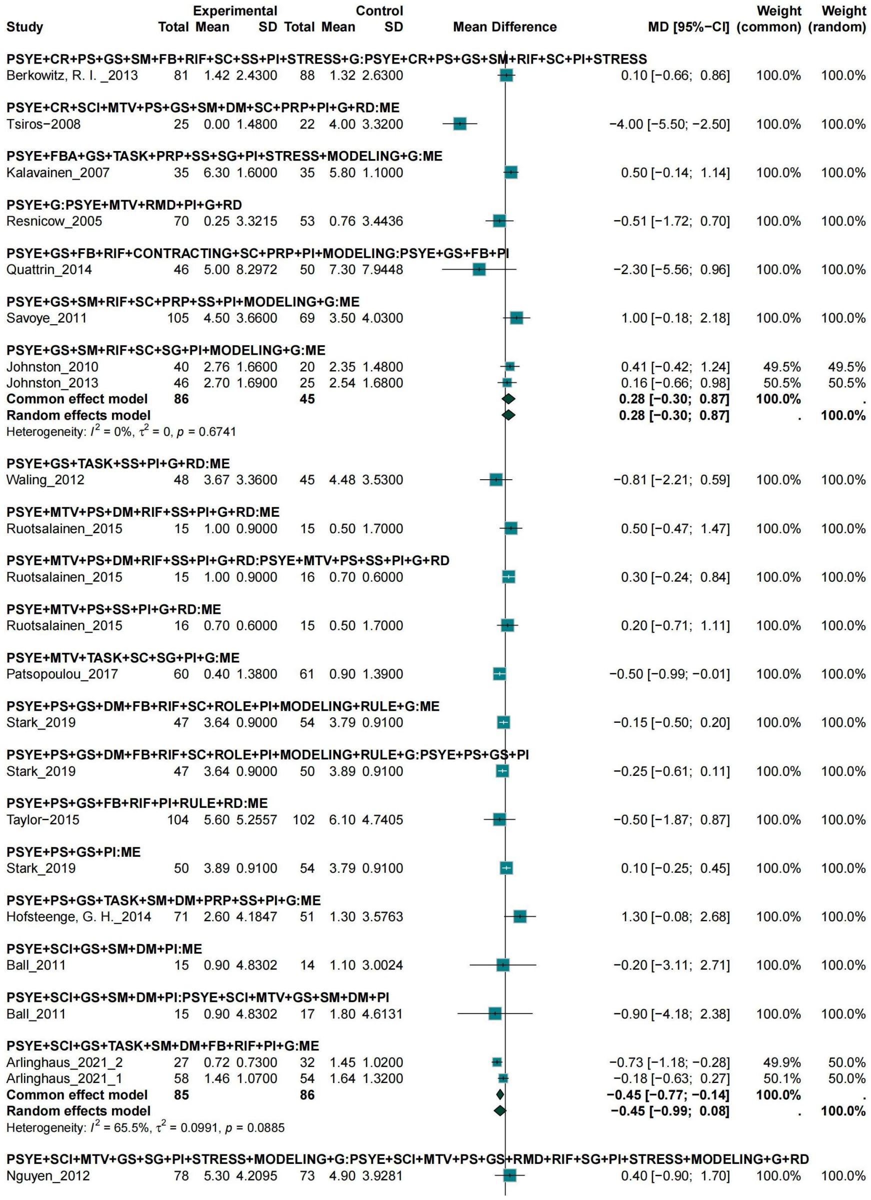
Experimental Control
Study Total Mean SD Total Mean SD Mean Difference MD [95%−CI] Weight (common) Weight (random)
PSYE+CR+PS+GS+SM+FB+RIF+SC+SS+PI+STRESS+G:PSYE+CR+PS+GS+SM+RIF+SC+PI+STRESS
Berkowitz, R. I. _2013 81 1.42 2.4300 88 1.32 2.6300 0.10 [−0.66; 0.86] 100.0% 100.0%
PSYE+CR+SCI+MTV+PS+GS+SM+DM+SC+PRP+PI+G+RD:ME
Tsiros−2008 25 0.00 1.4800 22 4.00 3.3200 −4.00 [−5.50; −2.50] 100.0% 100.0%
PSYE+FBA+GS+TASK+PRP+SS+SG+PI+STRESS+MODELING+G:ME
Kalavainen_2007 35 6.30 1.6000 35 5.80 1.1000 0.50 [−0.14; 1.14] 100.0% 100.0%
PSYE+G:PSYE+MTV+RMD+PI+G+RD
Resnicow_2005 70 0.25 3.3215 53 0.76 3.4436 −0.51 [−1.72; 0.70] 100.0% 100.0%
PSYE+GS+FB+RIF+CONTRACTING+SC+PRP+PI+MODELING:PSYE+GS+FB+PI
Quattrin_2014 46 5.00 8.2972 50 7.30 7.9448 −2.30 [−5.56; 0.96] 100.0% 100.0%
PSYE+GS+SM+RIF+SC+PRP+SS+PI+MODELING+G:ME
Savoye_2011 105 4.50 3.6600 69 3.50 4.0300 1.00 [−0.18; 2.18] 100.0% 100.0%
PSYE+GS+SM+RIF+SC+SG+PI+MODELING+G:ME
Johnston_2010 40 2.76 1.6600 20 2.35 1.4800 0.41 [−0.42; 1.24] 49.5% 49.5%
Johnston_2013 46 2.70 1.6900 25 2.54 1.6800 0.16 [−0.66; 0.98] 50.5% 50.5%
Common effect model 86 45 0.28 [−0.30; 0.87] 100.0% .
Random effects model 0.28 [−0.30; 0.87] . 100.0%
Heterogeneity: I^2 = 0%, τ^2 = 0, p = 0.6741
PSYE+GS+TASK+SS+PI+G+RD:ME
Waling_2012 48 3.67 3.3600 45 4.48 3.5300 −0.81 [−2.21; 0.59] 100.0% 100.0%
PSYE+MTV+PS+DM+RIF+SS+PI+G+RD:ME
Ruotsalainen_2015 15 1.00 0.9000 15 0.50 1.7000 0.50 [−0.47; 1.47] 100.0% 100.0%
PSYE+MTV+PS+DM+RIF+SS+PI+G+RD:PSYE+MTV+PS+SS+PI+G+RD
Ruotsalainen_2015 15 1.00 0.9000 16 0.70 0.6000 0.30 [−0.24; 0.84] 100.0% 100.0%
PSYE+MTV+PS+SS+PI+G+RD:ME
Ruotsalainen_2015 16 0.70 0.6000 15 0.50 1.7000 0.20 [−0.71; 1.11] 100.0% 100.0%
PSYE+MTV+TASK+SC+SG+PI+G:ME
Patsopoulou_2017 60 0.40 1.3800 61 0.90 1.3900 −0.50 [−0.99; −0.01] 100.0% 100.0%
PSYE+PS+GS+DM+FB+RIF+SC+ROLE+PI+MODELING+RULE+G:ME
Stark_2019 47 3.64 0.9000 54 3.79 0.9100 −0.15 [−0.50; 0.20] 100.0% 100.0%
PSYE+PS+GS+DM+FB+RIF+SC+ROLE+PI+MODELING+RULE+G:PSYE+PS+GS+PI
Stark_2019 47 3.64 0.9000 50 3.89 0.9100 −0.25 [−0.61; 0.11] 100.0% 100.0%
PSYE+PS+GS+FB+RIF+PI+RULE+RD:ME
Taylor−2015 104 5.60 5.2557 102 6.10 4.7405 −0.50 [−1.87; 0.87] 100.0% 100.0%
PSYE+PS+GS+PI:ME
Stark_2019 50 3.89 0.9100 54 3.79 0.9100 0.10 [−0.25; 0.45] 100.0% 100.0%
PSYE+PS+GS+TASK+SM+DM+PRP+SS+PI+G:ME
Hofsteenge, G. H._2014 71 2.60 4.1847 51 1.30 3.5763 1.30 [−0.08; 2.68] 100.0% 100.0%
PSYE+SCI+GS+SM+DM+PI:ME
Ball_2011 15 0.90 4.8302 14 1.10 3.0024 −0.20 [−3.11; 2.71] 100.0% 100.0%
PSYE+SCI+GS+SM+DM+PI:PSYE+SCI+MTV+GS+SM+DM+PI
Ball_2011 15 0.90 4.8302 17 1.80 4.6131 −0.90 [−4.18; 2.38] 100.0% 100.0%
PSYE+SCI+GS+TASK+SM+DM+FB+RIF+PI+G:ME
Arlinghaus_2021_2 27 0.72 0.7300 32 1.45 1.0200 −0.73 [−1.18; −0.28] 49.9% 50.0%
Arlinghaus_2021_1 58 1.46 1.0700 54 1.64 1.3200 −0.18 [−0.63; 0.27] 50.1% 50.0%
Common effect model 85 86 −0.45 [−0.77; −0.14] 100.0% .
Random effects model −0.45 [−0.99; 0.08] . 100.0%
Heterogeneity: I^2 = 65.5%, τ^2 = 0.0991, p = 0.0885
PSYE+SCI+MTV+GS+SG+PI+STRESS+MODELING+G:PSYE+SCI+MTV+PS+GS+RMD+RIF+SG+PI+STRESS+MODELING+G+RD
Nguyen_2012 78 5.30 4.2095 73 4.90 3.9281 0.40 [−0.90; 1.70] 100.0% 100.0%

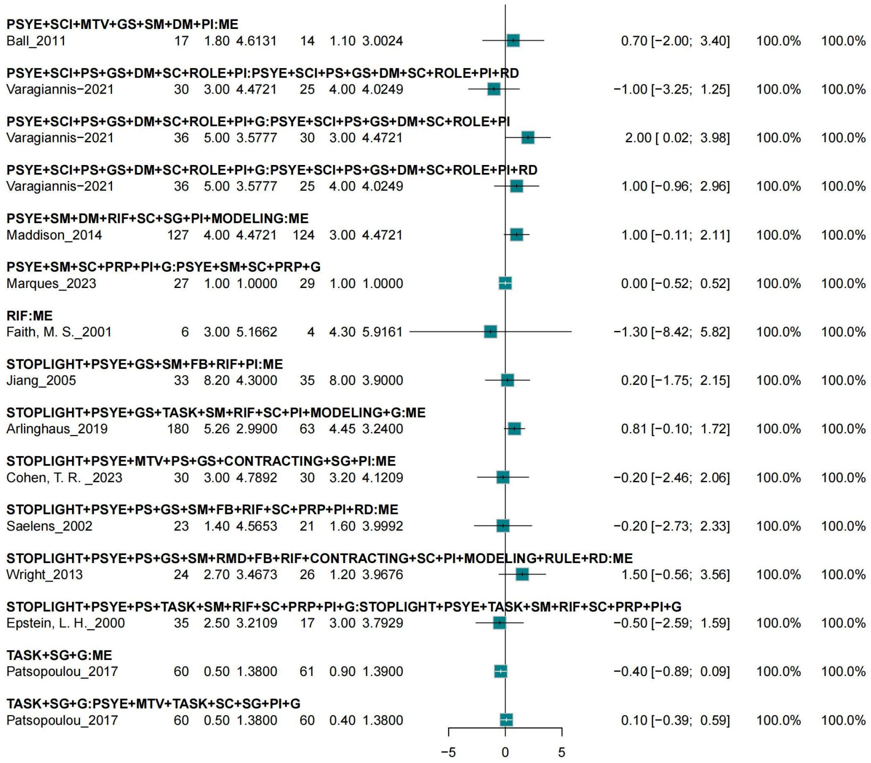

Notes: ME, minimal education; PSYE, psychoeducation; CR, cognitive restructuring; TWC, third-wave components; SCI, self-concept improvement; FBA, functional behavioural analysis; MTV, motivation; GS, goal-setting; TASK, task-setting; RULE, rule-setting; SM, self-monitoring; DM, device-monitoring; RMD, reminders; FB, feedback; PS, problem solving; PRP, preplanning; RIF, reinforcement; CONTRACTING, contracting; MODELING, modelling; SC, stimulus control; RT, relaxation training; IT, inhibition training; SS, social support; STRESS, stress management; STOPLIGHT, stoplight approach; ROLE, role playing; G, group; RD, remote; SG, serious games; PI, parental involvement

4.6.5 Quality of life

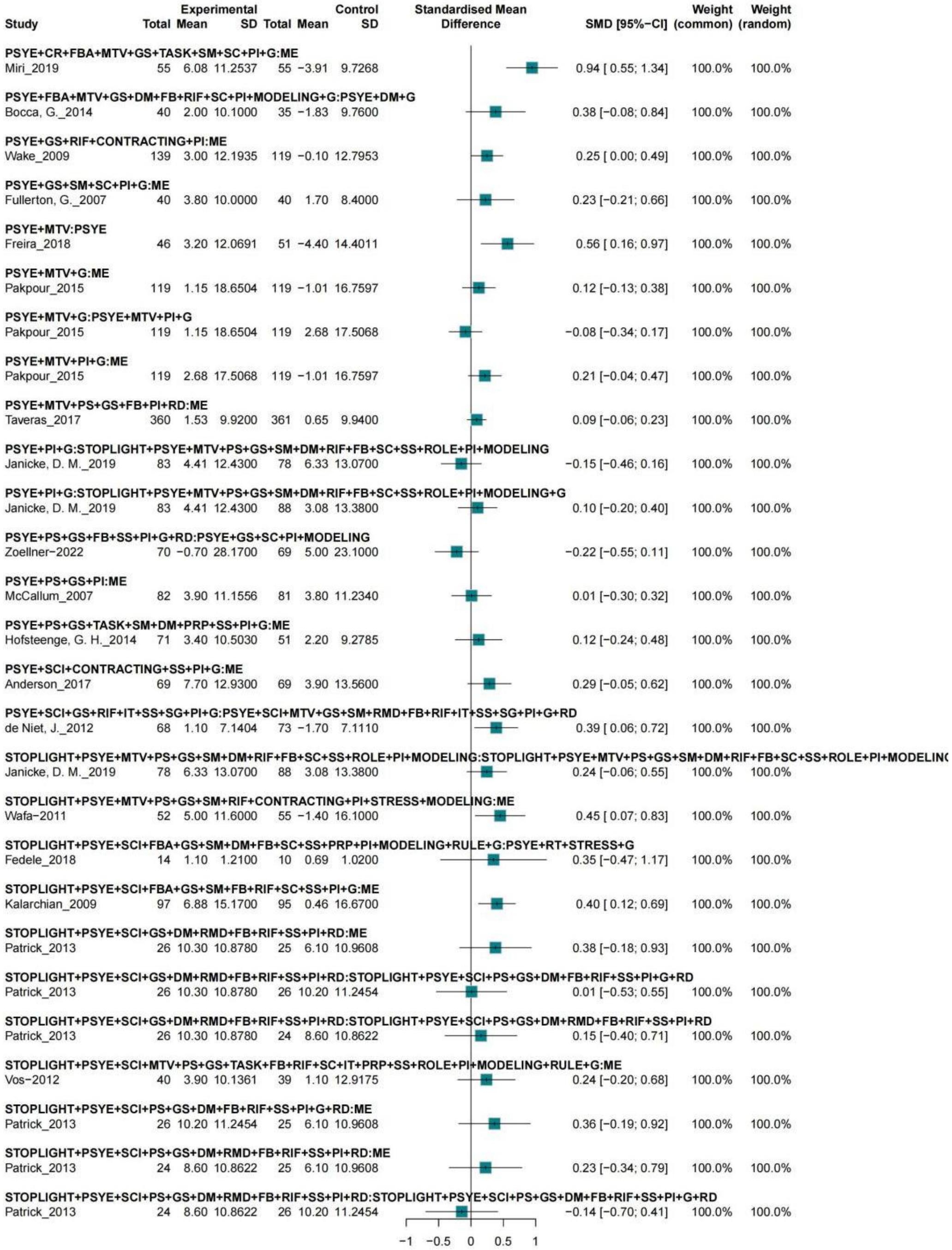

Notes: ME, minimal education; PSYE, psychoeducation; CR, cognitive restructuring; TWC, third-wave components; SCI, self-concept improvement; FBA, functional behavioural analysis; MTV, motivation; GS, goal-setting; TASK, task-setting; RULE, rule-setting; SM, self-monitoring; DM, device-monitoring; RMD, reminders; FB, feedback; PS, problem solving; PRP, preplanning; RIF, reinforcement; CONTRACTING, contracting; MODELING, modelling; SC, stimulus control; RT, relaxation training; IT, inhibition training; SS, social support; STRESS, stress management; STOPLIGHT, stoplight approach; ROLE, role playing; G, group; RD, remote; SG, serious games; PI, parental involvement

4.6.6 Mental health

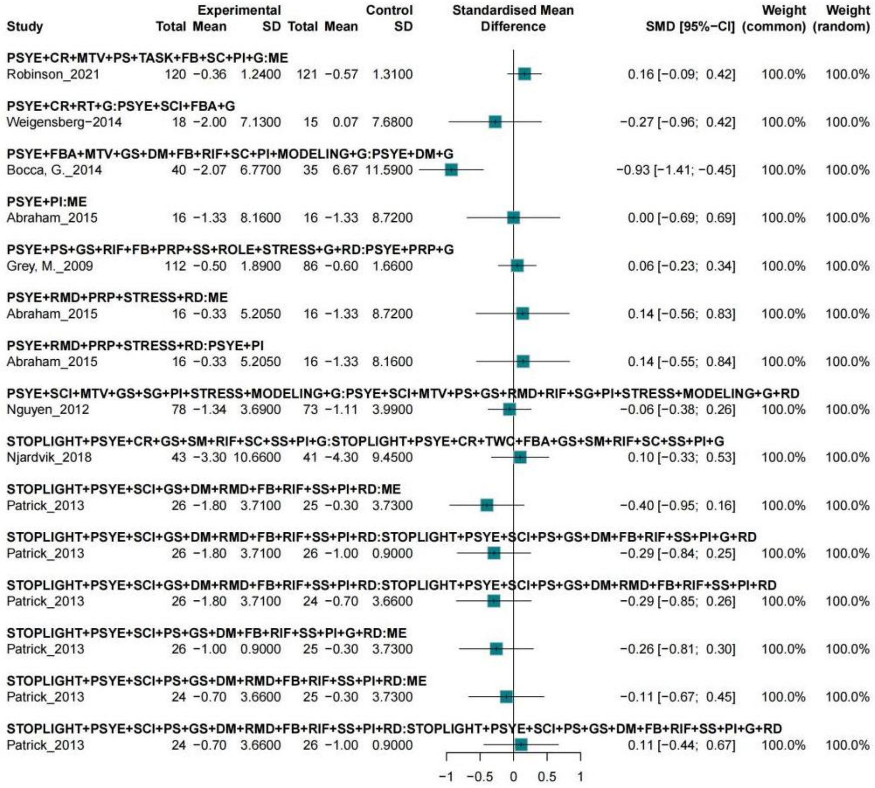

Notes: ME, minimal education; PSYE, psychoeducation; CR, cognitive restructuring; TWC, third-wave components; SCI, self-concept improvement; FBA, functional behavioural analysis; MTV, motivation; GS, goal-setting; TASK, task-setting; RULE, rule-setting; SM, self-monitoring; DM, device-monitoring; RMD, reminders; FB, feedback; PS, problem solving; PRP, preplanning; RIF, reinforcement; CONTRACTING, contracting; MODELING, modelling; SC, stimulus control; RT, relaxation training; IT, inhibition training; SS, social support; STRESS, stress management; STOPLIGHT, stoplight approach; ROLE, role playing; G, group; RD, remote; SG, serious games; PI, parental involvement.

CNMA

4.7 Heterogeneity (Inconsistency) assessed by pairwise meta-analyses

Notes: The study assess the between-study heterogeneity for all comparisons in each outcome by several pairwise random-effect meta-analyses. We first evaluated the potential heterogeneity if the point estimates vary greatly, or the confidence intervals are not overlapped, or the prediction intervals are contrary to the confidence intervals, or the P-value for Q-statistic is less than 0.1, or the I-squared is more than 50%. We then judged whether there is a clinical heterogeneity supervised by the clinical experts.

Direct estimate: mean difference for continuous outcomes (MD), except for quality-of-life score change and mental health score change; standardised mean difference (SMD) for quality-of-life score change and mental health score change.

4.7.1 Heterogeneity (Inconsistency) assessment in conceptual level NMA

Outcomes	Comparisons	Estimates (95% CI)	Prediction Interval	k	Q	pval.Q	tau^2	I^2
BMI z-score	BT vs CBT	0.08 (-0.03 to 0.18)	NA	2	0.515	0.473	0	0
BMI z-score	BT vs EDU	0 (-0.17 to 0.17)	NA	3	10.169	0.006	0.018	0.803
BMI z-score	BT vs ME	-0.08 (-0.11 to -0.05)	-0.22 to 0.06	41	121.177	0	0.004	0.67
BMI z-score	CBT vs CT	-0.07 (-0.21 to 0.07)	NA	1	0	NA	NA	NA
BMI z-score	CBT vs EDU	0 (-0.07 to 0.07)	NA	2	0.234	0.628	0	0
BMI z-score	CBT vs ME	-0.06 (-0.09 to -0.03)	-0.18 to 0.06	26	52.212	0.001	0.003	0.521
BMI z-score	CT vs EDU	0 (-0.15 to 0.15)	NA	1	0	NA	NA	NA
Body fat percentage	BT vs CBT	0.54 (0 to 1.09)	NA	1	0	NA	NA	NA
Body fat percentage	BT vs EDU	-1.68 (-2.9 to -0.46)	NA	4	9.228	0.026	1.035	0.675
Body fat percentage	BT vs ME	-1.26 (-2.09 to -0.44)	-4.69 to 2.17	17	94.761	0	2.44	0.831
Body fat percentage	CBT vs EDU	-0.3 (-1.38 to 0.78)	NA	1	0	NA	NA	NA
Body fat percentage	CBT vs ME	-1.02 (-1.88 to -0.16)	-3.78 to 1.73	10	41.893	0	1.291	0.785
Body fat percentage	EDU vs ME	-2.79 (-6.14 to 0.56)	NA	1	0	NA	NA	NA
Waist circumference	BT vs CBT	0.45 (-0.3 to 1.21)	NA	2	0.186	0.666	0	0
Waist circumference	BT vs EDU	-2.67 (-4.56 to -0.78)	NA	3	3.297	0.192	1.113	0.393
Waist circumference	BT vs ME	-1.65 (-2.65 to -0.65)	-5.78 to 2.48	17	434.478	0	3.538	0.963
Waist circumference	CBT vs ME	-1.72 (-2.93 to -0.5)	-6.59 to 3.16	16	91.626	0	4.852	0.836
Waist circumference	EDU vs ME	-0.37 (-3.84 to 3.1)	NA	1	0	NA	NA	NA
Height	BT vs EDU	0.51 (-0.7 to 1.72)	NA	1	0	NA	NA	NA
Height	BT vs ME	0.24 (-0.06 to 0.54)	-0.57 to 1.05	17	23.695	0.096	0.122	0.325
Height	CBT vs ME	-1.16 (-2.96 to 0.65)	NA	4	23.922	0	2.962	0.875
Quality of life	BT vs CBT	-0.36 (-1.18 to 0.46)	NA	1	0	NA	NA	NA
Quality of life	BT vs EDU	0.27 (-0.27 to 0.81)	NA	2	5.025	0.025	0.123	0.801
Quality of life	BT vs ME	0.15 (0.05 to 0.24)	0.03 to 0.27	7	4.68	0.585	0	0
Quality of life	CBT vs ME	0.44 (0.2 to 0.69)	-0.21 to 1.1	5	8.287	0.082	0.04	0.517
Mental health	BT vs EDU	1 (-3.74 to 5.74)	NA	1	0	NA	NA	NA
Mental health	BT vs ME	1 (-3.98 to 5.98)	NA	1	0	NA	NA	NA
Mental health	CBT vs ME	-0.04 (-0.93 to 0.86)	NA	2	1.679	0.195	0.24	0.404
Mental health	EDU vs ME	0 (-5.85 to 5.85)	NA	1	0	NA	NA	NA

Notes: BT, behavioural therapy; CBT, cognitive behavioural therapy; CT, cognitive therapy; EDU, psychoeducation; ME, minimal education; k, no. of studies; Q, Q statistic; I, I statistic; tau, standard deviation of the random-effect distribution; CI, confidence interval

4.7.2 Heterogeneity (Inconsistency) assessment in technical level CNMA

Outcomes	Comparisons	MD (95% CI)	Prediction Interval	k	Q	pval. Q	tau^2	I^2
BMI z-score	STOPLIGHT+PSYE+MTV+PS+GS+CONTRACTING+SG+PI vs ME	-0.15 (-0.35 to 0.06)	NA	2	1.595	0.207	0.009	0.373
BMI z-score	MTV vs ME	-0.12 (-0.27 to 0.04)	NA	2	1.312	0.252	0.004	0.238
BMI z-score	G vs ME	-0.03 (-0.13 to 0.07)	-0.37 to 0.31	7	48.255	0	0.016	0.876
BMI z-score	PI vs ME	0.12 (-0.08 to 0.32)	NA	2	4.998	0.025	0.016	0.8
BMI z-score	PSYE+SCI+PS+GS+SM+DM+FB+SS+PI+RD vs ME	-0.04 (-0.14 to 0.05)	NA	2	1.075	0.3	0	0.07
BMI z-score	PS vs ME	-0.09 (-0.41 to 0.22)	NA	3	7.461	0.024	0.058	0.732
BMI z-score	PSYE+PS+GS+PI vs ME	0.01 (-0.14 to 0.15)	NA	2	3.104	0.078	0.008	0.678
BMI z-score	DM+FB vs ME	0.03 (-0.08 to 0.14)	NA	2	0.332	0.564	0	0
BMI z-score	PSYE+SCI+GS+TASK+SM+DM+FB+RIF+PI+G vs ME	0.01 (-0.11 to 0.14)	NA	2	8.132	0.004	0.007	0.877
BMI z-score	PSYE+MTV+GS+SM+RMD+FB+RD vs ME	0.05 (-0.02 to 0.13)	NA	3	4.022	0.134	0.002	0.503
Body fat percentage	STOPLIGHT+PSYE+MTV+PS+GS+CONTRACTING+SG+PI vs ME	-0.23 (-0.47 to 0)	NA	2	0.24	0.624	0	0
Body fat percentage	PSYE+GS+SM+RIF+SC+SG+PI+MODELING+G vs ME	-1.92 (-3.19 to -0.65)	NA	2	0.007	0.931	0	0
Waist circumference	STOPLIGHT+PSYE+MTV+PS+GS+CONTRACTING+SG+PI vs ME	-1.82 (-3.76 to 0.11)	NA	2	0.229	0.633	0	0
Waist circumference	MTV vs ME	-3.95 (-5.94 to -1.95)	NA	2	0.555	0.456	0	0
Height	PSYE+SCI+GS+TASK+SM+DM+FB+RIF+PI+G vs ME	-0.45 (-0.99 to 0.08)	NA	2	2.902	0.088	0.099	0.655
Height	PSYE+GS+SM+RIF+SC+SG+PI+MODELING+G vs ME	0.28 (-0.3 to 0.87)	NA	2	0.177	0.674	0	0

Notes: ME, minimal education; PSYE, psychoeducation; CR, cognitive restructuring; TWC, third-wave components; SCI, self-concept improvement; FBA, functional behavioural analysis; MTV, motivation; GS, goal-setting; TASK, task-setting; RULE, rule-setting; SM, self-monitoring; DM, device-monitoring; RMD, reminders; FB, feedback; PS, problem solving; PRP, preplanning; RIF, reinforcement; CONTRACTING, contracting; MODELING, modelling; SC, stimulus control; RT, relaxation training; IT, inhibition training; SS, social support; STRESS, stress management; STOPLIGHT, stoplight approach; ROLE, role playing; G, group; RD, remote; SG, serious games; PI, parental involvement; k, no. of studies; Q, Q statistic; I, I statistic; tau, standard deviation of the random-effect distribution; CI, confidence interval

4.8 Publication bias assessments

4.8.1BMI z-score

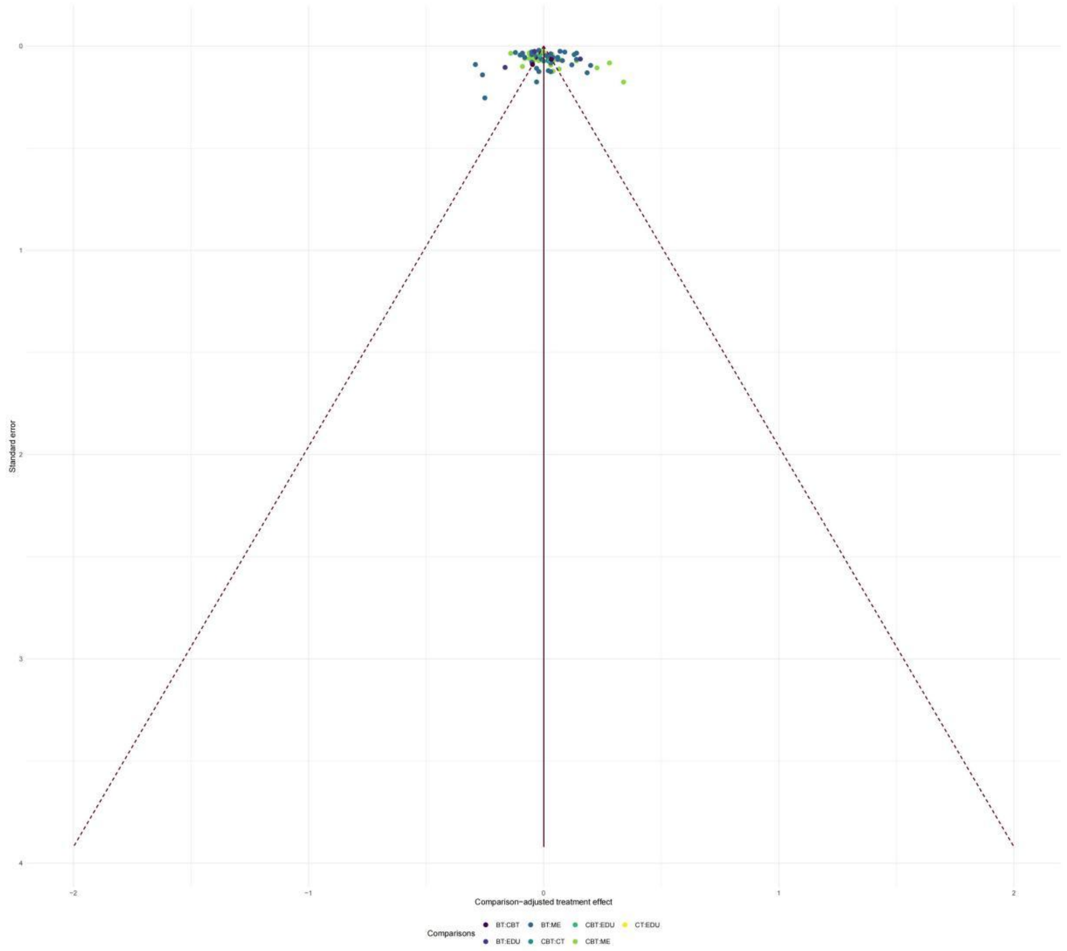

Notes: BT, behavioural therapy; CBT, cognitive behavioural therapy; CT, cognitive therapy; EDU, psychoeducation; ME, minimal education

4.8.2 Body fat

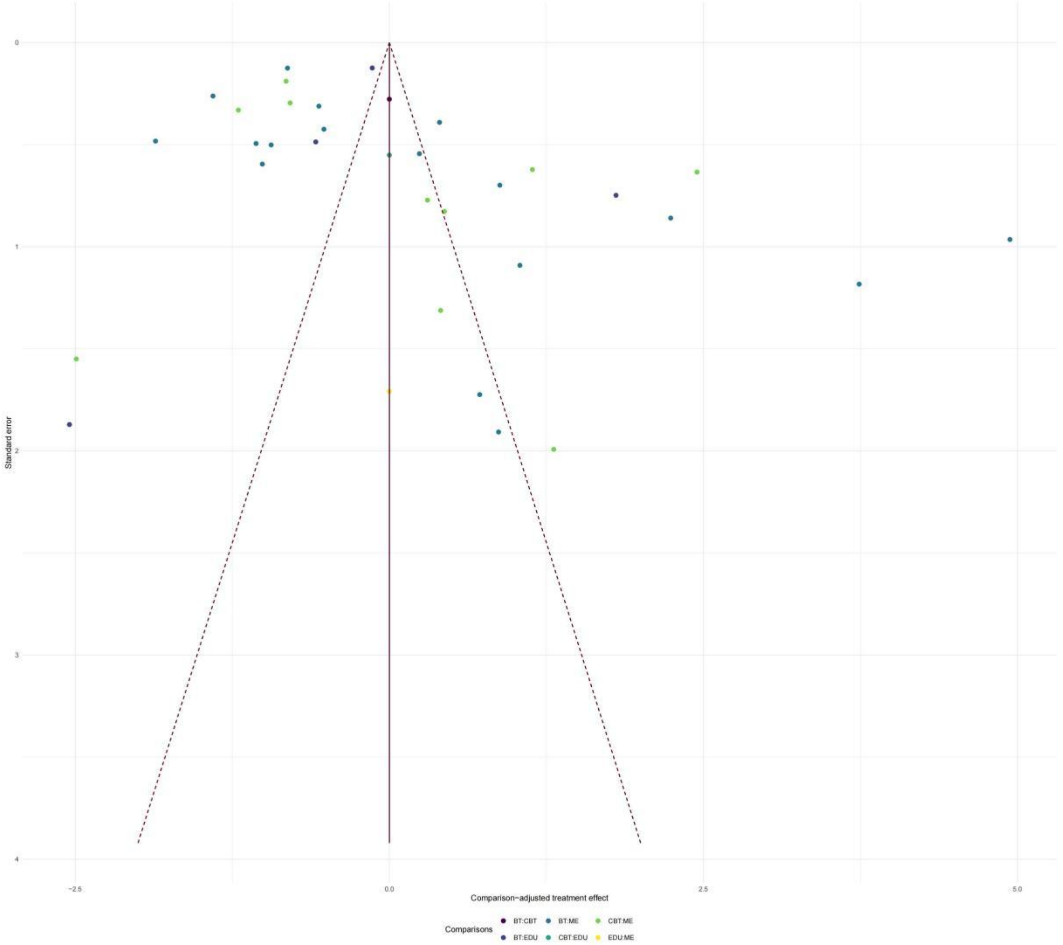

Notes: BT, behavioural therapy; CBT, cognitive behavioural therapy; CT, cognitive therapy; EDU, psychoeducation; ME, minimal education

4.8.3 Waist circumference

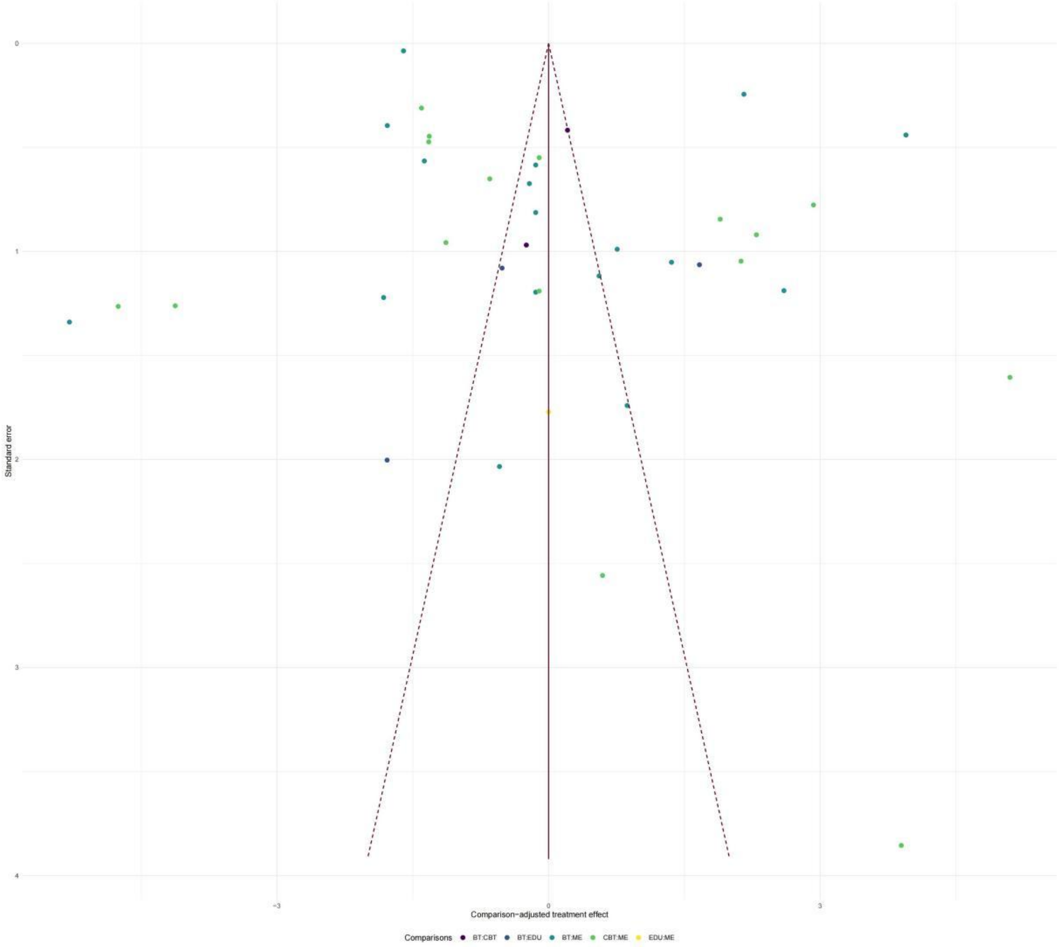

Notes: BT, behavioural therapy; CBT, cognitive behavioural therapy; CT, cognitive therapy; EDU, psychoeducation; ME, minimal education

4.8.4 Height

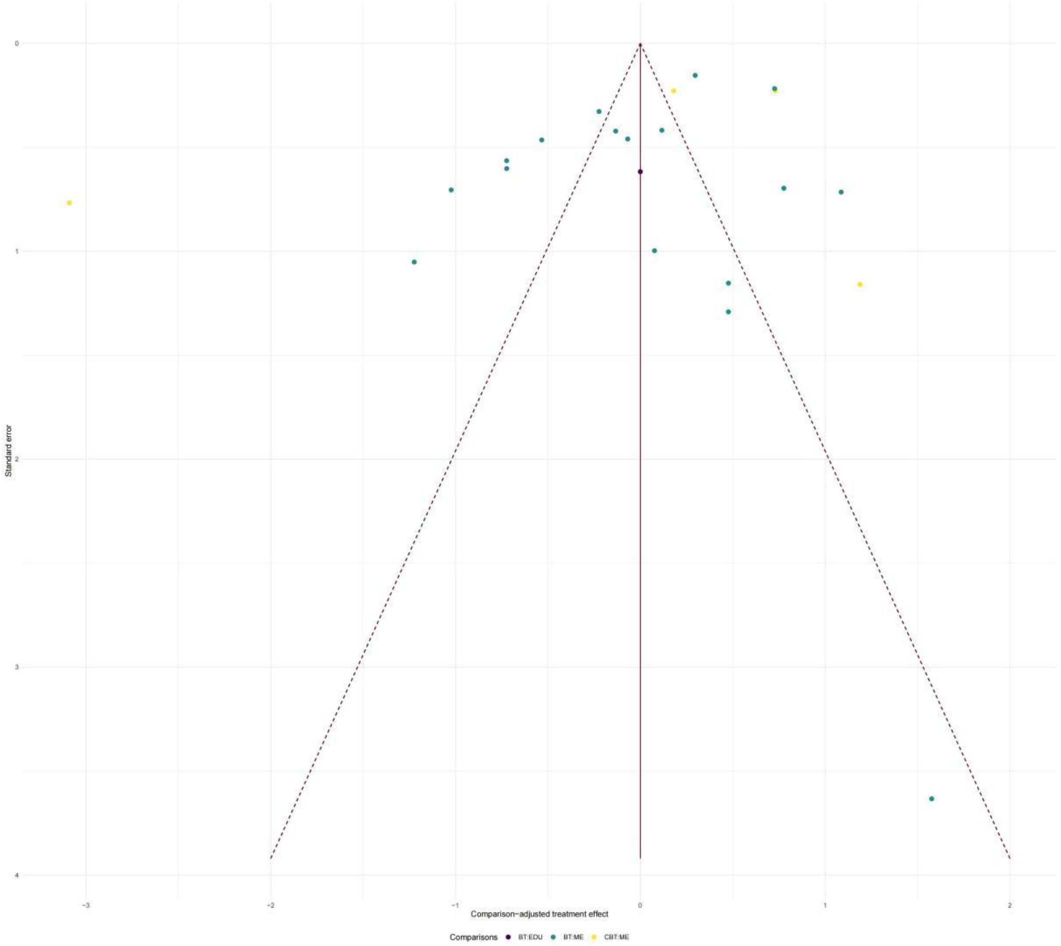

Notes: BT, behavioural therapy; CBT, cognitive behavioural therapy; CT, cognitive therapy; EDU, psychoeducation; ME, minimal education

4.9 Intransitivity assessments

Notes: The study assessed the intransitivity between direct comparisons by comparing their distribution of patients' characteristics. We chose the potential effect modifiers from the prognostic variables identified by the prognostic research and the systematic review of risk prediction models. The illustrated figures show pooled mean or proportion and corresponding confidence interval by the random-effect single-arm meta-analyses via a random-intercept logistic regression. The duration of baseline diabetes was shown using median and interquartile due to skew distribution.

4.9.1 BMI z-score

4.9.1.1 Age

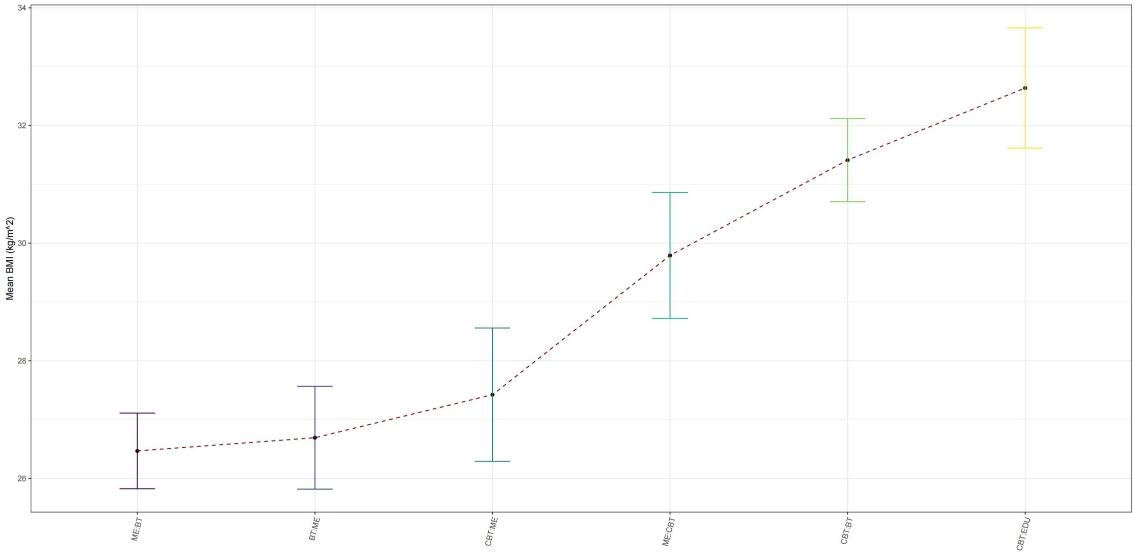

4.9.1.2 Body mass index

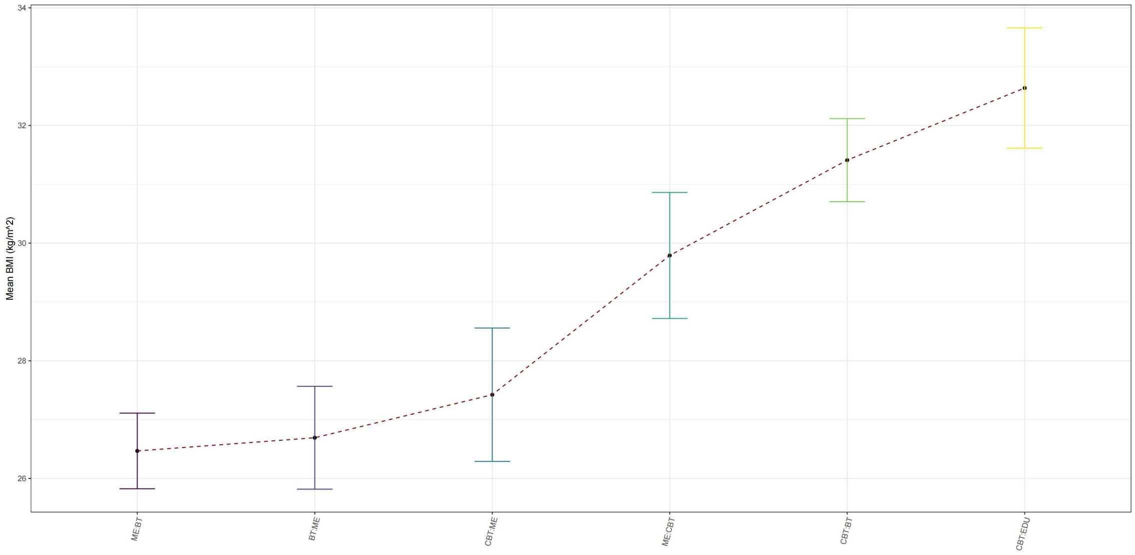

4.9.1.3 Duration of treatment

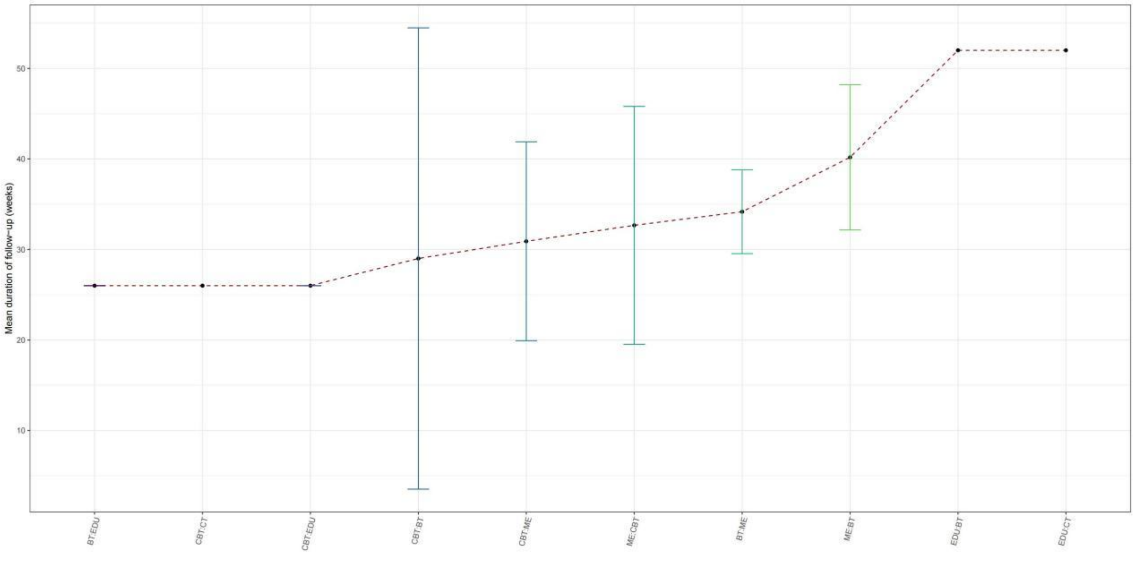

4.9.1.4 Proportion of girls

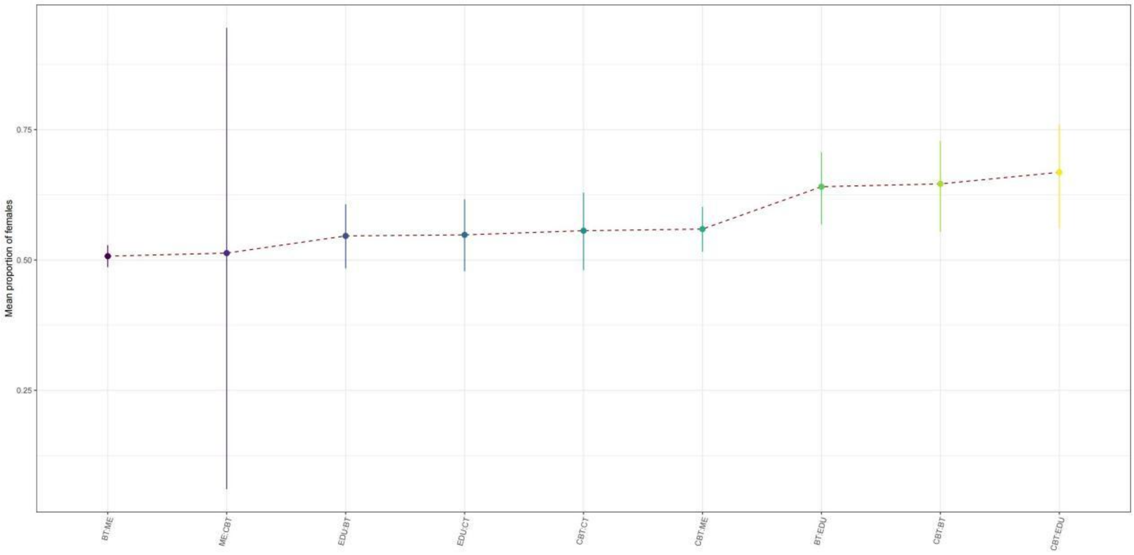

4.9.2 Body fat

4.9.2.1 Age

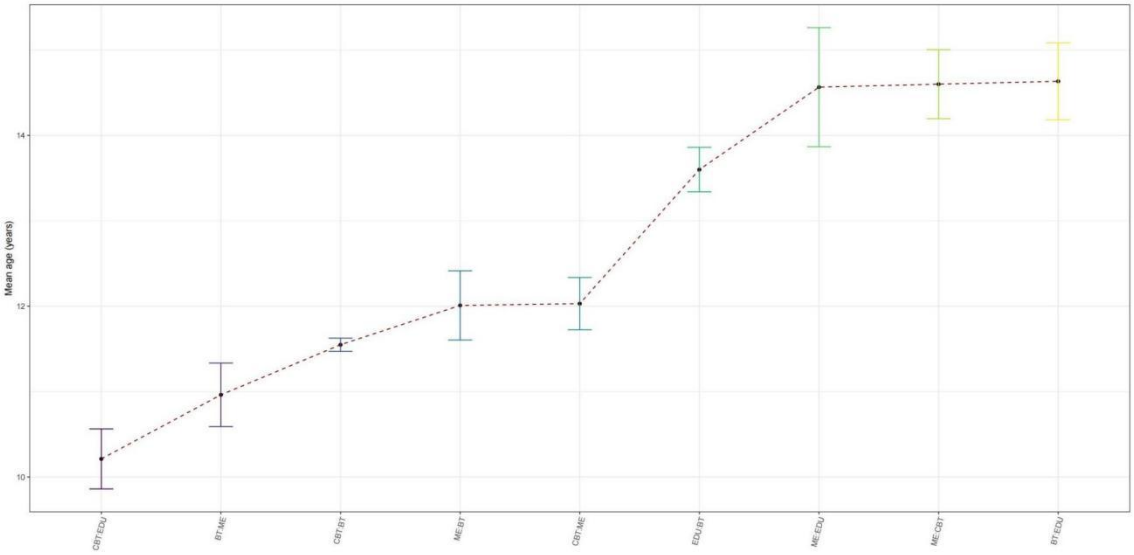

4.9.2.2 Body mass index

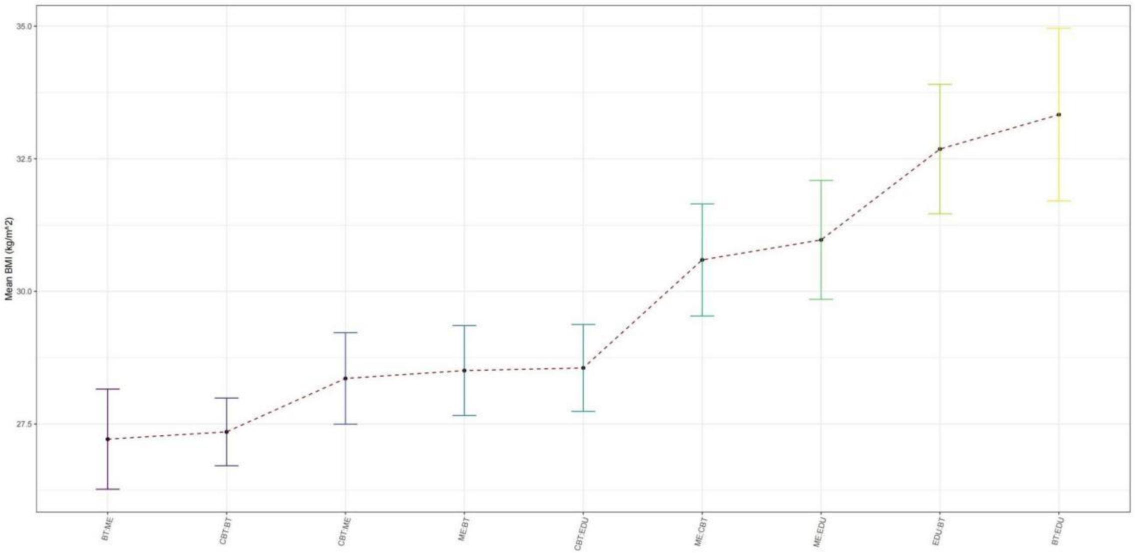

4.9.2.3 Duration of treatment

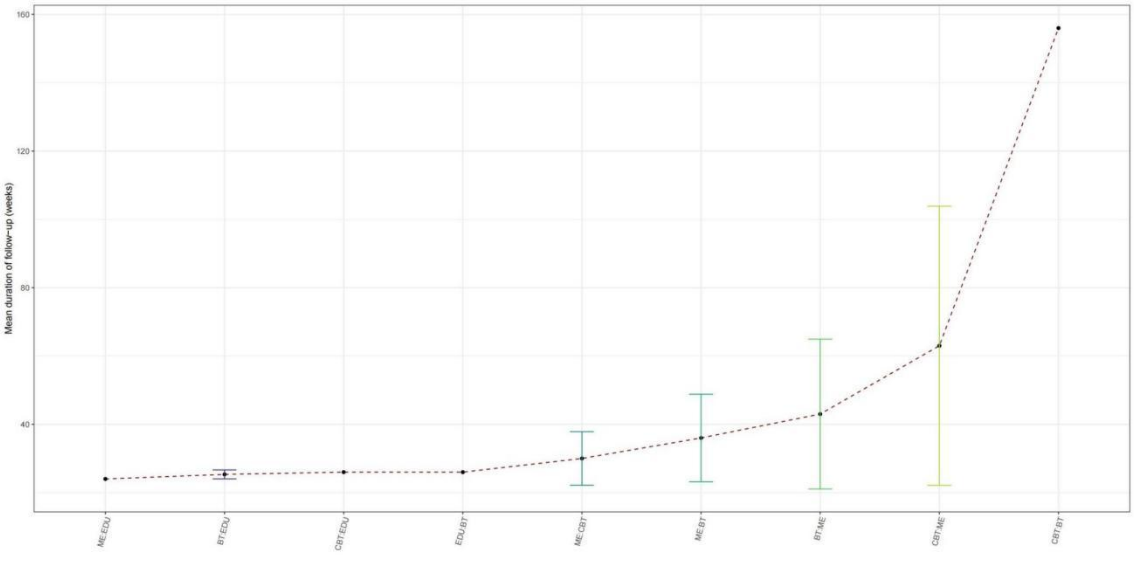

4.9.2.4 Proportion of girls

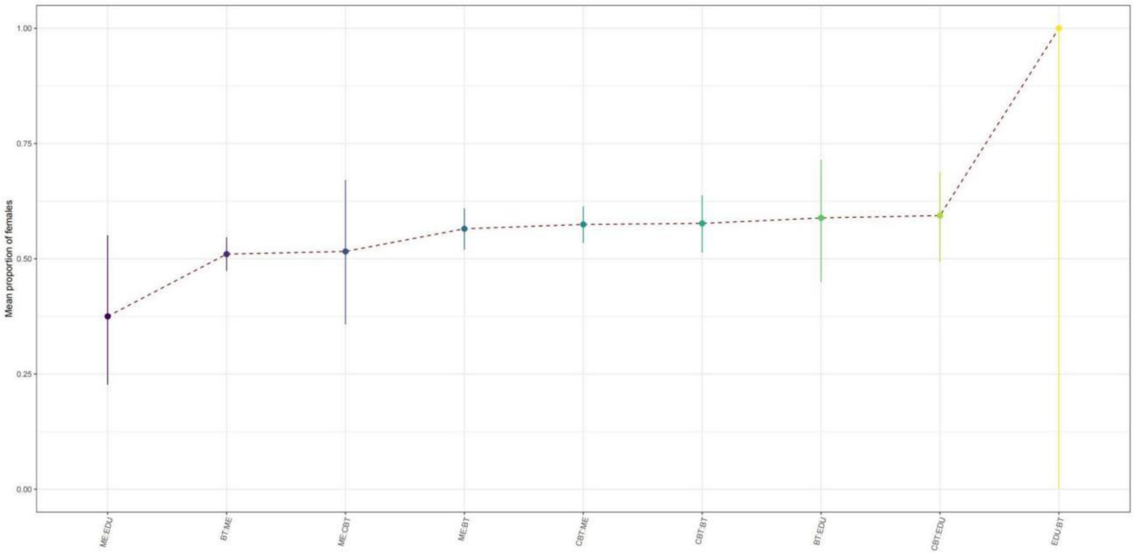

4.9.3 Waist circumference

4.9.3.1 Age

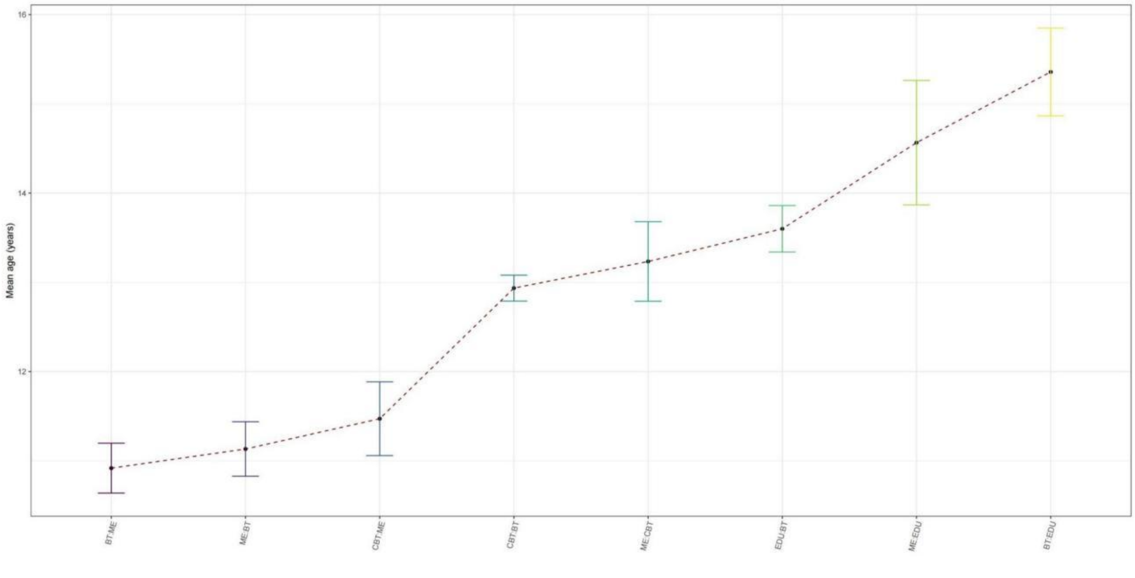

4.9.3.2 Body mass index

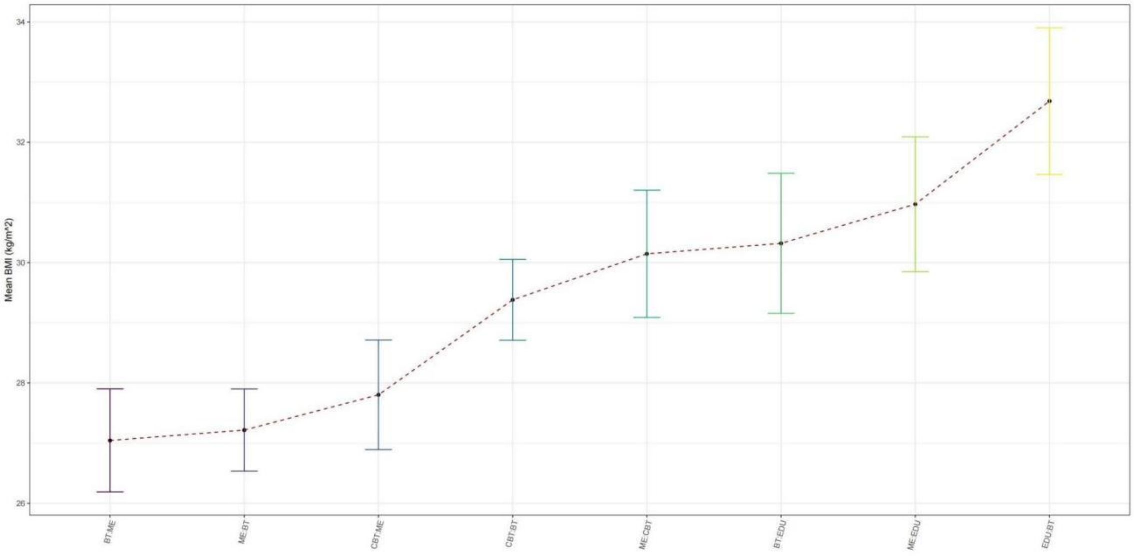

4.9.3.3 Duration of treatment

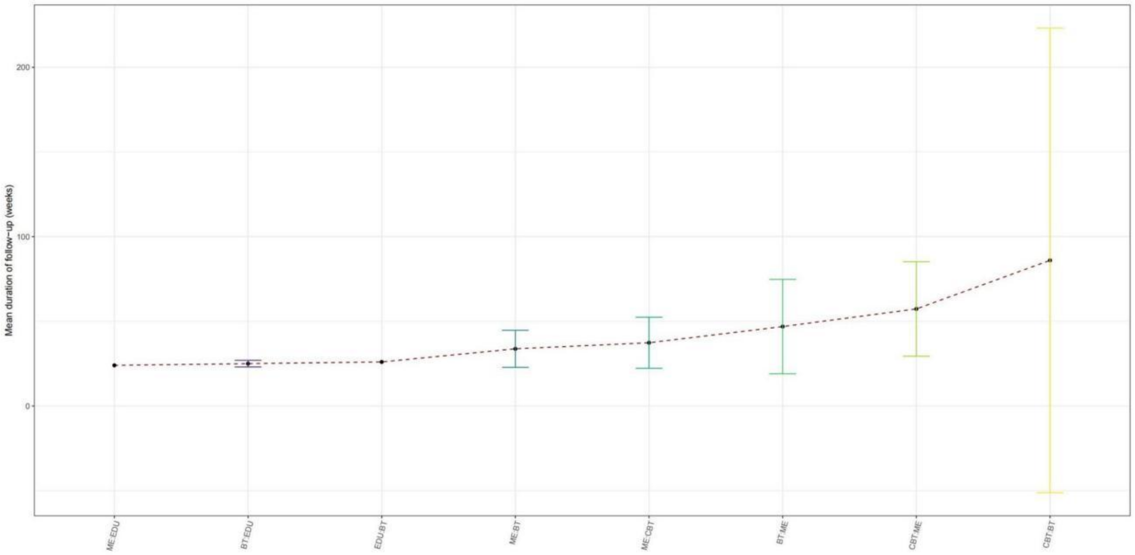

4.9.3.4 Proportion of girls

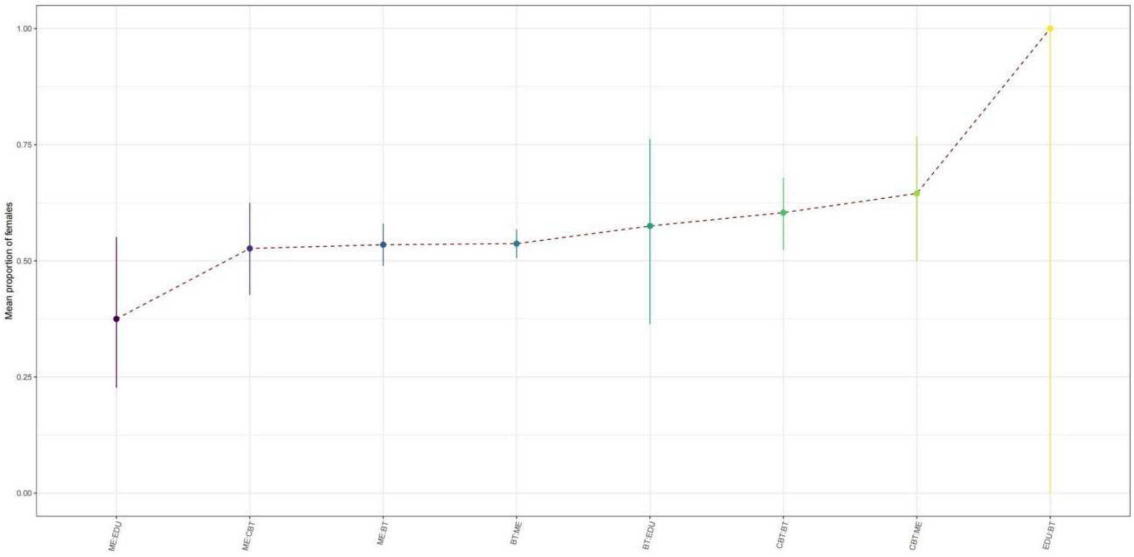

4.9.4 Height

4.9.4.1 Age

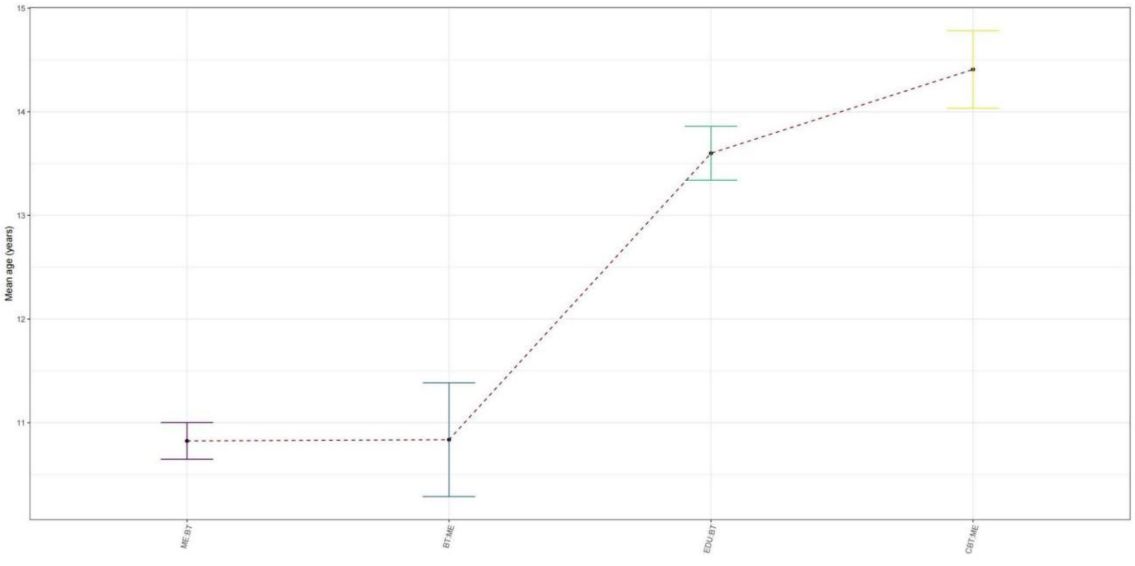

4.9.4.2 Body mass index

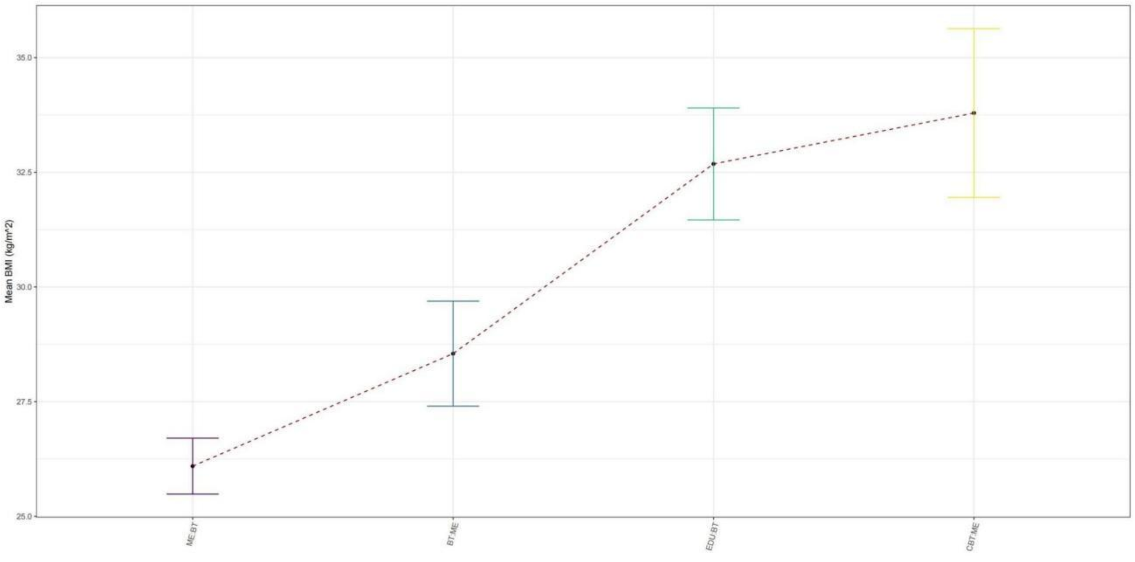

4.9.4.3 Duration of treatment

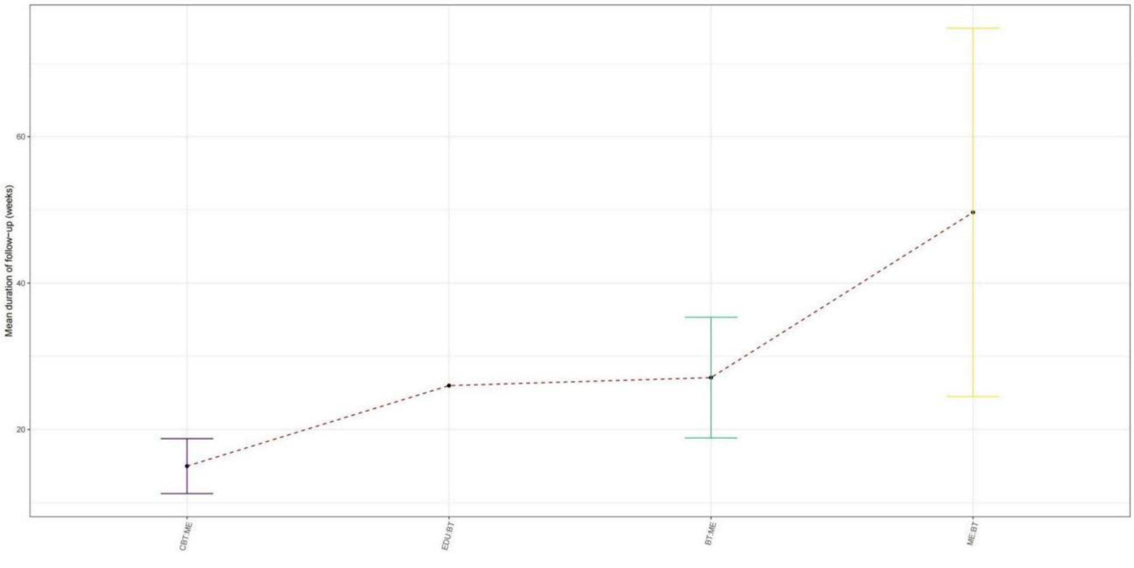

4.9.4.4 Proportion of girls

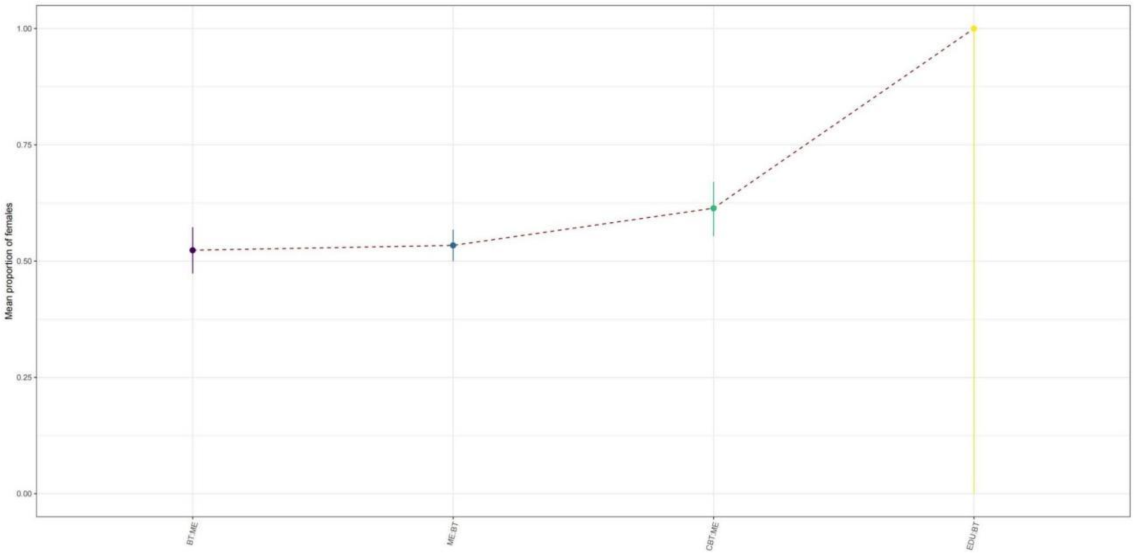

4.9.5 Quality of life

4.9.5.1 Age

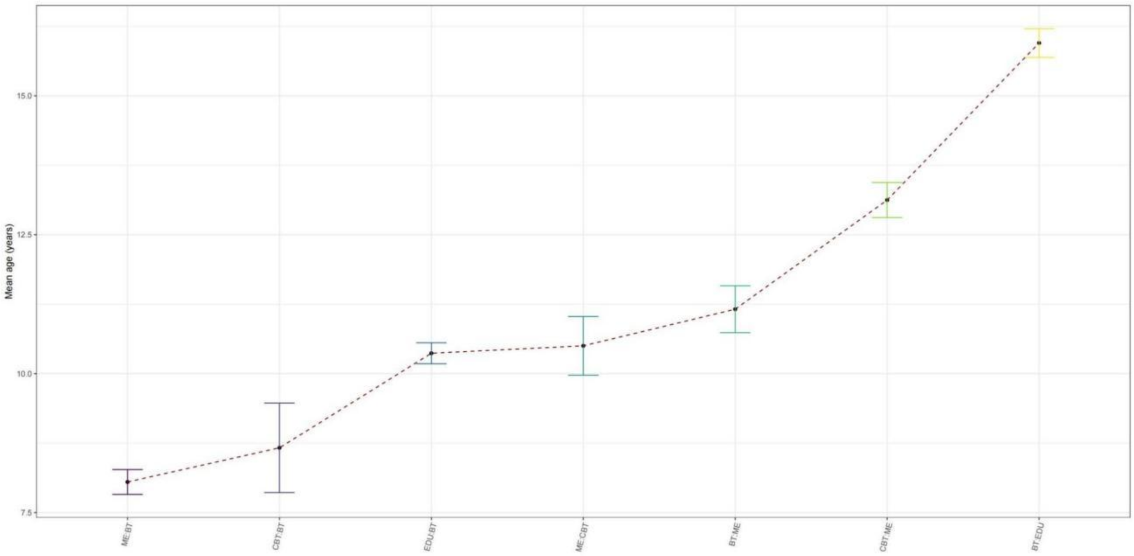

4.9.5.2 Body mass index

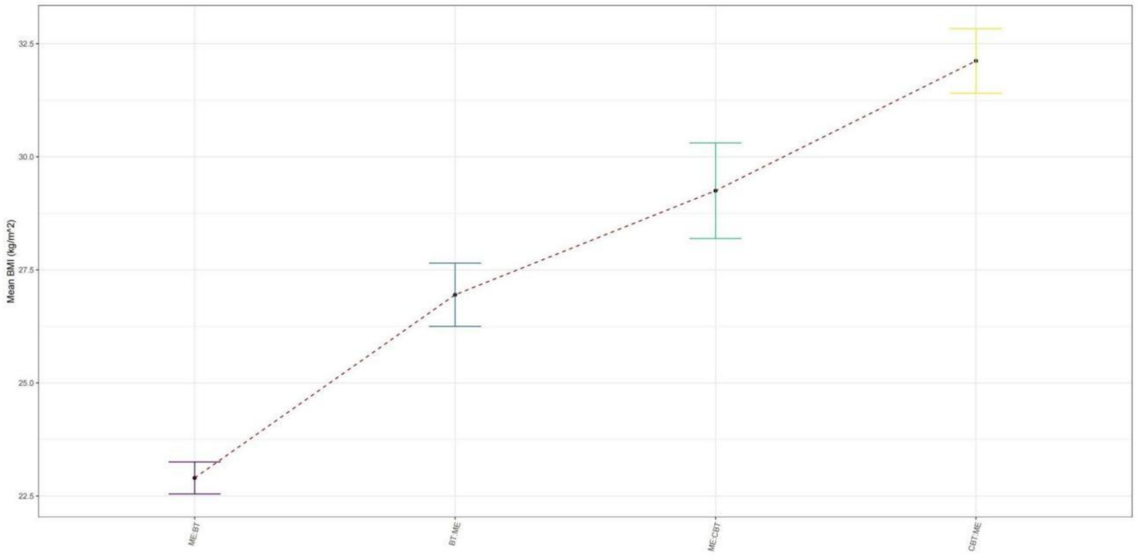

4.9.5.3 Duration of treatment

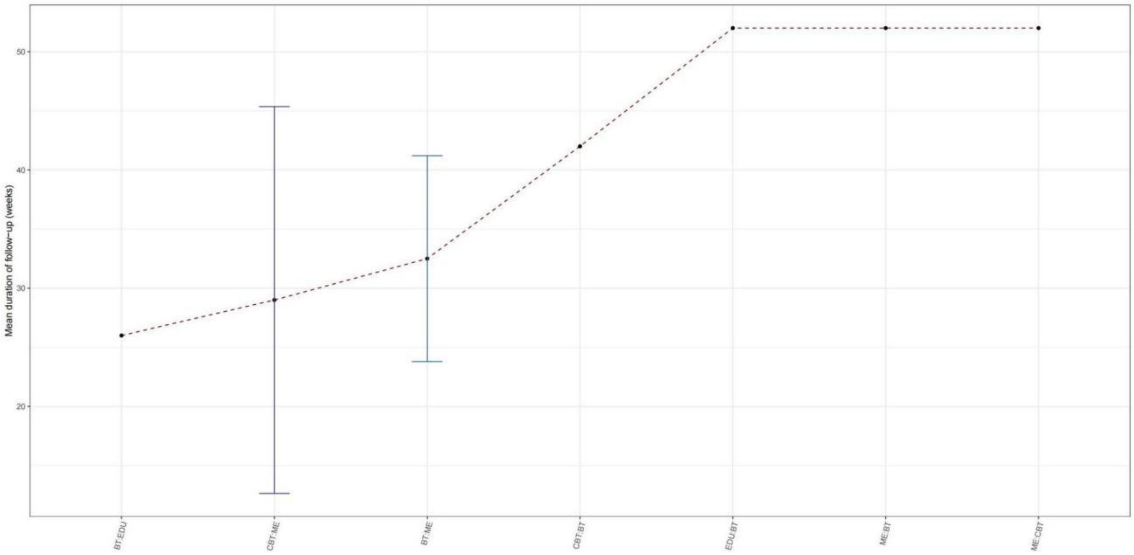

4.9.5.4 Proportion of girls

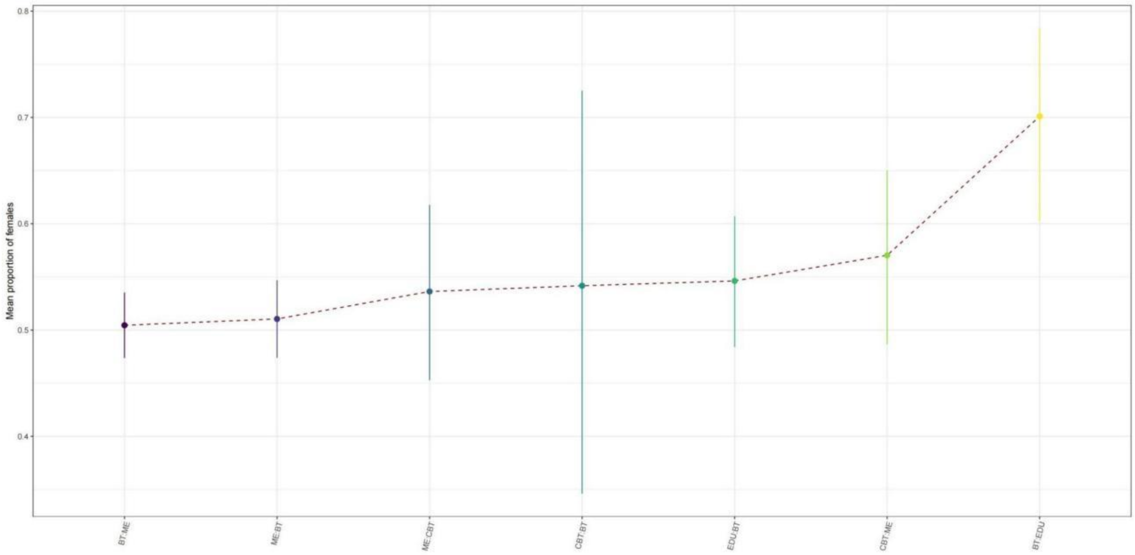

4.9.6 Mental health

4.9.6.1 Age

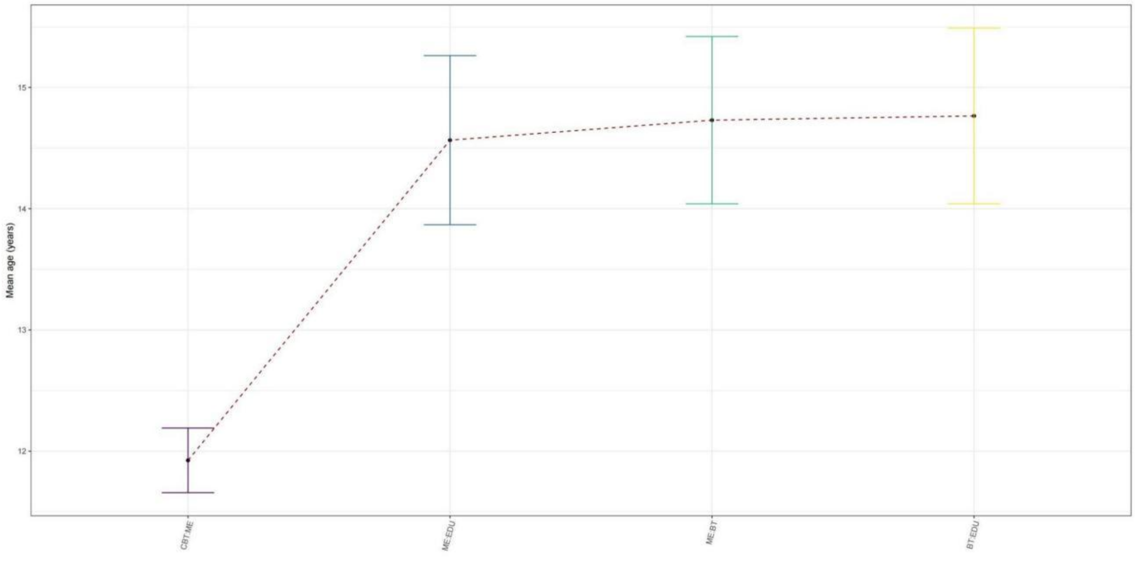

4.9.6.2 Body mass index

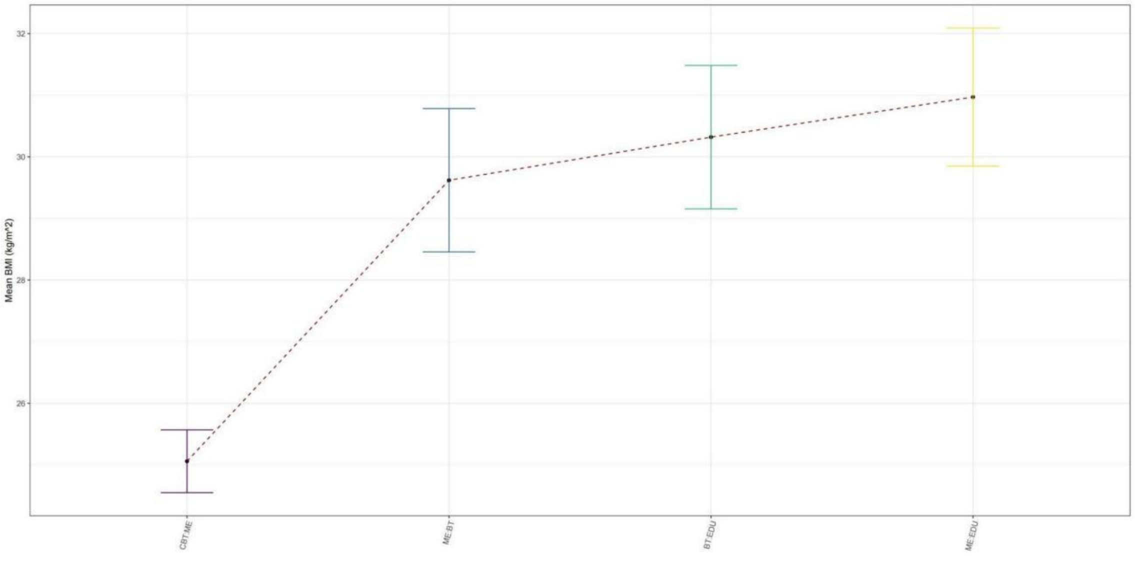

4.9.6.3 Duration of treatment

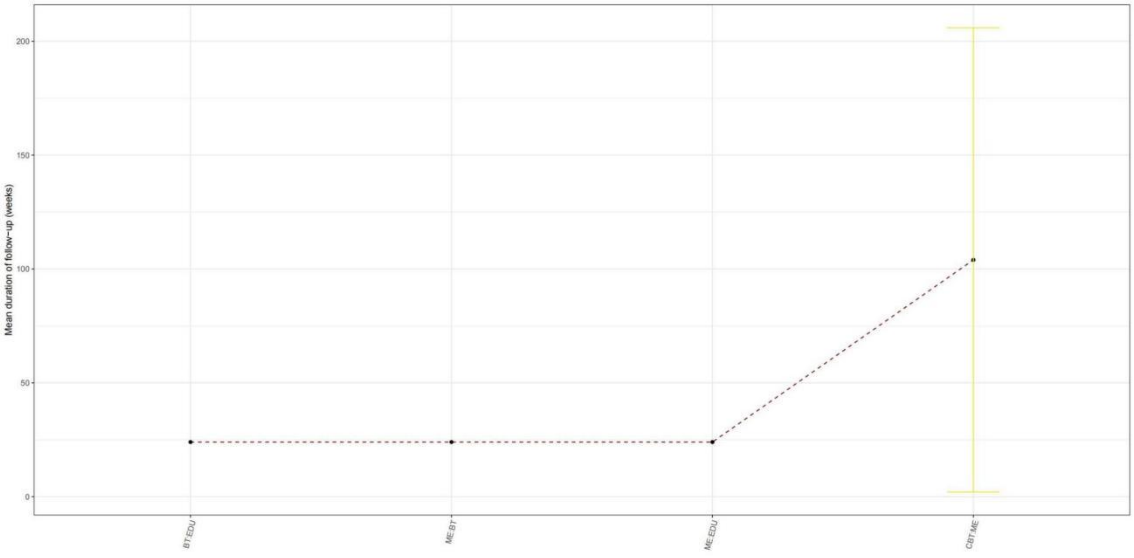

4.9.6.4 Proportion of girls

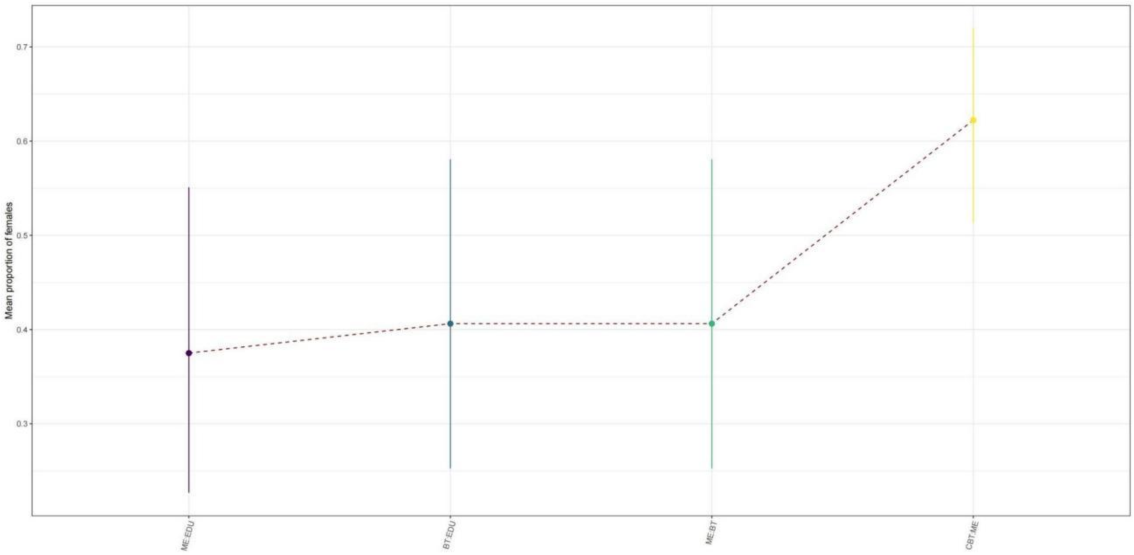

Appendix 5 GRADE certainty of evidence assessments

5.1 Summary of findings table in conceptual level NMA

Population: Children and adolescents with overweight or obesity

Settings: Any

Intervention: Conceptual-level treatment components of CBT

Comparison: Minimal education

Treatment Components	Outcomes Follow-up	Number of participants (studies) from direct evidence	Number of participants (studies) treated with the specific component	Certainty of the evidence (GRADE)	Anticipated effects (95% CI)	
					Risk with minimal education alone	Risk with target treatment
Behavioural therapy	**BMI z-score** (assessed at the end of treatment)	6419 (41 studies)	3934 (44 studies)	⊕⊕⊖⊖ Low[e]	The mean BMI z-score at the end of the treatment ranged from 1.34 to 4.2.	0.08 lower BMI z-score (0.10 lower to 0.05 lower)
	**Body fat (%)** (assessed at the end of treatment)	2318 (17 studies)	1428 (20 studies)	⊕⊕⊕⊖ Moderate[b]	The mean fat mass at the end of the treatment ranged from 26.2% to 47.9%.	1.16 lower fat mass % (1.68 lower to 0.64 lower)
	**Waist circumference (cm)** (assessed at the end of treatment)	2225 (17 studies)	1438 (21 studies)	⊕⊕⊕⊖ Moderate[b]	The mean waist circumference at the end of the treatment ranged from 66 cm to 115.2 cm.	1.70 lower waist circumference in cm (2.74 lower to 0.67 lower)
	**Height (cm)** (assessed at the end of treatment)	1900 (17 studies)	1173 (18 studies)	⊕⊕⊕⊖ Moderate[c]	The mean height at the end of the treatment ranged from 121.5 cm to 171.7 cm.	0.28 higher height in cm (0.09 lower to 0.65 higher)
	**Quality-of-life score** (assessed at the end of treatment)	1808 (7 studies)	1204 (10 studies)	⊕⊕⊕⊖ Moderate[a]	The mean quality-of-life score at the end of the treatment ranged from 63.7 to 86.6.	0.16 higher quality-of-life score (0.03 higher to 0.30 higher)
	**Mental health score** (assessed at the end of treatment)	32 (1 study)	16 (1 study)	⊕⊕⊕⊖ Moderate[c]	The mean mental health score at the end of the treatment ranged from 5.7 to 6.67.	1.00 higher mental health score (4.07 lower to 6.07 higher)
Cognitive behavioural therapy	**BMI z-score** (assessed at the end of treatment)	2619 (26 studies)	1622 (29 studies)	⊕⊖⊖⊖ Very low[f]	The mean BMI z-score at the end of the treatment ranged from 1.34 to 4.2.	0.07 lower BMI z-score (0.10 lower to 0.03 lower)
	**Body fat (%)** (assessed at the end of treatment)	1277 (10 studies)	710 (11 studies)	⊕⊕⊖⊖ Low[d]	The mean fat mass at the end of the treatment ranged from 26.2% to 47.9%.	1.00 lower fat mass % (1.69 lower to 0.31 lower)
	**Waist circumference (cm)** (assessed at the end of treatment)	2268 (16 studies)	1200 (17 studies)	⊕⊕⊖⊖ Low[d]	The mean waist circumference at the end of the treatment ranged from 66 cm to 115.2 cm.	1.76 lower waist circumference in cm (2.86 lower to 0.67 lower)

Treatment Components	Outcomes Follow-up	Number of participants (studies) from direct evidence	Number of participants (studies) treated with the specific component	Certainty of the evidence (GRADE)	Anticipated effects (95% CI)	
					Risk with minimal education alone	Risk with target treatment
	**Height (cm)** (assessed at the end of treatment)	264 (4 studies)	142 (3 studies)	⊕⊕⊕⊝ Moderate[b]	The mean height at the end of the treatment ranged from 121.5 cm to 171.7 cm.	0.91 lower height in cm (1.60 lower to 0.22 lower)
	**Quality-of-life score** (assessed at the end of treatment)	620 (5 studies)	351 (6 studies)	⊕⊕⊝⊝ Low[d]	The mean quality-of-life score at the end of the treatment ranged from 63.7 to 86.6.	0.44 higher quality-of-life score (0.26 higher to 0.63 higher)
	**Mental health score** (assessed at the end of treatment)	342 (2 studies)	196 (2 studies)	⊕⊕⊕⊝ Moderate[c]	The mean mental health score at the end of the treatment ranged from 5.7 to 6.67.	0.04 lower mental health score (0.93 lower to 0.86 higher)
**Cogníntive therapy**	**BMI z-score** (assessed at the end of treatment)	0 (0 study)	184 (2 studies)	⊕⊕⊝⊝ Low[e]	The mean BMI z-score at the end of the treatment ranged from 1.34 to 4.2.	0.03 lower BMI z-score (0.17 lower to 0.11 higher)
**Psychoeducation**	**BMI z-score** (assessed at the end of treatment)	0 (0 study)	408 (6 studies)	⊕⊝⊝⊝ Very low[c]	The mean BMI z-score at the end of the treatment ranged from 1.34 to 4.2.	0.06 lower BMI z-score (0.14 lower to 0.01 higher)
	**Body fat (%)** (assessed at the end of treatment)	32 (1 study)	216 (5 studies)	⊕⊝⊝⊝ Very low[c]	The mean fat mass at the end of the treatment ranged from 26.2% to 47.9%.	0.15 higher fat mass % (0.91 lower to 1.22 higher)
	**Waist circumference (cm)** (assessed at the end of treatment)	32 (1 study)	137 (3 studies)	⊕⊕⊝⊝ Low[e]	The mean waist circumference at the end of the treatment ranged from 66 cm to 115.2 cm.	0.74 higher waist circumference in cm (2.03 lower to 3.51 higher)
	**Height (cm)** (assessed at the end of treatment)	0 (0 study)	70 (1 study)	⊕⊕⊕⊝ Moderate[c]	The mean height at the end of the treatment ranged from 121.5 cm to 171.7 cm.	0.23 lower height in cm (1.85 lower to 1.39 higher)
	**Quality-of-life score** (assessed at the end of treatment)	0 (0 study)	134 (2 studies)	⊕⊝⊝⊝ Very low[c]	The mean quality-of-life score at the end of the treatment ranged from 63.7 to 86.6.	0.05 lower quality-of-life score (0.35 lower to 0.26 higher)
	**Mental health score** (assessed at the end of treatment)	32 (1 study)	16 (1 study)	⊕⊕⊕⊝ Moderate[c]	The mean mental health score at the end of the treatment ranged from 5.7 to 6.67.	0.00 higher mental health score (5.93 lower to 5.93 higher)

BMI: body mass index; CI: confidence interval

[a] Downgraded by one level for imprecision.

[a] Downgraded by one level for risk of bias.

[b] Downgraded by one level for heterogeneity.

[c] Downgraded by one level for imprecision.

[d] Downgraded by one level for risk of bias and one level for heterogeneity.

[e] Downgraded by one level for risk of bias and one level for imprecision.

[f] Downgraded by one level for risk of bias, one level for heterogeneity, and one level for imprecision.

NOTES: The downgrading reasons for risk of bias, heterogeneity, indirectness and publication bias were shown only for direct estimates due to the method limitation. The downgrading reasons due to inadditivity, intransitivity,

incoherence, and imprecision were shown for all pooled estimates.

High certainty: we are very confident that the true effect lies close to that of the estimate of the effect.
Moderate certainty: we are moderately confident in the effect estimate: the true effect is likely to be close to the estimate of the effect, but there is a possibility that it is substantially different.
Low certainty: our confidence in the effect estimate is limited: the true effect may be substantially different from the estimate of the effect.
Very low certainty: we have very little confidence in the effect estimate: the true effect is likely to be substantially different from the estimate of effect

5.2 Summary of findings table in technical level CNMA

Population: Children and adolescents with overweight or obesity

Settings: Any

Intervention: Technical-level treatment components of cognitive behavioural therapy

Comparison: Minimal education

Treatment components	Outcomes (follow up)	Number of participants (studies) from direct evidence	Number of participants (studies) treated with the specific component	Certainty of the evidence (GRADE)	Anticipated effects (95% CI)	
					Risk with minimal education alone	Risk with target treatment
Cognitive restructuring	**BMI z-score** (assessed at the end of treatment)	0 (0 study)	544 (10 studies)	⊕⊕⊝⊝ Low[a]	The mean BMI z-score at the end of the treatment ranged from 1.34 to 4.2.	0.00 lower BMI z-score (0.11 lower to 0.10 higher)
	**Body fat (%)** (assessed at the end of treatment)	0 (0 study)	272 (5 studies)	⊕⊕⊕⊝ Moderate[a]	The mean fat mass at the end of the treatment ranged from 26.2% to 47.9%.	3.67 lower fat mass % (7.94 lower to 0.61 higher)
	**Waist circumference (cm)** (assessed at the end of treatment)	0 (0 study)	642 (8 studies)	⊕⊕⊝⊝ Low[a]	The mean waist circumference at the end of the treatment ranged from 66 cm to 115.2 cm.	3.80 higher waist circumference in cm (0.09 lower to 7.68 higher)
	**Height (cm)** (assessed at the end of treatment)	0 (0 study)	211 (3 studies)	⊕⊕⊝⊝ Low	The mean height at the end of the treatment ranged from 121.5 cm to 171.7 cm.	5.59 lower height in cm (9.78 lower to 1.40 lower)
	**Quality-of-life score** (assessed at the end of treatment)	0 (0 study)	55 (1 studies)	⊕⊝⊝⊝ Very low[a]	The mean quality-of-life score at the end of the treatment ranged from 63.7 to 86.6.	0.29 higher quality-of-life score (0.23 lower to 0.80 higher)
	**Mental health score** (assessed at the end of treatment)	0 (0 study)	222 (3 studies)	⊕⊕⊝⊝ Low[a]	The mean mental health score at the end of the treatment ranged from 5.7 to 6.67.	0.05 lower mental health score (0.29 lower to 0.18 higher)
Contracting	**BMI z-score** (assessed at the end of treatment)	0 (0 study)	585 (12 studies)	⊕⊕⊕⊝ Moderate[a]	The mean BMI z-score at the end of the treatment ranged from 1.34 to 4.2.	0.05 lower BMI z-score (0.12 lower to 0.03 higher)
	**Body fat (%)** (assessed at the end of treatment)	0 (0 study)	121 (4 studies)	⊕⊕⊝⊝ Low[a]	The mean fat mass at the end of the treatment ranged from 26.2% to 47.9%.	1.97 higher fat mass % (2.55 lower to 6.49 higher)
	**Waist circumference (cm)** (assessed at the end of treatment)	0 (0 study)	251 (5 studies)	⊕⊝⊝⊝ Very low[a]	The mean waist circumference at the end of the treatment ranged from 66 cm to 115.2 cm.	0.11 higher waist circumference in cm (3.60 lower to 3.83 higher)
	**Height (cm)** (assessed at the end of treatment)	0 (0 study)	100 (3 studies)	⊕⊕⊝⊝ Low[a]	The mean height at the end of the treatment ranged from 121.5 cm to 171.7 cm.	1.22 lower height in cm (3.75 lower to 1.30 higher)

Treatment components	Outcomes (follow up)	Number of participants (studies) from direct evidence	Number of participants (studies) treated with the specific component	Certainty of the evidence (GRADE)	Anticipated effects (95% CI)	
					Risk with minimal education alone	Risk with target treatment
	**Quality-of-life score** (assessed at the end of treatment)	0 (0 study)	260 (3 studies)	⊕⊝⊝⊝ Very low[a]	The mean quality-of-life score at the end of the treatment ranged from 63.7 to 86.6.	0.20 lower quality-of-life score (1.12 lower to 0.72 higher)
**Device-monitoring**	**BMI z-score** (assessed at the end of treatment)	0 (0 study)	1167 (18 studies)	⊕⊕⊝⊝ Low[a]	The mean BMI z-score at the end of the treatment ranged from 1.34 to 4.2.	0.00 higher BMI z-score (0.06 lower to 0.07 higher)
	**Body fat (%)** (assessed at the end of treatment)	0 (0 study)	502 (7 studies)	⊕⊕⊕⊝ Moderate	The mean fat mass at the end of the treatment ranged from 26.2% to 47.9%.	3.69 higher fat mass % (0.56 higher to 6.83 higher)
	**Waist circumference (cm)** (assessed at the end of treatment)	0 (0 study)	638 (8 studies)	⊕⊕⊝⊝ Low[a]	The mean waist circumference at the end of the treatment ranged from 66 cm to 115.2 cm.	1.48 higher waist circumference in cm (2.16 lower to 5.11 higher)
	**Height (cm)** (assessed at the end of treatment)	0 (0 study)	493 (8 studies)	⊕⊕⊝⊝ Low	The mean height at the end of the treatment ranged from 121.5 cm to 171.7 cm.	1.46 higher height in cm (0.20 higher to 2.72 higher)
	**Quality-of-life score** (assessed at the end of treatment)	0 (0 study)	402 (5 studies)	⊕⊕⊝⊝ Low[a]	The mean quality-of-life score at the end of the treatment ranged from 63.7 to 86.6.	0.15 higher quality-of-life score (0.17 lower to 0.48 higher)
	**Mental health score** (assessed at the end of treatment)	0 (0 study)	151 (2 studies)	⊕⊕⊝⊝ Low[a]	The mean mental health score at the end of the treatment ranged from 5.7 to 6.67.	0.07 lower mental health score (0.30 lower to 0.17 higher)
**Feedback**	**BMI z-score** (assessed at the end of treatment)	0 (0 study)	3594 (45 studies)	⊕⊕⊝⊝ Low[a]	The mean BMI z-score at the end of the treatment ranged from 1.34 to 4.2.	0.02 higher BMI z-score (0.02 lower to 0.07 higher)
	**Body fat (%)** (assessed at the end of treatment)	0 (0 study)	919 (12 studies)	⊕⊕⊕⊝ Moderate	The mean fat mass at the end of the treatment ranged from 26.2% to 47.9%.	2.73 lower fat mass % (5.31 lower to 0.14 lower)
	**Waist circumference (cm)** (assessed at the end of treatment)	0 (0 study)	1125 (15 studies)	⊕⊕⊝⊝ Low[a]	The mean waist circumference at the end of the treatment ranged from 66 cm to 115.2 cm.	1.15 higher waist circumference in cm (1.11 lower to 3.41 higher)
	**Height (cm)** (assessed at the end of treatment)	0 (0 study)	493 (8 studies)	⊕⊕⊝⊝ Low[a]	The mean height at the end of the treatment ranged from 121.5 cm to 171.7 cm.	0.26 lower height in cm (1.59 lower to 1.08 higher)

Treatment components	Outcomes (follow up)	Number of participants (studies) from direct evidence	Number of participants (studies) treated with the specific component	Certainty of the evidence (GRADE)	Anticipated effects (95% CI)	
					Risk with minimal education alone	Risk with target treatment
	**Quality-of-life score** (assessed at the end of treatment)	0 (0 study)	936 (9 studies)	⊕⊕⊖⊖ Low	The mean quality-of-life score at the end of the treatment ranged from 63.7 to 86.6.	0.77 lower quality-of-life score (1.34 lower to 0.20 lower)
	**Mental health score** (assessed at the end of treatment)	0 (0 study)	348 (3 studies)	⊕⊕⊕⊖ Moderate[a]	The mean mental health score at the end of the treatment ranged from 5.7 to 6.67.	0.04 lower mental health score (0.24 lower to 0.17 higher)
**Functional behavioural analysis**	**BMI z-score** (assessed at the end of treatment)	0 (0 study)	989 (17 studies)	⊕⊕⊖⊖ Low[a]	The mean BMI z-score at the end of the treatment ranged from 1.34 to 4.2.	0.02 higher BMI z-score (0.05 lower to 0.09 higher)
	**Body fat (%)** (assessed at the end of treatment)	0 (0 study)	364 (6 studies)	⊕⊕⊖⊖ Low[a]	The mean fat mass at the end of the treatment ranged from 26.2% to 47.9%.	1.42 higher fat mass % (1.14 lower to 3.97 higher)
	**Waist circumference (cm)** (assessed at the end of treatment)	0 (0 study)	486 (8 studies)	⊕⊕⊖⊖ Low[a]	The mean waist circumference at the end of the treatment ranged from 66 cm to 115.2 cm.	1.89 lower waist circumference in cm (4.80 lower to 1.03 higher)
	**Quality-of-life score** (assessed at the end of treatment)	0 (0 study)	206 (3 studies)	⊕⊖⊖⊖ Very low[a]	The mean quality-of-life score at the end of the treatment ranged from 63.7 to 86.6.	0.00 lower quality-of-life score (0.35 lower to 0.35 higher)
	**Mental health score** (assessed at the end of treatment)	0 (0 study)	114 (3 studies)	⊕⊕⊖⊖ Low[a]	The mean mental health score at the end of the treatment ranged from 5.7 to 6.67.	0.06 lower mental health score (0.26 lower to 0.14 higher)
**Goal-setting**	**BMI z-score** (assessed at the end of treatment)	0 (0 study)	6462 (74 studies)	⊕⊕⊖⊖ Low[a]	The mean BMI z-score at the end of the treatment ranged from 1.34 to 4.2.	0.00 higher BMI z-score (0.06 lower to 0.06 higher)
	**Body fat (%)** (assessed at the end of treatment)	0 (0 study)	1623 (18 studies)	⊕⊕⊕⊖ Moderate[a]	The mean fat mass at the end of the treatment ranged from 26.2% to 47.9%.	0.26 higher fat mass % (1.99 lower to 2.50 higher)
	**Waist circumference (cm)** (assessed at the end of treatment)	0 (0 study)	2423 (24 studies)	⊕⊕⊖⊖ Low[a]	The mean waist circumference at the end of the treatment ranged from 66 cm to 115.2 cm.	1.98 lower waist circumference in cm (4.64 lower to 0.68 higher)
	**Height (cm)** (assessed at the end of treatment)	0 (0 study)	1485 (17 studies)	⊕⊕⊖⊖ Low[a]	The mean height at the end of the treatment ranged from 121.5 cm to 171.7 cm.	0.45 lower height in cm (1.82 lower to 0.92 higher)

Treatment components	Outcomes (follow up)	Number of participants (studies) from direct evidence	Number of participants (studies) treated with the specific component	Certainty of the evidence (GRADE)	Anticipated effects (95% CI)	
					Risk with minimal education alone	Risk with target treatment
	**Quality-of-life score** (assessed at the end of treatment)	0 (0 study)	1512 (13 studies)	⊕⊖ ⊖ ⊖ Very low[a]	The mean quality-of-life score at the end of the treatment ranged from 63.7 to 86.6.	0.27 higher quality-of-life score (0.58 lower to 1.13 higher)
	**Mental health score** (assessed at the end of treatment)	0 (0 study)	463 (4 studies)	⊕⊖ ⊖ ⊖ Very low[a]	The mean mental health score at the end of the treatment ranged from 5.7 to 6.67.	0.16 lower mental health score (0.37 lower to 0.05 higher)
Group	**BMI z-score** (assessed at the end of treatment)	0 (0 study)	4427 (57 studies)	⊕ ⊕⊖ ⊖ Low[a]	The mean BMI z-score at the end of the treatment ranged from 1.34 to 4.2.	0.00 lower BMI z-score (0.04 lower to 0.04 higher)
Group	**Body fat (%)** (assessed at the end of treatment)	0 (0 study)	1620 (22 studies)	⊕ ⊕ ⊕⊖ Moderate[a]	The mean fat mass at the end of the treatment ranged from 26.2% to 47.9%.	1.09 higher fat mass % (0.58 lower to 2.76 higher)
Group	**Waist circumference (cm)** (assessed at the end of treatment)	0 (0 study)	2258 (28 studies)	⊕ ⊕⊖ ⊖ Low[a]	The mean waist circumference at the end of the treatment ranged from 66 cm to 115.2 cm.	2.02 lower waist circumference in cm (4.25 lower to 0.20 higher)
Group	**Height (cm)** (assessed at the end of treatment)	0 (0 study)	1332 (19 studies)	⊕ ⊕⊖ ⊖ Low[a]	The mean height at the end of the treatment ranged from 121.5 cm to 171.7 cm.	1.28 higher height in cm (0.19 lower to 2.75 higher)
Group	**Quality-of-life score** (assessed at the end of treatment)	0 (0 study)	879 (13 studies)	⊕⊖ ⊖ ⊖ Very low[a]	The mean quality-of-life score at the end of the treatment ranged from 63.7 to 86.6.	0.25 lower quality-of-life score (0.56 lower to 0.06 higher)
Group	**Mental health score** (assessed at the end of treatment)	0 (0 study)	737 (7 studies)	⊕⊖ ⊖ ⊖ Very low[a]	The mean mental health score at the end of the treatment ranged from 5.7 to 6.67.	0.07 lower mental health score (0.49 lower to 0.36 higher)
Inhibition training	**BMI z-score** (assessed at the end of treatment)	0 (0 study)	377 (6 studies)	⊕⊖ ⊖ ⊖ Very low[a]	The mean BMI z-score at the end of the treatment ranged from 1.34 to 4.2.	0.04 higher BMI z-score (0.13 lower to 0.20 higher)
Inhibition training	**Waist circumference (cm)** (assessed at the end of treatment)	0 (0 study)	47 (1 studies)	⊕ ⊕⊖ ⊖ Low[a]	The mean waist circumference at the end of the treatment ranged from 66 cm to 115.2 cm.	1.48 lower waist circumference in cm (7.97 lower to 5.00 higher)
Inhibition training	**Quality-of-life score** (assessed at the end of treatment)	0 (0 study)	181 (2 studies)	⊕⊖ ⊖ ⊖ Very low[a]	The mean quality-of-life score at the end of the treatment ranged from 63.7 to 86.6.	0.33 higher quality-of-life score (0.49 lower to 1.16 higher)

Treatment components	Outcomes (follow up)	Number of participants (studies) from direct evidence	Number of participants (studies) treated with the specific component	Certainty of the evidence (GRADE)	Anticipated effects (95% CI)	
					Risk with minimal education alone	Risk with target treatment
Modelling	**BMI z-score** (assessed at the end of treatment)	0 (0 study)	2633 (34 studies)	⊕⊕⊝⊝ Low[a]	The mean BMI z-score at the end of the treatment ranged from 1.34 to 4.2.	0.01 higher BMI z-score (0.05 lower to 0.07 higher)
	**Body fat (%)** (assessed at the end of treatment)	0 (0 study)	476 (6 studies)	⊕⊕⊝⊝ Low[a]	The mean fat mass at the end of the treatment ranged from 26.2% to 47.9%.	0.25 higher fat mass % (3.04 lower to 3.55 higher)
	**Waist circumference (cm)** (assessed at the end of treatment)	0 (0 study)	388 (5 studies)	⊕⊕⊝⊝ Low[a]	The mean waist circumference at the end of the treatment ranged from 66 cm to 115.2 cm.	1.20 higher waist circumference in cm (2.28 lower to 4.69 higher)
	**Height (cm)** (assessed at the end of treatment)	0 (0 study)	801 (10 studies)	⊕⊝⊝⊝ Very low[a]	The mean height at the end of the treatment ranged from 121.5 cm to 171.7 cm.	0.90 higher height in cm (1.30 lower to 3.11 higher)
	**Quality-of-life score** (assessed at the end of treatment)	0 (0 study)	381 (6 studies)	⊕⊝⊝⊝ Very low[a]	The mean quality-of-life score at the end of the treatment ranged from 63.7 to 86.6.	0.45 lower quality-of-life score (1.30 lower to 0.41 higher)
	**Mental health score** (assessed at the end of treatment)	0 (0 study)	191 (2 studies)	⊕⊝⊝⊝ Very low[a]	The mean mental health score at the end of the treatment ranged from 5.7 to 6.67.	0.20 lower mental health score (0.45 lower to 0.06 higher)
Motivation	**BMI z-score** (assessed at the end of treatment)	0 (0 study)	3647 (41 studies)	⊕⊕⊝⊝ Low[a]	The mean BMI z-score at the end of the treatment ranged from 1.34 to 4.2.	0.03 lower BMI z-score (0.07 lower to 0.02 higher)
	**Body fat (%)** (assessed at the end of treatment)	0 (0 study)	1158 (17 studies)	⊕⊕⊝⊝ Low[a]	The mean fat mass at the end of the treatment ranged from 26.2% to 47.9%.	1.06 lower fat mass % (2.70 lower to 0.57 higher)
	**Waist circumference (cm)** (assessed at the end of treatment)	0 (0 study)	2002 (27 studies)	⊕⊕⊝⊝ Low[a]	The mean waist circumference at the end of the treatment ranged from 66 cm to 115.2 cm.	1.39 lower waist circumference in cm (3.47 lower to 0.69 higher)
	**Height (cm)** (assessed at the end of treatment)	0 (0 study)	367 (7 studies)	⊕⊝⊝⊝ Very low[a]	The mean height at the end of the treatment ranged from 121.5 cm to 171.7 cm.	0.42 lower height in cm (2.02 lower to 1.18 higher)
	**Quality-of-life score** (assessed at the end of treatment)	0 (0 study)	1070 (9 studies)	⊕⊝⊝⊝ Very low	The mean quality-of-life score at the end of the treatment ranged from 63.7 to 86.6.	0.57 higher quality-of-life score (0.16 higher to 0.98 higher)

Treatment components	Outcomes (follow up)	Number of participants (studies) from direct evidence	Number of participants (studies) treated with the specific component	Certainty of the evidence (GRADE)	Anticipated effects (95% CI)	
					Risk with minimal education alone	Risk with target treatment
	**Mental health score** (assessed at the end of treatment)	0 (0 study)	329 (4 studies)	⊕⊕⊝⊝ Low[a]	The mean mental health score at the end of the treatment ranged from 5.7 to 6.67.	0.07 lower mental health score (0.22 lower to 0.07 higher)
**Parental involvement**	**BMI z-score** (assessed at the end of treatment)	0 (0 study)	7953 (89 studies)	⊕⊕⊕⊝ Moderate	The mean BMI z-score at the end of the treatment ranged from 1.34 to 4.2.	0.09 lower BMI z-score (0.16 lower to 0.03 lower)
	**Body fat (%)** (assessed at the end of treatment)	0 (0 study)	1993 (28 studies)	⊕⊕⊕⊝ Moderate[a]	The mean fat mass at the end of the treatment ranged from 26.2% to 47.9%.	0.55 lower fat mass % (2.85 lower to 1.74 higher)
	**Waist circumference (cm)** (assessed at the end of treatment)	0 (0 study)	2760 (37 studies)	⊕⊕⊝⊝ Low[a]	The mean waist circumference at the end of the treatment ranged from 66 cm to 115.2 cm.	0.10 higher waist circumference in cm (2.13 lower to 2.32 higher)
	**Height (cm)** (assessed at the end of treatment)	0 (0 study)	1782 (25 studies)	⊕⊝⊝⊝ Very low[a]	The mean height at the end of the treatment ranged from 121.5 cm to 171.7 cm.	0.02 lower height in cm (0.55 lower to 0.50 higher)
	**Quality-of-life score** (assessed at the end of treatment)	0 (0 study)	1783 (17 studies)	⊕⊝⊝⊝ Very low[a]	The mean quality-of-life score at the end of the treatment ranged from 63.7 to 86.6.	0.09 higher quality-of-life score (0.17 lower to 0.34 higher)
	**Mental health score** (assessed at the end of treatment)	0 (0 study)	487 (6 studies)	⊕⊝⊝⊝ Very low[a]	The mean mental health score at the end of the treatment ranged from 5.7 to 6.67.	0.09 lower mental health score (0.46 lower to 0.28 higher)
**Preplanning**	**BMI z-score** (assessed at the end of treatment)	0 (0 study)	1779 (27 studies)	⊕⊕⊝⊝ Low[a]	The mean BMI z-score at the end of the treatment ranged from 1.34 to 4.2.	0.01 higher BMI z-score (0.05 lower to 0.07 higher)
	**Body fat (%)** (assessed at the end of treatment)	0 (0 study)	499 (8 studies)	⊕⊕⊕⊝ Moderate	The mean fat mass at the end of the treatment ranged from 26.2% to 47.9%.	3.05 lower fat mass % (5.82 lower to 0.28 lower)
	**Waist circumference (cm)** (assessed at the end of treatment)	0 (0 study)	615 (10 studies)	⊕⊕⊝⊝ Low[a]	The mean waist circumference at the end of the treatment ranged from 66 cm to 115.2 cm.	0.46 lower waist circumference in cm (3.57 lower to 2.65 higher)
	**Height (cm)** (assessed at the end of treatment)	0 (0 study)	413 (8 studies)	⊕⊕⊝⊝ Low[a]	The mean height at the end of the treatment ranged from 121.5 cm to 171.7 cm.	0.95 higher height in cm (1.01 lower to 2.91 higher)
	**Quality-of-life score** (assessed at the end of treatment)	0 (0 study)	125 (3 studies)	⊕⊝⊝⊝ Very low[a]	The mean quality-of-life score at the end of the treatment ranged from 63.7 to	0.20 lower quality-of-life score (1.05 lower to 0.64 higher)

Treatment components	Outcomes (follow up)	Number of participants (studies) from direct evidence	Number of participants (studies) treated with the specific component	Certainty of the evidence (GRADE)	Anticipated effects (95% CI)	
					Risk with minimal education alone	Risk with target treatment
					86.6.	
	**Mental health score** (assessed at the end of treatment)	0 (0 study)	214 (2 studies)	⊕⊖⊖⊖ Very low[a]	The mean mental health score at the end of the treatment ranged from 5.7 to 6.67.	0.01 lower mental health score (0.36 lower to 0.33 higher)
**Problem solving**	**BMI z-score** (assessed at the end of treatment)	0 (0 study)	3755 (49 studies)	⊕⊕⊖⊖ Low[a]	The mean BMI z-score at the end of the treatment ranged from 1.34 to 4.2.	0.00 lower BMI z-score (0.05 lower to 0.04 higher)
	**Body fat (%)** (assessed at the end of treatment)	0 (0 study)	1052 (20 studies)	⊕⊕⊕⊖ Moderate	The mean fat mass at the end of the treatment ranged from 26.2% to 47.9%.	2.90 higher fat mass % (0.30 higher to 5.50 higher)
	**Waist circumference (cm)** (assessed at the end of treatment)	0 (0 study)	1735 (25 studies)	⊕⊕⊖⊖ Low[a]	The mean waist circumference at the end of the treatment ranged from 66 cm to 115.2 cm.	1.18 lower waist circumference in cm (3.78 lower to 1.42 higher)
	**Height (cm)** (assessed at the end of treatment)	0 (0 study)	773 (16 studies)	⊕⊕⊖⊖ Low[a]	The mean height at the end of the treatment ranged from 121.5 cm to 171.7 cm.	0.38 lower height in cm (1.51 lower to 0.75 higher)
	**Quality-of-life score** (assessed at the end of treatment)	0 (0 study)	891 (10 studies)	⊕⊖⊖⊖ Very low[a]	The mean quality-of-life score at the end of the treatment ranged from 63.7 to 86.6.	0.15 lower quality-of-life score (0.71 lower to 0.40 higher)
	**Mental health score** (assessed at the end of treatment)	0 (0 study)	355 (5 studies)	⊕⊕⊖⊖ Low[a]	The mean mental health score at the end of the treatment ranged from 5.7 to 6.67.	0.34 higher mental health score (0.22 lower to 0.90 higher)
**Psychoeducation**	**BMI z-score** (assessed at the end of treatment)	0 (0 study)	8766 (95 studies)	⊕⊕⊖⊖ Low[a]	The mean BMI z-score at the end of the treatment ranged from 1.34 to 4.2.	0.02 higher BMI z-score (0.05 lower to 0.10 higher)
	**Body fat (%)** (assessed at the end of treatment)	0 (0 study)	2823 (35 studies)	⊕⊕⊕⊖ Moderate[a]	The mean fat mass at the end of the treatment ranged from 26.2% to 47.9%.	1.09 lower fat mass % (3.56 lower to 1.38 higher)
	**Waist circumference (cm)** (assessed at the end of treatment)	0 (0 study)	3544 (43 studies)	⊕⊕⊖⊖ Low[a]	The mean waist circumference at the end of the treatment ranged from 66 cm to 115.2 cm.	1.18 higher waist circumference in cm (1.89 lower to 4.25 higher)
	**Height (cm)** (assessed at the end of treatment)	0 (0 study)	1934 (26 studies)	⊕⊖⊖⊖ Very low[a]	The mean height at the end of the treatment ranged from 121.5 cm to 171.7 cm.	0.94 higher height in cm (1.23 lower to 3.12 higher)

Treatments components	Outcomes (follow up)	Number of participants (studies) from direct evidence	Number of participants (studies) treated with the specific component	Certainty of the evidence (GRADE)	Anticipated effects (95% CI)	
					Risk with minimal education alone	Risk with target treatment
	**Quality-of-life score** (assessed at the end of treatment)	0 (0 study)	1993 (18 studies)	⊕⊖⊖⊖ Very low[a]	The mean quality-of-life score at the end of the treatment ranged from 63.7 to 86.6.	0.20 lower quality-of-life score (0.77 lower to 0.37 higher)
	**Mental health score** (assessed at the end of treatment)	0 (0 study)	769 (8 studies)	⊕⊖⊖⊖ Very low[a]	The mean mental health score at the end of the treatment ranged from 5.7 to 6.67.	0.09 higher mental health score (0.26 lower to 0.44 higher)
**Reinforcement**	**BMI z-score** (assessed at the end of treatment)	0 (0 study)	3776 (46 studies)	⊕⊕⊖⊖ Low[a]	The mean BMI z-score at the end of the treatment ranged from 1.34 to 4.2.	0.04 lower BMI z-score (0.10 lower to 0.02 higher)
	**Body fat (%)** (assessed at the end of treatment)	0 (0 study)	941 (13 studies)	⊕⊕⊖⊖ Low[a]	The mean fat mass at the end of the treatment ranged from 26.2% to 47.9%.	1.25 lower fat mass % (3.76 lower to 1.25 higher)
	**Waist circumference (cm)** (assessed at the end of treatment)	0 (0 study)	1218 (15 studies)	⊕⊕⊖⊖ Low[a]	The mean waist circumference at the end of the treatment ranged from 66 cm to 115.2 cm.	1.43 higher waist circumference in cm (1.93 lower to 4.80 higher)
	**Height (cm)** (assessed at the end of treatment)	0 (0 study)	1102 (16 studies)	⊕⊖⊖⊖ Very low[a]	The mean height at the end of the treatment ranged from 121.5 cm to 171.7 cm.	1.17 lower height in cm (2.37 lower to 0.04 higher)
	**Quality-of-life score** (assessed at the end of treatment)	0 (0 study)	751 (8 studies)	⊕⊖⊖⊖ Very low[a]	The mean quality-of-life score at the end of the treatment ranged from 63.7 to 86.6.	0.28 higher quality-of-life score (0.43 lower to 1.00 higher)
	**Mental health score** (assessed at the end of treatment)	0 (0 study)	312 (4 studies)	⊕⊖⊖⊖ Very low[a]	The mean mental health score at the end of the treatment ranged from 5.7 to 6.67.	0.25 lower mental health score (0.61 lower to 0.12 higher)
**Relaxation training**	**BMI z-score** (assessed at the end of treatment)	0 (0 study)	68 (4 studies)	⊕⊕⊕⊖ Moderate	The mean BMI z-score at the end of the treatment ranged from 1.34 to 4.2.	0.16 higher BMI z-score (0.00 lower to 0.32 higher)
	**Body fat (%)** (assessed at the end of treatment)	0 (0 study)	52 (3 studies)	⊕⊕⊕⊖ Moderate	The mean fat mass at the end of the treatment ranged from 26.2% to 47.9%.	8.08 higher fat mass % (0.82 higher to 15.34 higher)
	**Waist circumference (cm)** (assessed at the end of treatment)	0 (0 study)	52 (3 studies)	⊕⊕⊖⊖ Low[a]	The mean waist circumference at the end of the treatment ranged from 66 cm to 115.2 cm.	5.50 lower waist circumference in cm (12.46 lower to 1.46 higher)

Treatment components	Outcomes (follow up)	Number of participants (studies) from direct evidence	Number of participants (studies) treated with the specific component	Certainty of the evidence (GRADE)	Anticipated effects (95% CI)	
					Risk with minimal education alone	Risk with target treatment
	**Quality-of-life score** (assessed at the end of treatment)	0 (0 study)	10 (1 studies)	⊕⊖ ⊖ ⊖ Very low[a]	The mean quality-of-life score at the end of the treatment ranged from 63.7 to 86.6.	0.22 lower quality-of-life score (0.73 lower to 0.29 higher)
	**Mental health score** (assessed at the end of treatment)	0 (0 study)	34 (2 studies)	⊕ ⊕ ⊕⊖ Moderate[a]	The mean mental health score at the end of the treatment ranged from 5.7 to 6.67.	0.18 lower mental health score (0.46 lower to 0.11 higher)
Reminders	**BMI z-score** (assessed at the end of treatment)	0 (0 study)	851 (12 studies)	⊕ ⊕⊖ ⊖ Low[a]	The mean BMI z-score at the end of the treatment ranged from 1.34 to 4.2.	0.02 higher BMI z-score (0.04 lower to 0.08 higher)
Reminders	**Body fat (%)** (assessed at the end of treatment)	0 (0 study)	119 (3 studies)	⊕ ⊕ ⊕⊖ Moderate[a]	The mean fat mass at the end of the treatment ranged from 26.2% to 47.9%.	1.44 higher fat mass % (1.38 lower to 4.26 higher)
Reminders	**Waist circumference (cm)** (assessed at the end of treatment)	0 (0 study)	185 (4 studies)	⊕ ⊕⊖ ⊖ Low[a]	The mean waist circumference at the end of the treatment ranged from 66 cm to 115.2 cm.	1.80 lower waist circumference in cm (5.24 lower to 1.64 higher)
Reminders	**Height (cm)** (assessed at the end of treatment)	0 (0 study)	77 (2 studies)	⊕⊖ ⊖ ⊖ Very low[a]	The mean height at the end of the treatment ranged from 121.5 cm to 171.7 cm.	1.31 higher height in cm (0.36 lower to 2.99 higher)
Reminders	**Quality-of-life score** (assessed at the end of treatment)	0 (0 study)	123 (2 studies)	⊕⊖ ⊖ ⊖ Very low[a]	The mean quality-of-life score at the end of the treatment ranged from 63.7 to 86.6.	0.40 lower quality-of-life score (1.03 lower to 0.24 higher)
Reminders	**Mental health score** (assessed at the end of treatment)	0 (0 study)	66 (2 studies)	⊕⊖ ⊖ ⊖ Very low[a]	The mean mental health score at the end of the treatment ranged from 5.7 to 6.67.	0.03 higher mental health score (0.20 lower to 0.26 higher)
Remote	**BMI z-score** (assessed at the end of treatment)	0 (0 study)	1960 (26 studies)	⊕ ⊕⊖ ⊖ Low[a]	The mean BMI z-score at the end of the treatment ranged from 1.34 to 4.2.	0.03 lower BMI z-score (0.09 lower to 0.03 higher)
Remote	**Body fat (%)** (assessed at the end of treatment)	0 (0 study)	920 (13 studies)	⊕ ⊕⊖ ⊖ Low[a]	The mean fat mass at the end of the treatment ranged from 26.2% to 47.9%.	1.80 lower fat mass % (3.94 lower to 0.33 higher)
Remote	**Waist circumference (cm)** (assessed at the end of treatment)	0 (0 study)	943 (12 studies)	⊕ ⊕⊖ ⊖ Low[a]	The mean waist circumference at the end of the treatment ranged from 66 cm to 115.2 cm.	0.11 lower waist circumference in cm (2.63 lower to 2.42 higher)
Remote	**Height (cm)** (assessed at the end of	0 (0 study)	406 (9 studies)	⊕ ⊕⊖ ⊖ Low[a]	The mean height at the end of the treatment ranged from	0.23 lower height in cm (1.39 lower to 0.93 higher)

Treatment components	Outcomes (follow up)	Number of participants (studies) from direct evidence	Number of participants (studies) treated with the specific component	Certainty of the evidence (GRADE)	Anticipated effects (95% CI)	
					Risk with minimal education alone	Risk with target treatment
	treatment)				121.5 cm to 171.7 cm.	
	**Quality-of-life score** (assessed at the end of treatment)	0 (0 study)	579 (4 studies)	⊕⊖ ⊖ ⊖ Very low[a]	The mean quality-of-life score at the end of the treatment ranged from 63.7 to 86.6.	0.28 higher quality-of-life score (0.24 lower to 0.80 higher)
	**Mental health score** (assessed at the end of treatment)	0 (0 study)	277 (4 studies)	⊕⊖ ⊖ ⊖ Very low[a]	The mean mental health score at the end of the treatment ranged from 5.7 to 6.67.	0.06 lower mental health score (0.37 lower to 0.25 higher)
**Role playing**	**BMI z-score** (assessed at the end of treatment)	0 (0 study)	375 (5 studies)	⊕ ⊕⊖ ⊖ Low[a]	The mean BMI z-score at the end of the treatment ranged from 1.34 to 4.2.	0.00 higher BMI z-score (0.12 lower to 0.13 higher)
	**Body fat (%)** (assessed at the end of treatment)	0 (0 study)	169 (2 studies)	⊕⊖ ⊖ ⊖ Very low[a]	The mean fat mass at the end of the treatment ranged from 26.2% to 47.9%.	0.78 higher fat mass % (5.48 lower to 7.05 higher)
	**Waist circumference (cm)** (assessed at the end of treatment)	0 (0 study)	103 (2 studies)	⊕⊖ ⊖ ⊖ Very low[a]	The mean waist circumference at the end of the treatment ranged from 66 cm to 115.2 cm.	3.71 lower waist circumference in cm (11.02 lower to 3.61 higher)
	**Height (cm)** (assessed at the end of treatment)	0 (0 study)	138 (2 studies)	⊕ ⊕⊖ ⊖ Low[a]	The mean height at the end of the treatment ranged from 121.5 cm to 171.7 cm.	3.02 lower height in cm (6.14 lower to 0.09 higher)
	**Quality-of-life score** (assessed at the end of treatment)	0 (0 study)	206 (2 studies)	⊕ ⊕⊖ ⊖ Low	The mean quality-of-life score at the end of the treatment ranged from 63.7 to 86.6.	0.76 lower quality-of-life score (1.50 lower to 0.03 lower)
	**Mental health score** (assessed at the end of treatment)	0 (0 study)	112 (1 studies)	⊕ ⊕⊖ ⊖ Low[a]	The mean mental health score at the end of the treatment ranged from 5.7 to 6.67.	0.10 higher mental health score (0.14 lower to 0.34 higher)
**Rule-setting**	**BMI z-score** (assessed at the end of treatment)	0 (0 study)	964 (16 studies)	⊕ ⊕⊖ ⊖ Low[a]	The mean BMI z-score at the end of the treatment ranged from 1.34 to 4.2.	0.00 higher BMI z-score (0.08 lower to 0.09 higher)
	**Body fat (%)** (assessed at the end of treatment)	0 (0 study)	245 (2 studies)	⊕ ⊕ ⊕⊖ Moderate	The mean fat mass at the end of the treatment ranged from 26.2% to 47.9%.	4.32 higher fat mass % (0.16 higher to 8.47 higher)
	**Waist circumference (cm)** (assessed at the end of treatment)	0 (0 study)	482 (5 studies)	⊕⊖ ⊖ ⊖ Very low[a]	The mean waist circumference at the end of the treatment ranged from 66 cm to 115.2 cm.	0.95 lower waist circumference in cm (7.26 lower to 5.36 higher)

Treatment components	Outcomes (follow up)	Number of participants (studies) from direct evidence	Number of participants (studies) treated with the specific component	Certainty of the evidence (GRADE)	Anticipated effects (95% CI)	
					Risk with minimal education alone	Risk with target treatment
	**Height (cm)** (assessed at the end of treatment)	0 (0 study)	175 (3 studies)	⊕⊖⊖⊖ Very low[a]	The mean height at the end of the treatment ranged from 121.5 cm to 171.7 cm.	1.17 higher height in cm (0.93 lower to 3.27 higher)
	**Quality-of-life score** (assessed at the end of treatment)	0 (0 study)	54 (2 studies)	⊕⊖⊖⊖ Very low[a]	The mean quality-of-life score at the end of the treatment ranged from 63.7 to 86.6.	0.56 higher quality-of-life score (0.42 lower to 1.53 higher)
**Self-concept improvement**	**BMI z-score** (assessed at the end of treatment)	0 (0 study)	2366 (34 studies)	⊕⊕⊖⊖ Low[a]	The mean BMI z-score at the end of the treatment ranged from 1.34 to 4.2.	0.04 higher BMI z-score (0.01 lower to 0.10 higher)
	**Body fat (%)** (assessed at the end of treatment)	0 (0 study)	605 (7 studies)	⊕⊕⊕⊖ Moderate[a]	The mean fat mass at the end of the treatment ranged from 26.2% to 47.9%.	1.20 higher fat mass % (1.73 lower to 4.14 higher)
	**Waist circumference (cm)** (assessed at the end of treatment)	0 (0 study)	1230 (12 studies)	⊕⊕⊖⊖ Low[a]	The mean waist circumference at the end of the treatment ranged from 66 cm to 115.2 cm.	1.99 lower waist circumference in cm (4.42 lower to 0.45 higher)
	**Height (cm)** (assessed at the end of treatment)	0 (0 study)	455 (5 studies)	⊕⊕⊖⊖ Low[a]	The mean height at the end of the treatment ranged from 121.5 cm to 171.7 cm.	0.75 lower height in cm (3.65 lower to 2.14 higher)
	**Quality-of-life score** (assessed at the end of treatment)	0 (0 study)	508 (6 studies)	⊕⊖⊖⊖ Very low[a]	The mean quality-of-life score at the end of the treatment ranged from 63.7 to 86.6.	0.23 higher quality-of-life score (0.74 lower to 1.19 higher)
	**Mental health score** (assessed at the end of treatment)	0 (0 study)	260 (3 studies)	⊕⊕⊖⊖ Low[a]	The mean mental health score at the end of the treatment ranged from 5.7 to 6.67.	0.11 higher mental health score (0.14 lower to 0.36 higher)
**Self-monitoring**	**BMI z-score** (assessed at the end of treatment)	0 (0 study)	3853 (50 studies)	⊕⊕⊖⊖ Low[a]	The mean BMI z-score at the end of the treatment ranged from 1.34 to 4.2.	0.02 higher BMI z-score (0.03 lower to 0.07 higher)
	**Body fat (%)** (assessed at the end of treatment)	0 (0 study)	916 (12 studies)	⊕⊕⊖⊖ Low[a]	The mean fat mass at the end of the treatment ranged from 26.2% to 47.9%.	2.51 lower fat mass % (5.96 lower to 0.95 higher)
	**Waist circumference (cm)** (assessed at the end of treatment)	0 (0 study)	987 (12 studies)	⊕⊕⊖⊖ Low[a]	The mean waist circumference at the end of the treatment ranged from 66 cm to 115.2 cm.	0.33 higher waist circumference in cm (2.61 lower to 3.28 higher)
	**Height (cm)** (assessed at the end of	0 (0 study)	1068 (15 studies)	⊕⊕⊖⊖ Low[a]	The mean height at the end of the treatment ranged from	0.18 lower height in cm (2.24 lower to 1.88 higher)

Treatment components	Outcomes (follow up)	Number of participants (studies) from direct evidence	Number of participants (studies) treated with the specific component	Certainty of the evidence (GRADE)	Anticipated effects (95% CI)	
					Risk with minimal education alone	Risk with target treatment
	treatment)				121.5 cm to 171.7 cm.	
	**Quality-of-life score** (assessed at the end of treatment)	0 (0 study)	568 (8 studies)	⊕⊖⊖⊖ Very low[a]	The mean quality-of-life score at the end of the treatment ranged from 63.7 to 86.6.	0.08 lower quality-of-life score (0.87 lower to 0.72 higher)
**Serious games**	**BMI z-score** (assessed at the end of treatment)	0 (0 study)	1137 (18 studies)	⊕⊕⊖⊖ Low[a]	The mean BMI z-score at the end of the treatment ranged from 1.34 to 4.2.	0.03 lower BMI z-score (0.09 lower to 0.03 higher)
	**Body fat (%)** (assessed at the end of treatment)	0 (0 study)	436 (9 studies)	⊕⊕⊖⊖ Low[a]	The mean fat mass at the end of the treatment ranged from 26.2% to 47.9%.	1.91 lower fat mass % (4.27 lower to 0.44 higher)
	**Waist circumference (cm)** (assessed at the end of treatment)	0 (0 study)	852 (12 studies)	⊕⊕⊖⊖ Low[a]	The mean waist circumference at the end of the treatment ranged from 66 cm to 115.2 cm.	1.56 higher waist circumference in cm (0.92 lower to 4.05 higher)
	**Height (cm)** (assessed at the end of treatment)	0 (0 study)	549 (7 studies)	⊕⊕⊖⊖ Low[a]	The mean height at the end of the treatment ranged from 121.5 cm to 171.7 cm.	0.37 lower height in cm (1.46 lower to 0.71 higher)
**Social support**	**BMI z-score** (assessed at the end of treatment)	0 (0 study)	2260 (33 studies)	⊕⊕⊖⊖ Low[a]	The mean BMI z-score at the end of the treatment ranged from 1.34 to 4.2.	0.02 lower BMI z-score (0.08 lower to 0.04 higher)
	**Body fat (%)** (assessed at the end of treatment)	0 (0 study)	708 (10 studies)	⊕⊕⊖⊖ Low[a]	The mean fat mass at the end of the treatment ranged from 26.2% to 47.9%.	0.14 lower fat mass % (3.15 lower to 2.87 higher)
	**Waist circumference (cm)** (assessed at the end of treatment)	0 (0 study)	1481 (20 studies)	⊕⊕⊖⊖ Low[a]	The mean waist circumference at the end of the treatment ranged from 66 cm to 115.2 cm.	2.03 higher waist circumference in cm (0.53 lower to 4.58 higher)
	**Height (cm)** (assessed at the end of treatment)	0 (0 study)	522 (7 studies)	⊕⊖⊖⊖ Very low[a]	The mean height at the end of the treatment ranged from 121.5 cm to 171.7 cm.	0.91 lower height in cm (2.86 lower to 1.04 higher)
	**Quality-of-life score** (assessed at the end of treatment)	0 (0 study)	744 (9 studies)	⊕⊖⊖⊖ Very low[a]	The mean quality-of-life score at the end of the treatment ranged from 63.7 to 86.6.	0.62 higher quality-of-life score (0.14 lower to 1.38 higher)
	**Mental health score** (assessed at the end of treatment)	0 (0 study)	423 (4 studies)	⊕⊖⊖⊖ Very low[a]	The mean mental health score at the end of the treatment ranged from 5.7 to 6.67.	0.03 higher mental health score (0.11 lower to 0.18 higher)

Treatment components	Outcomes (follow up)	Number of participants (studies) from direct evidence	Number of participants (studies) treated with the specific component	Certainty of the evidence (GRADE)	Anticipated effects (95% CI)	
					Risk with minimal education alone	Risk with target treatment
Stimulus control	**BMI z-score** (assessed at the end of treatment)	0 (0 study)	3487 (42 studies)	⊕⊕⊕⊝ Moderate	The mean BMI z-score at the end of the treatment ranged from 1.34 to 4.2.	0.07 lower BMI z-score (0.12 lower to 0.01 lower)
	**Body fat (%)** (assessed at the end of treatment)	0 (0 study)	1027 (15 studies)	⊕⊕⊝⊝ Low[a]	The mean fat mass at the end of the treatment ranged from 26.2% to 47.9%.	2.66 higher fat mass % (1.12 lower to 6.44 higher)
	**Waist circumference (cm)** (assessed at the end of treatment)	0 (0 study)	1176 (17 studies)	⊕⊕⊝⊝ Low[a]	The mean waist circumference at the end of the treatment ranged from 66 cm to 115.2 cm.	0.42 lower waist circumference in cm (3.04 lower to 2.20 higher)
	**Height (cm)** (assessed at the end of treatment)	0 (0 study)	1091 (15 studies)	⊕⊕⊝⊝ Low[a]	The mean height at the end of the treatment ranged from 121.5 cm to 171.7 cm.	0.60 lower height in cm (2.34 lower to 1.13 higher)
	**Quality-of-life score** (assessed at the end of treatment)	0 (0 study)	521 (8 studies)	⊕⊝⊝⊝ Very low[a]	The mean quality-of-life score at the end of the treatment ranged from 63.7 to 86.6.	0.39 higher quality-of-life score (0.89 lower to 1.67 higher)
	**Mental health score** (assessed at the end of treatment)	0 (0 study)	244 (3 studies)	⊕⊕⊝⊝ Low[a]	The mean mental health score at the end of the treatment ranged from 5.7 to 6.67.	0.07 lower mental health score (0.22 lower to 0.07 higher)
Stoplight approach	**BMI z-score** (assessed at the end of treatment)	0 (0 study)	1668 (25 studies)	⊕⊕⊝⊝ Low[a]	The mean BMI z-score at the end of the treatment ranged from 1.34 to 4.2.	0.05 higher BMI z-score (0.01 lower to 0.12 higher)
	**Body fat (%)** (assessed at the end of treatment)	0 (0 study)	224 (5 studies)	⊕⊕⊕⊝ Moderate[a]	The mean fat mass at the end of the treatment ranged from 26.2% to 47.9%.	1.38 lower fat mass % (5.62 lower to 2.86 higher)
	**Waist circumference (cm)** (assessed at the end of treatment)	0 (0 study)	350 (7 studies)	⊕⊕⊝⊝ Low[a]	The mean waist circumference at the end of the treatment ranged from 66 cm to 115.2 cm.	0.81 lower waist circumference in cm (4.40 lower to 2.78 higher)
	**Height (cm)** (assessed at the end of treatment)	0 (0 study)	342 (6 studies)	⊕⊕⊝⊝ Low[a]	The mean height at the end of the treatment ranged from 121.5 cm to 171.7 cm.	1.45 higher height in cm (0.71 lower to 3.61 higher)
	**Quality-of-life score** (assessed at the end of treatment)	0 (0 study)	445 (6 studies)	⊕⊝⊝⊝ Very low[a]	The mean quality-of-life score at the end of the treatment ranged from 63.7 to 86.6.	0.18 lower quality-of-life score (1.35 lower to 0.99 higher)

Treatment components	Outcomes (follow up)	Number of participants (studies) from direct evidence	Number of participants (studies) treated with the specific component	Certainty of the evidence (GRADE)	Anticipated effects (95% CI)	
					Risk with minimal education alone	Risk with target treatment
	**Mental health score** (assessed at the end of treatment)	0 (0 study)	160 (2 studies)	⊕⊕⊝⊝ Low[a]	The mean mental health score at the end of the treatment ranged from 5.7 to 6.67.	0.07 lower mental health score (0.30 lower to 0.17 higher)
**Stress management**	**BMI z-score** (assessed at the end of treatment)	0 (0 study)	1094 (13 studies)	⊕⊕⊝⊝ Low[a]	The mean BMI z-score at the end of the treatment ranged from 1.34 to 4.2.	0.03 higher BMI z-score (0.06 lower to 0.11 higher)
	**Body fat (%)** (assessed at the end of treatment)	0 (0 study)	400 (4 studies)	⊕⊕⊕⊝ Moderate[a]	The mean fat mass at the end of the treatment ranged from 26.2% to 47.9%.	2.70 higher fat mass % (0.63 lower to 6.03 higher)
	**Waist circumference (cm)** (assessed at the end of treatment)	0 (0 study)	926 (9 studies)	⊕⊕⊝⊝ Low[a]	The mean waist circumference at the end of the treatment ranged from 66 cm to 115.2 cm.	1.77 higher waist circumference in cm (1.90 lower to 5.44 higher)
	**Quality-of-life score** (assessed at the end of treatment)	0 (0 study)	62 (2 studies)	⊕⊝⊝⊝ Very low[a]	The mean quality-of-life score at the end of the treatment ranged from 63.7 to 86.6.	0.49 higher quality-of-life score (0.20 lower to 1.19 higher)
	**Mental health score** (assessed at the end of treatment)	0 (0 study)	279 (3 studies)	⊕⊕⊝⊝ Low[a]	The mean mental health score at the end of the treatment ranged from 5.7 to 6.67.	0.09 higher mental health score (0.13 lower to 0.31 higher)
**Task-setting**	**BMI z-score** (assessed at the end of treatment)	0 (0 study)	1393 (22 studies)	⊕⊕⊝⊝ Low[a]	The mean BMI z-score at the end of the treatment ranged from 1.34 to 4.2.	0.00 lower BMI z-score (0.06 lower to 0.05 higher)
	**Body fat (%)** (assessed at the end of treatment)	0 (0 study)	269 (5 studies)	⊕⊕⊝⊝ Low[a]	The mean fat mass at the end of the treatment ranged from 26.2% to 47.9%.	1.36 higher fat mass % (1.43 lower to 4.16 higher)
	**Waist circumference (cm)** (assessed at the end of treatment)	0 (0 study)	551 (8 studies)	⊕⊕⊝⊝ Low[a]	The mean waist circumference at the end of the treatment ranged from 66 cm to 115.2 cm.	2.24 lower waist circumference in cm (4.82 lower to 0.34 higher)
	**Height (cm)** (assessed at the end of treatment)	0 (0 study)	591 (7 studies)	⊕⊕⊝⊝ Low[a]	The mean height at the end of the treatment ranged from 121.5 cm to 171.7 cm.	1.31 lower height in cm (2.77 lower to 0.15 higher)
	**Quality-of-life score** (assessed at the end of treatment)	0 (0 study)	111 (2 studies)	⊕⊝⊝⊝ Very low[a]	The mean quality-of-life score at the end of the treatment ranged from 63.7 to 86.6.	0.13 lower quality-of-life score (0.66 lower to 0.39 higher)

Treatment components	Outcomes (follow up)	Number of participants (studies) from direct evidence	Number of participants (studies) treated with the specific component	Certainty of the evidence (GRADE)	Anticipated effects (95% CI)	
					Risk with minimal education alone	Risk with target treatment
	**Mental health score** (assessed at the end of treatment)	0 (0 study)	120 (1 studies)	⊕⊖⊖⊖ Very low[a]	The mean mental health score at the end of the treatment ranged from 5.7 to 6.67.	0.12 higher mental health score (0.14 lower to 0.39 higher)
**Third-wave components**	**BMI z-score** (assessed at the end of treatment)	0 (0 study)	351 (5 studies)	⊕⊕⊖⊖ Low[a]	The mean BMI z-score at the end of the treatment ranged from 1.34 to 4.2.	0.12 lower BMI z-score (0.26 lower to 0.01 higher)
	**Waist circumference (cm)** (assessed at the end of treatment)	0 (0 study)	103 (2 studies)	⊕⊖⊖⊖ Very low[a]	The mean waist circumference at the end of the treatment ranged from 66 cm to 115.2 cm.	3.20 lower waist circumference in cm (13.18 lower to 6.79 higher)
	**Mental health score** (assessed at the end of treatment)	0 (0 study)	57 (2 studies)	⊕⊕⊖⊖ Low[a]	The mean mental health score at the end of the treatment ranged from 5.7 to 6.67.	0.04 lower mental health score (0.33 lower to 0.25 higher)

[a] Downgraded by one level for imprecision.

NOTES: The downgrading reasons for risk of bias, heterogeneity, indirectness and publication bias were shown only for direct estimates due to the method limitation. The downgrading reasons due to inadditivity, intransitivity, incoherence, and imprecision were shown for all pooled estimates.

High certainty: we are very confident that the true effect lies close to that of the estimate of the effect.
Moderate certainty: we are moderately confident in the effect estimate: the true effect is likely to be close to the estimate of the effect, but there is a possibility that it is substantially different.
Low certainty: our confidence in the effect estimate is limited: the true effect may be substantially different from the estimate of the effect.
Very low certainty: we have very little confidence in the effect estimate: the true effect is likely to be substantially different from the estimate of effect

5.3 Percentage contribution matrix for conceptual level NMA

5.3.1 Contribution matrix of BMI z-score

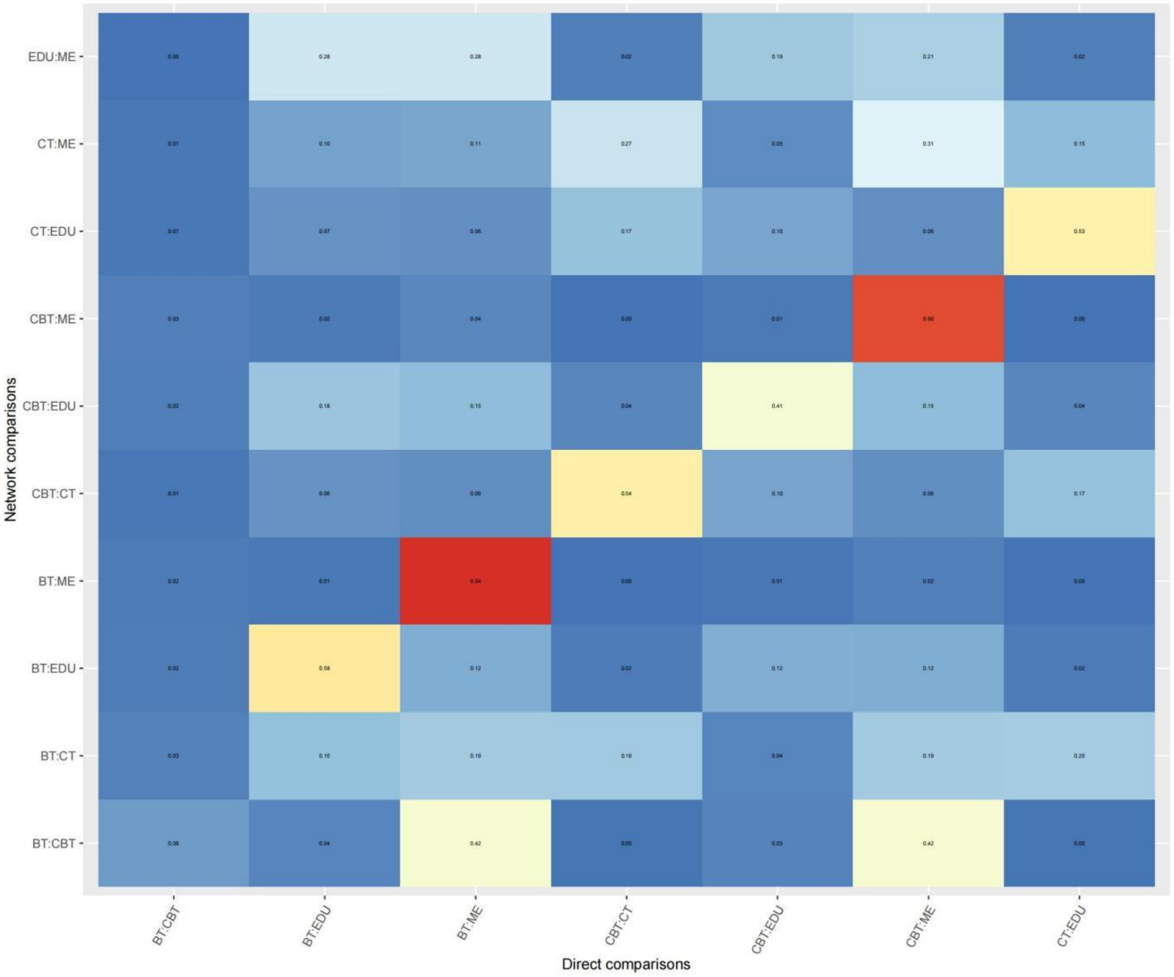

5.3.2 Contribution matrix of body fat

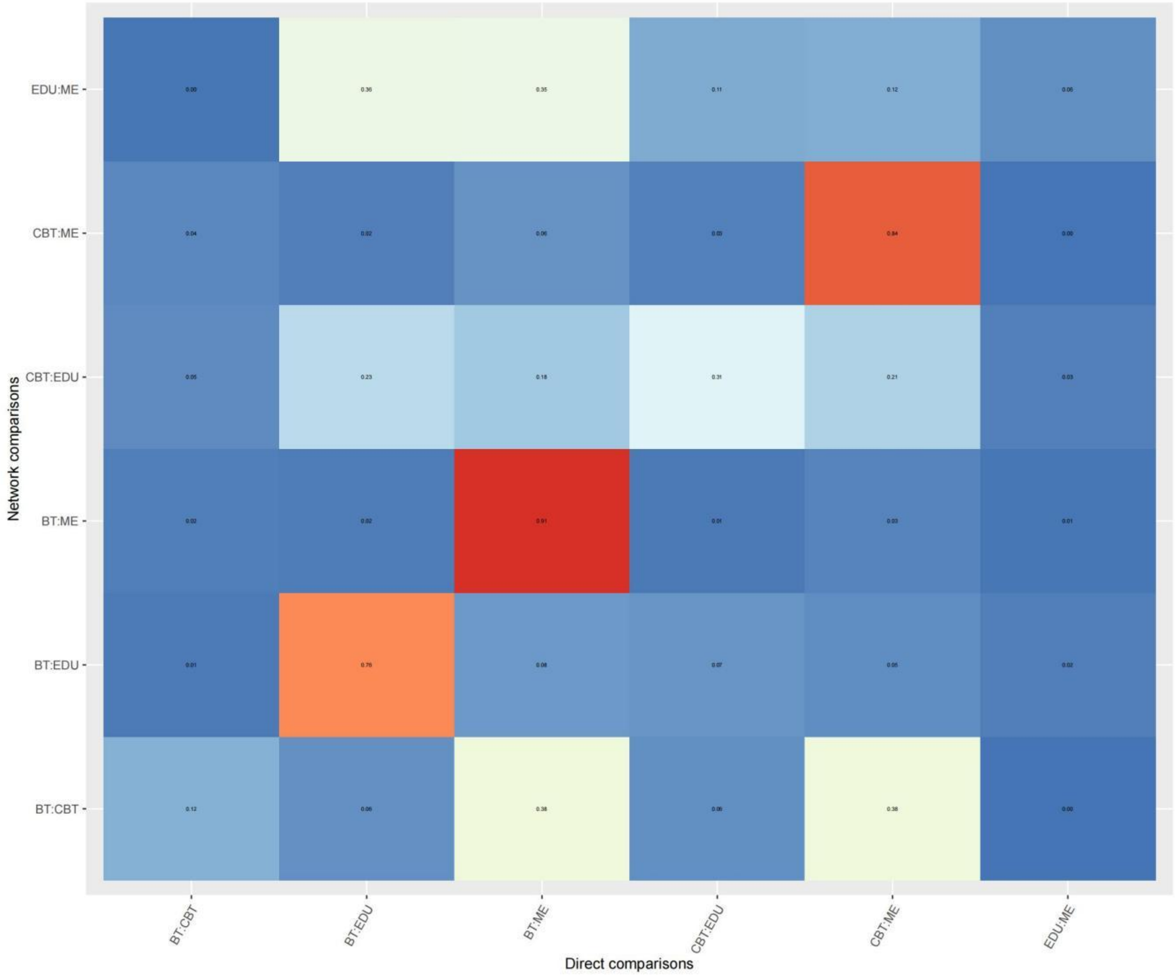

5.3.3 Contribution matrix of waist circumference

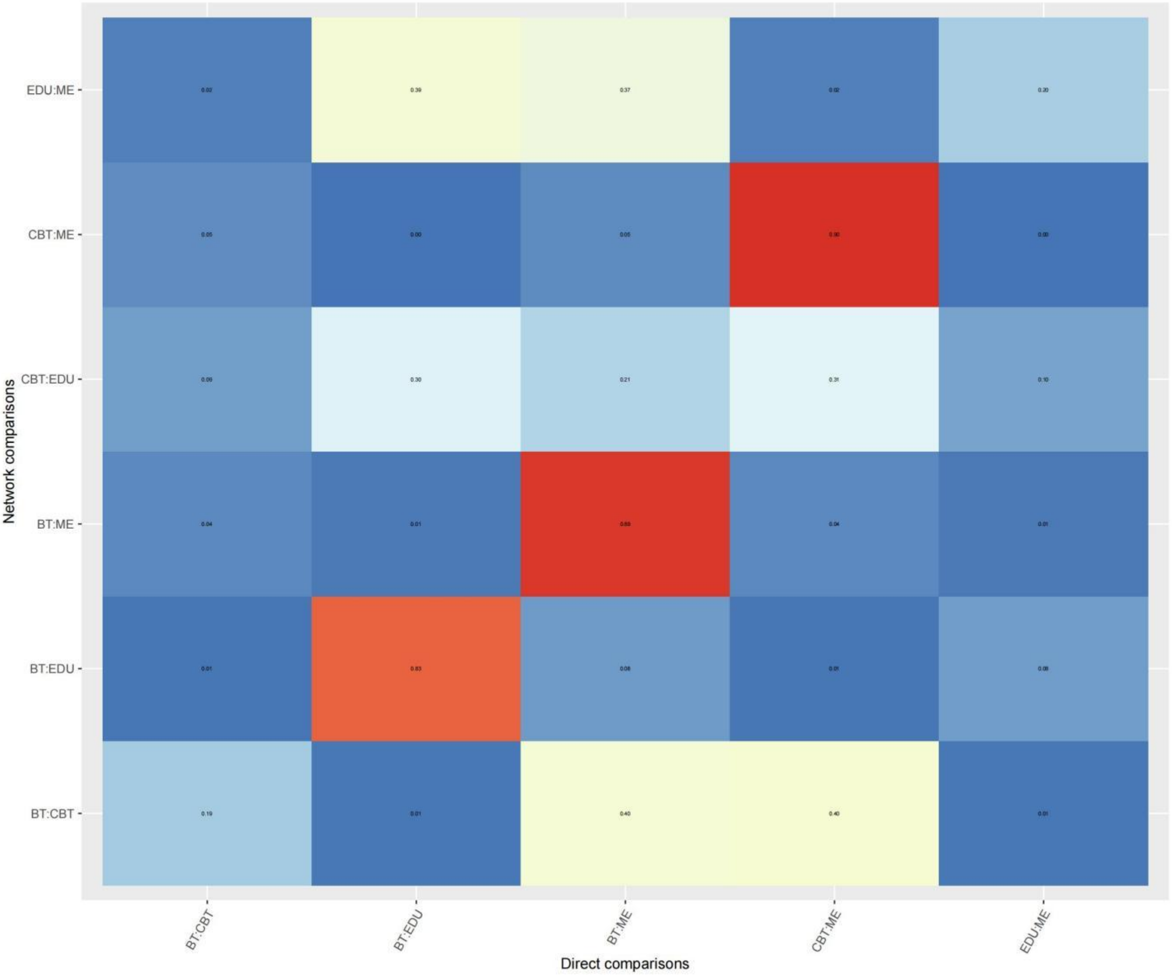

5.3.4 Contribution matrix of height

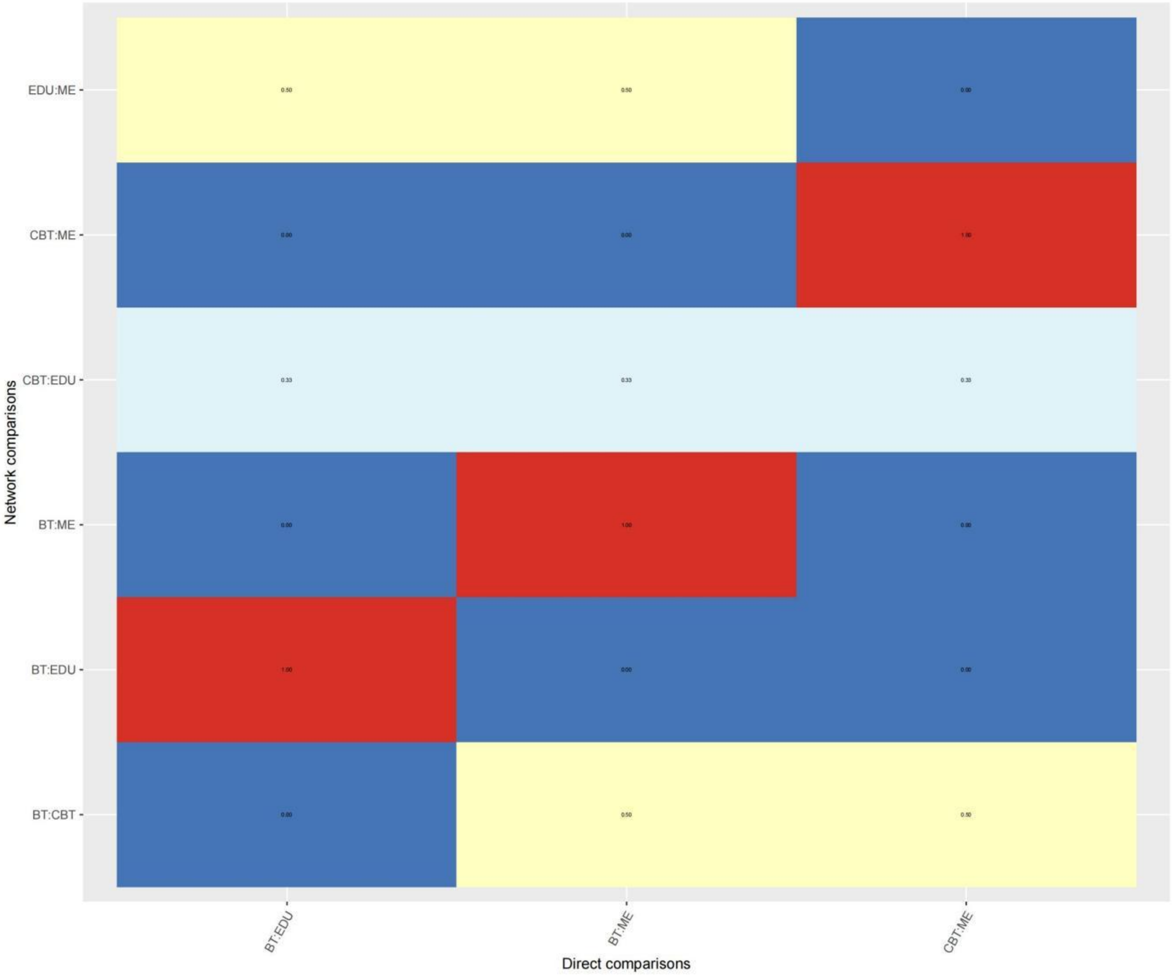

5.3.5 Contribution matrix of quality of life

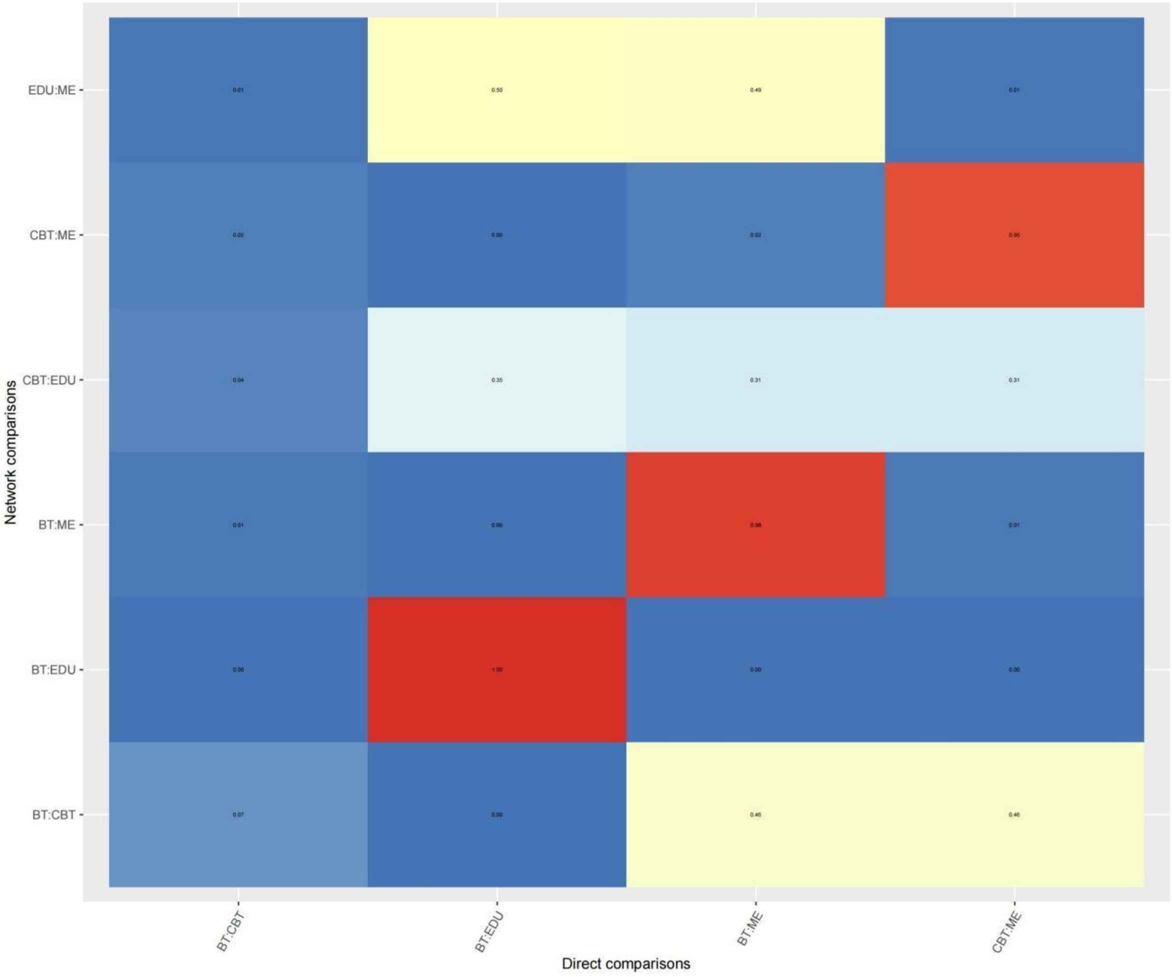

5.3.6 Contribution matrix of mental health

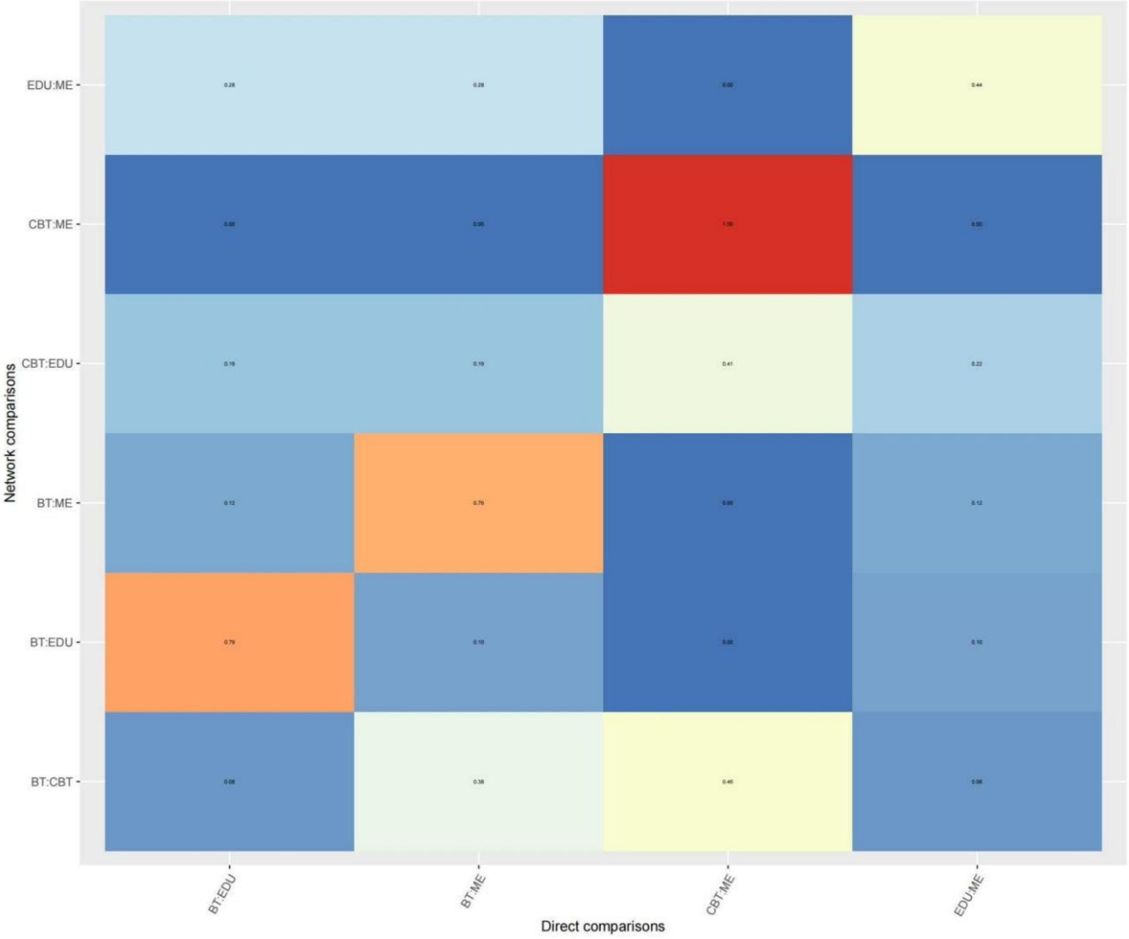

5.4 Percentage contribution matrix for technical level CNMA

Contribution matrix is used to determine the initial certainty of each stand-alone component estimate. The contribution matrix is a quantitative tool that objectively measures how much each direct comparison statistically influences the estimation of a component's effect size. Unlike traditional evidence path analysis (e.g., indirect comparisons via common comparators), this approach avoids explicit pathway enumeration and instead uses mathematical decomposition to assign proportional weights to all available evidence. (see supplement to method)

5.4.1 Contribution matrix of BMI z-score

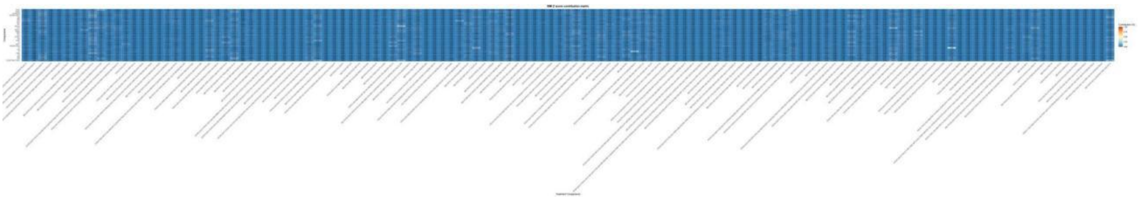

5.4.2 Contribution matrix of body fat percentage

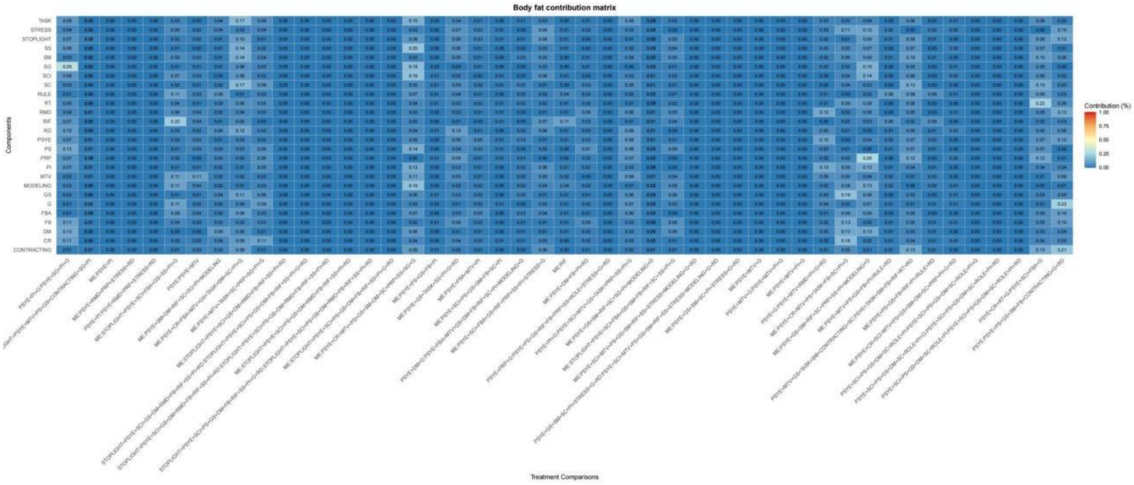

5.4.3 Contribution matrix of waist circumference

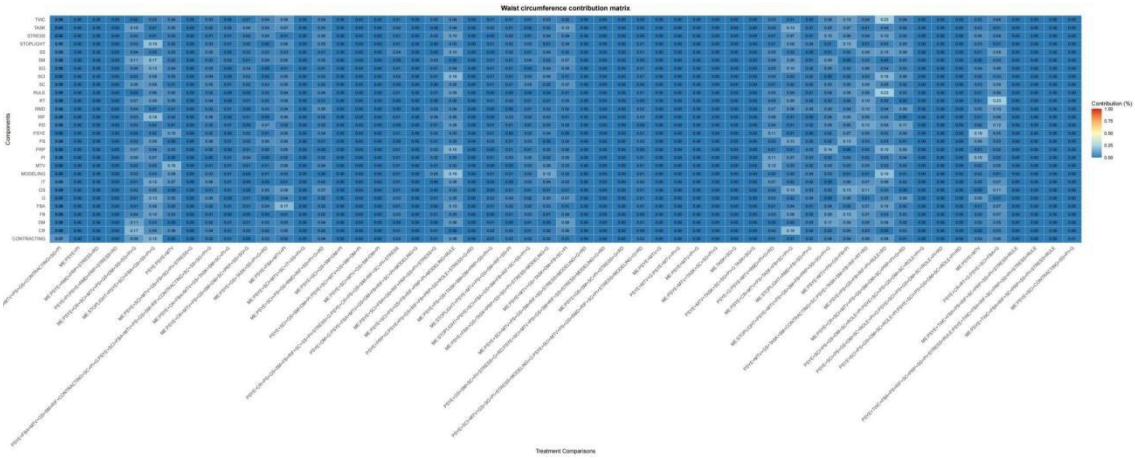

5.4.4 Contribution matrix of height

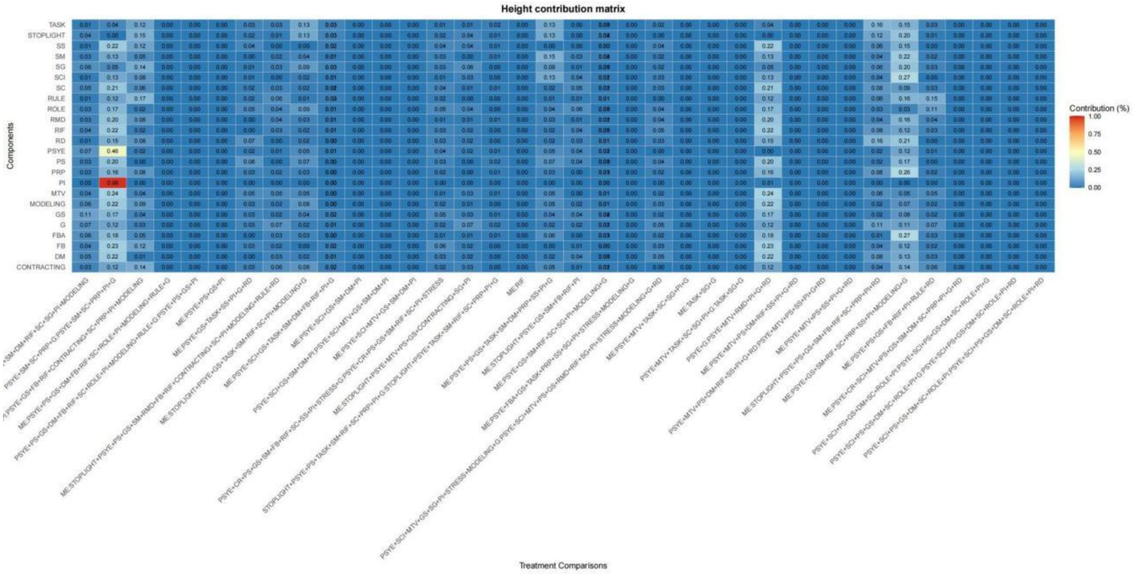

5.4.5 Contribution matrix of quality of life

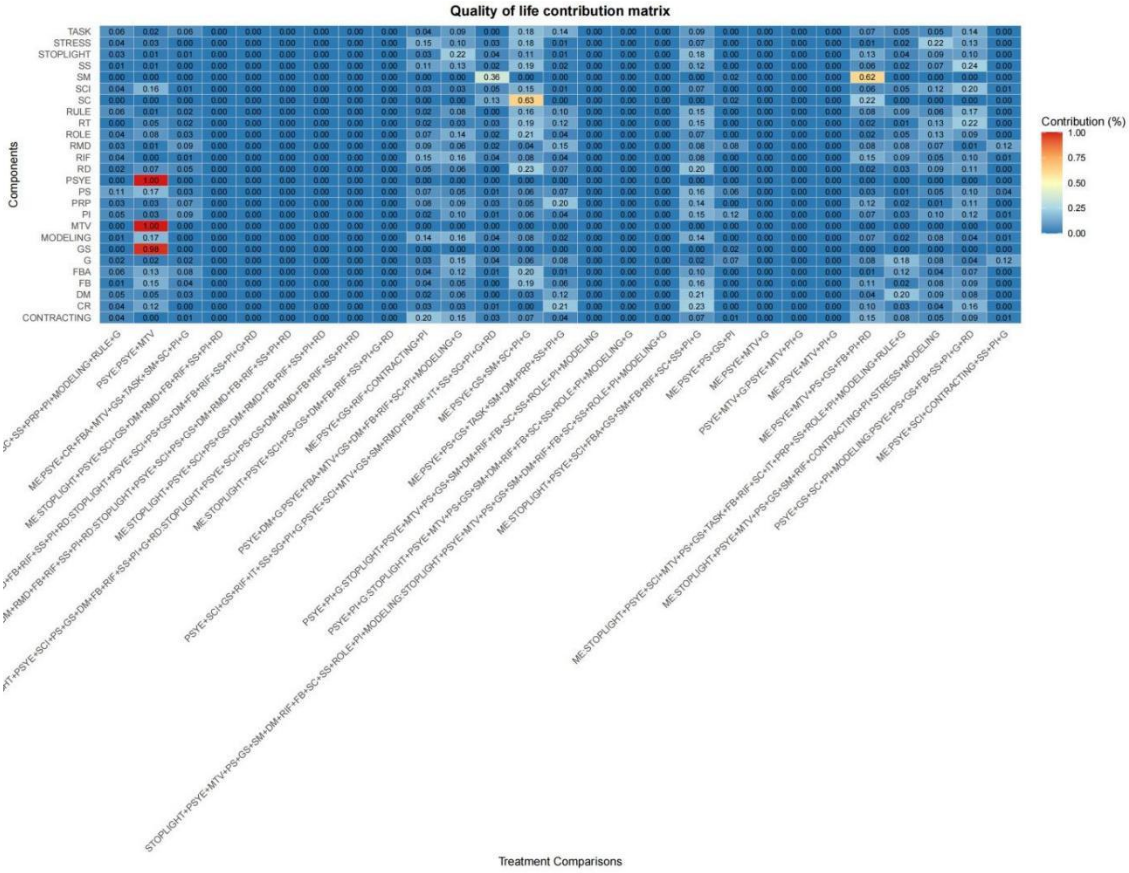

5.4.6 Contribution matrix of mental health

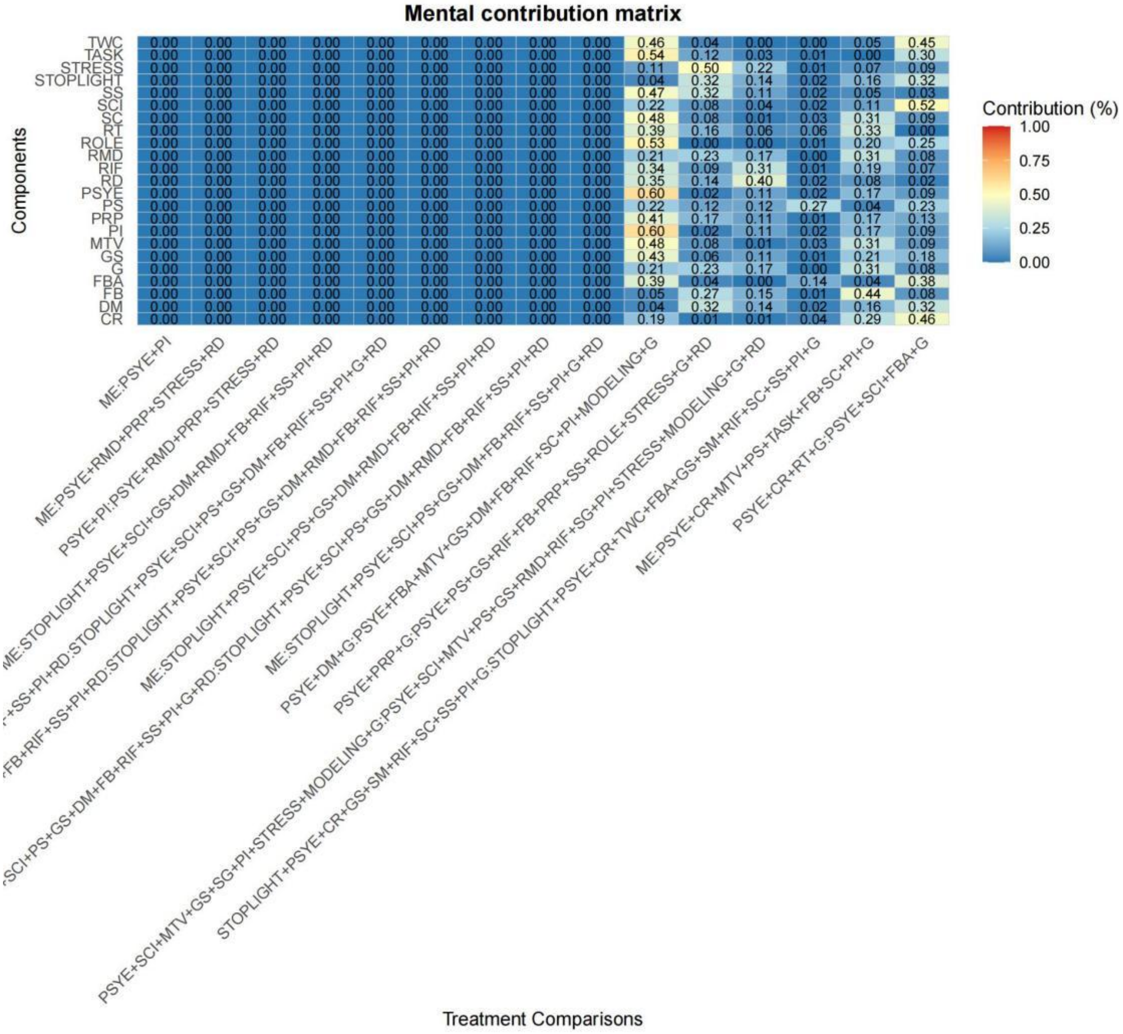

Appendix 6 Subgroup analyses

6.1 Subgroup analyses for the conceptual level NMA

6.1.1 Subgroup analysis for patients' age group at baseline

Comparisons	Adolescents	Children	Difference in difference	Interactive p value
**Absolute change of BMI z-score, mean difference (95% CI)**				
BT vs CBT	-0.00 (-0.07 to 0.07)	-0.01 (-0.06 to 0.04)	0.01 (0.06 to 0.17)	0.813
BT vs CT	-0.02 (-0.28 to 0.24)	-0.08 (-0.25 to 0.09)	0.06 (0.17 to 0.65)	0.687
BT vs EDU	-0.02 (-0.15 to 0.11)	0.01 (-0.07 to 0.10)	-0.03 (-0.16 to 0.16)	0.695
BT vs ME	-0.07 (-0.13 to -0.02)	-0.07 (-0.10 to -0.05)	-0.00 (0.01 to 0.14)	0.974
CBT vs CT	-0.02 (-0.28 to 0.25)	-0.07 (-0.24 to 0.10)	0.05 (-0.30 to 0.32)	0.734
CBT vs EDU	-0.02 (-0.15 to 0.12)	0.03 (-0.07 to 0.12)	-0.04 (0.07 to 0.25)	0.614
CBT vs ME	-0.07 (-0.13 to -0.02)	-0.06 (-0.11 to -0.02)	-0.01 (0.00 to 0.11)	0.739
CT vs EDU	-0.00 (-0.23 to 0.23)	0.10 (-0.09 to 0.28)	-0.10 (-0.44 to 0.14)	0.526
CT vs ME	-0.06 (-0.32 to 0.20)	0.01 (-0.16 to 0.18)	-0.07 (-0.35 to 0.29)	0.683
EDU vs ME	-0.06 (-0.19 to 0.07)	-0.09 (-0.17 to -0.00)	0.03 (-0.26 to 0.10)	0.711
**Absolute change of height, mean difference (95% CI)**				
BT vs ME	0.32 (-0.31 to 0.95)	0.25 (-0.14 to 0.65)	0.06 (0.55 to 1.50)	0.871
**Absolute change of body fat percentage, mean difference (95% CI)**				
BT vs CBT	-0.70 (-2.23 to 0.82)	0.13 (-0.69 to 0.94)	-0.83 (0.40 to 2.66)	0.347
BT vs EDU	-1.54 (-3.06 to -0.01)	-0.17 (-1.81 to 1.46)	-1.36 (-0.59 to 2.79)	0.232
BT vs ME	-2.26 (-3.31 to -1.21)	-0.29 (-0.75 to 0.17)	**-1.97 (-3.06 to -0.79)**	<0.001
CBT vs EDU	-0.83 (-2.95 to 1.28)	-0.30 (-1.72 to 1.12)	-0.53 (-0.66 to 5.47)	0.681
CBT vs ME	-1.56 (-2.79 to -0.33)	-0.41 (-1.08 to 0.26)	-1.15 (-2.71 to 0.11)	0.109
EDU vs ME	-0.72 (-2.52 to 1.07)	-0.11 (-1.68 to 1.46)	-0.61 (0.63 to 3.59)	0.615
**Absolute change of Waist circumference, mean difference (95% CI)**				
BT vs CBT	0.53 (-1.33 to 2.40)	-0.97 (-2.57 to 0.63)	1.50 (2.78 to 5.79)	0.232
BT vs ME	-1.83 (-3.33 to -0.34)	-1.56 (-2.57 to -0.55)	-0.27 (0.36 to 3.11)	0.768
CBT vs ME	-2.37 (-3.77 to -0.97)	-0.60 (-1.84 to 0.65)	-1.77 (-3.08 to 0.74)	0.063
**Absolute change of quality-of-life score, standardised mean difference (95% CI)**				
BT vs CBT	-0.31 (-0.74 to 0.13)	-0.27 (-0.56 to 0.02)	-0.04 (0.29 to 0.95)	0.880
BT vs EDU	0.57 (0.01 to 1.12)	0.02 (-0.25 to 0.28)	0.55 (0.73 to 1.72)	0.077
BT vs ME	0.15 (-0.19 to 0.48)	0.15 (0.04 to 0.25)	0.00 (-0.36 to 0.34)	0.994
CBT vs EDU	0.88 (0.17 to 1.58)	0.28 (-0.10 to 0.67)	0.59 (0.67 to 2.52)	0.148
CBT vs ME	0.46 (0.18 to 0.73)	0.41 (0.14 to 0.68)	0.04 (-0.45 to 0.32)	0.833
EDU vs ME	-0.42 (-1.07 to 0.23)	0.13 (-0.15 to 0.41)	-0.55 (0.06 to 0.69)	0.126
**Absolute change of mental health score, standardised mean difference (95% CI)**				
BT vs CBT	1.88 (-3.35 to 7.11)	NA	NA	NA
BT vs EDU	1.00 (-3.74 to 5.74)	NA	NA	NA
BT vs ME	1.00 (-3.98 to 5.98)	NA	NA	NA
CBT vs EDU	-0.88 (-6.95 to 5.19)	NA	NA	NA
CBT vs ME	-0.88 (-2.50 to 0.74)	NA	NA	NA

Comparisons	Adolescents	Children	Difference in difference	Interactive p value
EDU vs ME	0.00 (-5.85 to 5.85)	NA	NA	NA

Notes: BT, behavioural therapy; CBT, cognitive behavioural therapy; CT, cognitive therapy; ME, minimal education.

6.1.2 Subgroup analysis for patients' BMI at baseline

Comparisons	Obesity	Overweight	Difference in difference	Interactive p value
**Absolute change of BMI z-score, mean difference (95% CI)**				
BT vs CBT	0.00 (-0.04 to 0.05)	-0.03 (-0.16 to 0.09)	0.04 (0.01 to 0.29)	0.580
BT vs EDU	0.02 (-0.05 to 0.10)	-0.16 (-0.37 to 0.05)	0.18 (0.21 to 0.63)	0.111
BT vs ME	-0.08 (-0.10 to -0.05)	-0.06 (-0.15 to 0.03)	-0.01 (-0.04 to 0.15)	0.784
CBT vs EDU	0.02 (-0.06 to 0.09)	-0.13 (-0.37 to 0.12)	0.15 (-0.20 to 0.32)	0.265
CBT vs ME	-0.08 (-0.12 to -0.05)	-0.03 (-0.12 to 0.06)	-0.05 (-0.19 to -0.00)	0.299
EDU vs ME	-0.10 (-0.18 to -0.02)	0.10 (-0.13 to 0.33)	-0.20 (-0.27 to 0.20)	0.113
**Absolute change of height, mean difference (95% CI)**				
BT vs CBT	2.84 (1.36 to 4.32)	0.57 (-0.43 to 1.57)	**2.27 (3.39 to 5.65)**	0.013
BT vs ME	0.30 (-0.08 to 0.67)	0.12 (-0.73 to 0.97)	0.18 (0.35 to 2.01)	0.707
CBT vs ME	-2.54 (-3.97 to -1.12)	-0.45 (-0.99 to 0.08)	**-2.09 (-3.61 to -0.56)**	0.007
**Absolute change of body fat percentage, mean difference (95% CI)**				
BT vs CBT	-0.15 (-1.02 to 0.72)	0.89 (-0.84 to 2.62)	-1.04 (-0.95 to 2.45)	0.291
BT vs EDU	-0.83 (-1.96 to 0.31)	-3.49 (-4.96 to -2.02)	2.66 (3.28 to 6.21)	0.005
BT vs ME	-1.18 (-1.75 to -0.61)	-0.54 (-1.14 to 0.06)	-0.64 (-1.15 to 0.53)	0.129
CBT vs EDU	-0.68 (-1.95 to 0.60)	-4.38 (-6.65 to -2.11)	3.71 (1.38 to 6.50)	0.005
CBT vs ME	-1.03 (-1.77 to -0.29)	-1.43 (-3.05 to 0.19)	0.40 (-2.15 to 1.36)	0.658
EDU vs ME	-0.35 (-1.55 to 0.84)	2.95 (1.36 to 4.53)	-3.30 (-2.79 to 0.03)	0.001
**Absolute change of Waist circumference, mean difference (95% CI)**				
BT vs CBT	0.19 (-1.37 to 1.75)	-0.30 (-5.92 to 5.31)	0.49 (-1.08 to 10.54)	0.868
BT vs EDU	-1.54 (-4.87 to 1.78)	-4.18 (-9.30 to 0.94)	2.64 (4.40 to 14.51)	0.396
BT vs ME	-1.70 (-2.80 to -0.61)	-1.50 (-6.31 to 3.31)	-0.20 (-1.37 to 8.43)	0.936
CBT vs EDU	-1.73 (-5.33 to 1.86)	-3.88 (-11.47 to 3.72)	2.15 (-6.61 to 10.02)	0.617
CBT vs ME	-1.89 (-3.14 to -0.64)	-1.20 (-4.09 to 1.70)	-0.70 (-5.24 to 0.96)	0.665
EDU vs ME	-0.16 (-3.57 to 3.25)	2.68 (-4.34 to 9.70)	-2.84 (-3.37 to 10.90)	0.476
**Absolute change of quality-of-life score, standardised mean difference (95% CI)**				
BT vs EDU	0.02 (-0.29 to 0.32)	0.57 (0.16 to 0.98)	-0.55 (-0.42 to 0.30)	0.032
BT vs ME	0.14 (0.02 to 0.27)	0.25 (0.00 to 0.49)	-0.11 (-0.04 to 0.44)	0.447
EDU vs ME	0.13 (-0.20 to 0.45)	-0.32 (-0.80 to 0.15)	0.45 (0.16 to 1.31)	0.129
**Absolute change of mental health score, standardised mean difference (95% CI)**				
BT vs CBT	0.14 (-0.80 to 1.09)	NA	NA	NA
BT vs EDU	0.13 (-0.71 to 0.98)	NA	NA	NA
BT vs ME	0.13 (-0.71 to 0.98)	NA	NA	NA
CBT vs EDU	-0.01 (-0.95 to 0.93)	NA	NA	NA
CBT vs ME	-0.01 (-0.43 to 0.41)	NA	NA	NA
EDU vs ME	0.00 (-0.84 to 0.84)	NA	NA	NA

Notes: BT, behavioural therapy; CBT, cognitive behavioural therapy; CT, cognitive therapy; ME, minimal education.

6.1.3 Subgroup analysis for treatment settings

Comparison	School setting	Other settings	Difference in difference	Interactive p value
**Absolute change of BMI z-score, mean difference (95% CI)**				
BT vs CBT	-0.08 (-0.19 to 0.03)	0.01 (-0.03 to 0.05)	-0.09 (-0.00 to 0.16)	0.135
BT vs EDU	-0.16 (-0.38 to 0.06)	0.03 (-0.04 to 0.10)	-0.19 (-0.13 to 0.25)	0.106
BT vs ME	-0.09 (-0.16 to -0.02)	-0.07 (-0.09 to -0.05)	-0.02 (-0.10 to 0.06)	0.634
CBT vs EDU	-0.08 (-0.33 to 0.16)	0.01 (-0.05 to 0.08)	-0.10 (0.01 to 0.51)	0.453
CBT vs ME	-0.01 (-0.10 to 0.07)	-0.08 (-0.12 to -0.05)	0.07 (-0.02 to 0.16)	0.122
EDU vs ME	0.07 (-0.16 to 0.30)	-0.10 (-0.17 to -0.03)	0.17 (0.39 to 0.61)	0.166
**Absolute change of height, mean difference (95% CI)**				
BT vs CBT	0.90 (0.41 to 1.40)	2.70 (1.15 to 4.24)	**-1.79 (-2.13 to 1.27)**	0.031
BT vs ME	0.45 (0.07 to 0.83)	0.51 (-1.09 to 2.10)	0.26 (0.47 to 1.42)	0.395
CBT vs ME	-0.45 (-0.77 to -0.14)	-2.51 (-3.98 to -1.03)	**2.05 (1.71 to 4.70)**	0.008
**Absolute change of body fat percentage, mean difference (95% CI)**				
BT vs CBT	-0.49 (-2.55 to 1.57)	-0.06 (-0.89 to 0.77)	-0.43 (1.23 to 4.27)	0.706
BT vs EDU	-3.49 (-4.96 to -2.02)	-0.82 (-1.90 to 0.27)	**-2.67 (-2.02 to 0.74)**	0.004
BT vs ME	-1.92 (-3.19 to -0.65)	-1.06 (-1.59 to -0.52)	-0.86 (-2.19 to 0.56)	0.221
CBT vs EDU	-3.00 (-5.53 to -0.47)	-0.76 (-1.97 to 0.46)	-2.25 (-1.75 to 4.52)	0.117
CBT vs ME	-1.43 (-3.05 to 0.19)	-1.00 (-1.71 to -0.28)	-0.43 (-2.30 to 1.28)	0.631
EDU vs ME	1.57 (-0.37 to 3.51)	-0.24 (-1.39 to 0.90)	1.81 (3.48 to 5.81)	0.115
**Absolute change of Waist circumference, mean difference (95% CI)**				
BT vs CBT	-1.40 (-7.43 to 4.64)	0.53 (-1.04 to 2.11)	-1.93 (3.14 to 11.81)	0.544
BT vs EDU	-4.18 (-9.72 to 1.36)	-1.55 (-4.85 to 1.74)	-2.63 (-0.46 to 9.46)	0.424
BT vs ME	-1.50 (-6.87 to 3.87)	-1.74 (-2.82 to -0.66)	0.24 (-6.02 to 5.06)	0.932
CBT vs EDU	-2.78 (-10.97 to 5.41)	-2.09 (-5.66 to 1.49)	-0.69 (1.47 to 20.90)	0.879
CBT vs ME	-0.10 (-2.85 to 2.65)	-2.27 (-3.55 to -1.00)	2.17 (-1.08 to 5.07)	0.160
EDU vs ME	2.68 (-5.03 to 10.39)	-0.19 (-3.56 to 3.19)	2.87 (10.16 to 17.62)	0.504
**Absolute change of quality-of-life score, standardised mean difference (95% CI)**				
BT vs EDU	0.57 (0.16 to 0.98)	0.02 (-0.29 to 0.32)	**0.55 (0.85 to 1.49)**	0.033
BT vs ME	0.23 (-0.21 to 0.67)	0.15 (0.03 to 0.28)	0.07 (0.18 to 0.96)	0.755
EDU vs ME	-0.34 (-0.94 to 0.26)	0.14 (-0.19 to 0.47)	-0.48 (-1.08 to 0.31)	0.168
**Absolute change of mental health score, standardised mean difference (95% CI)**				
BT vs CBT	NA	0.14 (-0.80 to 1.09)	NA	NA
BT vs EDU	NA	0.13 (-0.71 to 0.98)	NA	NA
BT vs ME	NA	0.13 (-0.71 to 0.98)	NA	NA
CBT vs EDU	NA	-0.01 (-0.95 to 0.93)	NA	NA
CBT vs ME	NA	-0.01 (-0.43 to 0.41)	NA	NA
EDU vs ME	NA	0.00 (-0.84 to 0.84)	NA	NA

Notes: BT, behavioural therapy; CBT, cognitive behavioural therapy; CT, cognitive therapy; ME, minimal education.

6.2 Subgroup analyses for the technical level CNMA

6.2.1 Subgroup analysis for patients' age group at baseline

**1. Absolute BMI z-score change from baseline**								
**Components**	**Adolescents**			**Children**			**Difference in difference (95% CI)**	**Interactive p value**
	No. of studies	MD (95% CI)	p.value	No. of studies	MD (95% CI)	p.value		
contracting	3	0.11 (-0.13 to 0.36)	0.360	11	-0.08 (-0.19 to 0.03)	0.150	0.2 (-0.07 to 0.46)	0.151
cognitive restructuring	5	-0.02 (-0.32 to 0.29)	0.917	4	0.15 (-0.11 to 0.41)	0.252	-0.17 (-0.57 to 0.23)	0.411
device-monitoring	21	-0.1 (-0.26 to 0.06)	0.233	14	0.09 (-0.11 to 0.29)	0.388	-0.19 (-0.45 to 0.07)	0.156
feedback	28	0.12 (0.02 to 0.22)	0.022	36	0 (-0.08 to 0.08)	0.946	0.11 (-0.01 to 0.24)	0.082
functional behavioural analysis	10	0.06 (-0.09 to 0.2)	0.439	14	-0.03 (-0.19 to 0.14)	0.754	0.08 (-0.13 to 0.3)	0.455
group	30	-0.11 (-0.22 to 0)	0.043	43	0.01 (-0.05 to 0.06)	0.831	-0.12 (-0.24 to 0)	0.057
goal-setting	39	0.11 (-0.1 to 0.32)	0.294	64	0.04 (-0.07 to 0.15)	0.474	0.07 (-0.16 to 0.31)	0.552
inhibition training	3	0.14 (-0.1 to 0.39)	0.245	3	0.09 (-0.45 to 0.62)	0.749	0.06 (-0.53 to 0.64)	0.848
modelling	4	-0.12 (-0.4 to 0.16)	0.390	40	0.01 (-0.09 to 0.11)	0.783	-0.14 (-0.43 to 0.16)	0.367
motivation	27	-0.05 (-0.14 to 0.05)	0.338	28	-0.05 (-0.13 to 0.04)	0.275	0 (-0.13 to 0.13)	0.996
parental involvement	43	-0.15 (-0.25 to -0.04)	0.009	79	-0.07 (-0.21 to 0.07)	0.344	-0.08 (-0.26 to 0.1)	0.394
preplanning	12	0.13 (-0.07 to 0.32)	0.200	26	-0.02 (-0.13 to 0.08)	0.705	0.15 (-0.07 to 0.37)	0.191
problem solving	21	-0.02 (-0.11 to 0.07)	0.703	50	-0.03 (-0.12 to 0.06)	0.551	0.01 (-0.12 to 0.14)	0.865
psychoeducation	56	0.11 (-0.08 to 0.3)	0.259	78	0 (-0.14 to 0.15)	0.952	0.1 (-0.13 to 0.34)	0.391
remote	24	-0.07 (-0.19 to 0.06)	0.283	18	0.03 (-0.07 to 0.13)	0.568	-0.1 (-0.26 to 0.06)	0.232
reinforcement	23	0.09 (-0.04 to 0.22)	0.170	44	-0.07 (-0.17 to 0.04)	0.220	0.16 (-0.01 to 0.32)	0.066
reminders	12	-0.09 (-0.23 to 0.04)	0.169	9	-0.02 (-0.12 to 0.09)	0.735	-0.08 (-0.24 to 0.09)	0.381
role playing	1	0.55 (-0.26 to 1.35)	0.181	9	-0.09 (-0.3 to 0.12)	0.403	0.64 (-0.19 to 1.47)	0.132
relaxation training	2	0.2 (-0.19 to 0.59)	0.312	2	0.19 (-0.23 to 0.61)	0.378	0.01 (-0.56 to 0.58)	0.969

rule-setting	3	-0.53 (-1.33 to 0.26)	0.189	18	-0.01 (-0.13 to 0.12)	0.911	-0.52 (-1.33 to 0.28)	0.200
stimulus control	15	-0.16 (-0.39 to 0.07)	0.167	39	-0.04 (-0.13 to 0.06)	0.457	-0.13 (-0.38 to 0.12)	0.319
self-concept improvement	28	0.04 (-0.07 to 0.14)	0.511	22	0.07 (-0.03 to 0.17)	0.164	-0.04 (-0.18 to 0.11)	0.637
serious games	7	0.06 (-0.06 to 0.17)	0.350	12	-0.04 (-0.14 to 0.05)	0.366	0.1 (-0.05 to 0.25)	0.195
self-monitoring	30	-0.06 (-0.27 to 0.15)	0.588	39	-0.03 (-0.11 to 0.05)	0.494	-0.03 (-0.26 to 0.2)	0.795
social support	22	-0.03 (-0.15 to 0.08)	0.578	24	0.01 (-0.1 to 0.12)	0.879	-0.04 (-0.2 to 0.12)	0.612
stoplight approach	12	-0.19 (-0.4 to 0.03)	0.090	24	0.11 (-0.01 to 0.23)	0.070	**-0.3 (-0.54 to -0.05)**	0.018
stress management	11	-0.19 (-0.49 to 0.12)	0.226	8	0.09 (-0.08 to 0.27)	0.294	-0.28 (-0.63 to 0.07)	0.116
task-setting	16	-0.03 (-0.14 to 0.08)	0.632	16	-0.05 (-0.19 to 0.1)	0.523	0.02 (-0.16 to 0.2)	0.830
third-wave components	4	0.07 (-0.3 to 0.43)	0.715	5	-0.14 (-0.39 to 0.1)	0.257	0.21 (-0.23 to 0.65)	0.348
**2. Absolute height change from baseline**								
**Components**	**Adolescents**			**Children**			**Difference in difference (95% CI)**	**Interactive p value**
	No. of studies	MD (95% CI)	p.value	No. of studies	MD (95% CI)	p.value		
contracting	—	—	—	3	-0.72 (-2.12 to 0.69)	0.316	—	—
cognitive restructuring	2	-3.22 (-6.48 to 0.03)	0.052	—	—	—	—	—
device-monitoring	9	0.34 (-1.33 to 2)	0.693	6	-0.2 (-1.32 to 0.93)	0.732	0.53 (-1.48 to 2.54)	0.604
feedback	5	0.38 (-1.19 to 1.95)	0.636	5	-0.15 (-3.95 to 3.65)	0.938	0.53 (-3.58 to 4.64)	0.800
functional behavioural analysis	—	—	—	1	0.82 (-1.62 to 3.25)	0.510	—	—
group	17	-1.08 (-2.58 to 0.43)	0.160	8	2 (0.02 to 3.98)	0.048	**-3.08 (-5.56 to -0.59)**	0.015
goal-setting	14	0.54 (-0.39 to 1.47)	0.259	13	-0.01 (-2.07 to 2.06)	0.996	0.54 (-1.72 to 2.81)	0.639
modelling	4	1.13 (-0.37 to 2.63)	0.141	7	0.43 (-0.86 to 1.73)	0.511	0.69 (-1.29 to 2.67)	0.494
motivation	10	0.9 (-2.41 to 4.21)	0.594	1	-0.56 (-1.43 to 0.32)	0.211	1.46 (-1.96 to 4.88)	0.404
parental involvement	21	0 (-0.66 to 0.66)	1.000	15	0.3 (-0.76 to	0.576	-0.3 (-1.55 to 0.95)	0.636

					1.36)			
preplanning	5	0.12 (-1.1 to 1.34)	0.852	3	-0.33 (-3.49 to 2.83)	0.837	0.45 (-2.94 to 3.84)	0.795
problem solving	8	0.03 (-2.62 to 2.67)	0.984	10	-0.5 (-2.59 to 1.59)	0.640	0.53 (-2.84 to 3.9)	0.759
psychoeducation	21	-0.45 (-3.32 to 2.41)	0.757	15	0.3 (-0.76 to 1.36)	0.576	-0.76 (-3.81 to 2.3)	0.628
remote	7	0 (-2.9 to 2.91)	0.998	5	1 (-1.25 to 3.25)	0.383	-1 (-4.67 to 2.68)	0.595
reinforcement	11	-0.04 (-1.64 to 1.56)	0.965	9	-1.3 (-8.42 to 5.82)	0.721	1.26 (-6.03 to 8.56)	0.734
reminders	2	-0.4 (-2.64 to 1.85)	0.729	1	0.99 (-4.47 to 6.45)	0.722	-1.39 (-7.29 to 4.52)	0.645
role playing	—	—	—	5	-0.5 (-4.19 to 3.19)	0.789	—	—
rule-setting	—	—	—	4	-0.15 (-3.95 to 3.65)	0.938	—	—
stimulus control	9	-0.55 (-1.8 to 0.71)	0.393	10	-0.38 (-3.71 to 2.95)	0.821	-0.16 (-3.72 to 3.4)	0.928
self-concept improvement	7	-1.16 (-4.25 to 1.94)	0.464	3	—	—	—	—
serious games	6	0.2 (-1.89 to 2.29)	0.852	3	0.57 (-0.47 to 1.6)	0.281	-0.37 (-2.7 to 1.96)	0.756
self-monitoring	14	0.54 (-0.39 to 1.47)	0.259	4	1.27 (-0.55 to 3.1)	0.172	-0.73 (-2.78 to 1.31)	0.482
social support	6	0.8 (-1.15 to 2.75)	0.422	2	-2.19 (-5.33 to 0.94)	0.171	2.99 (-0.7 to 6.68)	0.112
stoplight approach	2	-0.76 (-2.67 to 1.14)	0.431	4	0.41 (-0.94 to 1.75)	0.553	-1.17 (-3.5 to 1.16)	0.324
stress management	2	—	—	1	0.82 (-1.62 to 3.25)	0.510	—	—
task-setting	6	0.48 (-0.5 to 1.45)	0.335	4	-2.22 (-5.42 to 0.98)	0.174	2.7 (-0.65 to 6.04)	0.114
**3. Absolute change of body fat percentage from baseline**								
**Components**	**Adolescents**			**Children**			**Difference in difference (95% CI)**	**Interactive p value**
	No. of studies	MD (95% CI)	p.value	No. of studies	MD (95% CI)	p.value		
contracting	2	16.62 (5.87 to 27.37)	0.002	2	3.19 (1.77 to 4.61)	<0.001	**13.43 (2.59 to 24.27)**	0.015
cognitive restructuring	4	1.21 (-1.9 to 4.32)	0.446	1	-0.51 (-1.56 to 0.53)	0.338	1.72 (-1.56 to 5)	0.304
device-monitoring	9	9.01 (1.72 to 16.29)	0.015	5	2.81 (1.22 to 4.41)	0.001	6.19 (-1.26 to 13.64)	0.103
feedback	10	-9.32 (-13.59 to	<0.001	7	-2.81 (-3.89 to	<0.001	**-6.51 (-10.92 to -2.1)**	0.004

		-5.04)			-1.72)			
functional behavioural analysis	5	5.74 (1.99 to 9.49)	0.003	2	-0.96 (-2.22 to 0.29)	0.134	**6.7 (2.75 to 10.66)**	0.001
group	22	4.51 (0.76 to 8.26)	0.018	8	3 (1.44 to 4.56)	<0.001	1.51 (-2.55 to 5.57)	0.466
goal-setting	20	-16.13 (-27.85 to -4.41)	0.007	13	0.6 (-0.11 to 1.32)	0.098	**-16.73 (-28.47 to -4.99)**	0.005
modelling	5	-3.7 (-8.29 to 0.88)	0.114	2	3.76 (1.68 to 5.84)	<0.001	**-7.47 (-12.51 to -2.43)**	0.004
motivation	13	-3.49 (-4.96 to -2.02)	<0.001	6	-0.8 (-3.22 to 1.62)	0.517	-2.69 (-5.51 to 0.14)	0.062
parental involvement	23	-0.62 (-1.42 to 0.18)	0.131	16	0.25 (-0.57 to 1.08)	0.549	-0.87 (-2.02 to 0.28)	0.138
preplanning	8	-7.85 (-12 to -3.7)	<0.001	1	2.65 (0.16 to 5.15)	0.037	**-10.5 (-15.34 to -5.66)**	<0.001
problem solving	11	0.7 (-1.39 to 2.79)	0.511	10	1.35 (0.37 to 2.32)	0.007	-0.65 (-2.95 to 1.65)	0.581
psychoeducation	32	-2.17 (-5.61 to 1.27)	0.217	16	-0.7 (-1.89 to 0.49)	0.250	-1.47 (-5.11 to 2.17)	0.429
remote	17	-0.2 (-4.51 to 4.11)	0.928	5	-0.2 (-2.01 to 1.61)	0.829	0 (-4.68 to 4.68)	1.000
reinforcement	14	6.75 (2.22 to 11.28)	0.004	5	-2.1 (-4.24 to 0.04)	0.054	**8.85 (3.84 to 13.86)**	0.001
reminders	8	3.21 (-1.11 to 7.53)	0.145	—	—	—	—	—
role playing	1	10.48 (0.74 to 20.23)	0.035	3	—	—	—	—
relaxation training	2	7.78 (-1.5 to 17.05)	0.100	—	—	—	—	—
rule-setting	—	—	—	2	3.1 (0.04 to 6.16)	0.047	—	—
stimulus control	10	7.61 (0.54 to 14.69)	0.035	8	0.85 (-0.47 to 2.17)	0.209	6.76 (-0.43 to 13.96)	0.066
self-concept improvement	12	2.86 (-1.39 to 7.1)	0.187	6	0.25 (-0.36 to 0.86)	0.421	2.61 (-1.68 to 6.9)	0.234
serious games	4	-0.43 (-3.18 to 2.32)	0.760	4	-5.41 (-6.51 to -4.31)	<0.001	**4.98 (2.02 to 7.94)**	0.001
self-monitoring	11	2.27 (-2.63 to 7.16)	0.365	3	0.41 (-0.65 to 1.47)	0.448	1.86 (-3.16 to 6.87)	0.468
social support	13	3.34 (0.17 to 6.5)	0.039	3	-2.21 (-3.17 to -1.24)	<0.001	**5.54 (2.23 to 8.85)**	0.001
stoplight approach	7	1.08 (-5.08 to 7.24)	0.731	3	1.28 (-0.34 to 2.9)	0.122	-0.2 (-6.57 to 6.17)	0.952
stress management	7	5.08 (0 to 10.16)	0.050	—	—	—	—	—
task-setting	3	-2.37 (-4.34 to -0.39)	0.019	3	-0.8 (-2.12 to 0.51)	0.231	-1.56 (-3.94 to 0.81)	0.198
**4. Absolute waist circumference change from baseline**								

Components	Adolescents			Children			Difference in difference (95% CI)	Interactive p value
	No. of studies	MD (95% CI)	p.value	No. of studies	MD (95% CI)	p.value		
contracting	3	7.49 (-1.61 to 16.59)	0.107	2	-0.04 (-1.29 to 1.21)	0.949	7.53 (-1.66 to 16.72)	0.108
cognitive restructuring	5	-4.91 (-17.97 to 8.15)	0.462	2	2.5 (0.92 to 4.08)	0.002	-7.41 (-20.56 to 5.74)	0.270
device-monitoring	7	-10.41 (-23.88 to 3.05)	0.130	5	0.97 (0.12 to 1.83)	0.026	-11.39 (-24.88 to 2.11)	0.098
feedback	5	5.73 (-0.64 to 12.1)	0.078	8	-2.09 (-3.33 to -0.84)	0.001	**7.82 (1.33 to 14.31)**	0.018
functional behavioural analysis	6	-0.28 (-5.99 to 5.43)	0.923	6	-1.78 (-3.19 to -0.37)	0.013	1.5 (-4.38 to 7.38)	0.617
group	25	-6.7 (-11.38 to -2.02)	0.005	10	2.6 (0.56 to 4.64)	0.012	**-9.3 (-14.4 to -4.2)**	<0.001
goal-setting	20	7.28 (-1.78 to 16.34)	0.115	15	-1.47 (-2.89 to -0.04)	0.043	8.75 (-0.43 to 17.92)	0.062
inhibition training	1	5.6 (-3.81 to 15)	0.243	—	—	—	—	—
modelling	3	3.84 (-9.18 to 16.86)	0.563	3	1.22 (0.01 to 2.43)	0.048	2.63 (-10.45 to 15.7)	0.694
motivation	22	-1.16 (-3.98 to 1.66)	0.420	8	2.81 (0.75 to 4.87)	0.008	**-3.97 (-7.46 to -0.48)**	0.026
parental involvement	26	-1.6 (-4.44 to 1.25)	0.271	20	-1.81 (-2.97 to -0.65)	0.002	0.22 (-2.86 to 3.29)	0.891
preplanning	7	8.09 (-3.93 to 20.12)	0.187	6	1.47 (0.24 to 2.7)	0.020	6.63 (-5.46 to 18.71)	0.283
problem solving	10	-4.46 (-9.87 to 0.96)	0.107	14	-1.65 (-4 to 0.7)	0.169	-2.81 (-8.7 to 3.09)	0.351
psychoeducation	35	2.96 (-0.43 to 6.36)	0.087	19	-0.04 (-2.25 to 2.18)	0.975	3 (-1.06 to 7.06)	0.148
remote	11	5.54 (-2.39 to 13.47)	0.171	4	1.3 (-1.05 to 3.65)	0.278	4.24 (-4.03 to 12.51)	0.315
reinforcement	9	1.85 (-4.5 to 8.2)	0.568	8	2.42 (1.13 to 3.7)	<0.001	-0.57 (-7.04 to 5.91)	0.864
reminders	5	-5.42 (-14.18 to 3.34)	0.226	1	-1.47 (-2.87 to -0.08)	0.039	-3.95 (-12.83 to 4.92)	0.383
role playing	1	-7.1 (-22.01 to 7.8)	0.350	3	—	—	—	—
relaxation training	2	1.84 (-16.12 to 19.81)	0.841	—	—	—	—	—
rule-setting	—	—	—	5	1.83 (0.21 to 3.46)	0.027	—	—
stimulus control	12	-0.45 (-5.92 to 5.01)	0.871	9	-1.1 (-2.82 to 0.63)	0.214	0.64 (-5.09 to 6.37)	0.826
self-concept improvement	15	-4.12 (-8.15 to	0.046	6	-0.27 (-1.92 to	0.751	-3.85 (-8.21 to 0.51)	0.084

		-0.08)			1.38)			
serious games	7	6.86 (1.23 to 12.48)	0.017	6	0.65 (-1.21 to 2.52)	0.493	**6.2 (0.28 to 12.13)**	0.040
self-monitoring	13	4.01 (-3.17 to 11.19)	0.274	3	1.23 (0.06 to 2.41)	0.039	2.77 (-4.5 to 10.05)	0.455
social support	12	1.46 (-4.8 to 7.73)	0.647	9	-1.25 (-3.53 to 1.02)	0.280	2.72 (-3.94 to 9.38)	0.424
stoplight approach	1	-5.82 (-15.17 to 3.54)	0.223	6	-0.28 (-1.23 to 0.67)	0.563	-5.54 (-14.94 to 3.87)	0.249
stress management	10	-10.91 (-25.44 to 3.63)	0.141	4	0.44 (-2.23 to 3.11)	0.746	-11.35 (-26.12 to 3.43)	0.132
task-setting	7	-3.77 (-8.94 to 1.39)	0.152	3	-1.53 (-3.55 to 0.48)	0.136	-2.24 (-7.79 to 3.3)	0.428
third-wave components	—	—	—	3	-0.4 (-1.39 to 0.59)	0.427	—	—
**5. Quality-of-life score change from baseline**								
**Components**	**Adolescents**			**Children**			**Difference in difference (95% CI)**	**Interactive p value**
	No. of studies	SMD (95% CI)	p.value	No. of studies	SMD (95% CI)	p.value		
contracting	1	0.21 (-0.02 to 0.43)	0.068	2	0.11 (-0.09 to 0.31)	0.261	0.09 (-0.21 to 0.39)	0.545
cognitive restructuring	1	0.16 (0.02 to 0.29)	0.025	—	—	—	—	—
device-monitoring	7	0.13 (-0.1 to 0.36)	0.277	5	-0.15 (-0.34 to 0.03)	0.100	0.28 (-0.01 to 0.58)	0.061
feedback	7	0.01 (-0.11 to 0.12)	0.876	9	0.01 (-0.09 to 0.11)	0.872	0 (-0.15 to 0.15)	0.989
functional behavioural analysis	1	0.16 (0.02 to 0.29)	0.025	3	0.11 (-0.11 to 0.33)	0.317	0.04 (-0.21 to 0.3)	0.741
group	10	-0.1 (-0.35 to 0.14)	0.421	9	-0.25 (-0.56 to 0.06)	0.108	0.15 (-0.24 to 0.54)	0.453
goal-setting	9	0.17 (0.02 to 0.32)	0.022	13	0.01 (-0.1 to 0.12)	0.888	0.16 (-0.02 to 0.35)	0.080
inhibition training	1	-0.11 (-0.21 to -0.01)	0.024	1	—	—	—	—
modelling	1	-0.11 (-0.21 to -0.01)	0.024	7	-0.08 (-0.3 to 0.15)	0.500	-0.03 (-0.28 to 0.21)	0.784
motivation	6	0.57 (0.16 to 0.98)	0.006	7	-0.03 (-0.19 to 0.14)	0.753	**0.6 (0.16 to 1.03)**	0.008
parental involvement	12	0.09 (-0.17 to 0.34)	0.504	13	0.21 (0 to 0.42)	0.055	-0.12 (-0.46 to 0.21)	0.469
preplanning	2	-0.1 (-0.3 to 0.09)	0.291	1	0.05 (-0.1 to 0.2)	0.539	-0.15 (-0.4 to 0.09)	0.226
problem solving	7	-0.15 (-0.71 to 0.4)	0.585	7	-0.15 (-0.38 to 0.09)	0.217	-0.01 (-0.61 to 0.6)	0.982
psychoeducation	14	-0.35 (-0.72 to	0.066	13	-0.06 (-0.34 to	0.669	-0.29 (-0.75 to 0.18)	0.227

		0.02)			0.22)			
remote	6	0.12 (-0.02 to 0.27)	0.104	3	0.1 (-0.11 to 0.31)	0.357	0.02 (-0.23 to 0.28)	0.864
reinforcement	7	0.01 (-0.11 to 0.12)	0.876	8	-0.02 (-0.2 to 0.16)	0.805	0.03 (-0.18 to 0.25)	0.770
reminders	5	-0.25 (-0.65 to 0.16)	0.232	1	-0.62 (-1.17 to -0.07)	0.026	0.37 (-0.31 to 1.05)	0.280
role playing	1	-0.11 (-0.21 to -0.01)	0.024	3	-0.2 (-0.39 to -0.01)	0.035	0.09 (-0.12 to 0.3)	0.400
relaxation training	—	—	—	1	-0.05 (-0.2 to 0.1)	0.539	—	—
rule-setting	1	-0.11 (-0.21 to -0.01)	0.024	1	0.05 (-0.1 to 0.2)	0.539	-0.16 (-0.34 to 0.02)	0.084
stimulus control	2	0.04 (-0.1 to 0.18)	0.537	8	0.17 (-0.05 to 0.39)	0.121	-0.13 (-0.39 to 0.13)	0.328
self-concept improvement	8	0.22 (0.04 to 0.4)	0.018	3	-0.11 (-0.45 to 0.23)	0.521	0.33 (-0.06 to 0.71)	0.095
serious games	—	—	—	1	—	—	—	—
self-monitoring	2	0.16 (0.04 to 0.28)	0.008	8	0.15 (-0.2 to 0.49)	0.406	0.02 (-0.35 to 0.38)	0.934
social support	9	0.22 (-0.02 to 0.47)	0.076	7	0.17 (-0.06 to 0.4)	0.154	0.06 (-0.28 to 0.39)	0.746
stoplight approach	7	0.01 (-0.11 to 0.12)	0.876	6	0.02 (-0.25 to 0.29)	0.874	-0.01 (-0.31 to 0.28)	0.932
stress management	—	—	—	2	0.29 (0 to 0.57)	0.048	—	—
task-setting	3	0.05 (-0.06 to 0.16)	0.368	—	—	—	—	—
**6. Mental health score change from baseline**								
**Components**	**Adolescents**			**Children**			**Difference in difference (95% CI)**	**Interactive p value**
	No. of studies	SMD (95% CI)	p.value	No. of studies	SMD (95% CI)	p.value		
cognitive restructuring	1	-0.08 (-0.27 to 0.11)	0.400	2	0.09 (0.04 to 0.15)	0.001	-0.18 (-0.37 to 0.02)	0.085
device-monitoring	6	-0.05 (-0.29 to 0.19)	0.681	1	—	—	—	—
feedback	7	-0.04 (-0.17 to 0.09)	0.540	2	-0.07 (-0.13 to -0.02)	0.006	0.04 (-0.1 to 0.17)	0.611
functional behavioural analysis	1	0.08 (-0.11 to 0.27)	0.400	2	-0.13 (-0.33 to 0.07)	0.191	0.22 (-0.06 to 0.49)	0.127
group	6	-0.1 (-0.5 to 0.3)	0.632	3	0.09 (0.04 to 0.15)	0.001	-0.19 (-0.6 to 0.22)	0.356
goal-setting	8	-0.04 (-0.17 to 0.09)	0.540	2	-0.17 (-0.26 to -0.07)	<0.001	0.13 (-0.03 to 0.29)	0.109
modelling	1	—	—	1	-0.17 (-0.26 to	<0.001	—	—

					-0.07)			
motivation	1	—	—	2	-0.07 (-0.13 to -0.02)	0.006	—	—
parental involvement	9	-0.04 (-0.42 to 0.33)	0.819	3	-0.07 (-0.13 to -0.02)	0.006	0.03 (-0.35 to 0.41)	0.876
preplanning	3	0.09 (-0.15 to 0.33)	0.475	—	—	—	—	—
problem solving	7	0.34 (-0.22 to 0.9)	0.229	1	0.09 (0.04 to 0.15)	0.001	0.25 (-0.31 to 0.81)	0.384
psychoeducation	12	0.04 (-0.31 to 0.4)	0.806	3	0.09 (0.04 to 0.15)	0.001	-0.05 (-0.41 to 0.31)	0.792
remote	10	-0.09 (-0.39 to 0.21)	0.539	—	—	—	—	—
reinforcement	8	-0.18 (-0.6 to 0.24)	0.394	2	-0.17 (-0.26 to -0.07)	<0.001	-0.01 (-0.44 to 0.41)	0.947
reminders	8	-0.01 (-0.21 to 0.2)	0.959	—	—	—	—	—
role playing	1	0.01 (-0.16 to 0.18)	0.906	—	—	—	—	—
relaxation training	1	-0.08 (-0.27 to 0.11)	0.400	—	—	—	—	—
stimulus control	—	—	—	3	-0.07 (-0.13 to -0.02)	0.006	—	—
self-concept improvement	8	0.03 (-0.19 to 0.26)	0.772	—	—	—	—	—
serious games	1	—	—	—	—	—	—	—
self-monitoring	—	—	—	1	—	—	—	—
social support	7	-0.04 (-0.17 to 0.09)	0.540	1	—	—	—	—
stoplight approach	6	-0.05 (-0.29 to 0.19)	0.681	1	—	—	—	—
stress management	4	0.1 (-0.11 to 0.31)	0.359	—	—	—	—	—
task-setting	—	—	—	1	0.09 (0.04 to 0.15)	0.001	—	—
third-wave components	—	—	—	1	0.03 (-0.2 to 0.27)	0.776	—	—

6.2.2 Subgroup analysis for patients' BMI at baseline

**1. Absolute BMI z-score change from baseline**								
**Components**	**Obesity**			**Overweight**			**Difference in difference (95% CI)**	**Interactive p value**
	No. of studies	MD (95% CI)	p.value	No. of studies	MD (95% CI)	p.value		
contracting	14	-0.05 (-0.13 to 0.03)	0.228	—	—	—	—	—
cognitive restructuring	8	-0.01 (-0.12 to 0.1)	0.887	1	0.04 (-0.04 to 0.11)	0.359	-0.04 (-0.18 to 0.09)	0.528
device-monitoring	32	-0.01 (-0.08 to 0.06)	0.797	3	0.02 (-0.06 to 0.1)	0.601	-0.03 (-0.14 to 0.08)	0.574
feedback	58	0.02 (-0.03 to 0.07)	0.439	6	0.07 (-0.01 to 0.14)	0.089	-0.05 (-0.14 to 0.04)	0.318
functional behavioural analysis	24	0.01 (-0.07 to 0.08)	0.847	—	—	—	—	—
group	65	-0.01 (-0.05 to 0.03)	0.615	8	-0.01 (-0.09 to 0.07)	0.742	0 (-0.09 to 0.09)	0.957
goal-setting	93	-0.01 (-0.08 to 0.06)	0.765	10	0.07 (-0.03 to 0.17)	0.157	-0.08 (-0.2 to 0.04)	0.182
inhibition training	5	0.09 (-0.1 to 0.27)	0.354	1	-0.05 (-0.17 to 0.06)	0.347	0.14 (-0.07 to 0.35)	0.200
modelling	41	0.01 (-0.06 to 0.09)	0.736	3	-0.03 (-0.1 to 0.04)	0.410	0.04 (-0.06 to 0.15)	0.417
motivation	49	-0.01 (-0.06 to 0.04)	0.719	6	-0.16 (-0.37 to 0.05)	0.134	0.15 (-0.06 to 0.37)	0.168
parental involvement	113	-0.07 (-0.14 to 0)	0.036	9	-0.1 (-0.19 to -0.02)	0.019	0.03 (-0.08 to 0.14)	0.580
preplanning	37	0 (-0.07 to 0.07)	0.946	1	0.04 (-0.04 to 0.11)	0.359	-0.03 (-0.14 to 0.07)	0.521
problem solving	68	0 (-0.05 to 0.05)	0.949	3	-0.01 (-0.13 to 0.11)	0.853	0.01 (-0.12 to 0.14)	0.886
psychoeducation	122	0.01 (-0.07 to 0.08)	0.879	12	0.02 (-0.1 to 0.13)	0.766	-0.01 (-0.15 to 0.13)	0.872
remote	39	-0.02 (-0.09 to 0.04)	0.507	3	0.05 (-0.06 to 0.16)	0.358	-0.07 (-0.2 to 0.05)	0.259
reinforcement	62	-0.05 (-0.13 to 0.04)	0.270	5	-0.13 (-0.28 to 0.02)	0.085	0.09 (-0.08 to 0.26)	0.322
reminders	19	0.01 (-0.06 to 0.08)	0.704	2	0.11 (-0.02 to 0.24)	0.106	-0.09 (-0.24 to 0.05)	0.213
role playing	9	-0.02 (-0.17 to 0.13)	0.774	1	0.03 (-0.08 to 0.14)	0.564	-0.05 (-0.24 to 0.13)	0.571
relaxation training	4	0.14 (-0.02 to 0.31)	0.095	—	—	—	—	—
rule-setting	20	0.01 (-0.08 to 0.1)	0.850	1	-0.07 (-0.16 to	0.149	0.08 (-0.05 to 0.2)	0.246

					0.02)			
stimulus control	50	-0.05 (-0.12 to 0.02)	0.184	4	-0.08 (-0.16 to 0)	0.063	0.03 (-0.08 to 0.14)	0.571
self-concept improvement	45	0.04 (-0.02 to 0.1)	0.165	5	0.01 (-0.08 to 0.1)	0.796	0.03 (-0.08 to 0.14)	0.600
serious games	16	-0.03 (-0.1 to 0.04)	0.415	3	0 (-0.09 to 0.09)	0.956	-0.03 (-0.14 to 0.08)	0.595
self-monitoring	63	0.01 (-0.05 to 0.07)	0.746	6	0.06 (-0.02 to 0.14)	0.158	-0.05 (-0.15 to 0.05)	0.331
social support	44	0 (-0.06 to 0.07)	0.959	2	-0.02 (-0.15 to 0.11)	0.782	0.02 (-0.13 to 0.17)	0.787
stoplight approach	34	0.05 (-0.03 to 0.12)	0.207	2	0.05 (-0.03 to 0.14)	0.220	-0.01 (-0.12 to 0.1)	0.906
stress management	18	0.04 (-0.05 to 0.14)	0.356	1	0.05 (-0.04 to 0.13)	0.256	-0.01 (-0.13 to 0.12)	0.938
task-setting	29	-0.02 (-0.08 to 0.05)	0.613	3	0.02 (-0.05 to 0.08)	0.615	-0.03 (-0.13 to 0.06)	0.475
third-wave components	9	-0.14 (-0.28 to 0)	0.058	—	—	—	—	—
**2. Absolute height change from baseline**								
**Components**	**Obesity**			**Overweight**			**Difference in difference (95% CI)**	**Interactive p value**
	No. of studies	MD (95% CI)	p.value	No. of studies	MD (95% CI)	p.value		
contracting	3	-2.28 (-5.83 to 1.28)	0.209	—	—	—	—	—
cognitive restructuring	2	-5.3 (-9.55 to -1.06)	0.014	—	—	—	—	—
device-monitoring	13	1.59 (0.15 to 3.03)	0.031	2	-0.09 (-0.27 to 0.09)	0.322	**1.68 (0.23 to 3.13)**	0.023
feedback	7	-0.55 (-2.37 to 1.27)	0.553	3	-0.17 (-0.4 to 0.06)	0.153	-0.38 (-2.21 to 1.45)	0.682
functional behavioural analysis	1	-0.6 (-2.62 to 1.42)	0.561	—	—	—	—	—
group	22	1.13 (-0.42 to 2.67)	0.154	3	0.05 (-0.15 to 0.24)	0.630	1.08 (-0.48 to 2.63)	0.175
goal-setting	23	-0.24 (-1.8 to 1.32)	0.764	4	-0.03 (-0.16 to 0.11)	0.697	-0.21 (-1.78 to 1.35)	0.791
modelling	10	0.97 (-1.43 to 3.37)	0.429	1	0.14 (-0.07 to 0.35)	0.193	0.83 (-1.58 to 3.24)	0.500
motivation	11	-0.38 (-2.2 to 1.44)	0.681	—	—	—	—	—
parental involvement	32	-0.03 (-0.55 to 0.49)	0.912	4	-0.03 (-0.16 to 0.11)	0.697	0 (-0.54 to 0.54)	0.992
preplanning	8	1.16 (-0.94 to 3.25)	0.279	—	—	—	—	—
problem solving	17	-0.38 (-1.51 to 0.76)	0.514	1	-0.08 (-0.36 to 0.21)	0.608	-0.3 (-1.47 to 0.87)	0.612
psychoeducation	32	0.74 (-1.58 to 3.06)	0.531	4	-0.03 (-0.16 to	0.697	0.77 (-1.56 to 3.09)	0.518

					0.11)			
remote	11	-0.29 (-1.46 to 0.89)	0.633	1	-0.08 (-0.36 to 0.21)	0.608	-0.21 (-1.42 to 1)	0.732
reinforcement	16	-1.28 (-2.7 to 0.14)	0.077	4	-0.03 (-0.16 to 0.11)	0.697	-1.25 (-2.68 to 0.17)	0.084
reminders	3	1.36 (-0.61 to 3.33)	0.176	—	—	—	—	—
role playing	5	-4.06 (-8.62 to 0.5)	0.081	—	—	—	—	—
rule-setting	3	2.4 (-1.6 to 6.41)	0.239	1	-0.08 (-0.36 to 0.21)	0.608	2.48 (-1.54 to 6.5)	0.226
stimulus control	18	-0.44 (-2.56 to 1.68)	0.682	1	0.14 (-0.07 to 0.35)	0.193	-0.58 (-2.71 to 1.55)	0.592
self-concept improvement	8	-0.92 (-3.99 to 2.15)	0.557	2	-0.09 (-0.27 to 0.09)	0.322	-0.83 (-3.9 to 2.25)	0.598
serious games	8	0.02 (-1.48 to 1.52)	0.977	1	0.14 (-0.07 to 0.35)	0.193	-0.12 (-1.63 to 1.39)	0.878
self-monitoring	15	-0.63 (-2.97 to 1.71)	0.597	3	0.05 (-0.15 to 0.24)	0.630	-0.68 (-3.02 to 1.67)	0.571
social support	8	-0.5 (-2.79 to 1.79)	0.667	—	—	—	—	—
stoplight approach	6	2.13 (-0.83 to 5.1)	0.158	—	—	—	—	—
stress management	3	-0.6 (-2.62 to 1.42)	0.561	—	—	—	—	—
task-setting	8	-1.55 (-3.31 to 0.22)	0.087	2	-0.09 (-0.27 to 0.09)	0.322	-1.45 (-3.23 to 0.32)	0.109
**3. Absolute change of body fat percentage from baseline**								
**Components**	**Obesity**			**Overweight**			**Difference in difference (95% CI)**	**Interactive p value**
	No. of studies	MD (95% CI)	p.value	No. of studies	MD (95% CI)	p.value		
contracting	4	3.83 (-0.83 to 8.5)	0.107	—	—	—	—	—
cognitive restructuring	4	-4.47 (-8.41 to -0.52)	0.027	1	0.46 (0.02 to 0.9)	0.040	**-4.93 (-8.9 to -0.96)**	0.015
device-monitoring	13	2.78 (-0.25 to 5.81)	0.073	1	0.46 (0.02 to 0.9)	0.040	2.32 (-0.75 to 5.38)	0.139
feedback	16	-3.07 (-5.54 to -0.61)	0.014	1	0.02 (-0.22 to 0.27)	0.861	**-3.1 (-5.57 to -0.62)**	0.014
functional behavioural analysis	7	0.52 (-1.92 to 2.96)	0.676	—	—	—	—	—
group	28	1.07 (-0.46 to 2.61)	0.171	2	-0.05 (-0.44 to 0.34)	0.789	1.12 (-0.46 to 2.71)	0.164
goal-setting	30	-0.23 (-2.34 to 1.88)	0.831	3	-0.03 (-0.21 to 0.15)	0.737	-0.2 (-2.31 to 1.92)	0.854
modelling	6	1.85 (-1.99 to 5.68)	0.346	1	-0.52 (-1.23 to 0.2)	0.156	2.36 (-1.54 to 6.27)	0.236
motivation	17	-1.45 (-3.42 to 0.51)	0.148	2	-3.49 (-4.96 to -2.02)	<0.001	2.04 (-0.41 to 4.49)	0.103
parental involvement	37	0.2 (-1.98 to 2.37)	0.860	2	-0.49 (-1 to 0.02)	0.058	0.69 (-1.54 to 2.92)	0.545

preplanning	8	-3.09 (-5.63 to -0.55)	0.017	1	0.46 (0.02 to 0.9)	0.040	**-3.55 (-6.14 to -0.97)**	0.007
problem solving	19	1.92 (-0.64 to 4.48)	0.141	2	0.48 (-0.13 to 1.1)	0.125	1.44 (-1.2 to 4.07)	0.285
psychoeducation	44	-0.8 (-3.11 to 1.51)	0.497	4	-0.03 (-0.21 to 0.15)	0.737	-0.77 (-3.08 to 1.55)	0.516
remote	21	-1.62 (-3.61 to 0.36)	0.108	1	0.02 (-0.22 to 0.27)	0.861	-1.65 (-3.64 to 0.35)	0.106
reinforcement	17	-0.34 (-2.99 to 2.31)	0.801	2	-0.49 (-1 to 0.02)	0.058	0.15 (-2.55 to 2.85)	0.912
reminders	8	1.04 (-1.56 to 3.65)	0.432	—	—	—	—	—
role playing	4	4.21 (-2.68 to 11.1)	0.231	—	—	—	—	—
relaxation training	2	9.03 (2.28 to 15.78)	0.009	—	—	—	—	—
rule-setting	1	5.86 (1.37 to 10.35)	0.010	1	0.02 (-0.22 to 0.27)	0.861	**5.84 (1.35 to 10.33)**	0.011
stimulus control	16	2.07 (-1.44 to 5.58)	0.248	2	-0.05 (-0.44 to 0.34)	0.789	2.12 (-1.41 to 5.65)	0.239
self-concept improvement	18	3.21 (-0.28 to 6.7)	0.071	—	—	—	—	—
serious games	6	-4.03 (-6.76 to -1.3)	0.004	2	-0.05 (-0.44 to 0.34)	0.789	**-3.98 (-6.74 to -1.22)**	0.005
self-monitoring	12	-2.83 (-6.13 to 0.48)	0.093	2	-0.05 (-0.44 to 0.34)	0.789	-2.77 (-6.1 to 0.55)	0.102
social support	15	-3.15 (-7.17 to 0.87)	0.124	1	0.46 (0.02 to 0.9)	0.040	-3.61 (-7.65 to 0.43)	0.080
stoplight approach	10	0 (-4.12 to 4.11)	0.999	—	—	—	—	—
stress management	7	2.99 (-0.11 to 6.09)	0.059	—	—	—	—	—
task-setting	6	3.57 (0.34 to 6.79)	0.030	—	—	—	—	—

4. Absolute waist circumference change from baseline

**Components**	**Obesity**			**Overweight**			**Difference in difference (95% CI)**	**Interactive p value**
	No. of studies	MD (95% CI)	p.value	No. of studies	MD (95% CI)	p.value		
contracting	5	0.63 (-3.46 to 4.73)	0.762	—	—	—	—	—
cognitive restructuring	6	3.48 (-0.86 to 7.82)	0.116	1	0.77 (0.44 to 1.11)	<0.001	2.7 (-1.65 to 7.06)	0.223
device-monitoring	11	2.49 (-2 to 6.97)	0.278	1	0.77 (0.44 to 1.11)	<0.001	1.71 (-2.79 to 6.21)	0.456
feedback	11	1.2 (-1.45 to 3.85)	0.374	2	-0.1 (-0.36 to 0.17)	0.482	1.3 (-1.36 to 3.96)	0.339
functional behavioural analysis	12	-0.91 (-4.49 to 2.66)	0.616	—	—	—	—	—
group	32	-1.73 (-4.15 to 0.69)	0.162	3	0.74 (0.19 to 1.29)	0.008	-2.47 (-4.96 to 0.01)	0.051
goal-setting	32	-2.13 (-5.19 to	0.174	3	0.68 (0.32 to	<0.001	-2.8 (-5.89 to 0.28)	0.075

		0.94)			1.04)			
inhibition training	—	—	—	1	-0.53 (-0.99 to -0.07)	0.023	—	—
modelling	6	-0.65 (-5.34 to 4.04)	0.786	—	—	—	—	—
motivation	26	-0.54 (-3.16 to 2.07)	0.685	4	-4.18 (-6.27 to -2.09)	<0.001	**3.64 (0.29 to 6.98)**	0.033
parental involvement	43	-0.23 (-2.87 to 2.41)	0.865	3	-0.63 (-1.05 to -0.21)	0.004	0.4 (-2.27 to 3.07)	0.771
preplanning	12	-1.39 (-5.31 to 2.53)	0.487	1	0.77 (0.44 to 1.11)	<0.001	-2.16 (-6.09 to 1.77)	0.281
problem solving	22	-1.17 (-4.05 to 1.71)	0.428	2	0.18 (-0.17 to 0.53)	0.312	-1.35 (-4.25 to 1.56)	0.363
psychoeducation	49	0.53 (-3.12 to 4.17)	0.777	5	0.15 (-0.21 to 0.51)	0.424	0.38 (-3.28 to 4.04)	0.839
remote	14	0.37 (-2.5 to 3.23)	0.802	1	-0.59 (-0.88 to -0.3)	<0.001	0.96 (-1.92 to 3.84)	0.513
reinforcement	16	1.76 (-1.97 to 5.48)	0.355	1	-0.59 (-0.88 to -0.3)	<0.001	2.35 (-1.38 to 6.09)	0.218
reminders	6	-2.18 (-5.93 to 1.56)	0.253	—	—	—	—	—
role playing	4	-6.27 (-15.31 to 2.76)	0.173	—	—	—	—	—
relaxation training	2	-5.25 (-13 to 2.5)	0.185	—	—	—	—	—
rule-setting	4	4.76 (-5.8 to 15.33)	0.377	1	-0.59 (-0.88 to -0.3)	<0.001	5.36 (-5.2 to 15.92)	0.320
stimulus control	19	-0.51 (-3.64 to 2.62)	0.750	2	0.24 (-0.22 to 0.71)	0.307	-0.75 (-3.92 to 2.41)	0.641
self-concept improvement	19	-3.07 (-6.76 to 0.61)	0.102	2	-0.03 (-0.55 to 0.49)	0.900	-3.04 (-6.76 to 0.68)	0.110
serious games	11	1.92 (-1.24 to 5.08)	0.233	2	1.27 (0.74 to 1.8)	<0.001	0.65 (-2.55 to 3.85)	0.691
self-monitoring	15	0.34 (-2.86 to 3.54)	0.835	1	0.77 (0.44 to 1.11)	<0.001	-0.43 (-3.65 to 2.78)	0.791
social support	19	2.92 (-0.22 to 6.05)	0.069	2	0.24 (-0.22 to 0.71)	0.307	2.67 (-0.5 to 5.84)	0.099
stoplight approach	7	-1.23 (-5.37 to 2.91)	0.561	—	—	—	—	—
stress management	13	2.72 (-1.99 to 7.44)	0.258	1	0.5 (0.12 to 0.88)	0.010	2.22 (-2.51 to 6.96)	0.357
task-setting	10	-2.52 (-5.85 to 0.81)	0.138	—	—	—	—	—
third-wave components	3	-10.07 (-24.88 to 4.75)	0.183	—	—	—	—	—
**5. Quality-of-life score change from baseline**								

Components	Obesity			Overweight			Difference in difference (95% CI)	Interactive p value
	No. of studies	SMD (95% CI)	p.value	No. of studies	SMD (95% CI)	p.value		
contracting	2	0.19 (-0.32 to 0.69)	0.465	1	0.05 (0 to 0.1)	0.048	0.14 (-0.37 to 0.64)	0.592
cognitive restructuring	1	0.42 (0 to 0.85)	0.053	—	—	—	—	—
device-monitoring	12	0.23 (-0.1 to 0.56)	0.178	—	—	—	—	—
feedback	16	-0.41 (-0.84 to 0.01)	0.058	—	—	—	—	—
functional behavioural analysis	4	0.11 (-0.19 to 0.41)	0.472	—	—	—	—	—
group	19	-0.25 (-0.56 to 0.06)	0.108	—	—	—	—	—
goal-setting	21	-0.13 (-0.75 to 0.49)	0.689	1	0.05 (0 to 0.1)	0.048	-0.18 (-0.8 to 0.45)	0.579
inhibition training	2	0.21 (-0.46 to 0.88)	0.540	—	—	—	—	—
modelling	8	-0.39 (-1.02 to 0.24)	0.227	—	—	—	—	—
motivation	12	0.17 (-0.03 to 0.36)	0.090	1	0.57 (0.16 to 0.98)	0.006	-0.4 (-0.85 to 0.05)	0.082
parental involvement	24	0.09 (-0.17 to 0.34)	0.504	1	0.05 (0 to 0.1)	0.048	0.04 (-0.22 to 0.3)	0.780
preplanning	3	-0.08 (-0.72 to 0.55)	0.792	—	—	—	—	—
problem solving	14	-0.15 (-0.71 to 0.4)	0.585	—	—	—	—	—
psychoeducation	25	0.2 (-0.1 to 0.51)	0.194	2	0.05 (0 to 0.1)	0.048	0.15 (-0.16 to 0.47)	0.332
remote	9	0.32 (-0.11 to 0.76)	0.144	—	—	—	—	—
reinforcement	14	0.55 (-0.49 to 1.6)	0.298	1	0.05 (0 to 0.1)	0.048	0.51 (-0.54 to 1.55)	0.344
reminders	6	-0.4 (-1.03 to 0.24)	0.221	—	—	—	—	—
role playing	4	-0.43 (-1.13 to 0.28)	0.238	—	—	—	—	—
relaxation training	1	-0.32 (-0.81 to 0.18)	0.209	—	—	—	—	—
rule-setting	2	0.53 (-0.34 to 1.39)	0.232	—	—	—	—	—
stimulus control	10	0.39 (-0.89 to 1.67)	0.550	—	—	—	—	—
self-concept improvement	11	-0.22 (-1 to 0.56)	0.584	—	—	—	—	—
serious games	1	—	—	—	—	—	—	—
self-monitoring	10	-0.08 (-0.87 to 0.72)	0.850	—	—	—	—	—
social support	16	0.28 (-0.19 to 0.75)	0.248	—	—	—	—	—
stoplight approach	13	-0.14 (-1.21 to 0.94)	0.803	—	—	—	—	—
stress management	2	0.13 (-0.57 to 0.84)	0.710	—	—	—	—	—
task-setting	3	0.02 (-0.39 to 0.43)	0.926	—	—	—	—	—

6.2.3 Subgroup analysis for treatment settings

1. Absolute BMI z-score change from baseline								
**Components**	**School setting**			**Other settings**			**Difference in difference (95% CI)**	**Interactive p value**
	No. of studies	MD (95% CI)	p.value	No. of studies	MD (95% CI)	p.value		
contracting	—	—	—	14	-0.06 (-0.14 to 0.02)	0.155	—	—
cognitive restructuring	1	0.12 (-0.01 to 0.25)	0.065	8	-0.03 (-0.15 to 0.08)	0.602	0.15 (-0.02 to 0.32)	0.086
device-monitoring	3	0.05 (-0.01 to 0.1)	0.117	32	-0.01 (-0.09 to 0.06)	0.757	0.06 (-0.04 to 0.15)	0.230
feedback	8	0.13 (-0.03 to 0.29)	0.113	56	0.02 (-0.03 to 0.07)	0.476	0.11 (-0.06 to 0.28)	0.192
functional behavioural analysis	1	-0.05 (-0.13 to 0.03)	0.226	23	0.02 (-0.06 to 0.09)	0.646	-0.07 (-0.18 to 0.04)	0.229
group	11	0.02 (-0.07 to 0.12)	0.642	62	-0.01 (-0.05 to 0.04)	0.758	0.03 (-0.08 to 0.14)	0.582
goal-setting	15	0.05 (-0.02 to 0.11)	0.151	88	0 (-0.07 to 0.07)	0.978	0.05 (-0.05 to 0.14)	0.322
inhibition training	—	—	—	6	0.03 (-0.15 to 0.2)	0.768	—	—
modelling	6	0.06 (-0.1 to 0.22)	0.463	38	0.02 (-0.06 to 0.09)	0.690	0.05 (-0.14 to 0.23)	0.621
motivation	7	-0.16 (-0.35 to 0.03)	0.103	48	-0.02 (-0.07 to 0.04)	0.549	-0.14 (-0.34 to 0.05)	0.154
parental involvement	11	-0.21 (-0.34 to -0.07)	0.002	111	-0.07 (-0.16 to 0.01)	0.069	-0.13 (-0.29 to 0.02)	0.097
preplanning	3	0.02 (-0.08 to 0.13)	0.649	35	0 (-0.07 to 0.07)	0.991	0.03 (-0.1 to 0.15)	0.700
problem solving	3	-0.04 (-0.17 to 0.1)	0.599	68	0 (-0.05 to 0.06)	0.888	-0.04 (-0.18 to 0.1)	0.590
psychoeducation	16	0.05 (-0.02 to 0.11)	0.151	118	0.01 (-0.08 to 0.1)	0.869	0.04 (-0.07 to 0.15)	0.477
remote	5	0.1 (0 to 0.2)	0.051	37	-0.03 (-0.1 to 0.03)	0.332	**0.13 (0.01 to 0.26)**	0.031
reinforcement	7	0.04 (-0.1 to 0.17)	0.575	60	-0.05 (-0.12 to 0.02)	0.180	0.09 (-0.07 to 0.24)	0.257
reminders	5	-0.02 (-0.1 to 0.06)	0.643	16	0.03 (-0.04 to 0.1)	0.404	-0.05 (-0.16 to 0.06)	0.370
role playing	1	0.01 (-0.18 to 0.2)	0.922	9	-0.02 (-0.17 to 0.14)	0.825	0.03 (-0.22 to 0.27)	0.830
relaxation training	—	—	—	4	0.17 (0 to 0.34)	0.052	—	—
rule-setting	—	—	—	21	0.01 (-0.08 to 0.1)	0.851	—	—
stimulus control	5	0 (-0.09 to 0.09)	0.967	49	-0.05 (-0.12 to 0.03)	0.212	0.05 (-0.07 to 0.16)	0.407

self-concept improvement	5	0.07 (-0.01 to 0.16)	0.101	45	0.03 (-0.03 to 0.09)	0.337	0.04 (-0.06 to 0.15)	0.445
serious games	5	-0.14 (-0.31 to 0.03)	0.102	14	-0.01 (-0.09 to 0.06)	0.701	-0.13 (-0.31 to 0.06)	0.178
self-monitoring	13	-0.08 (-0.17 to 0.01)	0.077	56	0.02 (-0.04 to 0.07)	0.527	-0.1 (-0.21 to 0.01)	0.066
social support	2	0.07 (-0.03 to 0.17)	0.171	44	0 (-0.08 to 0.07)	0.898	0.07 (-0.05 to 0.19)	0.238
stoplight approach	3	0.02 (-0.15 to 0.19)	0.800	33	0.04 (-0.03 to 0.12)	0.258	-0.02 (-0.21 to 0.16)	0.820
stress management	2	0.13 (0 to 0.26)	0.058	17	0.03 (-0.06 to 0.13)	0.494	0.1 (-0.07 to 0.26)	0.253
task-setting	6	-0.1 (-0.28 to 0.07)	0.249	26	-0.01 (-0.09 to 0.06)	0.720	-0.09 (-0.28 to 0.1)	0.365
third-wave components	—	—	—	9	-0.13 (-0.27 to 0.02)	0.086	—	—
**2. Absolute height change from baseline**								
**Components**	**School setting**			**Other settings**			**Difference in difference (95% CI)**	**Interactive p value**
	No. of studies	MD (95% CI)	p.value	No. of studies	MD (95% CI)	p.value		
contracting	—	—	—	3	2.3 (-6.05 to 10.66)	0.589	—	—
cognitive restructuring	—	—	—	2	-6.2 (-14.43 to 2.02)	0.139	—	—
device-monitoring	2	-0.21 (-0.52 to 0.09)	0.170	13	0.76 (-2.71 to 4.23)	0.666	-0.98 (-4.46 to 2.51)	0.582
feedback	3	-0.17 (-0.69 to 0.35)	0.527	7	-0.83 (-3.87 to 2.22)	0.595	0.66 (-2.43 to 3.75)	0.676
functional behavioural analysis	1	0.05 (-0.12 to 0.22)	0.556	—	—	—	—	—
group	6	-0.02 (-0.54 to 0.51)	0.948	19	2.08 (0.11 to 4.05)	0.038	**-2.1 (-4.13 to -0.06)**	0.044
goal-setting	7	0.03 (-0.23 to 0.28)	0.827	20	2.12 (-1.88 to 6.13)	0.299	-2.09 (-6.11 to 1.92)	0.307
modelling	4	0.2 (-0.09 to 0.49)	0.182	7	-2.82 (-9.89 to 4.25)	0.434	3.02 (-4.06 to 10.09)	0.403
motivation	—	—	—	11	0.57 (-2.44 to 3.58)	0.711	—	—
parental involvement	7	0.03 (-0.23 to 0.28)	0.827	29	-0.02 (-0.54 to 0.51)	0.951	0.04 (-0.54 to 0.63)	0.880
preplanning	1	0.05 (-0.12 to 0.22)	0.556	7	-2.53 (-8.25 to 3.18)	0.385	2.58 (-3.13 to 8.3)	0.376
problem solving	—	—	—	18	0.01 (-1.67 to 1.69)	0.991	—	—

psychoeducation	7	0.03 (-0.23 to 0.28)	0.827	29	-2.03 (-7.16 to 3.11)	0.439	2.06 (-3.09 to 7.2)	0.433
remote	—	—	—	12	0.71 (-1.38 to 2.81)	0.504	—	—
reinforcement	6	-0.02 (-0.2 to 0.16)	0.811	14	-0.48 (-3.89 to 2.94)	0.784	0.46 (-2.96 to 3.87)	0.794
reminders	—	—	—	3	-0.67 (-4.42 to 3.07)	0.724	—	—
role playing	—	—	—	5	-0.43 (-9.64 to 8.79)	0.928	—	—
rule-setting	—	—	—	4	0.08 (-5.7 to 5.87)	0.978	—	—
stimulus control	3	0.15 (-0.26 to 0.55)	0.483	16	1.38 (-3.81 to 6.58)	0.603	-1.23 (-6.45 to 3.98)	0.642
self-concept improvement	2	-0.21 (-0.52 to 0.09)	0.170	8	-5.27 (-13.13 to 2.58)	0.188	5.06 (-2.81 to 12.92)	0.208
serious games	3	-0.08 (-0.48 to 0.31)	0.677	6	-0.15 (-3.4 to 3.1)	0.929	0.06 (-3.21 to 3.34)	0.969
self-monitoring	6	-0.02 (-0.2 to 0.16)	0.811	12	4.42 (-4.48 to 13.31)	0.331	-4.44 (-13.33 to 4.46)	0.328
social support	1	0.05 (-0.12 to 0.22)	0.556	7	-1.16 (-4.54 to 2.22)	0.500	1.21 (-2.17 to 4.6)	0.482
stoplight approach	2	0.33 (-0.3 to 0.95)	0.306	4	-2.87 (-11.65 to 5.91)	0.522	3.19 (-5.61 to 11.99)	0.477
stress management	1	0.05 (-0.12 to 0.22)	0.556	2	—	—	—	—
task-setting	4	0.12 (-0.51 to 0.74)	0.714	6	-2.35 (-6.17 to 1.47)	0.228	2.47 (-1.41 to 6.34)	0.2117
**3. Absolute change of body fat percentage from baseline**								
**Components**	**School setting**			**Other settings**			**Difference in difference (95% CI)**	**Interactive p value**
	No. of studies	MD (95% CI)	p.value	No. of studies	MD (95% CI)	p.value		
contracting	—	—	—	4	1.05 (-3.37 to 5.47)	0.641	—	—
cognitive restructuring	1	0.82 (0.45 to 1.19)	<0.001	4	-2.01 (-6.54 to 2.52)	0.385	2.83 (-1.71 to 7.37)	0.222
device-monitoring	1	0.82 (0.45 to 1.19)	<0.001	13	2.49 (-0.41 to 5.4)	0.092	-1.67 (-4.6 to 1.26)	0.263
feedback	1	-0.22 (-0.5 to 0.07)	0.142	16	-1.18 (-3.64 to 1.27)	0.343	0.97 (-1.5 to 3.44)	0.441
functional behavioural analysis	—	—	—	7	1.12 (-0.92 to 3.16)	0.282	—	—
group	5	0.8 (0.52 to 1.08)	<0.001	25	0.85 (-0.48 to 2.18)	0.210	-0.05 (-1.41 to 1.31)	0.943

goal-setting	4	0.58 (0.33 to 0.83)	<0.001	29	0.1 (-1.93 to 2.14)	0.922	0.48 (-1.57 to 2.53)	0.645
modelling	2	-0.02 (-0.34 to 0.3)	0.881	5	-1.35 (-5.38 to 2.68)	0.512	1.32 (-2.72 to 5.37)	0.521
motivation	2	-3.49 (-4.96 to -2.02)	<0.001	17	-0.37 (-1.98 to 1.24)	0.652	**-3.12 (-5.3 to -0.94)**	0.005
parental involvement	3	-0.02 (-0.34 to 0.3)	0.881	36	-0.21 (-1.98 to 1.56)	0.814	0.19 (-1.61 to 1.99)	0.838
preplanning	2	0.82 (0.45 to 1.19)	<0.001	7	-3.8 (-6.01 to -1.59)	0.001	**4.62 (2.38 to 6.87)**	<0.001
problem solving	2	0.61 (0.26 to 0.96)	0.001	19	1.93 (-0.42 to 4.27)	0.107	-1.32 (-3.69 to 1.05)	0.276
psychoeducation	6	0.8 (0.52 to 1.08)	<0.001	42	-1.83 (-3.74 to 0.09)	0.062	**2.63 (0.69 to 4.56)**	0.008
remote	1	-0.22 (-0.5 to 0.07)	0.142	21	-1.46 (-3.18 to 0.25)	0.095	1.25 (-0.49 to 2.99)	0.160
reinforcement	3	-0.24 (-0.67 to 0.19)	0.272	16	-1.45 (-3.4 to 0.5)	0.145	1.21 (-0.78 to 3.21)	0.234
reminders	—	—	—	8	0.81 (-1.36 to 2.98)	0.464	—	—
role playing	1	-0.22 (-0.5 to 0.07)	0.142	3	—	—	—	—
relaxation training	—	—	—	2	5.92 (-0.74 to 12.58)	0.081	—	—
rule-setting	—	—	—	2	3.71 (0.3 to 7.12)	0.033	—	—
stimulus control	3	0.8 (0.52 to 1.08)	<0.001	15	2.2 (-0.74 to 5.14)	0.142	-1.4 (-4.36 to 1.55)	0.352
self-concept improvement	—	—	—	18	1.38 (-1.6 to 4.35)	0.365	—	—
serious games	4	-5.41 (-6.51 to -4.31)	<0.001	4	1.61 (-4.25 to 7.47)	0.591	**-7.02 (-12.98 to -1.06)**	0.021
self-monitoring	3	0.8 (0.52 to 1.08)	<0.001	11	-2.43 (-5.09 to 0.22)	0.073	**3.23 (0.56 to 5.9)**	0.018
social support	2	0.61 (0.26 to 0.96)	0.001	14	1.81 (-2.75 to 6.36)	0.437	-1.2 (-5.77 to 3.37)	0.607
stoplight approach	—	—	—	10	-2.8 (-7.11 to 1.5)	0.202	—	—
stress management	1	-0.22 (-0.5 to 0.07)	0.142	6	3.3 (0.68 to 5.92)	0.013	**-3.52 (-6.15 to -0.88)**	0.009
task-setting	—	—	—	6	-0.19 (-4.38 to 4.01)	0.931	—	—
**4.Absolute waist circumference change from baseline**								
**Components**	**School setting**			**Other settings**			**Difference in difference (95% CI)**	**Interactive p value**
	No. of studies	MD (95% CI)	p.value	No. of studies	MD (95% CI)	p.value		

contracting	—	—	—	5	1.43 (-2.92 to 5.77)	0.520	—	—
cognitive restructuring	2	2.22 (1.44 to 2.99)	<0.001	5	1.18 (-4.86 to 7.22)	0.701	1.04 (-5.05 to 7.12)	0.738
device-monitoring	2	2.22 (1.44 to 2.99)	<0.001	10	0.29 (-5.24 to 5.81)	0.918	1.93 (-3.65 to 7.51)	0.498
feedback	2	0.7 (-0.08 to 1.48)	0.081	11	1.35 (-1.27 to 3.96)	0.312	-0.65 (-3.38 to 2.08)	0.642
functional behavioural analysis	1	-0.76 (-1.25 to -0.27)	0.002	11	-1.2 (-4.61 to 2.21)	0.491	0.44 (-3.01 to 3.88)	0.804
group	6	-0.15 (-0.72 to 0.43)	0.619	29	-2.09 (-4.49 to 0.32)	0.089	1.94 (-0.53 to 4.42)	0.124
goal-setting	6	0.3 (-0.04 to 0.64)	0.087	29	-2.81 (-6.25 to 0.63)	0.109	3.11 (-0.35 to 6.57)	0.078
inhibition training	—	—	—	1	-1.75 (-8.8 to 5.3)	0.626	—	—
modelling	1	-0.76 (-1.25 to -0.27)	0.002	5	0.79 (-4.22 to 5.79)	0.758	-1.55 (-6.58 to 3.48)	0.546
motivation	4	-4.18 (-6.27 to -2.09)	<0.001	26	-0.13 (-3.15 to 2.89)	0.934	**-4.05 (-7.72 to -0.38)**	0.030
parental involvement	4	-0.27 (-0.9 to 0.36)	0.407	42	0.12 (-2.44 to 2.68)	0.928	-0.38 (-3.02 to 2.25)	0.775
preplanning	3	-0.64 (-1.42 to 0.13)	0.104	10	0.38 (-3.14 to 3.9)	0.832	-1.02 (-4.63 to 2.58)	0.578
problem solving	4	0.81 (0.26 to 1.35)	0.004	20	-0.91 (-3.79 to 1.96)	0.533	1.72 (-1.2 to 4.64)	0.249
psychoeducation	7	-0.15 (-0.72 to 0.43)	0.619	47	-0.23 (-4.24 to 3.79)	0.913	0.08 (-3.98 to 4.13)	0.969
remote	2	-1.41 (-1.95 to -0.88)	<0.001	13	-0.12 (-2.84 to 2.61)	0.934	-1.3 (-4.07 to 1.48)	0.359
reinforcement	2	-1.41 (-1.95 to -0.88)	<0.001	15	0.43 (-3.36 to 4.22)	0.824	-1.84 (-5.67 to 1.99)	0.346
reminders	1	-1.86 (-2.64 to -1.07)	<0.001	5	-2.36 (-6.45 to 1.73)	0.258	0.5 (-3.66 to 4.67)	0.813
role playing	1	0.44 (-0.2 to 1.09)	0.180	3	—	—	—	—
relaxation training	—	—	—	2	-2.19 (-11.23 to 6.84)	0.634	—	—
rule-setting	—	—	—	5	1.43 (-5.91 to 8.76)	0.703	—	—
stimulus control	1	0.12 (-0.83 to 1.07)	0.804	20	-0.13 (-3.22 to 2.97)	0.935	0.25 (-2.99 to 3.49)	0.881
self-concept improvement	3	0.5 (-0.13 to 1.12)	0.119	18	-1.66 (-5.41 to 2.1)	0.388	2.15 (-1.66 to 5.96)	0.268

serious games	4	1.71 (1.08 to 2.34)	<0.001	9	0.18 (-3.87 to 4.22)	0.932	1.54 (-2.55 to 5.63)	0.462
self-monitoring	1	0.12 (-0.83 to 1.07)	0.804	15	1.37 (-2.11 to 4.86)	0.440	-1.25 (-4.87 to 2.36)	0.497
social support	5	0.04 (-0.65 to 0.74)	0.904	16	1.86 (-1.35 to 5.07)	0.256	-1.82 (-5.1 to 1.47)	0.278
stoplight approach	—	—	—	7	0.14 (-4.18 to 4.47)	0.948	—	—
stress management	3	-0.06 (-0.66 to 0.53)	0.833	11	3 (-1.98 to 7.97)	0.238	-3.06 (-8.07 to 1.95)	0.231
task-setting	1	-0.76 (-1.25 to -0.27)	0.002	9	-0.38 (-4.58 to 3.81)	0.858	-0.38 (-4.6 to 3.85)	0.861
third-wave components	—	—	—	3	-6.18 (-18.2 to 5.84)	0.314	—	—
**5. Quality-of-life score change from baseline**								
**Components**	**School setting**			**Other settings**			**Difference in difference (95% CI)**	**Interactive p value**
	No. of studies	SMD (95% CI)	p.value	No. of studies	SMD (95% CI)	p.value		
contracting	—	—	—	3	-0.07 (-0.95 to 0.82)	0.879	—	—
cognitive restructuring	—	—	—	1	0.44 (-0.02 to 0.9)	0.059	—	—
device-monitoring	—	—	—	12	0.16 (-0.14 to 0.47)	0.284	—	—
feedback	—	—	—	16	-0.21 (-0.47 to 0.06)	0.123	—	—
functional behavioural analysis	—	—	—	4	0.37 (0.02 to 0.71)	0.037	—	—
group	1	0.04 (-0.04 to 0.11)	0.311	18	-0.25 (-0.56 to 0.06)	0.108	0.29 (-0.03 to 0.6)	0.072
goal-setting	1	0.04 (-0.04 to 0.11)	0.311	21	-0.07 (-0.69 to 0.56)	0.840	0.1 (-0.53 to 0.74)	0.750
inhibition training	—	—	—	2	0.04 (-0.66 to 0.74)	0.907	—	—
modelling	—	—	—	8	0.01 (-0.78 to 0.8)	0.973	—	—
motivation	1	0.57 (0.16 to 0.98)	0.006	12	0.23 (0.01 to 0.45)	0.041	0.34 (-0.12 to 0.8)	0.152
parental involvement	1	0.04 (-0.04 to 0.11)	0.311	24	0.09 (-0.17 to 0.34)	0.504	-0.05 (-0.31 to 0.22)	0.718
preplanning	—	—	—	3	-0.15 (-0.97 to 0.67)	0.725	—	—
problem solving	—	—	—	14	-0.15 (-0.71 to	0.585	—	—

					0.4)			
psychoeducation	2	0.04 (-0.04 to 0.11)	0.311	25	0.14 (-0.12 to 0.41)	0.296	-0.1 (-0.38 to 0.17)	0.460
remote	—	—	—	9	0.06 (-0.21 to 0.33)	0.686	—	—
reinforcement	—	—	—	15	0.15 (-0.49 to 0.79)	0.638	—	—
reminders	—	—	—	6	-0.4 (-1.03 to 0.24)	0.221	—	—
role playing	—	—	—	4	-0.19 (-0.56 to 0.18)	0.313	—	—
relaxation training	—	—	—	1	-0.03 (-0.36 to 0.3)	0.875	—	—
rule-setting	—	—	—	2	0.07 (-0.48 to 0.62)	0.807	—	—
stimulus control	1	0.04 (-0.04 to 0.11)	0.3107	9	-0.2 (-1.37 to 0.98)	0.745	0.23 (-0.95 to 1.41)	0.698
self-concept improvement	—	—	—	11	0.22 (-0.56 to 1.01)	0.573	—	—
serious games	—	—	—	1	—	—	—	—
self-monitoring	1	0.04 (-0.04 to 0.11)	0.3107	9	-0.08 (-0.87 to 0.72)	0.850	0.11 (-0.68 to 0.91)	0.778
social support	—	—	—	16	0.15 (-0.26 to 0.57)	0.469	—	—
stoplight approach	—	—	—	13	0.07 (-0.99 to 1.13)	0.897	—	—
stress management	—	—	—	2	0.12 (-0.34 to 0.59)	0.607	—	—
task-setting	—	—	—	3	0.27 (-0.07 to 0.6)	0.119	—	—

Appendix 7 Sensitivity analyses

Notes: The study performed several sensitivity analyses by Frequentist method including (1) sensitivity 1: using end-of-follow-up data instead of end-of-treatment data for analyses if different; (2) sensitivity 2: excluding trials with treatment duration of less than 24 weeks; (3) sensitivity 3: excluding trials with high risk of bias; (4) sensitivity 4: excluding trials with imbalanced lifestyle modification; and (5) sensitivity 5: splitting waiting list from minimal education as a separate treatment component.

P value reflects the statistical significance of the effect difference between components under the null hypothesis of no difference from the control (minimal education). BT, behavioural therapy; CBT, cognitive behavioural therapy; CT, cognitive therapy; ME, minimal education

7.1 Sensitivity analyses for conceptual level NMA

Outcome	Intervention	Comparator	Main estimates	Sensitivity 1	Sensitivity 2	Sensitivity 3	Sensitivity 4	Sensitivity 5
**BMI z-score**	BT	CBT	-0.01 (-0.05, 0.03)	-0.00 (-0.04, 0.04)	0.01 (-0.04, 0.07)	-0.01 (-0.06, 0.04)	0.00 (-0.04, 0.04)	-0.01 (-0.05, 0.03)
**BMI z-score**	BT	CT	-0.05 (-0.19, 0.09)	-0.06 (-0.21, 0.09)	-0.03 (-0.18, 0.12)	-0.08 (-0.28, 0.12)	-0.04 (-0.17, 0.09)	-0.05 (-0.19, 0.10)
**BMI z-score**	BT	EDU	-0.01 (-0.09, 0.06)	-0.02 (-0.09, 0.06)	-0.00 (-0.09, 0.08)	0.00 (-0.09, 0.10)	-0.01 (-0.07, 0.06)	-0.01 (-0.09, 0.06)
**BMI z-score**	BT	ME	**-0.08 (-0.10, -0.05)**	**-0.07 (-0.10, -0.04)**	**-0.08 (-0.11, -0.05)**	**-0.08 (-0.11, -0.05)**	**-0.06 (-0.08, -0.03)**	**-0.08 (-0.10, -0.05)**
**BMI z-score**	CBT	CT	-0.04 (-0.18, 0.10)	-0.06 (-0.20, 0.09)	-0.04 (-0.19, 0.10)	-0.07 (-0.26, 0.12)	-0.04 (-0.17, 0.09)	-0.04 (-0.18, 0.10)
**BMI z-score**	CBT	EDU	-0.00 (-0.08, 0.07)	-0.01 (-0.09, 0.07)	-0.02 (-0.10, 0.07)	0.02 (-0.08, 0.11)	-0.01 (-0.08, 0.06)	-0.01 (-0.08, 0.07)
**BMI z-score**	CBT	ME	**-0.07 (-0.10, -0.03)**	**-0.07 (-0.10, -0.03)**	**-0.09 (-0.15, -0.04)**	**-0.07 (-0.11, -0.03)**	**-0.06 (-0.09, -0.03)**	**-0.07 (-0.11, -0.03)**
**BMI z-score**	CT	EDU	0.03 (-0.11, 0.17)	0.04 (-0.10, 0.19)	0.03 (-0.12, 0.17)	0.09 (-0.13, 0.30)	0.03 (-0.10, 0.16)	0.03 (-0.11, 0.17)
**BMI z-score**	CT	ME	-0.03 (-0.17, 0.11)	-0.01 (-0.16, 0.13)	-0.05 (-0.20, 0.10)	-0.00 (-0.20, 0.20)	-0.02 (-0.15, 0.11)	-0.03 (-0.17, 0.11)
**BMI z-score**	EDU	ME	-0.06 (-0.14, 0.01)	-0.06 (-0.14, 0.02)	-0.08 (-0.16, 0.00)	-0.09 (-0.18, 0.01)	-0.05 (-0.12, 0.02)	-0.06 (-0.14, 0.01)
**Height**	BT	CBT	**1.19 (0.41, 1.97)**	**0.87 (0.18, 1.56)**	NA	**1.19 (0.41, 1.97)**	**1.41 (0.31, 2.51)**	**1.11 (0.35, 1.88)**
**Height**	BT	EDU	0.51 (-1.07, 2.08)	0.25 (-1.15, 1.66)	0.51 (-0.76, 1.78)	NA	NA	0.51 (-1.05, 2.06)
**Height**	BT	ME	0.28 (-0.09, 0.65)	0.02 (-0.30, 0.34)	**0.29 (0.01, 0.57)**	0.28 (-0.09, 0.65)	0.25 (-0.39, 0.88)	0.16 (-0.23, 0.56)
**Height**	CBT	EDU	-0.68 (-2.44, 1.08)	-0.62 (-2.18, 0.95)	NA	NA	NA	-0.61 (-2.34, 1.13)
**Height**	CBT	ME	**-0.91 (-1.60, -0.22)**	**-0.85 (-1.46, -0.24)**	NA	**-0.91 (-1.60, -0.22)**	**-1.16 (-2.06, -0.26)**	**-0.95 (-1.63, -0.27)**
**Height**	EDU	ME	-0.23 (-1.85, 1.39)	-0.23 (-1.67, 1.21)	-0.22 (-1.52, 1.08)	NA	NA	-0.34 (-1.95, 1.26)
**Body fat percentage**	BT	CBT	2.26 (-0.53, 5.05)	**3.43 (0.19, 6.68)**	1.87 (-1.12, 4.87)	2.93 (-0.89, 6.75)	**2.81 (0.89, 4.73)**	2.90 (-0.12, 5.92)
**Body fat percentage**	BT	ME	**-1.31 (-2.58, -0.05)**	-0.73 (-1.63, 0.17)	**-1.32 (-2.59, -0.04)**	**-1.33 (-2.62, -0.03)**	-0.38 (-1.00, 0.24)	**-1.63 (-3.01, -0.25)**
**Body fat percentage**	CBT	ME	**-3.57 (-6.06, -1.08)**	**-4.17 (-7.28, -1.05)**	**-3.19 (-5.90, -0.48)**	**-4.26 (-7.85, -0.67)**	**-3.19 (-5.01, -1.37)**	**-4.52 (-7.45, -1.60)**
**Waist circumference**	BT	CBT	0.06 (-1.37, 1.49)	-0.31 (-2.08, 1.46)	0.39 (-1.13, 1.91)	-0.11 (-1.78, 1.55)	0.36 (-1.28, 2.00)	0.19 (-1.30, 1.68)
**Waist circumference**	BT	EDU	-2.44 (-5.09, 0.20)	-1.99 (-5.17, 1.18)	-2.41 (-4.89, 0.07)	-1.03 (-5.78, 3.71)	-2.68 (-5.79, 0.43)	-2.44 (-5.10, 0.21)
**Waist circumference**	BT	ME	**-1.70 (-2.74, -0.67)**	**-1.83 (-3.14, -0.51)**	**-1.40 (-2.44, -0.35)**	**-1.65 (-2.73, -0.57)**	**-1.63 (-2.77, -0.48)**	**-1.72 (-2.75, -0.68)**
**Waist circumference**	CBT	EDU	-2.50 (-5.45, 0.44)	-1.68 (-5.23, 1.87)	-2.80 (-5.64, 0.05)	-0.92 (-5.83, 3.98)	-3.04 (-6.46, 0.39)	-2.64 (-5.62, 0.35)
**Waist circumference**	CBT	ME	**-1.76 (-2.86, -0.67)**	**-1.51 (-2.83, -0.19)**	**-1.78 (-2.95, -0.61)**	**-1.54 (-2.87, -0.21)**	**-1.99 (-3.24, -0.73)**	**-1.91 (-3.10, -0.72)**
**Waist circumference**	EDU	ME	0.74 (-2.03, 3.51)	0.17 (-3.17, 3.51)	1.01 (-1.61, 3.63)	-0.62 (-5.35, 4.11)	1.05 (-2.16, 4.26)	0.73 (-2.05, 3.51)
**Quality of life**	BT	CBT	-2.24 (-4.76, 0.28)	-2.16 (-5.21, 0.88)	-2.50 (-5.22, 0.22)	**-4.15 (-7.57, -0.74)**	-2.40 (-5.32, 0.52)	-1.96 (-4.53, 0.61)
**Quality of life**	BT	EDU	3.00 (-1.00, 7.01)	2.29 (-2.23, 6.80)	3.05 (-1.07, 7.17)	0.20 (-4.00, 4.40)	3.10 (-1.17, 7.38)	2.98 (-0.97, 6.93)
**Quality of life**	BT	ME	**2.50 (0.71, 4.30)**	**2.56 (0.65, 4.47)**	**2.57 (0.70, 4.43)**	**1.92 (0.36, 3.48)**	**2.61 (0.53, 4.68)**	**2.30 (0.46, 4.13)**
**Quality of life**	CBT	EDU	**5.24 (0.51, 9.97)**	4.45 (-1.00, 9.89)	**5.54 (0.61, 10.48)**	4.35 (-1.06, 9.77)	**5.50 (0.32, 10.68)**	**4.94 (0.23, 9.65)**
**Quality of life**	CBT	ME	**4.74 (2.47, 7.02)**	**4.72 (1.91, 7.54)**	**5.06 (2.55, 7.57)**	**6.08 (3.04, 9.11)**	**5.00 (2.33, 7.67)**	**4.26 (1.71, 6.80)**
**Quality of life**	EDU	ME	-0.50 (-4.89, 3.89)	0.27 (-4.63, 5.18)	-0.48 (-5.00, 4.04)	1.72 (-2.76, 6.21)	-0.50 (-5.25, 4.26)	-0.69 (-5.04, 3.67)
**Mental health**	BT	CBT	1.04 (-4.11, 6.18)	1.04 (-4.11, 6.18)	1.04 (-4.11, 6.18)	1.04 (-4.11, 6.18)	1.88 (-3.35, 7.11)	1.04 (-4.11, 6.18)
**Mental health**	BT	EDU	1.00 (-3.84, 5.84)	1.00 (-3.84, 5.84)	1.00 (-3.84, 5.84)	1.00 (-3.84, 5.84)	1.00 (-3.74, 5.74)	1.00 (-3.84, 5.84)
**Mental health**	BT	ME	1.00 (-4.07, 6.07)	1.00 (-4.07, 6.07)	1.00 (-4.07, 6.07)	1.00 (-4.07, 6.07)	1.00 (-3.98, 5.98)	1.00 (-4.07, 6.07)
**Mental health**	CBT	EDU	-0.04 (-6.03, 5.96)	-0.04 (-6.03, 5.96)	-0.04 (-6.03, 5.96)	-0.04 (-6.03, 5.96)	-0.88 (-6.95, 5.19)	-0.04 (-6.03, 5.96)
**Mental health**	CBT	ME	-0.04 (-0.93, 0.86)	-0.04 (-0.93, 0.86)	-0.04 (-0.93, 0.86)	-0.04 (-0.93, 0.86)	-0.88 (-2.50, 0.74)	-0.04 (-0.93, 0.86)
**Mental health**	EDU	ME	-0.00 (-5.93, 5.93)	-0.00 (-5.93, 5.93)	-0.00 (-5.93, 5.93)	-0.00 (-5.93, 5.93)	0.00 (-5.85, 5.85)	-0.00 (-5.93, 5.93)

7.2 Sensitivity analyses for technical level CNMA

7.2.1 BMI z-score change from baseline

Components	Main estimate		Sensitivity 1		Sensitivity 2		Sensitivity 3		Sensitivity 4		Sensitivity 5	
	No. of studies: 98		No. of studies: 98		No. of studies: 73		No. of studies: 76		No. of studies: 84		No. of studies:98	
	Mean difference (95% CI)	p value	Mean difference (95% CI)	p value	Mean difference (95% CI)	p value	Mean difference (95% CI)	p value	Mean difference (95% CI)	p value	Mean difference (95% CI)	p value
contracting	-0.05 (-0.12 to 0.03)	0.2173	-0.07 (-0.15 to 0.01)	0.1042	-0.08 (-0.18 to 0.02)	0.1146	-0.10 (-0.20 to 0.00)	0.0623	-0.11 (-0.19 to -0.02)	0.0179	-0.05 (-0.13 to 0.02)	0.1821
cognitive restructuring	-0.00 (-0.11 to 0.10)	0.9546	0.02 (-0.09 to 0.14)	0.6772	-0.04 (-0.23 to 0.16)	0.7142	-0.02 (-0.15 to 0.10)	0.7041	-0.05 (-0.16 to 0.07)	0.4381	-0.01 (-0.11 to 0.10)	0.9109
device-monitoring	0.00 (-0.06 to 0.07)	0.9651	0.02 (-0.05 to 0.08)	0.6436	0.01 (-0.10 to 0.11)	0.9106	-0.02 (-0.10 to 0.06)	0.5659	-0.01 (-0.09 to 0.06)	0.761	0.00 (-0.06 to 0.07)	0.97
feedback	0.02 (-0.02 to 0.07)	0.3252	0.02 (-0.02 to 0.07)	0.3465	0.00 (-0.06 to 0.06)	0.9922	0.03 (-0.02 to 0.09)	0.2528	0.02 (-0.03 to 0.07)	0.3938	0.02 (-0.02 to 0.07)	0.2835
functional behavioural analysis	0.02 (-0.05 to 0.09)	0.6057	-0.01 (-0.09 to 0.07)	0.8588	0.03 (-0.08 to 0.15)	0.5725	-0.00 (-0.09 to 0.09)	0.9675	0.05 (-0.03 to 0.12)	0.2494	0.02 (-0.05 to 0.09)	0.5357
group	-0.00 (-0.04 to 0.04)	0.8444	-0.01 (-0.05 to 0.04)	0.7776	-0.01 (-0.07 to 0.04)	0.6901	-0.02 (-0.07 to 0.03)	0.4205	0.00 (-0.04 to 0.04)	0.9984	-0.00 (-0.04 to 0.04)	0.9261
goal-setting	0.00 (-0.06 to 0.06)	0.9848	-0.01 (-0.08 to 0.06)	0.7792	0.03 (-0.06 to 0.11)	0.5467	-0.03 (-0.12 to 0.05)	0.4363	0.02 (-0.05 to 0.10)	0.5265	0.00 (-0.06 to 0.07)	0.8847
inhibition training	0.04 (-0.13 to 0.20)	0.6623	-0.03 (-0.19 to 0.14)	0.7399	0.04 (-0.20 to 0.27)	0.755	-0.08 (-0.32 to 0.15)	0.4914	0.02 (-0.14 to 0.19)	0.7849	0.04 (-0.13 to 0.20)	0.6668
modelling	0.01 (-0.05 to 0.07)	0.7793	0.03 (-0.04 to 0.09)	0.4134	0.02 (-0.08 to 0.11)	0.7532	0.04 (-0.05 to 0.12)	0.4138	0.04 (-0.04 to 0.12)	0.3376	0.01 (-0.05 to 0.07)	0.7764
motivation	-0.03 (-0.07 to 0.02)	0.2599	-0.02 (-0.07 to 0.02)	0.3141	-0.02 (-0.09 to 0.04)	0.4934	-0.01 (-0.07 to 0.05)	0.7448	-0.04 (-0.09 to 0.00)	0.0782	-0.03 (-0.07 to 0.02)	0.2652
parental involvement	**-0.09 (-0.16 to -0.03)**	0.0036	**-0.10 (-0.16 to -0.03)**	0.0057	**-0.13 (-0.22 to -0.04)**	0.004	**-0.14 (-0.22 to -0.05)**	0.0015	**-0.10 (-0.18 to -0.02)**	0.013	**-0.10 (-0.16 to -0.03)**	0.0032
preplanning	0.01 (-0.05 to 0.07)	0.8091	-0.01 (-0.07 to 0.06)	0.8844	0.01 (-0.08 to 0.10)	0.8606	-0.02 (-0.11 to 0.07)	0.6628	-0.02 (-0.10 to 0.05)	0.5635	0.01 (-0.05 to 0.07)	0.7756
problem solving	-0.00 (-0.05 to 0.04)	0.8547	-0.00 (-0.05 to 0.04)	0.8486	-0.01 (-0.07 to 0.05)	0.7147	0.02 (-0.04 to 0.08)	0.4452	-0.02 (-0.07 to 0.03)	0.3731	-0.01 (-0.05 to 0.04)	0.7893
psychoeducation	0.02 (-0.05 to 0.10)	0.523	0.04 (-0.04 to 0.12)	0.3654	0.04 (-0.06 to 0.15)	0.4501	0.06 (-0.04 to 0.16)	0.2188	0.04 (-0.06 to 0.14)	0.3922	0.02 (-0.05 to 0.10)	0.5339
remote	-0.03 (-0.09 to 0.03)	0.3211	-0.04 (-0.10 to 0.02)	0.1787	-0.01 (-0.09 to 0.07)	0.8055	-0.03 (-0.10 to 0.05)	0.4976	-0.04 (-0.10 to 0.02)	0.2439	-0.03 (-0.09 to 0.03)	0.3471
reinforcement	-0.04 (-0.10 to 0.02)	0.1742	-0.03 (-0.09 to 0.04)	0.4025	-0.06 (-0.14 to 0.03)	0.194	-0.03 (-0.10 to 0.05)	0.4738	-0.03 (-0.10 to 0.04)	0.3403	-0.04 (-0.10 to 0.02)	0.1769
reminders	0.02 (-0.04 to 0.08)	0.4813	0.03 (-0.03 to 0.10)	0.3567	0.01 (-0.07 to 0.08)	0.8257	0.02 (-0.06 to 0.10)	0.6271	0.01 (-0.06 to 0.08)	0.8622	0.02 (-0.04 to 0.08)	0.49
role playing	0.00 (-0.12 to 0.13)	0.951	0.03 (-0.11 to 0.17)	0.6332	0.03 (-0.13 to 0.20)	0.6843	-0.02 (-0.17 to 0.13)	0.8199	-0.05 (-0.18 to 0.09)	0.4915	0.01 (-0.12 to 0.13)	0.8895
relaxation training	0.16 (-0.00 to 0.32)	0.0564	0.13 (-0.04 to 0.30)	0.1276	0.21 (-0.08 to 0.50)	0.1612	0.17 (-0.04 to 0.39)	0.1126	**0.19 (0.01 to 0.36)**	0.0392	0.16 (-0.00 to 0.32)	0.0561
rule-setting	0.00 (-0.08 to 0.09)	0.9417	0.01 (-0.08 to 0.10)	0.8845	-0.01 (-0.14 to 0.11)	0.8387	-0.01 (-0.12 to 0.10)	0.8657	-0.00 (-0.10 to 0.09)	0.9248	-0.00 (-0.09 to 0.08)	0.9404
stimulus control	**-0.07 (-0.12 to -0.01)**	0.0296	**-0.08 (-0.14 to -0.01)**	0.02	-0.07 (-0.16 to 0.02)	0.1282	-0.06 (-0.13 to 0.02)	0.128	-0.07 (-0.14 to 0.00)	0.0595	**-0.07 (-0.13 to -0.01)**	0.0256
self-concept improvement	0.04 (-0.01 to 0.10)	0.1082	0.03 (-0.03 to 0.08)	0.3767	0.04 (-0.04 to 0.12)	0.3569	0.05 (-0.02 to 0.11)	0.1869	0.03 (-0.03 to 0.09)	0.3354	0.04 (-0.02 to 0.09)	0.1667
serious games	-0.03 (-0.09 to 0.03)	0.3385	-0.05 (-0.11 to 0.01)	0.1161	0.03 (-0.06 to 0.13)	0.5068	-0.02 (-0.10 to 0.06)	0.651	-0.03 (-0.11 to 0.05)	0.4786	-0.03 (-0.08 to 0.03)	0.3874
self-monitoring	0.02 (-0.03 to 0.07)	0.4726	0.02 (-0.03 to 0.07)	0.4664	0.00 (-0.06 to 0.07)	0.9033	0.03 (-0.03 to 0.09)	0.2913	0.03 (-0.03 to 0.08)	0.346	0.01 (-0.04 to 0.07)	0.6228
social support	-0.02 (-0.08 to 0.04)	0.5222	0.01 (-0.06 to 0.07)	0.8539	-0.01 (-0.09 to 0.07)	0.8148	-0.02 (-0.09 to 0.05)	0.5868	-0.01 (-0.09 to 0.06)	0.7258	-0.02 (-0.08 to 0.04)	0.5245
stoplight approach	0.05 (-0.01 to 0.12)	0.104	0.03 (-0.03 to 0.10)	0.3297	0.05 (-0.03 to 0.14)	0.2456	0.05 (-0.03 to 0.13)	0.2221	**0.10 (0.01 to 0.19)**	0.0237	0.05 (-0.01 to 0.12)	0.13
stress management	0.03 (-0.06 to 0.11)	0.5339	0.05 (-0.04 to 0.14)	0.2414	-0.02 (-0.15 to 0.12)	0.8232	0.00 (-0.12 to 0.13)	0.9378	0.02 (-0.08 to 0.12)	0.6844	0.02 (-0.06 to 0.10)	0.6403
task-setting	-0.00 (-0.06 to 0.05)	0.8778	-0.01 (-0.08 to 0.05)	0.676	-0.01 (-0.10 to 0.07)	0.7945	0.02 (-0.06 to 0.10)	0.5952	-0.01 (-0.08 to 0.06)	0.737	-0.01 (-0.07 to 0.05)	0.8136
third-wave components	-0.12 (-0.26 to 0.01)	0.0812	-0.14 (-0.29 to 0.01)	0.0651	-0.04 (-0.39 to 0.32)	0.837	-0.08 (-0.24 to 0.07)	0.2863	-0.12 (-0.26 to 0.02)	0.0918	-0.12 (-0.26 to 0.02)	0.0816
wait-list	—	—	—	—	—	—	—	—	—	—	-0.02 (-0.10 to 0.05)	0.5318

7.2.2 Height change from baseline

Components	Main estimate		Sensitivity 1		Sensitivity 2		Sensitivity 3		Sensitivity 4		Sensitivity 5	
	No. of studies: 27		No. of studies: 27		No. of studies: 19		No. of studies: 25		No. of studies: 19		No. of studies: 27	
	Mean difference (95% CI)	p value	Mean difference (95% CI)	p value	Mean difference (95% CI)	p value	Mean difference (95% CI)	p value	Mean difference (95% CI)	p value	Mean difference (95% CI)	p value
contracting	-1.22 (-3.75 to 1.30)	0.3415	-0.04 (-2.62 to 2.53)	0.9739	-1.27 (-4.06 to 1.52)	0.3726	-1.79 (-5.10 to 1.52)	0.2891	0.93 (-1.75 to 3.60)	0.4978	-2.43 (-6.41 to 1.56)	0.2328
cognitive restructuring	**-5.59 (-9.78 to -1.40)**	0.0089	**-6.22 (-10.39 to -2.05)**	0.0034	—	—	**-5.15 (-10.20 to -0.10)**	0.0455	-3.53 (-9.54 to 2.47)	0.2485	**-6.67 (-11.70 to -1.64)**	0.0093
device-monitoring	**1.46 (0.20 to 2.72)**	0.0233	**1.36 (0.09 to 2.64)**	0.0365	2.83 (-0.96 to 6.62)	0.143	1.52 (-0.82 to 3.87)	0.2025	1.60 (-5.59 to 8.79)	0.6629	**2.04 (0.08 to 4.00)**	0.0411
feedback	-0.26 (-1.59 to 1.08)	0.7042	-0.45 (-1.66 to 0.76)	0.4683	-0.88 (-2.72 to 0.96)	0.349	0.10 (-2.53 to 2.74)	0.938	-0.03 (-6.18 to 6.13)	0.9932	-0.64 (-2.30 to 1.02)	0.4477
functional behavioural analysis	-0.25 (-2.05 to 1.54)	0.7836	0.85 (-1.35 to 3.04)	0.4503	0.52 (-0.55 to 1.59)	0.3381	-0.82 (-3.88 to 2.23)	0.5976			-1.04 (-3.74 to 1.66)	0.4514
group	1.28 (-0.19 to 2.75)	0.0887	**1.59 (0.09 to 3.10)**	0.0379	**2.00 (0.02 to 3.98)**	0.0479	1.00 (-1.66 to 3.66)	0.4614	2.00 (-0.08 to 4.08)	0.0589	1.47 (-0.08 to 3.02)	0.0634
goal-setting	-0.45 (-1.82 to 0.92)	0.5192	-0.24 (-1.87 to 1.38)	0.7692	0.11 (-3.78 to 4.01)	0.9539	-0.15 (-2.23 to 1.94)	0.8906	-0.65 (-7.80 to 6.50)	0.8585	-1.45 (-4.36 to 1.46)	0.3285
inhibition training	—	—	—	—	—	—	—	—	—	—	—	—
modelling	0.90 (-1.30 to 3.11)	0.4226	0.08 (-2.24 to 2.41)	0.943	2.35 (-1.77 to 6.47)	0.2643	1.73 (-2.98 to 6.44)	0.4711	0.77 (-3.21 to 4.75)	0.7042	2.57 (-2.25 to 7.39)	0.2956
motivation	-0.42 (-2.02 to 1.18)	0.609	-0.40 (-2.07 to 1.27)	0.641	-0.13 (-1.80 to 1.55)	0.8836	0.31 (-2.65 to 3.27)	0.8379	-1.68 (-3.90 to 0.55)	0.1395	-0.69 (-2.44 to 1.06)	0.4394
parental involvement	-0.02 (-0.55 to 0.50)	0.9252	-0.05 (-0.57 to 0.48)	0.8638	-1.94 (-6.70 to 2.81)	0.4229	0.00 (-0.52 to 0.52)	1	-0.00 (-0.81 to 0.81)	1	-0.03 (-0.55 to 0.49)	0.9034
preplanning	0.95 (-1.01 to 2.91)	0.3429	0.48 (-1.54 to 2.51)	0.6382	0.33 (-1.14 to 1.80)	0.6626	1.17 (-2.20 to 4.54)	0.4979	0.12 (-4.81 to 5.06)	0.9608	0.97 (-0.99 to 2.92)	0.3341
problem solving	-0.38 (-1.51 to 0.75)	0.5109	-0.49 (-1.66 to 0.69)	0.4155	-0.50 (-2.59 to 1.59)	0.6397	-0.51 (-2.34 to 1.32)	0.5856	-0.50 (-2.68 to 1.68)	0.6534	-0.12 (-1.43 to 1.20)	0.864
psychoeducation	0.94 (-1.23 to 3.12)	0.3955	0.69 (-1.72 to 3.10)	0.5744	2.43 (-0.83 to 5.69)	0.1437	0.75 (-2.13 to 3.63)	0.6092	1.25 (-5.98 to 8.48)	0.7348	1.68 (-1.20 to 4.56)	0.2534
remote	-0.23 (-1.39 to 0.93)	0.6998	0.01 (-1.20 to 1.22)	0.9874	1.00 (-1.25 to 3.25)	0.3831	-0.40 (-2.23 to 1.43)	0.6657	1.00 (-1.33 to 3.33)	0.4003	-0.00 (-1.30 to 1.29)	0.9949
reinforcement	-1.17 (-2.37 to 0.04)	0.058	-1.06 (-2.27 to 0.16)	0.0893	-2.48 (-6.50 to 1.54)	0.227	-1.23 (-3.54 to 1.07)	0.2947	-1.30 (-8.45 to 5.85)	0.7215	-1.76 (-3.69 to 0.18)	0.0755
reminders	1.31 (-0.36 to 2.99)	0.1243	0.90 (-0.91 to 2.70)	0.3295	1.58 (-3.87 to 7.02)	0.57	1.85 (-1.12 to 4.81)	0.2223	0.40 (-7.56 to 8.36)	0.9215	1.41 (-0.29 to 3.10)	0.1032
role playing	-3.02 (-6.14 to 0.09)	0.0574	-2.09 (-5.32 to 1.13)	0.2027	-4.60 (-11.40 to 2.20)	0.1846	-3.27 (-8.00 to 1.47)	0.176	-0.20 (-12.00 to 11.60)	0.9735	-4.84 (-10.46 to 0.77)	0.091
relaxation training	—	—	—	—	—	—	—	—	—	—	—	—
rule-setting	1.17 (-0.93 to 3.27)	0.2759	0.53 (-1.45 to 2.51)	0.6	1.76 (-1.65 to 5.16)	0.3117	1.05 (-1.85 to 3.95)	0.4778	-0.27 (-2.95 to 2.40)	0.8412	1.97 (-0.97 to 4.90)	0.1892
stimulus control	-0.60 (-2.34 to 1.13)	0.4949	-0.17 (-1.98 to 1.63)	0.8512	-1.23 (-4.70 to 2.25)	0.4887	-1.15 (-4.04 to 1.73)	0.4329	-2.82 (-11.14 to 5.49)	0.5061	-1.05 (-3.12 to 1.03)	0.3225
self-concept improvement	-0.75 (-3.65 to 2.14)	0.6098	-1.62 (-5.25 to 2.00)	0.3805	—	—	-1.05 (-4.64 to 2.54)	0.5654	**-2.68 (-4.21 to -1.14)**	0.0006	0.69 (-4.01 to 5.40)	0.7734
serious games	-0.37 (-1.46 to 0.71)	0.5008	-0.92 (-2.06 to 0.21)	0.1104	-1.41 (-3.27 to 0.45)	0.1366	-0.32 (-1.53 to 0.90)	0.6076	-0.39 (-2.45 to 1.68)	0.7137	-0.28 (-1.39 to 0.84)	0.627
self-monitoring	-0.18 (-2.24 to 1.88)	0.8652	1.05 (-1.86 to 3.97)	0.4792	0.45 (-1.87 to 2.77)	0.7027	-0.33 (-3.13 to 2.47)	0.8173	1.89 (-9.95 to 13.72)	0.7548	-0.84 (-3.50 to 1.83)	0.5386
social support	-0.91 (-2.86 to 1.04)	0.3598	-1.08 (-2.91 to 0.75)	0.2463	-1.02 (-3.15 to 1.11)	0.348	-0.95 (-4.83 to 2.93)	0.6301	-1.87 (-8.16 to 4.41)	0.5591	-0.73 (-2.73 to 1.27)	0.4741
stoplight approach	1.45 (-0.71 to 3.61)	0.187	0.62 (-2.03 to 3.28)	0.6457	2.50 (-0.17 to 5.18)	0.0665	1.32 (-1.12 to 3.77)	0.2884	0.84 (-1.58 to 3.25)	0.4971	3.21 (-1.79 to 8.22)	0.2084
stress management	-0.25 (-2.05 to 1.54)	0.7836	0.85 (-1.35 to 3.04)	0.4503	0.52 (-0.55 to 1.59)	0.3381	-0.82 (-3.88 to 2.23)	0.5976	—	—	-1.04 (-3.74 to 1.66)	0.4514
task-setting	-1.31 (-2.77 to 0.15)	0.0784	**-1.75 (-3.29 to -0.21)**	0.0255	**-3.39 (-6.64 to -0.14)**	0.0408	-1.08 (-3.29 to 1.12)	0.3364	-2.54 (-9.78 to 4.71)	0.4927	-1.62 (-3.28 to 0.04)	0.0559
third-wave components	—	—	—	—	—	—	—	—	—	—	—	—
wait-list	—	—	—	—	—	—	—	—	—	—	1.35 (-2.11 to 4.81)	0.4451

7.2.3 Body fat (%) change from baseline

**Components**	**Main estimate**		**Sensitivity 1**		**Sensitivity 2**		**Sensitivity 3**		**Sensitivity 4**		**Sensitivity 5**	
	No. of studies: 36		No. of studies: 36		No. of studies: 29		No. of studies: 30		No. of studies: 26		No. of studies: 36	
	Mean difference (95% CI)	p value	Mean difference (95% CI)	p value	Mean difference (95% CI)	p value	Mean difference (95% CI)	p value	Mean difference (95% CI)	p value	Mean difference (95% CI)	p value
contracting	1.97 (-2.55 to 6.49)	0.3931	2.95 (-1.77 to 7.67)	0.2204	5.16 (-1.69 to 12.01)	0.14	-1.28 (-9.29 to 6.73)	0.7543	3.42 (-0.71 to 7.54)	0.1047	0.73 (-4.27 to 5.74)	0.7744
cognitive restructuring	-3.67 (-7.94 to 0.61)	0.0928	**-6.94 (-11.52 to -2.36)**	0.003	-4.90 (-10.82 to 1.03)	0.1052	-4.20 (-9.47 to 1.06)	0.1174	**-7.47 (-11.31 to -3.62)**	0.0001	-2.17 (-7.14 to 2.80)	0.3922
device-monitoring	**3.69 (0.56 to 6.83)**	0.0209	**5.17 (1.54 to 8.81)**	0.0053	**4.53 (0.71 to 8.35)**	0.0202	3.16 (-2.37 to 8.68)	0.2624	2.13 (-0.14 to 4.40)	0.0664	**3.29 (0.04 to 6.54)**	0.0471
feedback	**-2.73 (-5.31 to -0.14)**	0.0387	**-3.16 (-6.22 to -0.09)**	0.0439	-3.58 (-7.22 to 0.05)	0.0534	-2.28 (-5.62 to 1.07)	0.1819	**-3.49 (-5.80 to -1.19)**	0.003	**-2.68 (-5.31 to -0.06)**	0.0453
functional behavioural analysis	1.42 (-1.14 to 3.97)	0.2775	1.63 (-1.07 to 4.33)	0.2366	1.87 (-1.90 to 5.65)	0.3305	0.17 (-3.28 to 3.63)	0.9216	**4.22 (2.60 to 5.84)**	0	1.13 (-1.51 to 3.76)	0.4025
group	1.09 (-0.58 to 2.76)	0.2003	1.43 (-0.33 to 3.18)	0.1123	1.33 (-0.61 to 3.27)	0.178	-0.12 (-3.31 to 3.06)	0.9393	**3.02 (1.73 to 4.32)**	0	1.57 (-0.30 to 3.43)	0.0993
goal-setting	0.26 (-1.99 to 2.50)	0.8212	-1.69 (-4.47 to 1.10)	0.2349	1.03 (-2.51 to 4.56)	0.5692	0.77 (-2.10 to 3.64)	0.5982	**3.42 (0.64 to 6.19)**	0.0158	1.27 (-1.54 to 4.08)	0.3756
inhibition training	—	—	—	—	—	—	—	—	—	—	—	—
modelling	0.25 (-3.04 to 3.55)	0.8794	-0.23 (-4.04 to 3.57)	0.9038	0.29 (-4.65 to 5.24)	0.9077	1.09 (-3.44 to 5.62)	0.6366	**10.95 (3.00 to 18.90)**	0.0069	0.87 (-2.62 to 4.36)	0.6253
motivation	-1.06 (-2.70 to 0.57)	0.2008	-0.76 (-2.51 to 0.99)	0.3967	-0.69 (-2.89 to 1.51)	0.5409	0.19 (-3.20 to 3.57)	0.9144	**-2.76 (-3.88 to -1.64)**	0	-0.97 (-2.63 to 0.70)	0.2559
parental involvement	-0.55 (-2.85 to 1.74)	0.6353	0.06 (-2.18 to 2.30)	0.9563	-1.48 (-4.21 to 1.25)	0.2885	-1.50 (-4.80 to 1.79)	0.3708	-0.64 (-1.44 to 0.16)	0.1191	-0.48 (-2.81 to 1.86)	0.6879
preplanning	**-3.05 (-5.82 to -0.28)**	0.0311	-0.99 (-3.85 to 1.88)	0.5003	-3.94 (-8.22 to 0.35)	0.0719	-2.77 (-5.79 to 0.26)	0.0735	-1.33 (-3.94 to 1.27)	0.3157	-2.16 (-5.32 to 0.99)	0.1784
problem solving	**2.90 (0.30 to 5.50)**	0.0291	**3.96 (0.87 to 7.04)**	0.0119	2.87 (-0.30 to 6.04)	0.0758	2.79 (-0.35 to 5.94)	0.0819	0.81 (-0.97 to 2.59)	0.3743	2.63 (-0.04 to 5.31)	0.0538
psychoeducation	-1.09 (-3.56 to 1.38)	0.3886	-1.34 (-3.90 to 1.22)	0.3047	-0.86 (-4.42 to 2.71)	0.6377	-0.59 (-4.20 to 3.02)	0.7483	-1.39 (-3.09 to 0.31)	0.1094	-1.46 (-4.04 to 1.13)	0.2691
remote	-1.80 (-3.94 to 0.33)	0.098	-1.56 (-3.74 to 0.63)	0.1623	-1.44 (-4.09 to 1.21)	0.2863	-0.74 (-5.88 to 4.41)	0.7788	-0.02 (-1.66 to 1.62)	0.9805	-1.39 (-3.66 to 0.89)	0.2318
reinforcement	-1.25 (-3.76 to 1.25)	0.3255	-1.68 (-4.24 to 0.88)	0.1981	-0.89 (-4.54 to 2.75)	0.6309	-1.77 (-5.59 to 2.06)	0.3655	**-3.69 (-4.98 to -2.40)**	0	-1.18 (-3.72 to 1.37)	0.3648
reminders	1.44 (-1.38 to 4.26)	0.318	1.68 (-1.42 to 4.78)	0.2884	1.79 (-1.42 to 5.00)	0.2746	0.06 (-4.49 to 4.61)	0.9785	1.72 (-0.09 to 3.54)	0.0627	1.21 (-1.67 to 4.10)	0.4106
role playing	0.78 (-5.48 to 7.05)	0.807	1.93 (-4.62 to 8.48)	0.5631	—	—	1.56 (-6.13 to 9.25)	0.6907	**10.43 (3.18 to 17.68)**	0.0048	0.27 (-6.14 to 6.68)	0.9339
relaxation training	**8.08 (0.82 to 15.34)**	0.0292	**10.14 (2.43 to 17.85)**	0.0099	**18.60 (1.71 to 35.49)**	0.0309	5.43 (-4.15 to 15.01)	0.2666	**16.97 (10.06 to 23.89)**	0	5.56 (-2.86 to 13.98)	0.1953
rule-setting	**4.32 (0.16 to 8.47)**	0.0416	**4.53 (0.20 to 8.86)**	0.0405	4.39 (-0.62 to 9.40)	0.0859	2.81 (-3.93 to 9.55)	0.4135	**4.61 (2.23 to 6.99)**	0.0001	3.31 (-1.21 to 7.83)	0.1513
stimulus control	2.66 (-1.12 to 6.44)	0.1675	3.48 (-0.53 to 7.49)	0.0893	6.79 (-0.26 to 13.84)	0.0592	2.22 (-2.24 to 6.67)	0.3294	**5.30 (0.46 to 10.15)**	0.032	1.11 (-3.48 to 5.69)	0.6359
self-concept improvement	1.20 (-1.73 to 4.14)	0.4215	0.72 (-2.35 to 3.78)	0.6469	2.45 (-3.97 to 8.87)	0.4549	-0.57 (-4.90 to 3.76)	0.7975	**4.90 (2.40 to 7.39)**	0.0001	0.24 (-3.12 to 3.60)	0.8879
serious games	-1.91 (-4.27 to 0.44)	0.1115	-2.35 (-5.06 to 0.36)	0.0896	-2.47 (-5.37 to 0.43)	0.0956	-2.49 (-5.24 to 0.26)	0.0765	-4.07 (-10.66 to 2.52)	0.2261	-1.86 (-4.26 to 0.54)	0.1281
self-monitoring	-2.51 (-5.96 to 0.95)	0.1555	-2.76 (-6.52 to 0.99)	0.149	-6.29 (-13.16 to 0.57)	0.0723	-0.77 (-6.39 to 4.86)	0.7896	**-8.70 (-14.93 to -2.47)**	0.0062	-3.19 (-6.88 to 0.50)	0.0903
social support	-0.14 (-3.15 to 2.87)	0.9251	0.29 (-3.12 to 3.70)	0.8658	0.07 (-4.37 to 4.50)	0.9759	-0.96 (-4.78 to 2.86)	0.622	**-6.09 (-11.19 to -1.00)**	0.0191	-1.00 (-4.35 to 2.35)	0.5586
stoplight approach	-1.38 (-5.62 to 2.86)	0.5244	-1.86 (-6.51 to 2.78)	0.4321	-4.02 (-10.57 to 2.53)	0.229	1.67 (-6.30 to 9.65)	0.6813	0.98 (-3.16 to 5.12)	0.6423	-0.69 (-5.14 to 3.76)	0.7611
stress management	2.70 (-0.63 to 6.03)	0.1127	2.60 (-0.90 to 6.10)	0.146	1.95 (-2.97 to 6.86)	0.4373	1.32 (-4.46 to 7.10)	0.655	-0.66 (-3.38 to 2.07)	0.6368	2.77 (-0.62 to 6.16)	0.1087
task-setting	1.36 (-1.43 to 4.16)	0.3386	**3.37 (0.43 to 6.31)**	0.0249	0.54 (-3.70 to 4.78)	0.8026	2.14 (-1.43 to 5.71)	0.2398	1.59 (-1.76 to 4.94)	0.3517	1.16 (-1.69 to 4.02)	0.425
third-wave components	—	—	—	—	—	—	—	—	—	—	—	—
wait-list	—	—	—	—	—	—	—	—	—	—	-2.91 (-7.65 to 1.83)	0.2285

7.2.4 Waist circumference change from baseline

Components	Main estimate		Sensitivity 1		Sensitivity 2		Sensitivity 3		Sensitivity 4		Sensitivity 5	
	No. of studies: 44		No. of studies: 44		No. of studies: 34		No. of studies: 35		No. of studies: 33		No. of studies: 44	
	Mean difference (95% CI)	p value	Mean difference (95% CI)	p value	Mean difference (95% CI)	p value	Mean difference (95% CI)	p value	Mean difference (95% CI)	p value	Mean difference (95% CI)	p value
contracting	0.11 (-3.60 to 3.83)	0.9518	0.62 (-3.63 to 4.88)	0.7739	-0.16 (-7.28 to 6.97)	0.9659	0.33 (-6.33 to 7.00)	0.9215	-7.15 (-23.42 to 9.13)	0.3894	0.11 (-3.67 to 3.88)	0.9556
cognitive restructuring	3.80 (-0.09 to 7.68)	0.0553	3.03 (-1.26 to 7.31)	0.1661	2.67 (-8.18 to 13.52)	0.6297	**4.25 (0.12 to 8.38)**	0.0435	-0.16 (-11.67 to 11.34)	0.9776	3.83 (-0.29 to 7.94)	0.0685
device-monitoring	1.48 (-2.16 to 5.11)	0.426	1.67 (-2.30 to 5.64)	0.4096	0.29 (-6.62 to 7.19)	0.9354	0.81 (-3.16 to 4.78)	0.6891	2.64 (-8.12 to 13.39)	0.6309	1.52 (-2.46 to 5.49)	0.4541
feedback	1.15 (-1.11 to 3.41)	0.3204	1.57 (-0.82 to 3.97)	0.1983	2.13 (-4.58 to 8.85)	0.5335	2.11 (-0.45 to 4.67)	0.1059	-1.59 (-7.22 to 4.05)	0.5812	1.15 (-1.15 to 3.46)	0.3275
functional behavioural analysis	-1.89 (-4.80 to 1.03)	0.2045	-0.76 (-4.21 to 2.69)	0.6665	-1.42 (-6.51 to 3.68)	0.5861	-1.52 (-4.30 to 1.26)	0.2848	1.19 (-5.46 to 7.84)	0.7259	-1.88 (-4.85 to 1.08)	0.2135
group	-2.02 (-4.25 to 0.20)	0.0741	-0.81 (-3.20 to 1.57)	0.5038	-2.42 (-6.16 to 1.32)	0.2044	**-4.72 (-7.58 to -1.86)**	0.0012	-1.28 (-5.81 to 3.26)	0.5806	-2.03 (-4.31 to 0.25)	0.0809
goal-setting	-1.98 (-4.64 to 0.68)	0.1454	-0.85 (-3.84 to 2.15)	0.5785	-1.02 (-9.40 to 7.35)	0.8109	**-3.25 (-6.42 to -0.07)**	0.0451	2.33 (-8.13 to 12.80)	0.6619	-1.98 (-4.69 to 0.73)	0.1514
inhibition training	-1.48 (-7.97 to 5.00)	0.6538	-1.51 (-8.79 to 5.78)	0.6856	-0.67 (-18.08 to 16.73)	0.9395	-2.58 (-10.42 to 5.26)	0.5185	1.42 (-19.56 to 22.41)	0.8943	-1.49 (-8.08 to 5.10)	0.6579
modelling	1.20 (-2.28 to 4.69)	0.498	1.05 (-2.48 to 4.58)	0.5588	-2.15 (-15.62 to 11.32)	0.7543	1.55 (-3.32 to 6.42)	0.5325	6.44 (-5.74 to 18.63)	0.2999	1.20 (-2.34 to 4.75)	0.5055
motivation	-1.39 (-3.47 to 0.69)	0.1909	-0.62 (-2.61 to 1.38)	0.5448	-1.16 (-5.01 to 2.70)	0.5567	-1.09 (-4.22 to 2.04)	0.4959	-2.58 (-7.26 to 2.10)	0.2804	-1.40 (-3.57 to 0.78)	0.2078
parental involvement	0.10 (-2.13 to 2.32)	0.9305	0.22 (-2.48 to 2.93)	0.8718	-0.45 (-4.03 to 3.14)	0.8069	0.04 (-2.44 to 2.53)	0.9718	-2.09 (-7.24 to 3.06)	0.4269	0.10 (-2.16 to 2.36)	0.9326
preplanning	-0.46 (-3.57 to 2.65)	0.7718	0.16 (-3.24 to 3.56)	0.9261	-2.25 (-9.50 to 5.00)	0.5427	-1.04 (-4.83 to 2.75)	0.5902	-0.35 (-9.98 to 9.29)	0.9439	-0.49 (-3.81 to 2.82)	0.7699
problem solving	-1.18 (-3.78 to 1.42)	0.3725	-0.74 (-3.70 to 2.22)	0.6256	-0.46 (-4.94 to 4.02)	0.8402	-1.49 (-4.42 to 1.43)	0.3167	-1.69 (-8.25 to 4.86)	0.6128	-1.21 (-3.93 to 1.52)	0.3853
psychoeducation	1.18 (-1.89 to 4.25)	0.4513	-0.40 (-3.18 to 2.37)	0.7746	1.37 (-3.31 to 6.06)	0.5651	1.19 (-2.80 to 5.18)	0.5588	2.28 (-3.99 to 8.55)	0.4756	1.19 (-1.96 to 4.35)	0.4585
remote	-0.11 (-2.63 to 2.42)	0.9323	0.01 (-2.86 to 2.87)	0.9952	-0.60 (-5.31 to 4.12)	0.8046	1.18 (-2.28 to 4.63)	0.5051	-0.50 (-4.38 to 3.37)	0.7987	-0.12 (-2.68 to 2.45)	0.9287
reinforcement	1.43 (-1.93 to 4.80)	0.4043	0.26 (-3.77 to 4.28)	0.8996	0.67 (-5.20 to 6.53)	0.8233	-0.45 (-5.83 to 4.93)	0.8692	1.21 (-8.54 to 10.96)	0.8081	1.45 (-1.99 to 4.89)	0.4082
reminders	-1.80 (-5.24 to 1.64)	0.3053	-1.47 (-5.29 to 2.35)	0.4517	-1.36 (-7.24 to 4.51)	0.6489	-2.25 (-6.74 to 2.24)	0.3256	-0.85 (-9.08 to 7.38)	0.8394	-1.80 (-5.29 to 1.70)	0.3138
role playing	-3.71 (-11.02 to 3.61)	0.3209	-4.23 (-12.60 to 4.15)	0.3229	—	—	-4.28 (-11.60 to 3.05)	0.2523	-1.48 (-13.88 to 10.91)	0.8145	-3.70 (-11.13 to 3.74)	0.3299
relaxation training	-5.50 (-12.46 to 1.46)	0.1214	-3.25 (-10.94 to 4.44)	0.4075	-4.28 (-21.45 to 12.88)	0.6248	-5.05 (-13.06 to 2.95)	0.2161	-3.03 (-19.56 to 13.49)	0.7191	-5.52 (-12.65 to 1.60)	0.1286
rule-setting	-0.95 (-7.26 to 5.36)	0.7679	-0.54 (-7.02 to 5.94)	0.8701	-3.15 (-20.06 to 13.76)	0.7149	0.50 (-6.94 to 7.93)	0.8955	-1.20 (-16.62 to 14.22)	0.8787	-0.94 (-7.35 to 5.46)	0.7728
stimulus control	-0.42 (-3.04 to 2.20)	0.753	-0.50 (-3.60 to 2.60)	0.7509	-1.44 (-9.43 to 6.55)	0.7234	0.29 (-2.69 to 3.27)	0.8498	2.01 (-11.05 to 15.07)	0.763	-0.43 (-3.10 to 2.24)	0.7516
self-concept improvement	-1.99 (-4.42 to 0.45)	0.1101	-2.22 (-4.95 to 0.52)	0.1127	-1.32 (-9.32 to 6.69)	0.7473	-0.83 (-3.99 to 2.33)	0.6069	-5.99 (-17.20 to 5.22)	0.2952	-1.98 (-4.52 to 0.56)	0.1275
serious games	1.56 (-0.92 to 4.05)	0.2169	0.05 (-2.69 to 2.79)	0.9707	3.31 (-7.09 to 13.70)	0.533	**2.73 (0.07 to 5.39)**	0.0441	8.01 (-10.33 to 26.36)	0.3921	1.58 (-0.98 to 4.15)	0.2259
self-monitoring	0.33 (-2.61 to 3.28)	0.8258	-0.29 (-3.59 to 3.00)	0.8611	1.54 (-4.22 to 7.30)	0.5994	1.92 (-1.63 to 5.46)	0.2895	-1.57 (-9.71 to 6.57)	0.7047	0.34 (-2.69 to 3.38)	0.8237
social support	2.03 (-0.53 to 4.58)	0.1201	1.83 (-1.23 to 4.90)	0.2407	2.08 (-4.14 to 8.30)	0.5122	**3.70 (0.17 to 7.23)**	0.0399	2.22 (-5.04 to 9.48)	0.5491	2.03 (-0.56 to 4.63)	0.1242
stoplight approach	-0.81 (-4.40 to 2.78)	0.6573	-1.40 (-5.90 to 3.10)	0.542	-3.23 (-9.95 to 3.49)	0.3462	-0.49 (-5.11 to 4.13)	0.8344	-1.20 (-15.54 to 13.14)	0.8696	-0.80 (-4.47 to 2.86)	0.6668
stress management	1.77 (-1.90 to 5.44)	0.3446	1.53 (-2.84 to 5.91)	0.4915	2.89 (-4.87 to 10.65)	0.4652	1.88 (-2.16 to 5.93)	0.3616	-1.10 (-12.49 to 10.30)	0.8503	1.76 (-1.97 to 5.49)	0.354
task-setting	-2.24 (-4.82 to 0.34)	0.0888	-3.35 (-6.26 to -0.45)	0.0235	-1.38 (-11.12 to 8.37)	0.7818	-1.33 (-3.76 to 1.10)	0.2828	-4.95 (-14.48 to 4.57)	0.308	-2.25 (-4.92 to 0.41)	0.0974
third-wave components	-3.20 (-13.18 to 6.79)	0.5301	-2.41 (-14.02 to 9.20)	0.6843	0.95 (-25.53 to 27.43)	0.9439	-4.87 (-16.39 to 6.64)	0.4068	-4.38 (-30.96 to 22.21)	0.7469	-3.19 (-13.36 to 6.98)	0.5388
wait-list	—	—	—	—	—	—	—	—	—	—	0.13 (-4.42 to 4.68)	0.954

7.2.5 Quality of life

**Components**	**Main estimate**		**Sensitivity 1**		**Sensitivity 2**		**Sensitivity 3**		**Sensitivity 4**		**Sensitivity 5**	
	No. of studies: 18		No. of studies: 18		No. of studies: 16		No. of studies: 12		No. of studies: 15		No. of studies: 18	
	Standardised mean difference (95% CI)	p value	Standardised mean difference (95% CI)	p value	Standardised mean difference (95% CI)	p value	Standardised mean difference (95% CI)	p value	Standardised mean difference (95% CI)	p value	Standardised mean difference (95% CI)	p value
contracting	-0.20 (-1.12 to 0.72)	0.6703	0.02 (-0.66 to 0.69)	0.9625	0.08 (-0.38 to 0.55)	0.7264	-0.04 (-0.41 to 0.34)	0.852	-0.05 (-0.57 to 0.47)	0.8469	-0.15 (-0.95 to 0.65)	0.717
cognitive restructuring	0.29 (-0.23 to 0.80)	0.271	0.44 (-0.23 to 1.10)	0.1996	0.24 (-0.20 to 0.67)	0.2857	0.17 (-0.15 to 0.50)	0.3019	0.28 (-0.17 to 0.73)	0.2278	0.16 (-0.17 to 0.48)	0.3518
device-monitoring	0.15 (-0.17 to 0.48)	0.3534	**1.02 (0.41 to 1.64)**	0.0011	0.18 (-0.20 to 0.55)	0.3531	0.05 (-0.14 to 0.24)	0.6337	0.12 (-0.17 to 0.41)	0.4251	-0.04 (-0.50 to 0.42)	0.8736
feedback	**-0.77 (-1.34 to -0.20)**	0.0078	0.78 (-0.33 to 1.89)	0.1684	**-0.67 (-1.12 to -0.23)**	0.0029	-0.10 (-0.25 to 0.04)	0.1643	-0.45 (-0.90 to 0.01)	0.0565	**-0.68 (-1.12 to -0.24)**	0.0024
functional behavioural analysis	-0.00 (-0.35 to 0.35)	0.9924	-0.30 (-1.47 to 0.86)	0.6114	-0.08 (-0.41 to 0.25)	0.6424	**0.29 (0.03 to 0.55)**	0.0274	0.20 (-0.49 to 0.89)	0.5755	0.04 (-0.31 to 0.39)	0.8414
group	-0.25 (-0.56 to 0.06)	0.1081	-0.00 (-0.31 to 0.30)	0.9959	-0.25 (-0.56 to 0.06)	0.1081	-0.25 (-0.56 to 0.06)	0.1081	-0.25 (-0.56 to 0.06)	0.1081	-0.25 (-0.56 to 0.06)	0.1081
goal-setting	0.27 (-0.58 to 1.13)	0.5293	0.76 (-0.09 to 1.61)	0.0802	0.27 (-0.58 to 1.13)	0.5293	-0.19 (-0.86 to 0.48)	0.579	0.27 (-0.58 to 1.13)	0.5293	0.27 (-0.58 to 1.13)	0.5293
inhibition training	0.33 (-0.49 to 1.16)	0.4258	0.28 (-0.46 to 1.02)	0.4592	—	—	-0.01 (-0.12 to 0.09)	0.798	0.03 (-0.44 to 0.51)	0.8865	0.28 (-0.40 to 0.96)	0.4235
modelling	-0.45 (-1.30 to 0.41)	0.3072	-0.91 (-1.98 to 0.17)	0.1	**-0.75 (-1.45 to -0.04)**	0.0377	-0.10 (-0.44 to 0.25)	0.5905	-0.36 (-0.79 to 0.06)	0.0925	-0.52 (-1.26 to 0.21)	0.1622
motivation	**0.57 (0.16 to 0.98)**	0.006	**0.57 (0.16 to 0.98)**	0.006	**0.57 (0.16 to 0.98)**	0.006	0.11 (-0.07 to 0.28)	0.2371	**0.57 (0.16 to 0.98)**	0.006	**0.57 (0.16 to 0.98)**	0.006
parental involvement	0.09 (-0.17 to 0.34)	0.504	0.09 (-0.17 to 0.34)	0.504	0.09 (-0.17 to 0.34)	0.504	0.09 (-0.17 to 0.34)	0.504	0.09 (-0.17 to 0.34)	0.504	0.09 (-0.17 to 0.34)	0.504
preplanning	-0.20 (-1.05 to 0.64)	0.6404	-0.38 (-0.90 to 0.15)	0.1585	-0.05 (-0.72 to 0.62)	0.8846	-0.02 (-0.28 to 0.24)	0.9002	-0.06 (-0.55 to 0.43)	0.8092	-0.03 (-0.51 to 0.45)	0.9112
problem solving	-0.15 (-0.71 to 0.40)	0.585	-0.15 (-0.71 to 0.40)	0.585	-0.15 (-0.71 to 0.40)	0.585	-0.15 (-0.71 to 0.40)	0.585	-0.15 (-0.71 to 0.40)	0.585	-0.15 (-0.71 to 0.40)	0.585
psychoeducation	-0.20 (-0.77 to 0.37)	0.4981	-0.45 (-1.01 to 0.12)	0.1237	-0.20 (-0.77 to 0.37)	0.4981	0.27 (-0.07 to 0.61)	0.1247	-0.20 (-0.77 to 0.37)	0.4981	-0.20 (-0.77 to 0.37)	0.4981
remote	0.28 (-0.24 to 0.80)	0.2852	**-1.51 (-2.89 to -0.12)**	0.0334	0.18 (-0.24 to 0.61)	0.3977	0.08 (-0.15 to 0.30)	0.5062	-0.04 (-0.38 to 0.29)	0.7957	0.19 (-0.24 to 0.62)	0.3841
reinforcement	0.28 (-0.43 to 1.00)	0.4346	-0.17 (-0.59 to 0.25)	0.4321	0.00 (-0.43 to 0.43)	0.9924	0.12 (-0.25 to 0.49)	0.5228	0.14 (-0.23 to 0.50)	0.4647	0.23 (-0.37 to 0.84)	0.4523
reminders	-0.40 (-1.03 to 0.24)	0.2205	-0.15 (-0.78 to 0.49)	0.6506	-0.40 (-1.03 to 0.24)	0.2205	-0.40 (-1.03 to 0.24)	0.2205	-0.40 (-1.03 to 0.24)	0.2205	-0.40 (-1.03 to 0.24)	0.2205
role playing	**-0.76 (-1.50 to -0.03)**	0.0416	-0.87 (-2.46 to 0.72)	0.2827	-0.54 (-1.12 to 0.04)	0.0701	-0.30 (-0.76 to 0.16)	0.204	-0.32 (-0.83 to 0.19)	0.2235	**-0.75 (-1.47 to -0.03)**	0.0422
relaxation training	-0.22 (-0.73 to 0.29)	0.3899	-0.04 (-0.62 to 0.54)	0.8941	-0.19 (-0.68 to 0.29)	0.4314	—	—	0.02 (-0.25 to 0.29)	0.8832	-0.17 (-0.68 to 0.35)	0.5253
rule-setting	0.56 (-0.42 to 1.53)	0.2617	**0.84 (0.18 to 1.51)**	0.0132	0.19 (-0.29 to 0.68)	0.4314	-0.01 (-0.12 to 0.09)	0.798	0.01 (-0.48 to 0.51)	0.9556	0.44 (-0.28 to 1.17)	0.231

stimulus control	0.39 (-0.89 to 1.67)	0.5497	-0.08 (-1.37 to 1.20)	0.8978	0.39 (-0.89 to 1.67)	0.5497	0.17 (-0.40 to 0.74)	0.5572	-0.17 (-0.78 to 0.44)	0.5856	0.39 (-0.89 to 1.67)	0.5497
self-concept improvement	0.23 (-0.74 to 1.19)	0.6449	0.96 (-0.27 to 2.19)	0.126	0.25 (-0.65 to 1.14)	0.5873	0.32 (-0.01 to 0.66)	0.0608	0.52 (-0.19 to 1.23)	0.1529	0.25 (-0.74 to 1.25)	0.6152
serious games	—	—	—	—	—	—	—	—	—	—	—	—
self-monitoring	-0.08 (-0.87 to 0.72)	0.8499	-0.09 (-0.88 to 0.70)	0.8262	-0.08 (-0.87 to 0.72)	0.8499	0.14 (-0.17 to 0.46)	0.3697	-0.08 (-0.87 to 0.72)	0.8499	-0.08 (-0.87 to 0.72)	0.8499
social support	0.62 (-0.14 to 1.38)	0.1083	-0.33 (-1.06 to 0.41)	0.3806	0.32 (-0.18 to 0.81)	0.2126	0.03 (-0.10 to 0.17)	0.6383	0.14 (-0.24 to 0.52)	0.4726	0.54 (-0.11 to 1.19)	0.1044
stoplight approach	-0.18 (-1.35 to 0.99)	0.7622	-0.63 (-1.37 to 0.11)	0.0961	0.36 (-0.10 to 0.82)	0.1239	0.12 (-0.25 to 0.49)	0.526	0.19 (-0.11 to 0.49)	0.2139	0.11 (-0.38 to 0.60)	0.6512
stress management	0.49 (-0.20 to 1.19)	0.1647	**1.42 (0.48 to 2.35)**	0.003	0.26 (-0.44 to 0.95)	0.471	0.08 (-0.17 to 0.34)	0.5162	0.04 (-0.48 to 0.56)	0.8722	0.60 (-0.19 to 1.39)	0.1369
task-setting	-0.13 (-0.66 to 0.39)	0.6148	0.02 (-0.38 to 0.42)	0.9236	-0.01 (-0.36 to 0.35)	0.9728	0.15 (-0.05 to 0.36)	0.1362	0.24 (-0.07 to 0.55)	0.128	-0.04 (-0.40 to 0.32)	0.8354
third-wave components	—	—	—	—	—	—	—	—	—	—	—	—
wait-list	—	—	—	—	—	—	—	—	—	—	0.32 (-0.52 to 1.17)	0.4563

7.2.6 Mental health

Components	Main estimate		Sensitivity 1		Sensitivity 2		Sensitivity 3		Sensitivity 4		Sensitivity 5	
	No. of studies: 8		No. of studies: 8		No. of studies: 4		No. of studies: 8		No. of studies:6		No. of studies: 8	
	Standardised mean difference (95% CI)	p value	Standardised mean difference (95% CI)	p value	Standardised mean difference (95% CI)	p value	Standardised mean difference (95% CI)	p value	Standardised mean difference (95% CI)	p value	Standardised mean difference (95% CI)	p value
contracting	—	—	—	—	—	—	—	—	—	—	—	—
cognitive restructuring	-0.05 (-0.29 to 0.18)	0.664	-0.05 (-0.29 to 0.18)	0.664	-0.01 (-0.16 to 0.14)	0.8937	-0.05 (-0.29 to 0.18)	0.664	-0.11 (-0.34 to 0.12)	0.3489	-0.05 (-0.29 to 0.18)	0.664
device-monitoring	-0.07 (-0.30 to 0.17)	0.5915	-0.07 (-0.30 to 0.17)	0.5915	-0.03 (-0.18 to 0.12)	0.7025	-0.07 (-0.30 to 0.17)	0.5915	-0.06 (-0.30 to 0.18)	0.6472	-0.07 (-0.30 to 0.17)	0.5915
feedback	-0.04 (-0.24 to 0.17)	0.7188	-0.04 (-0.24 to 0.17)	0.7188	-0.04 (-0.28 to 0.20)	0.7475	-0.04 (-0.24 to 0.17)	0.7188	-0.04 (-0.17 to 0.09)	0.5161	-0.04 (-0.24 to 0.17)	0.7188
functional behavioural analysis	-0.06 (-0.26 to 0.14)	0.5611	-0.06 (-0.26 to 0.14)	0.5611	—	—	-0.06 (-0.26 to 0.14)	0.5611	0.01 (-0.21 to 0.22)	0.9591	-0.06 (-0.26 to 0.14)	0.5611
group	-0.07 (-0.49 to 0.36)	0.7599	-0.07 (-0.49 to 0.36)	0.7599	-0.10 (-0.50 to 0.31)	0.6367	-0.07 (-0.49 to 0.36)	0.7599	-0.10 (-0.50 to 0.30)	0.6261	-0.07 (-0.49 to 0.36)	0.7599
goal-setting	-0.16 (-0.37 to 0.05)	0.1313	-0.16 (-0.37 to 0.05)	0.1313	-0.03 (-0.18 to 0.12)	0.7025	-0.16 (-0.37 to 0.05)	0.1313	-0.04 (-0.17 to 0.09)	0.5161	-0.16 (-0.37 to 0.05)	0.1313
inhibition training	—	—	—	—	—	—	—	—	—	—	—	—
modelling	-0.20 (-0.45 to 0.06)	0.1383	-0.20 (-0.45 to 0.06)	0.1383	—	—	-0.20 (-0.45 to 0.06)	0.1383	—	—	-0.20 (-0.45 to 0.06)	0.1383
motivation	-0.07 (-0.22 to 0.07)	0.3262	-0.07 (-0.22 to 0.07)	0.3262	-0.01 (-0.16 to 0.14)	0.8937	-0.07 (-0.22 to 0.07)	0.3262	—	—	-0.07 (-0.22 to 0.07)	0.3262
parental involvement	-0.09 (-0.46 to 0.28)	0.6251	-0.09 (-0.46 to 0.28)	0.6251	-0.05 (-0.41 to 0.32)	0.8028	-0.09 (-0.46 to 0.28)	0.6251	-0.04 (-0.42 to 0.34)	0.8205	-0.09 (-0.46 to 0.28)	0.6251
preplanning	-0.01 (-0.36 to 0.33)	0.9468	-0.01 (-0.36 to 0.33)	0.9468	0.09 (-0.13 to 0.32)	0.4266	-0.01 (-0.36 to 0.33)	0.9468	0.09 (-0.16 to 0.33)	0.48	-0.01 (-0.36 to 0.33)	0.9468
problem solving	0.34 (-0.22 to 0.90)	0.2293	0.34 (-0.22 to 0.90)	0.2293	0.34 (-0.22 to 0.90)	0.2293	0.34 (-0.22 to 0.90)	0.2293	0.34 (-0.22 to 0.90)	0.2293	0.34 (-0.22 to 0.90)	0.2293
psychoeducation	0.09 (-0.26 to 0.44)	0.6059	0.09 (-0.26 to 0.44)	0.6059	0.05 (-0.32 to 0.41)	0.8064	0.09 (-0.26 to 0.44)	0.6059	0.04 (-0.31 to 0.40)	0.8083	0.09 (-0.26 to 0.44)	0.6059
remote	-0.06 (-0.37 to 0.25)	0.6947	-0.06 (-0.37 to 0.25)	0.6947	-0.09 (-0.38 to 0.20)	0.5333	-0.06 (-0.37 to 0.25)	0.6947	-0.09 (-0.39 to 0.21)	0.5407	-0.06 (-0.37 to 0.25)	0.6947
reinforcement	-0.25 (-0.61 to 0.12)	0.1842	-0.25 (-0.61 to 0.12)	0.1842	-0.18 (-0.58 to 0.21)	0.3546	-0.25 (-0.61 to 0.12)	0.1842	-0.18 (-0.60 to 0.24)	0.3975	-0.25 (-0.61 to 0.12)	0.1842
reminders	0.03 (-0.20 to 0.26)	0.8176	0.03 (-0.20 to 0.26)	0.8176	-0.00 (-0.22 to 0.21)	0.9665	0.03 (-0.20 to 0.26)	0.8176	-0.01 (-0.21 to 0.20)	0.9462	0.03 (-0.20 to 0.26)	0.8176
role playing	0.10 (-0.14 to 0.34)	0.419	0.10 (-0.14 to 0.34)	0.419	—	—	0.10 (-0.14 to 0.34)	0.419	0.01 (-0.15 to 0.18)	0.8723	0.10 (-0.14 to 0.34)	0.419
relaxation training	-0.18 (-0.46 to 0.11)	0.2316	-0.18 (-0.46 to 0.11)	0.2316	—	—	-0.18 (-0.46 to 0.11)	0.2316	-0.11 (-0.34 to 0.12)	0.3489	-0.18 (-0.46 to 0.11)	0.2316
rule-setting	—	—	—	—	—	—	—	—	—	—	—	—
stimulus control	-0.07 (-0.22 to 0.07)	0.326	-0.07 (-0.22 to 0.07)	0.3262	-0.01 (-0.16 to 0.14)	0.8937	-0.07 (-0.22 to 0.07)	0.3262	—	—	-0.07 (-0.22 to 0.07)	0.326

		2									2
self-concept improvement	0.11 (-0.14 to 0.36)	0.392	0.11 (-0.14 to 0.36)	0.392	-0.03 (-0.18 to 0.12)	0.7025	0.11 (-0.14 to 0.36)	0.392	0.05 (-0.18 to 0.29)	0.6543	0.11 (-0.14 to 0.36)	0.392
serious games	—	—	—	—	—	—	—	—	—	—	—	—
self-monitoring	—	—	—	—	—	—	—	—	—	—	—	—
social support	0.03 (-0.11 to 0.18)	0.6472	0.03 (-0.11 to 0.18)	0.6472	-0.03 (-0.18 to 0.12)	0.7025	0.03 (-0.11 to 0.18)	0.6472	-0.04 (-0.17 to 0.09)	0.5161	0.03 (-0.11 to 0.18)	0.6472
stoplight approach	-0.07 (-0.30 to 0.17)	0.5915	-0.07 (-0.30 to 0.17)	0.5915	-0.03 (-0.18 to 0.12)	0.7025	-0.07 (-0.30 to 0.17)	0.5915	-0.06 (-0.30 to 0.18)	0.6472	-0.07 (-0.30 to 0.17)	0.5915
stress management	0.09 (-0.13 to 0.31)	0.4359	0.09 (-0.13 to 0.31)	0.4359	0.09 (-0.13 to 0.32)	0.4266	0.09 (-0.13 to 0.31)	0.4359	0.10 (-0.11 to 0.31)	0.3472	0.09 (-0.13 to 0.31)	0.4359
task-setting	0.12 (-0.14 to 0.39)	0.363	0.12 (-0.14 to 0.39)	0.363	-0.01 (-0.16 to 0.14)	0.8937	0.12 (-0.14 to 0.39)	0.363	—	—	0.12 (-0.14 to 0.39)	0.363
third-wave components	-0.04 (-0.33 to 0.25)	0.7869	-0.04 (-0.33 to 0.25)	0.7869	—	—	-0.04 (-0.33 to 0.25)	0.7869	-0.10 (-0.38 to 0.17)	0.4481	-0.04 (-0.33 to 0.25)	0.7869
wait-list	—	—	—	—	—	—	—	—	—	—	—	—

Appendix 8 Results of BMI

8.1 Network plots

8.1.1 Network plot in conceptual level NMA

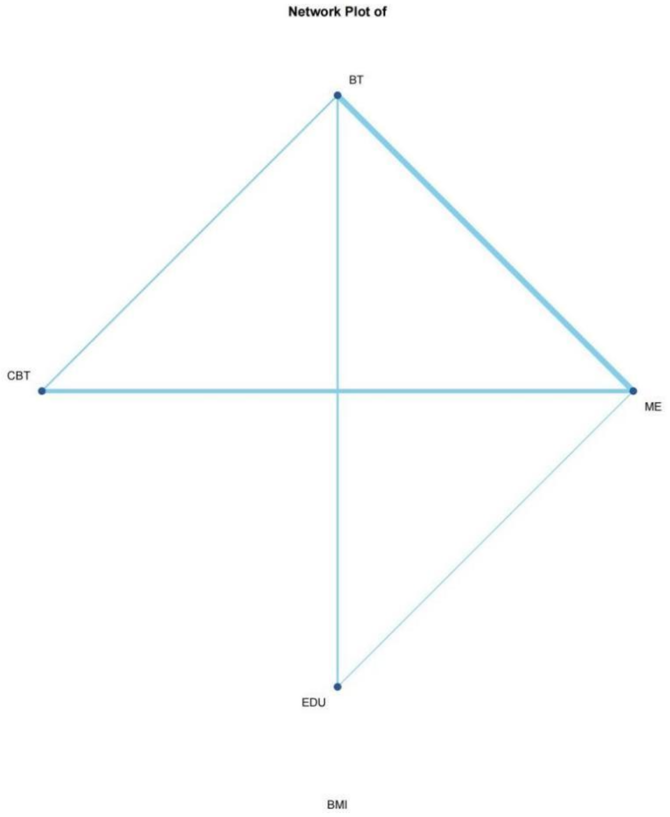

Notes: BT, behavioural therapy; CBT, cognitive behavioural therapy; CT, cognitive therapy; EDU, psychoeducation; ME, minimal education

8.1.2 Network plot in technical level NMA

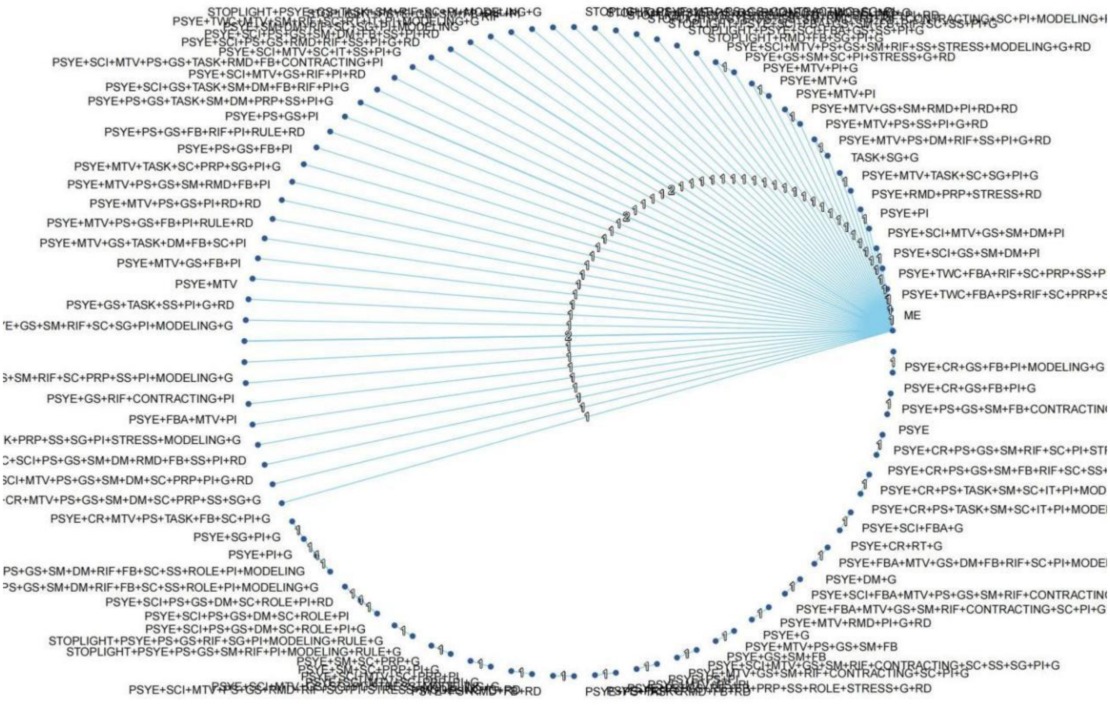

Notes: ME, minimal education; PSYE, psychoeducation; CR, cognitive restructuring; TWC, third-wave components; SCI, self-concept improvement; FBA, functional behavioural analysis; MTV, motivation; GS, goal-setting; TASK, task-setting; RULE, rule-setting; SM, self-monitoring; DM, device-monitoring; RMD, reminders; FB, feedback; PS, problem solving; PRP, preplanning; RIF, reinforcement; CONTRACTING, contracting; MODELING, modelling; SC, stimulus control; RT, relaxation training; IT, inhibition training; SS, social support; STRESS, stress management; STOPLIGHT, stoplight approach; ROLE, role playing; G, group; RD, remote; SG, serious games; PI, parental involvement

8.2 Forest plots

8.2.1 Forest plot in conceptual level NMA

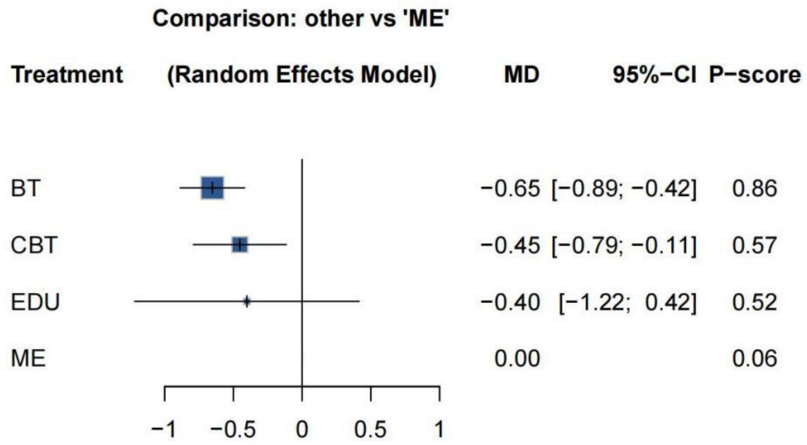

Notes: BT, behavioural therapy; CBT, cognitive behavioural therapy; CT, cognitive therapy; EDU, psychoeducation; ME, minimal education

8.2.2 Forest plot in technical level NMA

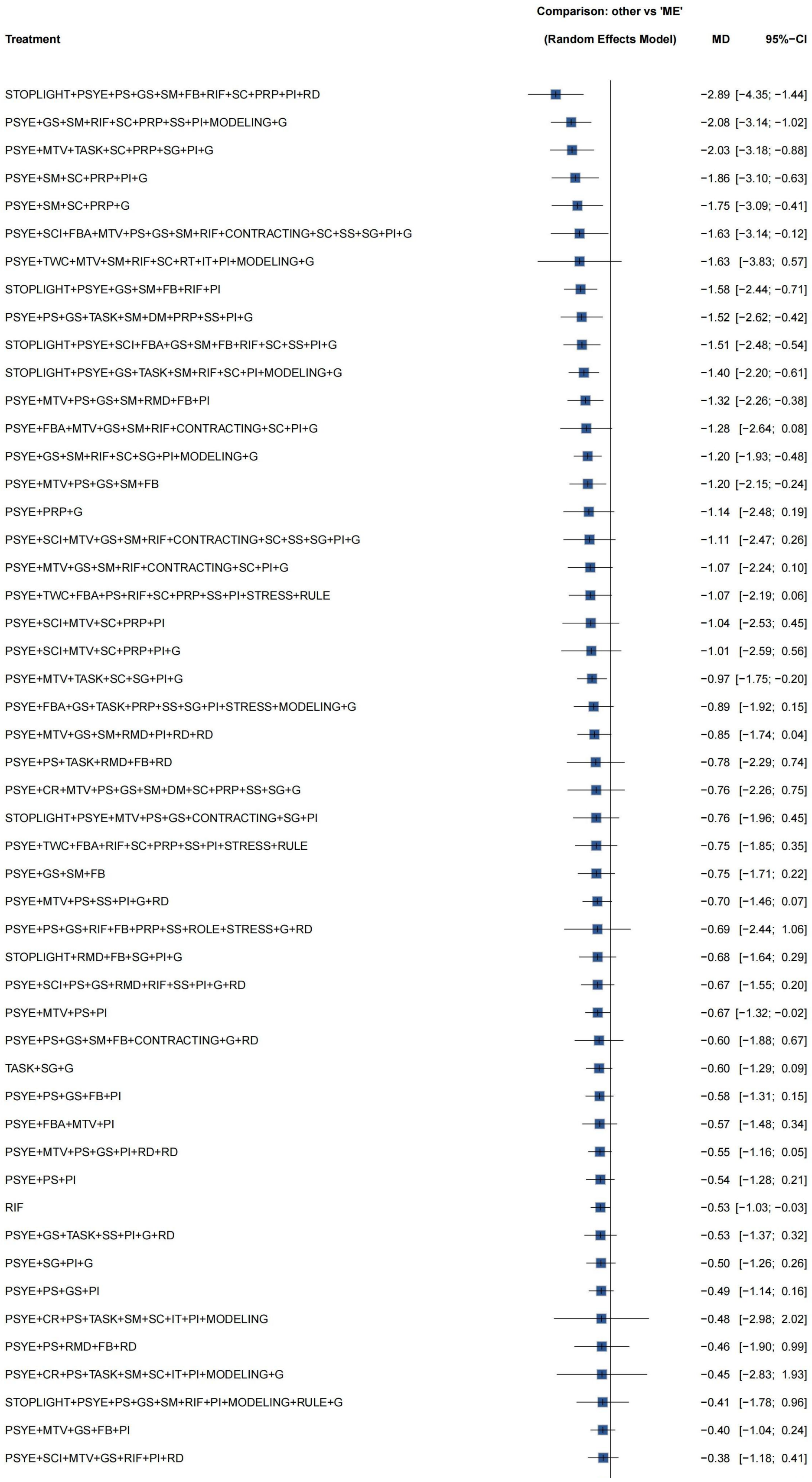

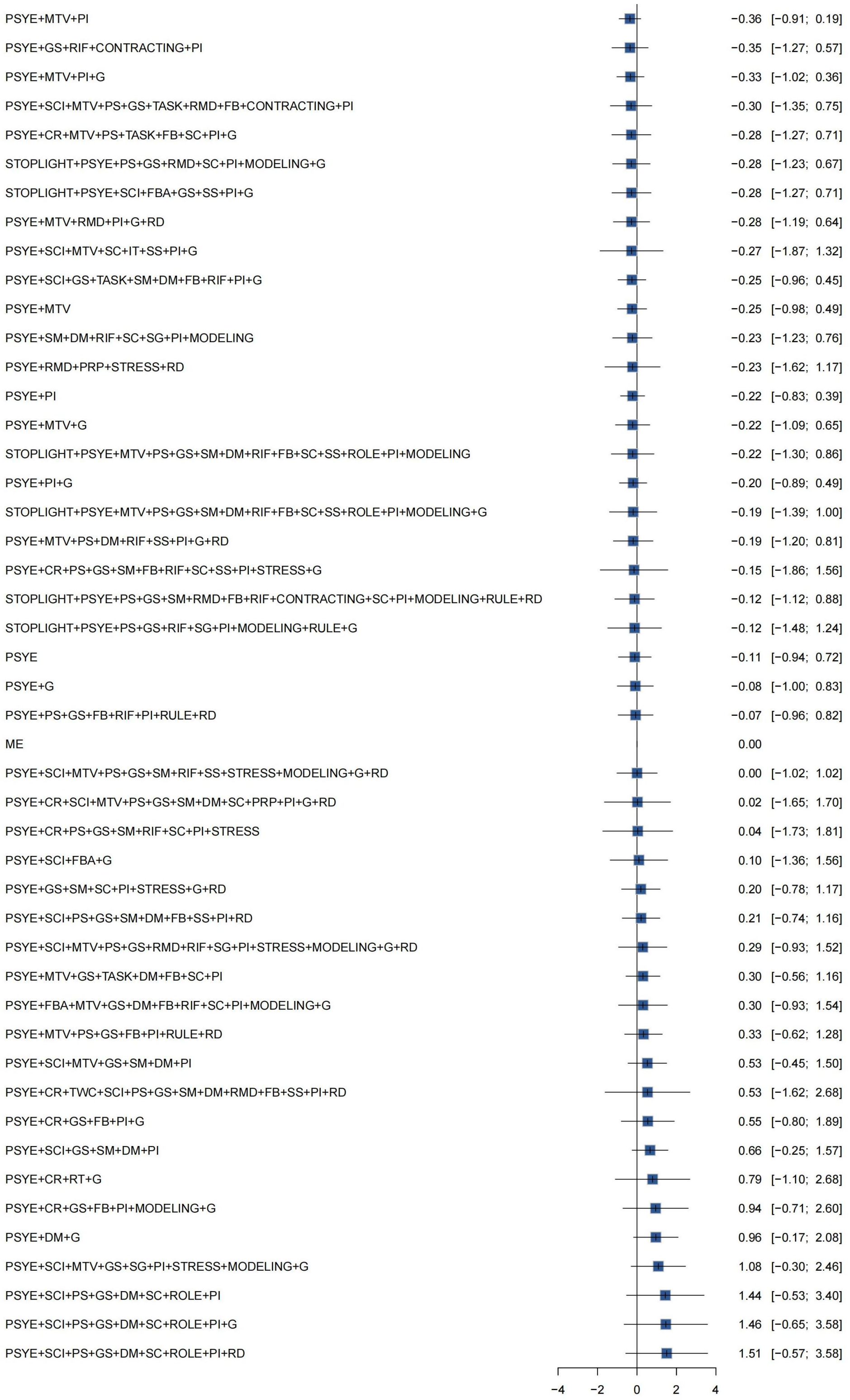

Notes: ME, minimal education; PSYE, psychoeducation; CR, cognitive restructuring; TWC, third-wave components; SCI, self-concept improvement; FBA, functional behavioural analysis; MTV, motivation; GS, goal-setting; TASK, task-setting; RULE, rule-setting; SM, self-monitoring; DM, device-monitoring; RMD, reminders; FB, feedback; PS, problem solving; PRP, preplanning; RIF, reinforcement; CONTRACTING, contracting; MODELING, modelling; SC, stimulus control; RT, relaxation training; IT, inhibition training; SS, social support; STRESS, stress management; STOPLIGHT, stoplight approach; ROLE, role playing; G, group; RD, remote; SG, serious games; PI, parental involvement

8.2.3 Pairwise forest plot in conceptual level NMA

Study	Experimental Total	Experimental Mean	Experimental SD	Control Total	Control Mean	Control SD	MD [95%-CI]	Weight (common)	Weight (random)
**BT:CBT**									
Jelalian_2006	39	−1.48	1.8700	37	0.18	1.8200	−1.66 [−2.49; −0.83]	100.0%	100.0%
**BT:EDU**									
Abraham_2015	16	0.03	3.6559	16	−0.10	3.2327	0.13 [−2.26; 2.52]	12.2%	12.2%
Williamson-2005	28	−0.19	2.4000	29	0.65	0.2300	−0.84 [−1.73; 0.05]	87.8%	87.8%
**Common effect model**	**44**			**45**			**−0.72 [−1.56; 0.12]**	**100.0%**	.
**Random effects model**							**−0.72 [−1.56; 0.12]**	.	**100.0%**
Heterogeneity: I^2 = 0%, τ^2 = 0, p = 0.4564									
**BT:ME**									
Faith, M. S._2001	6	−0.30	3.6213	4	1.00	2.1863	−1.30 [−4.90; 2.30]	0.1%	0.6%
Nemet_2005	24	−1.70	3.7565	22	−0.20	5.0000	−1.50 [−4.07; 1.07]	0.1%	1.0%
Walpole-2013	20	−0.20	2.5933	20	−0.20	4.7875	0.00 [−2.39; 2.39]	0.2%	1.1%
Abraham_2015	16	0.03	3.6559	16	0.13	2.7778	−0.10 [−2.35; 2.15]	0.2%	1.2%
Saelens_2002	23	0.10	3.8549	21	1.40	3.2949	−1.30 [−3.41; 0.81]	0.2%	1.3%
Cohen, T. R. _2023	30	0.40	3.1944	30	0.30	4.3152	0.10 [−1.82; 2.02]	0.3%	1.5%
Hofsteenge, G. H._2014	71	−0.50	4.3630	51	0.60	4.8812	−1.10 [−2.78; 0.58]	0.3%	1.8%
Pakpour_2015	238	−1.34	6.8870	119	0.03	7.8210	−1.37 [−3.03; 0.29]	0.4%	1.8%
Wake−-2013	62	0.90	3.1998	56	0.80	3.9462	0.10 [−1.20; 1.40]	0.6%	2.4%
Smith-2021	141	1.15	5.0825	99	0.52	4.8668	0.63 [−0.64; 1.90]	0.6%	2.4%
Johnston_2010	40	−0.99	3.7900	20	1.08	1.0000	−2.07 [−3.32; −0.82]	0.6%	2.4%
Maddison_2014	127	0.02	4.3138	124	0.13	4.9212	−0.11 [−1.26; 1.04]	0.7%	2.6%
Savoye_2011	105	−1.80	3.4000	69	1.90	3.6000	−3.70 [−4.77; −2.63]	0.9%	2.8%
Tsai-2024	82	0.98	2.7848	82	2.14	3.4567	−1.16 [−2.12; −0.20]	1.1%	3.0%
Christie_2017	87	0.18	2.8000	87	0.25	2.8000	−0.07 [−0.90; 0.76]	1.4%	3.3%
Taveras_2015	365	0.75	4.2100	184	1.20	4.1427	−0.45 [−1.19; 0.29]	1.8%	3.5%
Jiang_2005	33	−2.60	1.6000	35	−0.10	1.1000	−2.50 [−3.16; −1.84]	2.3%	3.6%
McCallum_2007	82	0.50	2.2789	81	0.80	1.9091	−0.30 [−0.95; 0.35]	2.4%	3.7%
Taylor-2015	104	0.10	2.5000	102	0.40	1.9779	−0.30 [−0.91; 0.31]	2.6%	3.7%
Kalavainen_2007	35	0.10	1.2000	35	0.80	1.3000	−0.70 [−1.29; −0.11]	2.9%	3.8%
Wright_2013	24	−0.01	1.1000	26	0.20	1.0000	−0.21 [−0.79; 0.37]	2.9%	3.8%
Ruotsalainen_2015	31	−0.36	0.9340	15	0.00	0.9000	−0.36 [−0.92; 0.20]	3.1%	3.8%
Crespo, N. C._2018	149	0.90	2.5374	148	1.00	2.3457	−0.10 [−0.66; 0.46]	3.2%	3.8%
Sauder_2018	29	0.40	1.0800	33	0.90	1.1500	−0.50 [−1.06; 0.06]	3.2%	3.8%
Wake_2009	139	0.30	2.3135	119	0.30	1.9412	0.00 [−0.52; 0.52]	3.6%	3.9%
Johnston_2013	46	−0.16	1.0500	25	0.64	0.9000	−0.80 [−1.27; −0.33]	4.5%	4.0%
Waling_2012	48	0.08	0.9500	45	0.20	1.1200	−0.12 [−0.54; 0.30]	5.5%	4.1%
Arlinghaus_2019	180	−0.03	1.5700	63	0.90	1.3500	−0.93 [−1.33; −0.53]	6.0%	4.1%
Tucker_2013	68	0.00	1.0000	57	0.30	1.2000	−0.30 [−0.69; 0.09]	6.4%	4.1%
Luque_2024	106	−0.02	1.4300	95	0.20	1.2800	−0.22 [−0.59; 0.15]	7.0%	4.2%
Moore_2019	123	1.08	1.1290	119	0.95	1.3180	0.13 [−0.18; 0.44]	10.2%	4.2%
Patsopoulou_2017	120	−1.25	1.0000	61	0.30	1.0000	−1.55 [−1.86; −1.24]	10.3%	4.2%
Broccoli_2016	187	0.46	1.3300	185	0.78	1.2100	−0.32 [−0.58; −0.06]	14.7%	4.3%
**Common effect model**	**2941**			**2248**			**−0.53 [−0.63; −0.43]**	**100.0%**	.
**Random effects model**							**−0.63 [−0.92; −0.34]**	.	**100.0%**
Heterogeneity: I^2 = 81.1%, τ^2 = 0.4980, p < 0.0001									
**CBT:BT**									
Jelalian_2011	45	−1.50	3.4399	44	−1.31	3.0657	−0.19 [−1.54; 1.16]	5.2%	5.2%
Moore_2019	118	0.82	1.3630	123	1.08	1.1290	−0.26 [−0.58; 0.05]	94.8%	94.8%
**Common effect model**	**163**			**167**			**−0.26 [−0.57; 0.05]**	**100.0%**	.
**Random effects model**							**−0.26 [−0.57; 0.05]**	.	**100.0%**
Heterogeneity: I^2 = 0%, τ^2 = 0, p = 0.9191									
**CBT:ME**									
Ball_2011	32	−0.91	7.0000	14	0.80	6.1497	−1.71 [−5.75; 2.32]	0.1%	0.7%
Tsiros-2008	25	−1.60	4.2214	22	0.30	7.4852	−1.90 [−5.44; 1.64]	0.1%	0.9%
Doyle_2008	40	−0.65	7.2205	40	0.21	6.3095	−0.86 [−3.83; 2.11]	0.2%	1.2%
Boutelle_2014	22	−1.00	4.3726	22	0.30	4.3701	−1.30 [−3.88; 1.28]	0.2%	1.5%
Norman_2016_2	24	−0.20	3.7523	28	0.20	3.7992	−0.40 [−2.46; 1.66]	0.4%	2.2%
Norman_2016_1	29	0.30	3.7336	25	0.10	3.7167	0.20 [−1.79; 2.19]	0.4%	2.4%
Pbert_2016	54	−0.14	5.2119	57	−0.04	4.8874	−0.10 [−1.98; 1.78]	0.4%	2.6%
Alustiza_2021	47	0.50	3.4251	45	0.90	3.1407	−0.40 [−1.74; 0.94]	0.9%	4.2%
Diaz_2010	21	−1.80	1.9000	22	0.40	2.1000	−2.20 [−3.40; −1.00]	1.1%	4.8%
Ham, O. K._2016	48	0.02	2.5610	27	0.77	2.2633	−0.75 [−1.87; 0.37]	1.3%	5.2%
Bagherniya_2018	87	−0.70	3.8623	85	0.40	2.7204	−1.10 [−2.10; −0.10]	1.6%	5.9%
Kalarchian_2009	97	−0.68	2.8600	95	0.54	2.0500	−1.22 [−1.92; −0.52]	3.2%	7.9%
Anna Ek_2019	87	−0.04	2.4400	87	0.78	2.0900	−0.82 [−1.50; −0.14]	3.5%	8.1%
Arlinghaus_2021_2	27	0.08	0.6200	32	−0.51	1.3600	0.59 [0.06; 1.12]	5.7%	9.2%
Arlinghaus_2021_1	58	−0.20	1.2900	54	0.22	0.7300	−0.42 [−0.80; −0.04]	10.7%	10.3%
Moore_2019	118	0.82	1.3630	119	0.95	1.3180	−0.13 [−0.47; 0.21]	13.6%	10.6%
Taveras_2011	253	0.31	1.4300	192	0.49	1.3900	−0.18 [−0.44; 0.08]	22.7%	11.0%
Robinson_2021	120	1.05	0.8800	121	1.11	0.8300	−0.06 [−0.28; 0.16]	33.9%	11.3%
**Common effect model**	**1189**			**1087**			**−0.22 [−0.35; −0.10]**	**100.0%**	.
**Random effects model**							**−0.48 [−0.83; −0.14]**	.	**100.0%**
Heterogeneity: I^2 = 57.8%, τ^2 = 0.2572, p = 0.0012									
**EDU:BT**									
Resnicow_2005	70	0.40	7.0963	53	−0.10	5.3068	0.50 [−1.69; 2.69]	5.1%	5.1%
Janicke, D. M._2019	83	1.11	1.8800	166	1.33	2.0200	−0.22 [−0.73; 0.29]	94.9%	94.9%
**Common effect model**	**153**			**219**			**−0.18 [−0.68; 0.31]**	**100.0%**	.
**Random effects model**							**−0.18 [−0.68; 0.31]**	.	**100.0%**
Heterogeneity: I^2 = 0%, τ^2 = 0, p = 0.5305									
**EDU:ME**									
Abraham_2015	16	−0.10	3.2327	16	0.13	2.7778	−0.23 [−2.32; 1.86]	100.0%	100.0%

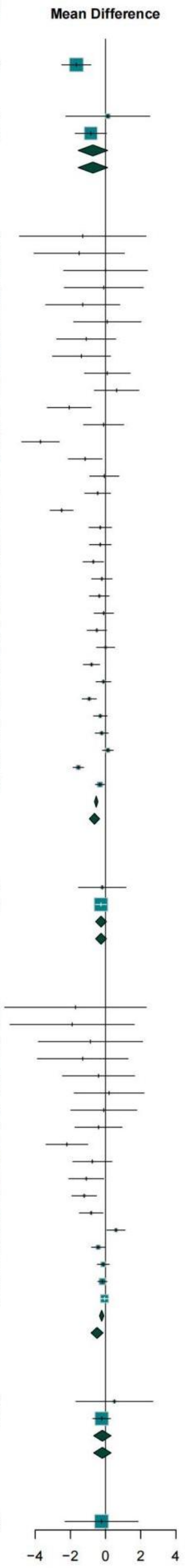

Notes: BT, behavioural therapy; CBT, cognitive behavioural therapy; CT, cognitive therapy; EDU, psychoeducation; ME, minimal education

8.2.4 Pairwise forest plot in technical level NMA

Study	Experimental Total	Experimental Mean	Experimental SD	Control Total	Control Mean	Control SD	MD [95%−CI]	Weight (common)	Weight (random)
**PSYE+CR+GS+FB+PI+G:PSYE+CR+GS+FB+PI+MODELING+G**									
Hadley_2015	19	−1.40	3.0600	19	−0.90	3.7149	−0.50 [−2.66; 1.66]	100.0%	100.0%
**PSYE+CR+MTV+PS+GS+SM+DM+SC+PRP+SS+SG+G:ME**									
Pbert_2016	54	−0.14	5.2119	57	−0.04	4.8874	−0.10 [−1.98; 1.78]	100.0%	100.0%
**PSYE+CR+MTV+PS+TASK+FB+SC+PI+G:ME**									
Robinson_2021	120	1.05	0.8800	121	1.11	0.8300	−0.06 [−0.28; 0.16]	100.0%	100.0%
**PSYE+CR+PS+GS+SM+FB+RIF+SC+SS+PI+STRESS+G:PSYE+CR+PS+GS+SM+RIF+SC+PI+STRESS**									
Berkowitz, R. I. _2013	81	−0.45	3.1500	88	−0.38	3.3800	−0.07 [−1.05; 0.91]	100.0%	100.0%
**PSYE+CR+PS+TASK+SM+SC+IT+PI+MODELING:PSYE+CR+PS+TASK+SM+SC+IT+PI+MODELING+G**									
Golan, M._2006	17	−1.09	1.2400	20	−0.14	1.3400	−0.95 [−1.78; −0.12]	100.0%	100.0%
**PSYE+CR+RT+G:PSYE+SCI+FBA+G**									
Weigensberg−2014	18	0.77	1.1800	15	0.16	1.3700	0.61 [−0.27; 1.49]	100.0%	100.0%
**PSYE+CR+SCI+MTV+PS+GS+SM+DM+SC+PRP+PI+G+RD:ME**									
Tsiros−2008	25	−1.60	4.2214	22	0.30	7.4852	−1.90 [−5.44; 1.64]	100.0%	100.0%
**PSYE+CR+TWC+SCI+PS+GS+SM+DM+RMD+FB+SS+PI+RD:ME**									
Doyle_2008	40	−0.65	7.2205	40	0.21	6.3095	−0.86 [−3.83; 2.11]	100.0%	100.0%
**PSYE+FBA+GS+TASK+PRP+SS+SG+PI+STRESS+MODELING+G:ME**									
Kalavainen_2007	35	0.10	1.2000	35	0.80	1.3000	−0.70 [−1.29; −0.11]	100.0%	100.0%
**PSYE+FBA+MTV+GS+DM+FB+RIF+SC+PI+MODELING+G:PSYE+DM+G**									
Bocca, G._2014	40	−1.20	1.0000	35	−0.60	1.1000	−0.60 [−1.08; −0.12]	100.0%	100.0%
**PSYE+FBA+MTV+PI:ME**									
Christie_2017	87	0.18	2.8000	87	0.25	2.8000	−0.07 [−0.90; 0.76]	100.0%	100.0%
**PSYE+G:PSYE+MTV+RMD+PI+G+RD**									
Resnicow_2005	70	0.40	7.0963	53	−0.10	5.3068	0.50 [−1.69; 2.69]	100.0%	100.0%
**PSYE+GS+RIF+CONTRACTING+PI:ME**									
Wake_2009	139	0.30	2.3135	119	0.30	1.9412	0.00 [−0.52; 0.52]	100.0%	100.0%
**PSYE+GS+SM+FB:PSYE+MTV+PS+GS+SM+FB**									
Gourlan, M._2013	34	0.12	5.5723	28	−1.61	4.3570	1.73 [−0.74; 4.20]	100.0%	100.0%
**PSYE+GS+SM+RIF+SC+PRP+SS+PI+MODELING+G:ME**									
Savoye_2011	105	−1.80	3.4000	69	1.90	3.6000	−3.70 [−4.77; −2.63]	100.0%	100.0%
**PSYE+GS+SM+RIF+SC+SG+PI+MODELING+G:ME**									
Johnston_2010	40	−0.99	3.7900	20	1.08	1.0000	−2.07 [−3.32; −0.82]	12.1%	39.1%
Johnston_2013	46	−0.16	1.0500	25	0.64	0.9000	−0.80 [−1.27; −0.33]	87.9%	60.9%
**Common effect model**	**86**			**45**			**−0.95 [−1.39; −0.52]**	**100.0%**	**.**
**Random effects model**							**−1.30 [−2.51; −0.08]**	**.**	**100.0%**
Heterogeneity: $I^2 = 71.1\%$, $\tau^2 = 0.5737$, $p = 0.0627$									
**PSYE+GS+SM+SC+PI+STRESS+G+RD:ME**									
Moore_2019	123	1.08	1.1290	119	0.95	1.3180	0.13 [−0.18; 0.44]	100.0%	100.0%
**PSYE+GS+TASK+SS+PI+G+RD:ME**									
Waling_2012	48	0.08	0.9500	45	0.20	1.1200	−0.12 [−0.54; 0.30]	100.0%	100.0%
**PSYE+MTV:ME**									
Walpole−2013	20	−0.20	2.5933	20	−0.20	4.7875	0.00 [−2.39; 2.39]	100.0%	100.0%
**PSYE+MTV+G:ME**									
Pakpour_2015	119	−0.63	7.7568	119	0.03	7.8210	−0.66 [−2.64; 1.32]	100.0%	100.0%
**PSYE+MTV+G:PSYE+MTV+PI+G**									
Pakpour_2015	119	−0.63	7.7568	119	−2.05	5.8028	1.42 [−0.32; 3.16]	100.0%	100.0%
**PSYE+MTV+GS+FB+PI:ME**									
Broccoli_2016	187	0.46	1.3300	185	0.78	1.2100	−0.32 [−0.58; −0.06]	100.0%	100.0%
**PSYE+MTV+GS+SM+RIF+CONTRACTING+SC+PI+G:PSYE+SCI+MTV+GS+SM+RIF+CONTRACTING+SC+SS+SG+PI+G**									
Jelalian_2006	39	−1.48	1.8700	37	0.18	1.8200	−1.66 [−2.49; −0.83]	100.0%	100.0%
**PSYE+MTV+GS+SM+RMD+PI+RD+RD:ME**									
Taveras_2015	171	0.80	4.1427	184	1.20	4.1427	−0.40 [−1.26; 0.46]	100.0%	100.0%
**PSYE+MTV+GS+TASK+DM+FB+SC+PI:ME**									
Luque_2024	106	−0.02	1.4300	95	0.20	1.2800	−0.22 [−0.59; 0.15]	100.0%	100.0%
**PSYE+MTV+PI:ME**									
Taveras_2015	194	0.70	4.2692	184	1.20	4.1427	−0.50 [−1.35; 0.35]	100.0%	100.0%
**PSYE+MTV+PI:PSYE+MTV+GS+SM+RMD+PI+RD+RD**									
Taveras_2015	194	0.70	4.2692	171	0.80	4.1427	−0.10 [−0.96; 0.76]	100.0%	100.0%
**PSYE+MTV+PI+G:ME**									
Pakpour_2015	119	−2.05	5.8028	119	0.03	7.8210	−2.08 [−3.83; −0.33]	100.0%	100.0%

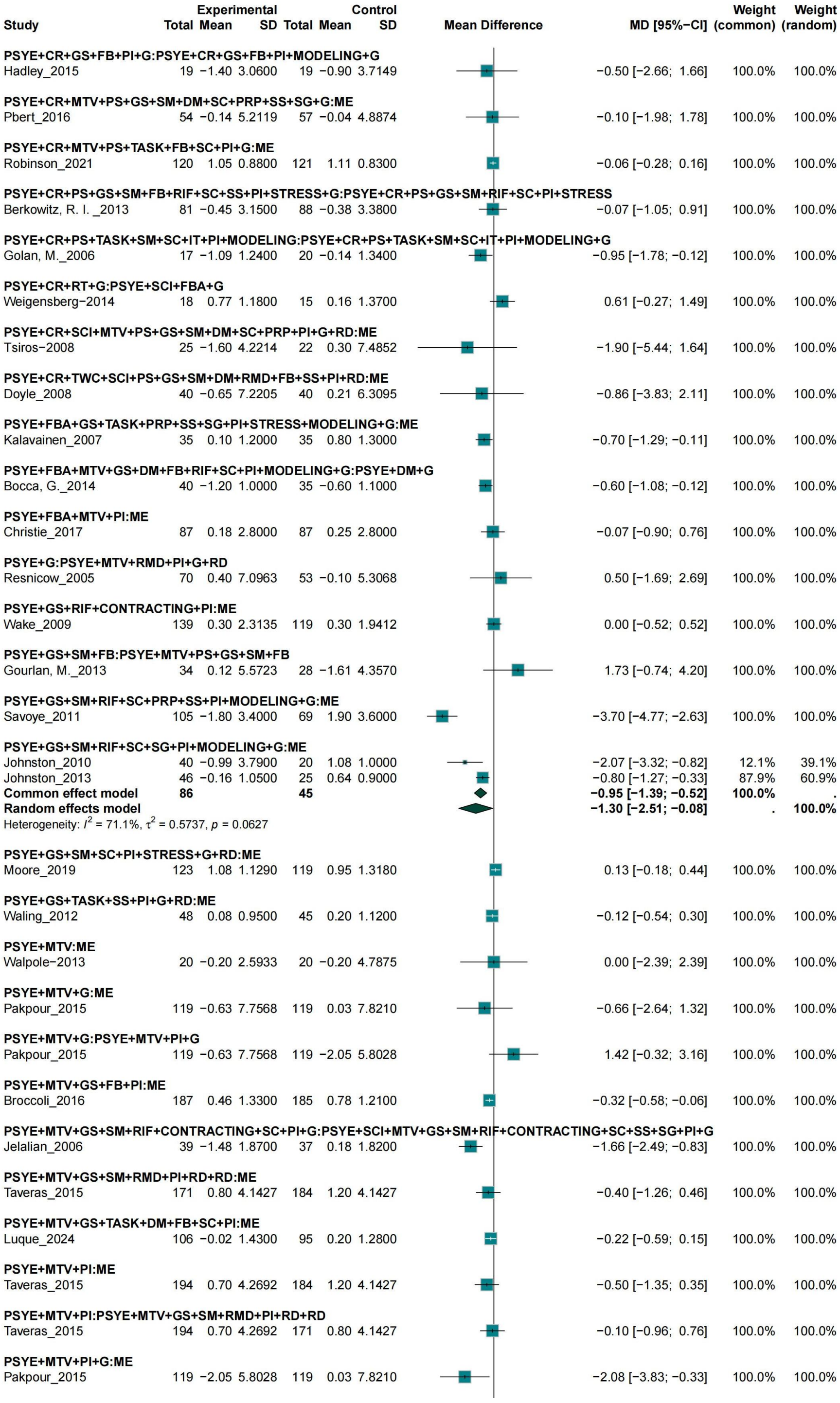

**PSYE+MTV+PS+DM+RIF+SS+PI+G+RD:ME**									
Ruotsalainen_2015	15	−0.10	0.9000	15	0.00	0.9000	−0.10 [−0.74; 0.54]	100.0%	100.0%
**PSYE+MTV+PS+DM+RIF+SS+PI+G+RD:PSYE+MTV+PS+SS+PI+G+RD**									
Ruotsalainen_2015	15	−0.10	0.9000	16	−0.60	0.9000	0.50 [−0.13; 1.13]	100.0%	100.0%
**PSYE+MTV+PS+GS+FB+PI+RULE+RD:ME**									
Smith−2021	141	1.15	5.0825	99	0.52	4.8668	0.63 [−0.64; 1.90]	100.0%	100.0%
**PSYE+MTV+PS+GS+PI+RD+RD:ME**									
Tucker_2013	68	0.00	1.0000	57	0.30	1.2000	−0.30 [−0.69; 0.09]	100.0%	100.0%
**PSYE+MTV+PS+GS+SM+RMD+FB+PI:ME**									
Tsai−2024	82	0.98	2.7848	82	2.14	3.4567	−1.16 [−2.12; −0.20]	100.0%	100.0%
**PSYE+MTV+PS+PI:PSYE+PS+PI**									
MacDonell_2012	22	0.50	6.4356	22	0.86	8.6864	−0.36 [−4.88; 4.16]	100.0%	100.0%
**PSYE+MTV+PS+SS+PI+G+RD:ME**									
Ruotsalainen_2015	16	−0.60	0.9000	15	0.00	0.9000	−0.60 [−1.23; 0.03]	100.0%	100.0%
**PSYE+MTV+TASK+SC+PRP+SG+PI+G:ME**									
Nemet_2005	24	−1.70	3.7565	22	−0.20	5.0000	−1.50 [−4.07; 1.07]	100.0%	100.0%
**PSYE+MTV+TASK+SC+SG+PI+G:ME**									
Patsopoulou_2017	60	−1.40	0.9900	61	0.30	1.0000	−1.70 [−2.05; −1.35]	100.0%	100.0%
**PSYE+PI:ME**									
Abraham_2015	16	−0.10	3.2327	16	0.13	2.7778	−0.23 [−2.32; 1.86]	100.0%	100.0%
**PSYE+PI+G:PSYE+SG+PI+G**									
Sanchez−Lopez A M_2020	49	1.37	3.3129	49	−1.65	3.8838	3.02 [1.59; 4.45]	100.0%	100.0%
**PSYE+PI+G:STOPLIGHT+PSYE+MTV+PS+GS+SM+DM+RIF+FB+SC+SS+ROLE+PI+MODELING**									
Janicke, D. M._2019	83	1.11	1.8800	78	1.11	1.9800	0.00 [−0.60; 0.60]	100.0%	100.0%
**PSYE+PI+G:STOPLIGHT+PSYE+MTV+PS+GS+SM+DM+RIF+FB+SC+SS+ROLE+PI+MODELING+G**									
Janicke, D. M._2019	83	1.11	1.8800	88	1.41	2.0600	−0.30 [−0.89; 0.29]	100.0%	100.0%
**PSYE+PS+GS+FB+PI:ME**									
Wake−−2013	62	0.90	3.1998	56	0.80	3.9462	0.10 [−1.20; 1.40]	100.0%	100.0%
**PSYE+PS+GS+FB+RIF+PI+RULE+RD:ME**									
Taylor−2015	104	0.10	2.5000	102	0.40	1.9779	−0.30 [−0.91; 0.31]	100.0%	100.0%
**PSYE+PS+GS+PI:ME**									
McCallum_2007	82	0.50	2.2789	81	0.80	1.9091	−0.30 [−0.95; 0.35]	100.0%	100.0%
**PSYE+PS+GS+RIF+FB+PRP+SS+ROLE+STRESS+G+RD:PSYE+PRP+G**									
Grey, M._2009	112	0.10	2.9700	86	−0.10	2.6000	0.20 [−0.58; 0.98]	100.0%	100.0%
**PSYE+PS+GS+SM+FB+CONTRACTING+G+RD:PSYE**									
Williamson−2005	28	−0.19	2.4000	29	0.65	0.2300	−0.84 [−1.73; 0.05]	100.0%	100.0%
**PSYE+PS+GS+TASK+SM+DM+PRP+SS+PI+G:ME**									
Hofsteenge, G. H._2014	71	−0.50	4.3630	51	0.60	4.8812	−1.10 [−2.78; 0.58]	100.0%	100.0%
**PSYE+PS+RMD+FB+RD:PSYE+PS+TASK+RMD+FB+RD**									
Kulendran_2016	13	1.06	2.3400	14	−0.12	1.9500	1.18 [−0.45; 2.81]	100.0%	100.0%
**PSYE+RMD+PRP+STRESS+RD:ME**									
Abraham_2015	16	0.03	3.6559	16	0.13	2.7778	−0.10 [−2.35; 2.15]	100.0%	100.0%
**PSYE+RMD+PRP+STRESS+RD:PSYE+PI**									
Abraham_2015	16	0.03	3.6559	16	−0.10	3.2327	0.13 [−2.26; 2.52]	100.0%	100.0%
**PSYE+SCI+FBA+MTV+PS+GS+SM+RIF+CONTRACTING+SC+SS+SG+PI+G:PSYE+FBA+MTV+GS+SM+RIF+CONTRACTING+SC+PI+G**									
Jelalian_2011	45	−1.50	3.4399	44	−1.31	3.0657	−0.19 [−1.54; 1.16]	100.0%	100.0%
**PSYE+SCI+GS+SM+DM+PI:ME**									
Ball_2011	15	−0.70	7.5562	14	0.80	6.1497	−1.50 [−6.50; 3.50]	100.0%	100.0%
**PSYE+SCI+GS+SM+DM+PI:PSYE+SCI+MTV+GS+SM+DM+PI**									
Ball_2011	15	−0.70	7.5562	17	−1.10	6.4696	0.40 [−4.51; 5.31]	100.0%	100.0%
**PSYE+SCI+GS+TASK+SM+DM+FB+RIF+PI+G:ME**									
Arlinghaus_2021_2	27	0.08	0.6200	32	−0.51	1.3600	0.59 [0.06; 1.12]	34.9%	48.4%
Arlinghaus_2021_1	58	−0.20	1.2900	54	0.22	0.7300	−0.42 [−0.80; −0.04]	65.1%	51.6%
**Common effect model**	**85**			**86**			**−0.07 [−0.38; 0.24]**	**100.0%**	**.**
**Random effects model**							**0.07 [−0.92; 1.06]**	**.**	**100.0%**
Heterogeneity: $I^2 = 89.2\%$, $\tau^2 = 0.4548$, $p = 0.0024$									
**PSYE+SCI+MTV+GS+RIF+PI+RD:ME**									
Taveras_2011	253	0.31	1.4300	192	0.49	1.3900	−0.18 [−0.44; 0.08]	100.0%	100.0%

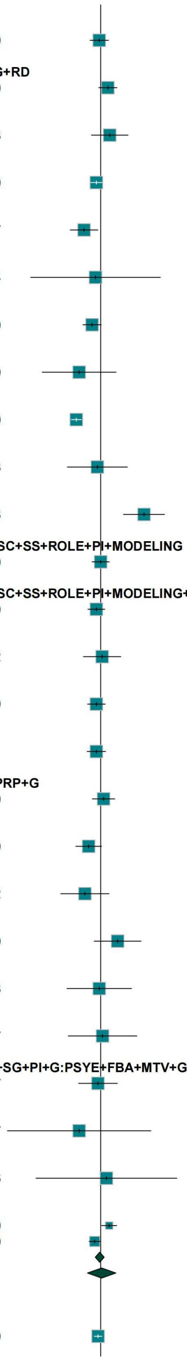

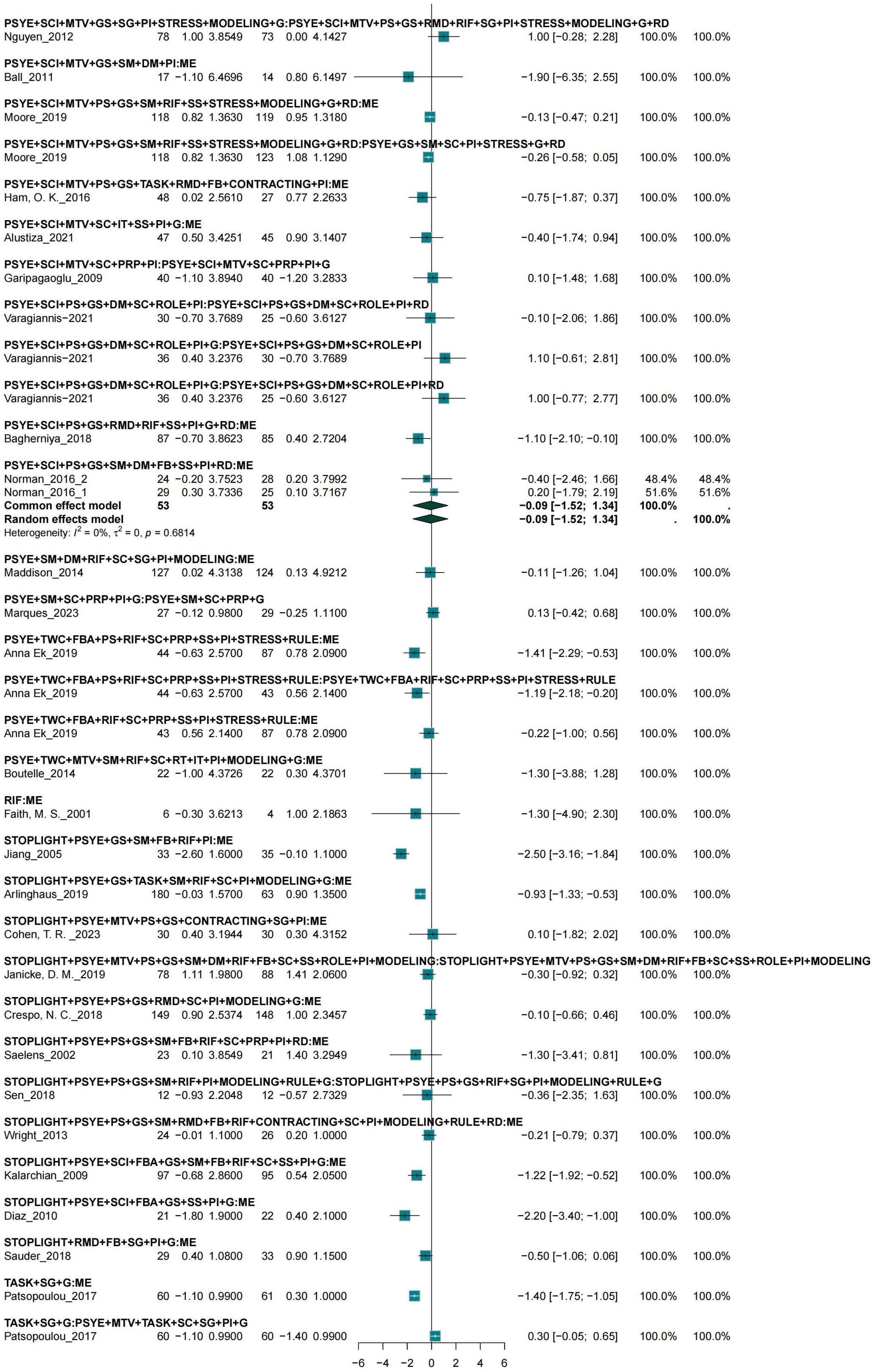

Notes: ME, minimal education; PSYE, psychoeducation; CR, cognitive restructuring; TWC, third-wave components; SCI, self-concept improvement; FBA, functional behavioural analysis; MTV, motivation; GS, goal-setting; TASK, task-setting; RULE, rule-setting; SM, self-monitoring; DM, device-monitoring; RMD, reminders; FB, feedback; PS, problem solving; PRP, preplanning; RIF, reinforcement; CONTRACTING, contracting; MODELING, modelling; SC, stimulus control; RT, relaxation training; IT, inhibition training; SS,

social support; STRESS, stress management; STOPLIGHT, stoplight approach; ROLE, role playing; G, group; RD, remote; SG, serious games; PI, parental involvement

8.2.5 Effect estimates for conceptual level and technical level components.

Components	Number of studies	Number of participants	MD (95% CI)	Certainty of evidence	P value
**Conceptual level**					
Behavioural therapy	38	3271	-0.65 (-0.89 to -0.42)	Low ⊕⊕○○	< 0.001
Cognitive behavioural therapy	18	1271	-0.45 (-0.79 to -0.11)	Low ⊕⊕○○	0.009
Psycho-education	4	198	-0.40 (-1.22 to 0.42)	Moderate ⊕⊕⊕○	0.335
**Technical level**					
Preplanning	12	774	-1.06 (-1.98 to -0.14)	Moderate ⊕⊕⊕○	0.025
Self-monitoring	29	2246	-0.59 (-1.15 to -0.03)	Moderate ⊕⊕⊕○	0.038
Reinforcement	28	2420	-0.53 (-1.03 to -0.03)	Low ⊕⊕○○	0.036
Third-wave components	4	165	-0.45 (-2.06 to 1.16)	Low ⊕⊕○○	0.587
Task-setting	13	908	-0.32 (-0.86 to 0.22)	Low ⊕⊕○○	0.247
Problem solving	44	2255	-0.31 (-0.82 to 0.19)	Low ⊕⊕○○	0.221
Serious games	15	815	-0.3 (-0.81 to 0.2)	Low ⊕⊕○○	0.235
Functional behavioral analysis	6	400	-0.21 (-1.02 to 0.6)	Low ⊕⊕○○	0.615
Inhibition training	4	106	-0.19 (-2.03 to 1.65)	Very low ⊕○○○	0.837
Stoplight approach	11	776	-0.18 (-0.83 to 0.46)	Low ⊕⊕○○	0.573
Motivation	32	2866	-0.14 (-0.61 to 0.34)	Low ⊕⊕○○	0.573
Social support	21	1506	-0.12 (-0.73 to 0.48)	Low ⊕⊕○○	0.686
Parental involvement	59	4966	-0.11 (-0.79 to 0.56)	Low ⊕⊕○○	0.745
Psycho-education	66	5848	-0.11 (-0.94 to 0.72)	Low ⊕⊕○○	0.793
Feedback	24	1788	-0.09 (-0.63 to 0.46)	Low ⊕⊕○○	0.751
Stimulus control	31	2263	-0.02 (-0.56 to 0.52)	Low ⊕⊕○○	0.952
Reminders	11	726	-0.01 (-0.62 to 0.59)	Low ⊕⊕○○	0.962
Group	45	3014	0.03 (-0.41 to 0.46)	Low ⊕⊕○○	0.908
Goal-setting	31	4008	0.05 (-0.57 to 0.66)	Low ⊕⊕○○	0.885
Remote	21	1643	0.07 (-0.51 to 0.65)	Moderate ⊕⊕⊕○	0.809
Relaxation training	3	56	0.09 (-1.78 to 1.96)	Low ⊕⊕○○	0.926
Contracting	7	434	0.36 (-0.39 to 1.11)	Low ⊕⊕○○	0.347
Self-concept improvement	12	1414	0.39 (-0.24 to 1.02)	Low ⊕⊕○○	0.223
Modeling	16	1283	0.4 (-0.27 to 1.07)	Low ⊕⊕○○	0.246
Role playing	3	369	0.51 (-1.06 to 2.08)	Low ⊕⊕○○	0.523
Cognitive restructuring	9	518	0.79 (-0.36 to 1.93)	Moderate ⊕⊕⊕○	0.177
Stress management	7	811	0.89 (-0.03 to 1.8)	Low ⊕⊕○○	0.058
Rule-setting	6	486	0.97 (-0.08 to 2.02)	Low ⊕⊕○○	0.069
Device-monitoring	13	940	1.04 (0.41 to 1.67)	Moderate ⊕⊕⊕○	0.001

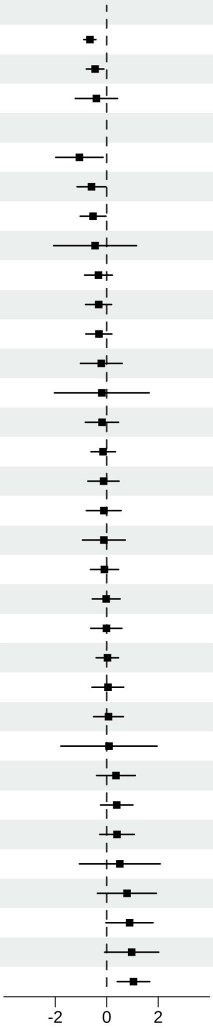

8.3 Heterogeneity (Inconsistency) assessed by pairwise meta-analyses

8.3.1 Heterogeneity (Inconsistency) assessment in conceptual NMA

Comparisons	Mean difference (95% CI)	Prediction Interval	k	Q	pval.Q	tau^2	I^2
BT vs CBT	-0.41 (-1.67 to 0.85)	NA	3	18.054	0	1.047	0.889
BT vs EDU	-0.21 (-0.94 to 0.53)	NA	4	4.276	0.233	0.219	0.298
BT vs ME	-0.63 (-0.92 to -0.34)	(-2.1 to 0.84)	33	168.901	0	0.498	0.811
CBT vs ME	-0.48 (-0.83 to -0.14)	(-1.62 to 0.65)	18	40.325	0.001	0.257	0.578
EDU vs ME	-0.23 (-2.32 to 1.86)	NA	1	0	NA	NA	NA

Notes: BT, behavioural therapy; CBT, cognitive behavioural therapy; CT, cognitive therapy; EDU, psychoeducation; ME, minimal education; k, no. of studies; Q, Q statistic; I, I statistic; tau, standard deviation of the random-effect distribution; CI, confidence interval

8.3.2 Heterogeneity (Inconsistency) assessment in technical CNMA

Comparisons	Mean difference (95% CI)	Prediction Interval	k	Q	pval.Q	tau^2	I^2
G vs ME	-0.29 (-0.94 to 0.36)	NA	4	5.082	0.166	0.162	0.41
PI vs ME	0.5 (-0.64 to 1.64)	NA	2	1.92	0.166	0.399	0.479
PSYE+SCI+PS+GS+SM+DM+FB+SS+PI+RD vs ME	-0.09 (-1.52 to 1.34)	NA	2	0.169	0.681	0	0
PSYE+SCI+GS+TASK+SM+DM+FB+RIF+PI+G vs ME	0.07 (-0.92 to 1.06)	NA	2	9.224	0.002	0.455	0.892
MTV vs ME	-0.01 (-3.33 to 3.31)	NA	2	0.05	0.823	0	0
PSYE+GS+SM+RIF+SC+SG+PI+MODELING+G vs ME	-1.3 (-2.51 to -0.08)	NA	2	3.465	0.063	0.574	0.711

Notes: ME, minimal education; PSYE, psychoeducation; CR, cognitive restructuring; TWC, third-wave components; SCI, self-concept improvement; FBA, functional behavioural analysis; MTV, motivation; GS, goal-setting; TASK, task-setting; RULE, rule-setting; SM, self-monitoring; DM, device-monitoring; RMD, reminders; FB, feedback; PS, problem solving; PRP, preplanning; RIF, reinforcement; CONTRACTING, contracting; MODELING, modelling; SC, stimulus control; RT, relaxation training; IT, inhibition training; SS, social support; STRESS, stress management; STOPLIGHT, stoplight approach; ROLE, role playing; G, group; RD, remote; SG, serious games; PI, parental involvement; k, no. of studies; Q, Q statistic; I, I statistic; tau, standard deviation of the random-effect distribution; CI, confidence interval

8.4 Publication bias assessments

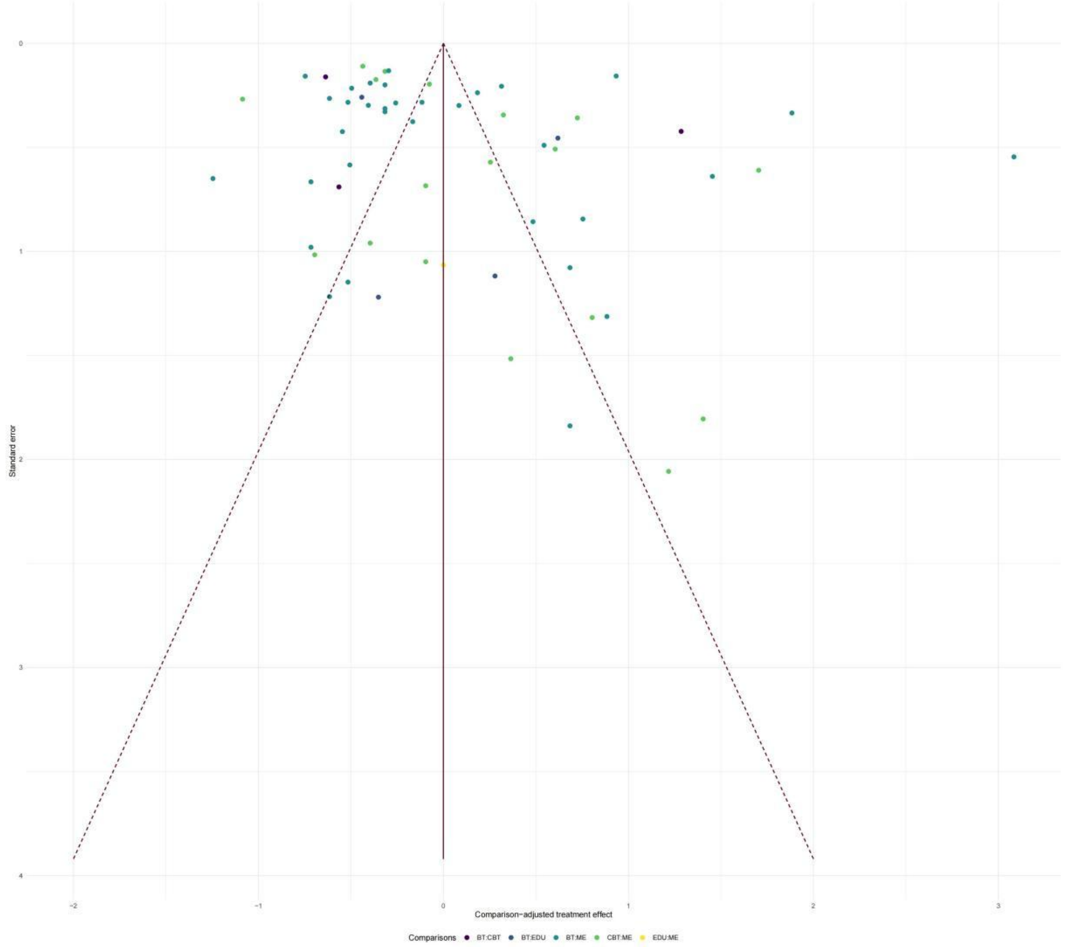

Notes: BT, behavioural therapy; CBT, cognitive behavioural therapy; CT, cognitive therapy; EDU, psychoeducation; ME, minimal education; k, no. of studies; Q, Q statistic; I, I statistic; tau, standard deviation of the random-effect distribution; CI, confidence interval

8.5 Intransitivity assessments

8.5.1 Age

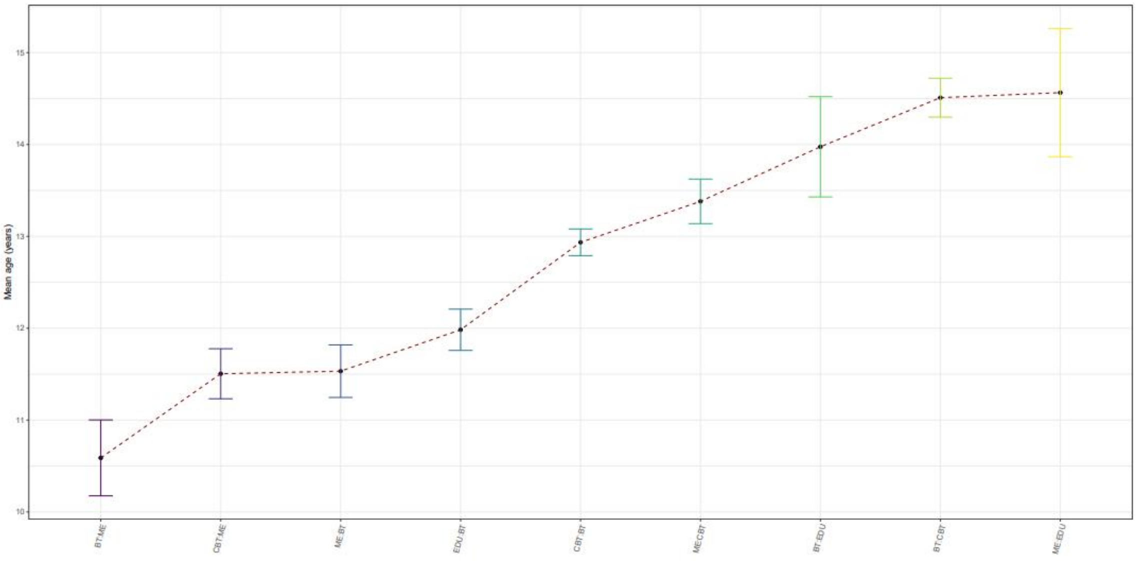

8.5.2 Body mass index

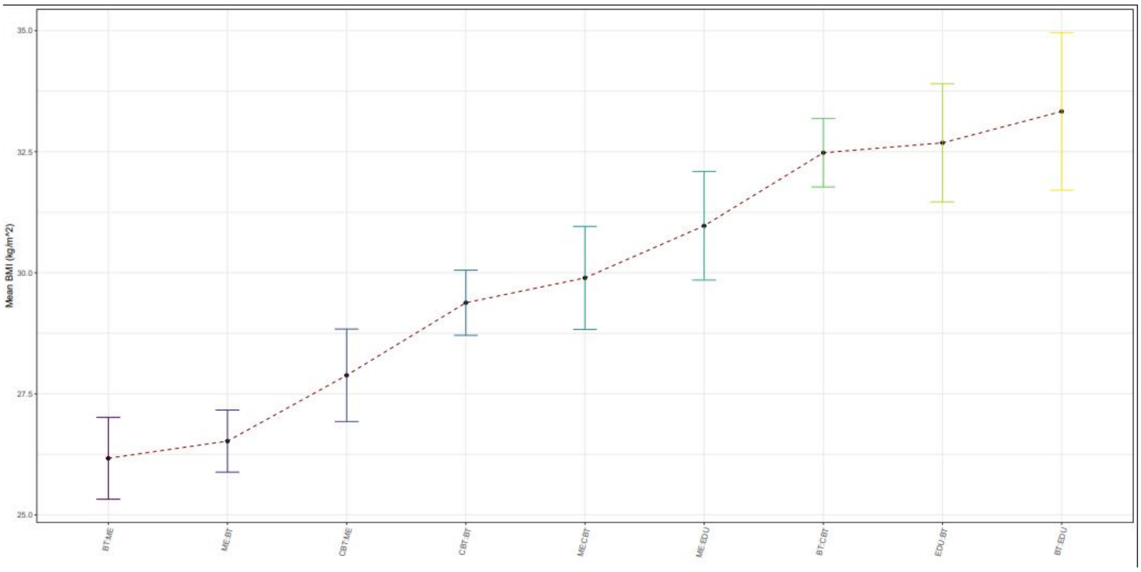

8.5.3 Duration of treatment

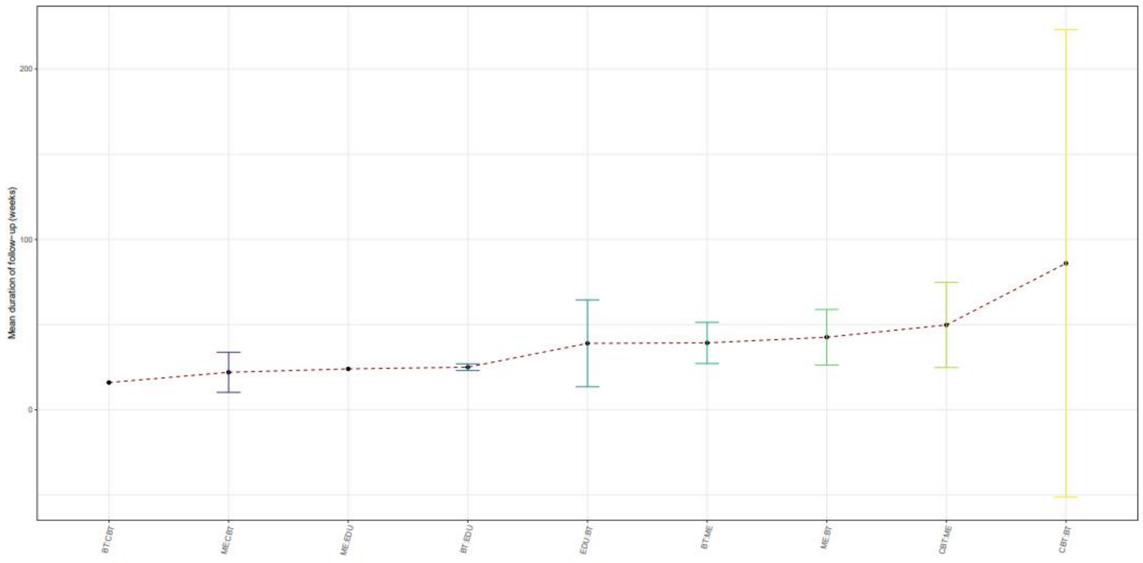

8.5.4 Proportion of girls

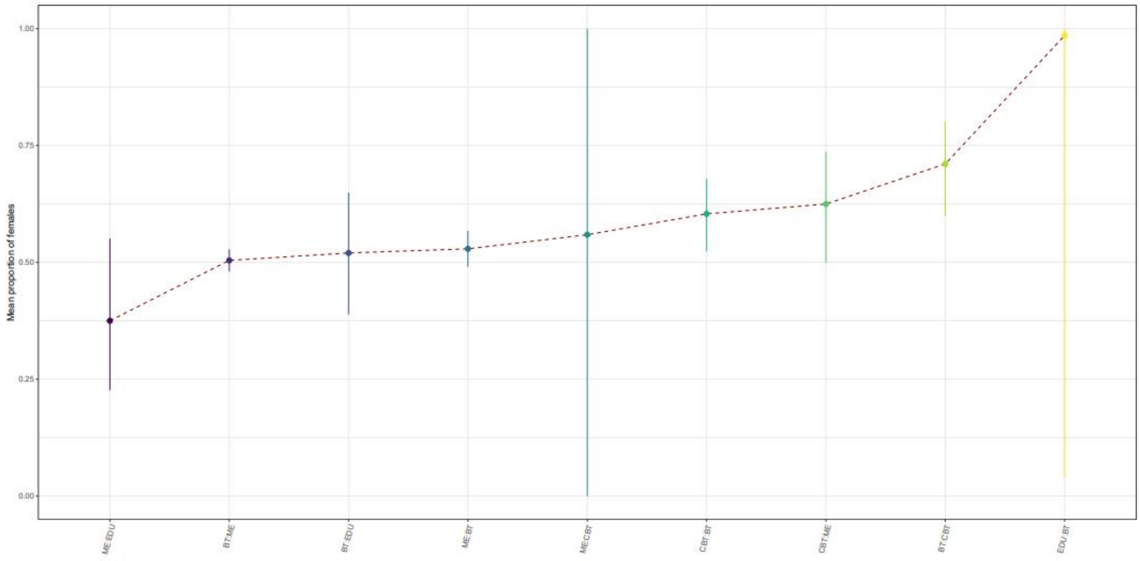

8.6 GRADE certainty of evidence assessments

8.6.1 GRADE assessment table in conceptual level NMA

Comparisons	k	Direct estimates (95% CI)	D1	D2	D3	D4	Certainty of direct evidence	Indirect estimates (95% CI)	Initial indirect certainty	D5	Certainty of indirect evidence	NMA estimates (95% CI)	Prop	initial NMA certainty	D6	D7	NMA certainty
BT vs ME	33	-0.62 (-0.86 to -0.37)	0	0	-1	0	Moderate	-1.29 (-2.29 to -0.28)	Moderate	0	Moderate	-0.65 (-0.89 to -0.42)	0.95	Moderate	0	-1	Low
CBT vs ME	18	-0.50 (-0.86 to -0.14)	0	0	-1	0	Moderate	-0.13 (-1.12 to 0.85)	Moderate	0	Moderate	-0.45 (-0.79 to -0.11)	0.88	Moderate	0	-1	Low
BT vs EDU	4	-0.22 (-1.03 to 0.59)	-1	0	0	0	Moderate	-0.88 (-4.59 to 2.83)	Moderate	0	Moderate	-0.25 (-1.04 to 0.54)	0.95	Moderate	0	0	Moderate
BT vs CBT	3	-0.38 (-1.15 to 0.40)	0	0	-1	0	Moderate	-0.14 (-0.59 to 0.31)	Moderate	0	Moderate	-0.20 (-0.59 to 0.19)	0.25	Moderate	NA	-1	Low
EDU vs ME	1	-0.23 (-2.58 to 2.12)	0	0	0	0	High	-0.43 (-1.30 to 0.45)	Moderate	0	Moderate	-0.40 (-1.22 to 0.42)	0.12	Moderate	0	0	Moderate
CBT vs EDU	0	NA	NA	NA	NA	NA	NA	-0.05 (-0.93 to 0.82)	Moderate	0	Moderate	-0.05 (-0.93 to 0.82)	0	Moderate	0	-1	Low

Notes: BT, behavioural therapy; CBT, cognitive behavioural therapy; CT, cognitive therapy; EDU, psychoeducation; ME, minimal education; D1, risk of bias, D2, indirectness, D3, heterogeneity, D4, publication bias, D5, intransitivity, D6, incoherence, D7, imprecision, k, number of studies, NMA, network meta-analysis, Prop, proportion of contribution of direct estimates to network estimates

8.6.2 GRADE assessment table in technical level CNMA

Components	Number of studies	Number of participants	Direct estimates (95% CI)	Weight of direct evidence (%)	Certainty of direct evidence	Additive estimates (95% CI)	Weight of additive evidence (%)	Certainty of additive evidence	Component estimates (95% CI)	High certainty evidence prop.	Moderate certainty evidence prop.	Low certainty evidence prop.	Very low certainty evidence prop.	Initial GRADE	Inadditivity	Incoherence	Imprecision	Final GRADE
contracting	7	434	NA	0%	NA	0.36 (-0.39 to 1.11)	100%	Low	0.36 (-0.39 to 1.11)	12%	57%	24%	0%	Moderate	0	0	-1	Low
cognitive restructuring	9	518	NA	0%	NA	0.79 (-0.36 to 1.93)	100%	Low	0.79 (-0.36 to 1.93)	43%	42%	12%	1%	High	0	0	-1	Moderate
device-monitoring	13	940	NA	0%	NA	1.04 (0.41 to 1.67)	100%	Low	1.04 (0.41 to 1.67)	10%	72%	16%	2%	Moderate	0	0	0	Moderate
feedback	24	1788	NA	0%	NA	-0.09 (-0.63 to 0.46)	100%	Low	-0.09 (-0.63 to 0.46)	7%	68%	23%	2%	Moderate	0	0	-1	Low
functional behavioural analysis	6	400	NA	0%	NA	-0.21 (-1.02 to 0.60)	100%	Low	-0.21 (-1.02 to 0.60)	5%	64%	29%	1%	Moderate	0	0	-1	Low
group	45	3014	NA	0%	NA	0.03 (-0.41 to 0.46)	100%	Low	0.03 (-0.41 to 0.46)	6%	65%	24%	2%	Moderate	0	0	-1	Low
goal-setting	31	4008	NA	0%	NA	0.05 (-0.57 to 0.66)	100%	Low	0.05 (-0.57 to 0.66)	15%	62%	19%	0%	Moderate	0	0	-1	Low
inhibition training	4	106	NA	0%	NA	-0.19 (-2.03 to 1.65)	100%	Low	-0.19 (-2.03 to 1.65)	1%	21%	71%	0%	Low	0	0	-1	Very low
modelling	16	1283	NA	0%	NA	0.40 (-0.27 to 1.07)	100%	Low	0.40 (-0.27 to 1.07)	9%	64%	24%	1%	Moderate	0	0	-1	Low
motivation	32	2866	NA	0%	NA	-0.14 (-0.61 to 0.34)	100%	Low	-0.14 (-0.61 to 0.34)	7%	66%	25%	0%	Moderate	0	0	-1	Low
parental involvement	59	4966	NA	0%	NA	-0.11 (-0.79 to 0.56)	100%	Low	-0.11 (-0.79 to 0.56)	6%	55%	35%	2%	Moderate	0	0	-1	Low
preplanning	12	774	NA	0%	NA	-1.06 (-1.98 to -0.14)	100%	Low	-1.06 (-1.98 to -0.14)	5%	72%	14%	1%	Moderate	0	0	0	Moderate
problem solving	44	2255	NA	0%	NA	-0.31 (-0.82 to 0.19)	100%	Low	-0.31 (-0.82 to 0.19)	8%	66%	21%	3%	Moderate	0	0	-1	Low
psychoeducation	66	5848	NA	0%	NA	-0.11 (-0.94 to 0.72)	100%	Low	-0.11 (-0.94 to 0.72)	4%	56%	37%	2%	Moderate	0	0	-1	Low
remote	21	1643	NA	0%	NA	0.07 (-0.51 to 0.65)	100%	Low	0.07 (-0.51 to 0.65)	6%	66%	21%	2%	Moderate	0	0	-1	Low
reinforcement	28	2420	NA	0%	NA	-0.53 (-1.03 to -0.03)	100%	Low	-0.53 (-1.03 to -0.03)	7%	58%	32%	0%	Moderate	0	0	0	Moderate
reminders	11	726	NA	0%	NA	-0.01 (-0.62 to 0.59)	100%	Low	-0.01 (-0.62 to 0.59)	8%	59%	31%	1%	Moderate	0	0	-1	Low
role playing	3	369	NA	0%	NA	0.51 (-1.06 to 2.08)	100%	Low	0.51 (-1.06 to 2.08)	6%	69%	19%	1%	Moderate	0	0	-1	Low
relaxation training	3	56	NA	0%	NA	0.09 (-1.78	100%	Low	0.09 (-1.78 to	9%	78%	11%	0%	Moderate	0	0	-1	Low

Components	Number of studies	Number of participants	Direct estimates (95% CI)	Weight of direct evidence (%)	Certainty of direct evidence	Additive estimates (95% CI)	Weight of additive evidence (%)	Certainty of additive evidence	Component estimates (95% CI)	High certainty evidence prop.	Moderate certainty evidence prop.	Low certainty evidence prop.	Very low certainty evidence prop.	Initial GRADE	Inadditivity	Incoherence	Imprecision	Final GRADE
						to 1.96)			1.96)									
rule-setting	6	486	NA	0%	NA	0.97 (-0.08 to 2.02)	100%	Low	0.97 (-0.08 to 2.02)	15%	43%	37%	0%	Moderate	0	0	-1	Low
stimulus control	31	2263	NA	0%	NA	-0.02 (-0.56 to 0.52)	100%	Low	-0.02 (-0.56 to 0.52)	5%	66%	23%	1%	Moderate	0	0	-1	Low
self-concept improvement	12	1414	NA	0%	NA	0.39 (-0.24 to 1.02)	100%	Low	0.39 (-0.24 to 1.02)	5%	73%	21%	2%	Moderate	0	0	-1	Low
serious games	15	815	NA	0%	NA	-0.30 (-0.81 to 0.20)	100%	Low	-0.30 (-0.81 to 0.20)	5%	70%	16%	7%	Moderate	0	0	-1	Low
self-monitoring	29	2246	NA	0%	NA	-0.59 (-1.15 to -0.03)	100%	Low	-0.59 (-1.15 to -0.03)	6%	70%	16%	4%	Moderate	0	0	0	Moderate
social support	21	1506	NA	0%	NA	-0.12 (-0.73 to 0.48)	100%	Low	-0.12 (-0.73 to 0.48)	6%	72%	22%	1%	Moderate	0	0	-1	Low
stoplight approach	11	776	NA	0%	NA	-0.18 (-0.83 to 0.46)	100%	Low	-0.18 (-0.83 to 0.46)	7%	70%	15%	0%	Moderate	0	0	-1	Low
stress management	7	811	NA	0%	NA	0.89 (-0.03 to 1.80)	100%	Low	0.89 (-0.03 to 1.80)	5%	73%	21%	1%	Moderate	0	0	-1	Low
task-setting	13	908	NA	0%	NA	-0.32 (-0.86 to 0.22)	100%	Low	-0.32 (-0.86 to 0.22)	9%	73%	14%	1%	Moderate	0	0	-1	Low
third-wave components	4	165	NA	0%	NA	-0.45 (-2.06 to 1.16)	100%	Low	-0.45 (-2.06 to 1.16)	19%	56%	24%	0%	Moderate	0	0	-1	Low

Notes: ME, minimal education; PSYE, psychoeducation; CR, cognitive restructuring; TWC, third-wave components; SCI, self-concept improvement; FBA, functional behavioural analysis; MTV, motivation; GS, goal-setting; TASK, task-setting; RULE, rule-setting; SM, self-monitoring; DM, device-monitoring; RMD, reminders; FB, feedback; PS, problem solving; PRP, preplanning; RIF, reinforcement; CONTRACTING, contracting; MODELING, modelling; SC, stimulus control; RT, relaxation training; IT, inhibition training; SS, social support; STRESS, stress management; STOPLIGHT, stoplight approach; ROLE, role playing; G, group; RD, remote; SG, serious games; PI, parental involvement

8.6.3 Percentage contribution matrix for conceptual level NMA

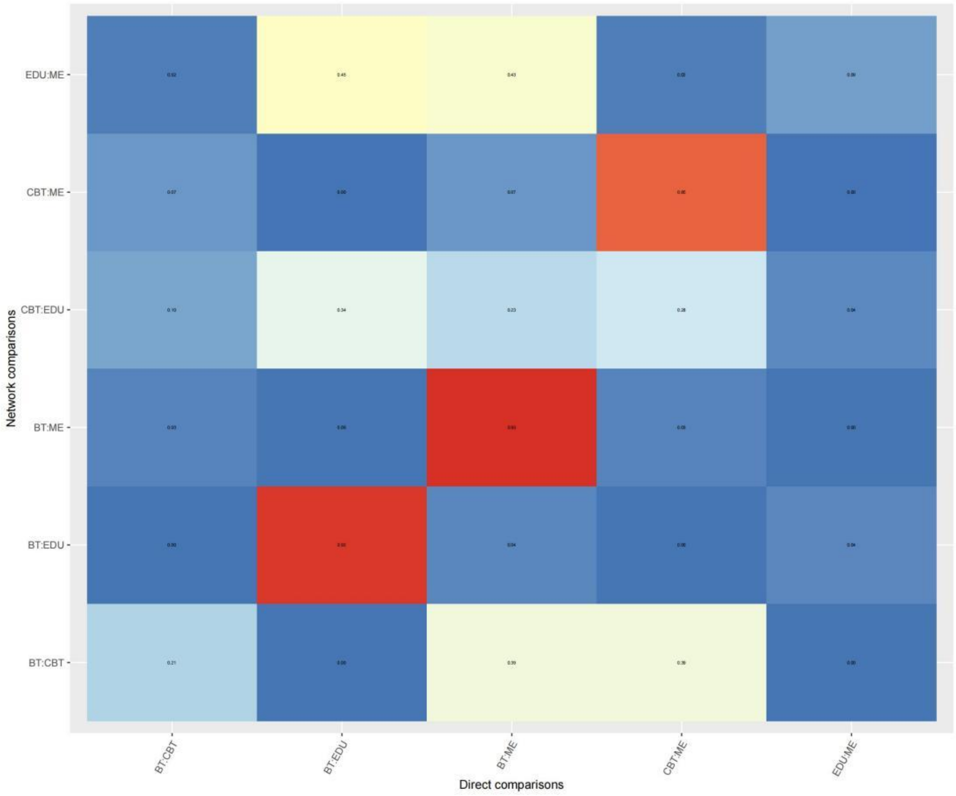

Notes: BT, behavioural therapy; CBT, cognitive behavioural therapy; CT, cognitive therapy; EDU, psychoeducation; ME, minimal education

8.6.4 Percentage contribution matrix for technical level CNMA

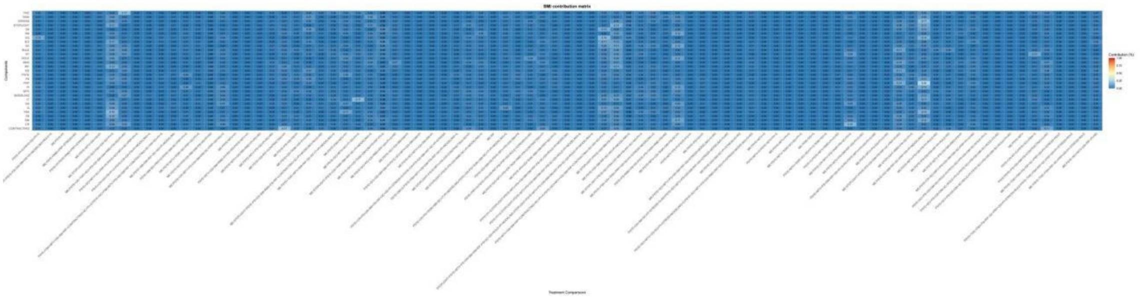

Notes: ME, minimal education; PSYE, psychoeducation; CR, cognitive restructuring; TWC, third-wave components; SCI, self-concept improvement; FBA, functional behavioural analysis; MTV, motivation; GS, goal-setting; TASK, task-setting; RULE, rule-setting; SM, self-monitoring; DM, device-monitoring; RMD, reminders; FB, feedback; PS, problem solving; PRP, preplanning; RIF, reinforcement; CONTRACTING, contracting; MODELING, modelling; SC, stimulus control; RT, relaxation training; IT, inhibition training; SS, social support; STRESS, stress management; STOPLIGHT, stoplight approach; ROLE, role playing; G, group; RD, remote; SG, serious games; PI, parental involvement

8.7 Subgroup analyses

8.7.1 Subgroup analyses for the conceptual level NMA

8.7.1.1 Subgroup analysis for patients' age group at baseline

Comparisons	Adolescents	Children	Difference in difference	Interactive p value
BT vs CBT	-0.57 (-1.24 to 0.10)	0.03 (-0.30 to 0.36)	-0.60 (-0.06 to 0.93)	0.112
BT vs EDU	-0.66 (-1.94 to 0.63)	0.22 (-0.43 to 0.87)	-0.88 (-0.45 to 1.85)	0.233
BT vs ME	-1.12 (-1.60 to -0.64)	-0.35 (-0.52 to -0.17)	**-0.78 (-1.29 to -0.26)**	0.003
CBT vs EDU	-0.08 (-1.51 to 1.35)	0.19 (-0.53 to 0.91)	-0.27 (-0.04 to 3.58)	0.739
CBT vs ME	-0.55 (-1.13 to 0.03)	-0.38 (-0.65 to -0.10)	-0.17 (-0.86 to 0.43)	0.597
EDU vs ME	-0.47 (-1.81 to 0.88)	-0.57 (-1.24 to 0.10)	0.10 (1.32 to 2.75)	0.895

Notes: BT, behavioural therapy; CBT, cognitive behavioural therapy; CT, cognitive therapy; ME, minimal education

8.7.1.2 Subgroup analysis for patients' BMI at baseline

Comparisons	Obesity	Overweight	Difference in difference	Interactive p value
BT vs CBT	-0.14 (-0.61 to 0.33)	-0.30 (-0.90 to 0.30)	0.16 (0.40 to 1.44)	0.678
BT vs ME	-0.75 (-1.03 to -0.46)	-0.32 (-0.66 to 0.01)	-0.42 (-0.27 to 0.41)	0.060
CBT vs ME	-0.61 (-1.03 to -0.20)	-0.03 (-0.52 to 0.47)	-0.59 (-0.96 to 0.34)	0.076

Notes: BT, behavioural therapy; CBT, cognitive behavioural therapy; CT, cognitive therapy; ME, minimal education.

8.7.1.3 Subgroup analysis for treatment settings

Comparisons	School setting	Other settings	Difference in difference	Interactive p value
BT vs CBT	-0.98 (-1.85 to -0.12)	-0.02 (-0.45 to 0.41)	-0.96 (-0.26 to 1.02)	0.051
BT vs ME	-1.29 (-1.85 to -0.73)	-0.50 (-0.75 to -0.24)	**-0.79 (-0.62 to 0.38)**	0.011
CBT vs ME	-0.30 (-0.97 to 0.36)	-0.47 (-0.86 to -0.09)	0.17 (-0.49 to 1.08)	0.663

Notes: BT, behavioural therapy; CBT, cognitive behavioural therapy; CT, cognitive therapy; ME, minimal education.

8.7.2 Subgroup analyses for the technical level CNMA

8.7.2.1 Subgroup analysis for patients' age group at baseline

**Components**	**Adolescents**			**Children**			**Difference in difference (95% CI)**	**Interactive p value**
	No. of studies	MD (95% CI)	p.value	No. of studies	MD (95% CI)	p.value		
contracting	3	-0.27 (-3.58 to 3.03)	0.871	4	0.47 (-0.5 to 1.45)	0.339	-0.75 (-4.19 to 2.7)	0.670
cognitive restructuring	6	0.69 (-2.96 to 4.35)	0.710	2	6.99 (-2.94 to 16.92)	0.167	-6.3 (-16.88 to 4.28)	0.243
device-monitoring	14	0.65 (-0.87 to 2.18)	0.400	8	5.79 (1.38 to 10.2)	0.010	-5.14 (-9.8 to -0.48)	0.031
feedback	14	0.55 (-0.73 to 1.84)	0.399	14	-0.15 (-1.3 to 0.99)	0.794	0.71 (-1.02 to 2.43)	0.421
functional behavioural analysis	4	0.35 (-1.46 to 2.15)	0.705	6	3.55 (-4.1 to 11.2)	0.363	-3.2 (-11.06 to 4.65)	0.424
group	34	-0.56 (-1.67 to 0.54)	0.317	18	0.53 (-0.1 to 1.17)	0.098	-1.1 (-2.37 to 0.17)	0.091
goal-setting	30	-0.02 (-2.38 to 2.34)	0.987	27	0.55 (-0.98 to 2.08)	0.480	-0.57 (-3.38 to 2.24)	0.691
inhibition training	1	0.67 (-2.55 to 3.89)	0.685	2	-2.82 (-10.7 to 5.05)	0.482	3.49 (-5.02 to 12)	0.422
modelling	7	-0.62 (-2.39 to 1.15)	0.493	12	3.48 (-4.07 to 11.02)	0.367	-4.1 (-11.85 to 3.66)	0.301
motivation	25	-0.32 (-1.51 to 0.87)	0.601	17	-0.33 (-1.62 to 0.96)	0.619	0.01 (-1.75 to 1.77)	0.992
parental involvement	40	-0.29 (-1.4 to 0.83)	0.616	38	-1.07 (-4.88 to 2.74)	0.581	0.79 (-3.18 to 4.75)	0.698
preplanning	10	-0.34 (-2.08 to 1.39)	0.700	5	-2.67 (-9.77 to 4.43)	0.461	2.33 (-4.98 to 9.64)	0.532
problem solving	21	-0.42 (-1.96 to 1.11)	0.590	21	-0.73 (-1.49 to 0.04)	0.063	0.3 (-1.41 to 2.02)	0.729
psychoeducation	50	0.12 (-1.46 to 1.7)	0.880	37	0.93 (-3.74 to 5.6)	0.697	-0.8 (-5.73 to 4.13)	0.749
remote	19	0.57 (-1.29 to 2.43)	0.548	10	0.26 (-0.95 to 1.48)	0.674	0.31 (-1.91 to 2.53)	0.785
reinforcement	17	0.23 (-1.05 to 1.52)	0.724	17	-0.84 (-2.27 to 0.59)	0.248	1.07 (-0.85 to 2.99)	0.274
reminders	7	-1.13 (-3.37 to 1.1)	0.321	7	-0.08 (-1.34 to 1.18)	0.899	-1.05 (-3.62 to 1.52)	0.422
role playing	1	-1.79 (-6.17 to 2.59)	0.423	6	-2.37 (-8.85 to 4.11)	0.474	0.58 (-7.24 to 8.4)	0.885
relaxation training	1	0.61 (-4.1 to 5.32)	0.800	1	-2.82 (-10.7 to 5.05)	0.482	3.43 (-5.75 to 12.61)	0.464
rule-setting	—	—	—	7	0.97 (-1.07 to 3.01)	0.353	—	—
stimulus control	17	-0.28 (-1.87 to 1.31)	0.730	19	-5.79 (-16 to 4.43)	0.267	5.51 (-4.84 to 15.85)	0.297
self-concept improvement	18	0.34 (-0.82 to 1.51)	0.562	7	0.27 (-1.27 to 1.81)	0.735	0.08 (-1.85 to 2.01)	0.936
serious games	10	0.61 (-0.7 to 1.92)	0.359	6	-2 (-3.36 to -0.64)	0.004	**2.61 (0.73 to 4.49)**	0.007
self-monitoring	25	-0.69 (-2.73 to 1.36)	0.512	13	-0.6 (-1.77 to 0.56)	0.311	-0.08 (-2.44 to 2.27)	0.944
social support	18	-0.09 (-1.42 to 1.25)	0.900	9	-0.87 (-3.22 to 1.49)	0.470	0.78 (-1.92 to 3.49)	0.571
stoplight approach	3	-1.98 (-4.22 to 0.26)	0.083	10	2.27 (-0.71 to 5.25)	0.136	**-4.25 (-7.98 to -0.52)**	0.025
stress management	8	1.16 (-0.97 to 3.3)	0.286	4	-2.67 (-9.77 to 4.43)	0.461	3.84 (-3.58 to 11.25)	0.311
task-setting	9	-0.59 (-2.04 to 0.86)	0.422	6	-0.45 (-1.71 to 0.81)	0.481	-0.14 (-2.06 to 1.78)	0.886
third-wave components	1	-1.17 (-6.45 to 4.12)	0.666	4	8.04 (-9.63 to 25.71)	0.373	-9.21 (-27.66 to 9.24)	0.328

8.7.2.2 Subgroup analysis for patients' BMI at baseline

Components	Obesity			Overweight			Difference in difference (95% CI)	Interactive p value
	No. of studies	MD (95% CI)	p.value	No. of studies	MD (95% CI)	p.value		
contracting	6	0.11 (-1.09 to 1.3)	0.859	1	0.4 (-0.41 to 1.2)	0.334	-0.29 (-1.73 to 1.15)	0.695
cognitive restructuring	7	0.54 (-1.17 to 2.25)	0.534	1	0.2 (-0.28 to 0.68)	0.410	0.34 (-1.44 to 2.12)	0.707
device-monitoring	19	0.78 (-0.03 to 1.58)	0.060	3	0.42 (-0.11 to 0.94)	0.120	0.36 (-0.6 to 1.32)	0.462
feedback	24	-0.02 (-0.74 to 0.69)	0.946	4	0.05 (-0.93 to 1.04)	0.917	-0.08 (-1.29 to 1.14)	0.901
functional behavioural analysis	10	-0.22 (-1.21 to 0.77)	0.660	—	—	—	—	—
group	46	-0.07 (-0.63 to 0.48)	0.800	6	-0.06 (-0.39 to 0.27)	0.715	-0.01 (-0.66 to 0.64)	0.976
goal-setting	48	-0.03 (-0.88 to 0.82)	0.947	9	0.01 (-0.45 to 0.48)	0.953	-0.04 (-1.01 to 0.92)	0.931
inhibition training	2	0.34 (-4.87 to 5.54)	0.899	1	-0.03 (-0.49 to 0.43)	0.896	0.37 (-4.86 to 5.59)	0.891
modelling	17	0.46 (-0.47 to 1.38)	0.331	2	-0.45 (-0.99 to 0.09)	0.105	0.91 (-0.17 to 1.98)	0.098
motivation	38	-0.14 (-0.74 to 0.47)	0.658	4	-0.15 (-0.73 to 0.43)	0.609	0.01 (-0.82 to 0.85)	0.972
parental involvement	69	-0.16 (-0.98 to 0.65)	0.693	9	-0.22 (-0.67 to 0.23)	0.340	0.05 (-0.88 to 0.99)	0.909
preplanning	14	-1.14 (-2.17 to -0.1)	0.032	1	0.2 (-0.28 to 0.68)	0.410	**-1.34 (-2.48 to -0.2)**	0.022
problem solving	38	-0.22 (-0.89 to 0.44)	0.509	4	0.29 (-0.35 to 0.93)	0.371	-0.51 (-1.43 to 0.41)	0.273
psychoeducation	77	-0.11 (-1.08 to 0.87)	0.832	10	-0.02 (-0.27 to 0.23)	0.895	-0.09 (-1.09 to 0.91)	0.862
remote	27	0.18 (-0.56 to 0.92)	0.633	2	-0.22 (-1.01 to 0.57)	0.588	0.4 (-0.68 to 1.48)	0.471
reinforcement	29	-0.6 (-1.31 to 0.11)	0.097	5	-0.17 (-0.73 to 0.38)	0.542	-0.43 (-1.33 to 0.48)	0.353
reminders	13	-0.04 (-0.79 to 0.71)	0.916	1	0.31 (-0.16 to 0.77)	0.195	-0.35 (-1.23 to 0.53)	0.439
role playing	7	0.29 (-1.68 to 2.25)	0.775	—	—	—	—	—
relaxation training	2	0.08 (-2.77 to 2.93)	0.955	—	—	—	—	—
rule-setting	6	1.04 (-0.48 to 2.56)	0.181	1	-0.03 (-0.74 to 0.68)	0.936	1.07 (-0.61 to 2.75)	0.213
stimulus control	32	0.15 (-0.58 to 0.88)	0.681	4	-0.28 (-0.74 to 0.18)	0.241	0.43 (-0.43 to 1.29)	0.330
self-concept improvement	22	0.24 (-0.65 to 1.12)	0.598	3	0.18 (-0.35 to 0.71)	0.500	0.05 (-0.98 to 1.09)	0.917
serious games	14	-0.21 (-0.85 to 0.44)	0.532	2	-0.55 (-1.15 to 0.04)	0.068	0.35 (-0.53 to 1.22)	0.439
self-monitoring	34	-0.6 (-1.38 to 0.19)	0.136	4	-0.34 (-0.89 to 0.21)	0.231	-0.26 (-1.22 to 0.7)	0.596
social support	25	0.02 (-0.89 to 0.92)	0.970	2	0.17 (-0.44 to 0.78)	0.584	-0.15 (-1.25 to 0.94)	0.782
stoplight approach	12	-0.15 (-0.98 to 0.69)	0.726	1	0.31 (-0.16 to 0.77)	0.195	-0.46 (-1.41 to 0.5)	0.349
stress management	12	0.87 (-0.27 to 2)	0.133	—	—	—	—	—
task-setting	13	-0.33 (-1.07 to 0.4)	0.373	2	0.21 (-0.21 to 0.64)	0.328	-0.55 (-1.4 to 0.3)	0.207
third-wave components	5	-0.65 (-2.94 to 1.63)	0.574	—	—	—	—	—

8.7.2.3 Subgroup analysis for treatment settings

Components	School setting			Other settings			Difference in difference (95% CI)	Interactive p value
	No. of studies	MD (95% CI)	p.value	No. of studies	MD (95% CI)	p.value		
contracting	1	0.7 (-0.88 to 2.29)	0.385	6	0.37 (-0.5 to 1.24)	0.407	0.34 (-1.47 to 2.15)	0.716
cognitive restructuring	1	0.33 (-0.36 to 1.01)	0.350	7	0.85 (-0.51 to 2.21)	0.219	-0.52 (-2.04 to 0.99)	0.498
device-monitoring	3	0.48 (-0.03 to 0.98)	0.065	19	0.69 (-0.03 to 1.41)	0.062	-0.21 (-1.09 to 0.67)	0.638
feedback	6	-0.05 (-0.61 to 0.5)	0.852	22	0.09 (-0.5 to 0.67)	0.773	-0.14 (-0.95 to 0.67)	0.735
functional behavioural analysis	1	0.29 (-0.18 to 0.76)	0.229	9	-0.23 (-1.13 to 0.67)	0.614	0.52 (-0.49 to 1.54)	0.314
group	10	0.5 (-0.08 to 1.08)	0.090	42	-0.15 (-0.63 to 0.32)	0.528	0.65 (-0.1 to 1.41)	0.087
goal-setting	12	0.3 (-0.27 to 0.87)	0.304	45	0.07 (-0.61 to 0.75)	0.847	0.23 (-0.66 to 1.12)	0.610
inhibition training	—	—	—	3	0.03 (-1.96 to 2.03)	0.975	—	—
modelling	4	0.78 (0.06 to 1.51)	0.035	15	0.13 (-0.66 to 0.92)	0.745	0.65 (-0.42 to 1.73)	0.232
motivation	3	0.21 (-0.23 to 0.66)	0.353	39	-0.2 (-0.75 to 0.35)	0.474	0.41 (-0.3 to 1.12)	0.254
parental involvement	11	-0.29 (-0.82 to 0.24)	0.278	67	-0.22 (-0.92 to 0.48)	0.532	-0.07 (-0.95 to 0.81)	0.878
preplanning	3	0.62 (-0.29 to 1.52)	0.182	12	-1.27 (-2.27 to -0.27)	0.013	**1.89 (0.54 to 3.24)**	0.006
problem solving	5	-0.28 (-1 to 0.43)	0.438	37	-0.27 (-0.78 to 0.24)	0.298	-0.02 (-0.9 to 0.86)	0.973
psychoeducation	13	0.03 (-0.75 to 0.82)	0.932	74	0.08 (-0.73 to 0.9)	0.839	-0.05 (-1.18 to 1.08)	0.931
remote	2	-0.5 (-1.16 to 0.17)	0.144	27	0.16 (-0.51 to 0.82)	0.644	-0.65 (-1.59 to 0.29)	0.174
reinforcement	8	-0.2 (-0.77 to 0.37)	0.485	26	-0.39 (-0.97 to 0.2)	0.195	0.18 (-0.63 to 1)	0.661
reminders	3	-0.87 (-1.93 to 0.18)	0.103	11	0.14 (-0.7 to 0.97)	0.749	-1.01 (-2.35 to 0.33)	0.140
role playing	1	0.26 (-0.2 to 0.73)	0.267	6	0.79 (-1.42 to 3.01)	0.484	-0.53 (-2.79 to 1.73)	0.647
relaxation training	—	—	—	2	-0.2 (-2.44 to 2.05)	0.863	—	—
rule-setting	—	—	—	7	0.54 (-0.52 to 1.6)	0.320	—	—
stimulus control	4	0.82 (-0.03 to 1.67)	0.058	32	0.05 (-0.59 to 0.7)	0.867	0.76 (-0.3 to 1.83)	0.159
self-concept improvement	4	0.09 (-0.61 to 0.8)	0.791	21	0.13 (-0.76 to 1.02)	0.775	-0.04 (-1.17 to 1.1)	0.951
serious games	5	-3.02 (-4.99 to -1.05)	0.003	11	0.06 (-0.63 to 0.75)	0.869	**-3.08 (-5.17 to -0.99)**	0.004
self-monitoring	8	-0.2 (-1.03 to 0.63)	0.635	30	-0.73 (-1.42 to -0.03)	0.041	0.53 (-0.55 to 1.61)	0.340
social support	4	0.12 (-0.42 to 0.66)	0.662	23	0.11 (-0.56 to 0.77)	0.757	0.02 (-0.85 to 0.88)	0.972
stoplight approach	2	-2.08 (-3.55 to -0.61)	0.005	11	-0.27 (-1.28 to 0.75)	0.607	**-1.82 (-3.6 to -0.03)**	0.046
stress management	2	0.55 (0.03 to 1.08)	0.038	10	0.95 (-0.07 to 1.96)	0.068	-0.39 (-1.54 to 0.75)	0.500
task-setting	5	-0.59 (-2.09 to 0.91)	0.442	10	-0.62 (-1.34 to 0.1)	0.094	0.03 (-1.63 to 1.69)	0.973
third-wave components	—	—	—	5	-0.35 (-2.1 to 1.4)	0.695	—	—

8.8 Sensitivity analyses

Notes: The study performed several sensitivity analyses by Frequentist method including (1) sensitivity 1: using end-of-follow-up data instead of end-of-treatment data for analyses if different; (2) sensitivity 2: excluding trials with treatment duration of less than 24 weeks; (3) sensitivity 3: excluding trials with high risk of bias; (4) sensitivity 4: excluding trials with imbalanced lifestyle modification; and (5) sensitivity 5: splitting waiting list from minimal education as a separate treatment component.

P value reflects the statistical significance of the effect difference between components under the null hypothesis of no difference from the control (minimal education). BT, behavioural therapy; CBT, cognitive behavioural therapy; CT, cognitive therapy; ME, minimal education

8.8.1 Sensitivity analyses for conceptual level NMA

Intervention	Comparator	Main estimate	Sensitivity 1	Sensitivity 2	Sensitivity 3	Sensitivity 4	Sensitivity 5
BT	CBT	-0.20 (-0.59, 0.19)	-0.17 (-0.65, 0.30)	0.06 (-0.38, 0.51)	-0.34 (-0.76, 0.07)	0.14 (-0.10, 0.37)	-0.20 (-0.59, 0.19)
BT	EDU	-0.25 (-1.04, 0.54)	-0.08 (-1.05, 0.89)	-0.24 (-0.98, 0.51)	-0.22 (-1.05, 0.60)	-0.07 (-0.56, 0.42)	-0.25 (-1.05, 0.54)
BT	ME	**-0.65 (-0.89, -0.42)**	**-0.67 (-0.96, -0.38)**	**-0.58 (-0.83, -0.34)**	**-0.68 (-0.92, -0.44)**	**-0.22 (-0.36, -0.07)**	**-0.66 (-0.91, -0.41)**
CBT	EDU	-0.05 (-0.93, 0.82)	0.09 (-0.98, 1.17)	-0.30 (-1.17, 0.57)	0.12 (-0.80, 1.04)	-0.21 (-0.75, 0.33)	-0.05 (-0.94, 0.83)
CBT	ME	**-0.45 (-0.79, -0.11)**	**-0.49 (-0.90, -0.08)**	**-0.65 (-1.04, -0.26)**	-0.33 (-0.70, 0.03)	**-0.35 (-0.55, -0.15)**	**-0.46 (-0.81, -0.11)**
EDU	ME	-0.40 (-1.22, 0.42)	-0.59 (-1.59, 0.41)	-0.35 (-1.13, 0.43)	-0.45 (-1.31, 0.40)	-0.14 (-0.65, 0.36)	-0.41 (-1.23, 0.42)

Notes: BT, behavioural therapy; CBT, cognitive behavioural therapy; CT, cognitive therapy; ME, minimal education.

8.8.2 Sensitivity analyses for technical level CNMA

**Components**	**Main estimate**		**Sensitivity 1**		**Sensitivity 2**		**Sensitivity 3**		**Sensitivity 4**		**Sensitivity 5**	
	No. of studies: 68		No. of studies: 68		No. of studies: 46		No. of studies: 61		No. of studies: 51		No. of studies: 68	
	Mean difference (95% CI)	p value	Mean difference (95% CI)	p value	Mean difference (95% CI)	p value	Mean difference (95% CI)	p value	Mean difference (95% CI)	p value	Mean difference (95% CI)	p value
contracting	0.36 (-0.39 to 1.11)	0.3469	0.34 (-0.59 to 1.27)	0.4735	0.27 (-0.75 to 1.30)	0.6017	0.37 (-0.43 to 1.17)	0.3616	0.26 (-0.21 to 0.72)	0.2858	0.32 (-0.46 to 1.09)	0.4249
cognitive restructuring	0.79 (-0.36 to 1.93)	0.1772	0.40 (-0.97 to 1.77)	0.5647	1.23 (-0.71 to 3.16)	0.2141	0.82 (-0.39 to 2.03)	0.1819	-0.25 (-2.29 to 1.78)	0.8067	0.78 (-0.38 to 1.93)	0.188
device-monitoring	**1.04 (0.41 to 1.67)**	0.0012	0.90 (0.12 to 1.68)	0.0239	1.22 (-0.07 to 2.51)	0.0641	**1.06 (0.37 to 1.74)**	0.0025	**1.11 (0.47 to 1.75)**	0.0007	**1.01 (0.37 to 1.66)**	0.002
feedback	-0.09 (-0.63 to 0.46)	0.7514	0.11 (-0.53 to 0.76)	0.7285	-0.11 (-0.82 to 0.60)	0.7598	-0.10 (-0.68 to 0.48)	0.7312	-0.37 (-0.75 to 0.00)	0.0525	-0.08 (-0.63 to 0.47)	0.7745
functional behavioural analysis	-0.21 (-1.02 to 0.60)	0.6148	-0.58 (-1.80 to 0.63)	0.347	0.14 (-0.99 to 1.28)	0.8021	-0.31 (-1.26 to 0.65)	0.5269	0.06 (-0.71 to 0.83)	0.8833	-0.24 (-1.08 to 0.59)	0.5666
group	0.03 (-0.41 to 0.46)	0.9078	0.04 (-0.46 to 0.54)	0.8812	0.27 (-0.39 to 0.94)	0.4218	-0.05 (-0.53 to 0.43)	0.8313	0.24 (-0.08 to 0.56)	0.1352	0.04 (-0.41 to 0.48)	0.8729
goal-setting	0.05 (-0.57 to 0.66)	0.8850	0.00 (-0.75 to 0.75)	0.9955	0.21 (-0.72 to 1.14)	0.655	0.01 (-0.64 to 0.67)	0.9665	0.36 (-0.15 to 0.86)	0.1661	0.12 (-0.56 to 0.80)	0.7314
inhibition training	-0.19 (-2.03 to 1.65)	0.8372	-0.28 (-2.43 to 1.87)	0.7988	0.79 (-1.84 to 3.41)	0.5571	-0.08 (-2.03 to 1.87)	0.936	0.04 (-1.56 to 1.65)	0.9589	-0.14 (-2.00 to 1.73)	0.8851
modelling	0.40 (-0.27 to 1.07)	0.2459	0.39 (-0.38 to 1.16)	0.3231	0.41 (-0.76 to 1.57)	0.4907	0.47 (-0.33 to 1.26)	0.2479	0.42 (-0.21 to 1.06)	0.1883	0.37 (-0.32 to 1.05)	0.2935
motivation	-0.14 (-0.61 to 0.34)	0.5726	-0.22 (-0.76 to 0.32)	0.4305	-0.16 (-0.93 to 0.61)	0.6784	-0.17 (-0.70 to 0.36)	0.5277	-0.12 (-0.46 to 0.23)	0.5124	-0.12 (-0.60 to 0.36)	0.6322
parental involvement	-0.11 (-0.79 to 0.56)	0.7453	-0.14 (-0.92 to 0.64)	0.7194	-0.50 (-1.54 to 0.54)	0.3433	-0.07 (-0.82 to 0.68)	0.8594	0.02 (-0.46 to 0.50)	0.9428	-0.12 (-0.81 to 0.56)	0.7216
preplanning	**-1.06 (-1.98 to -0.14)**	0.0245	-0.14 (-1.22 to 0.95)	0.8065	-1.03 (-2.20 to 0.13)	0.0816	**-1.01 (-1.97 to -0.04)**	0.0407	-0.40 (-1.44 to 0.65)	0.4555	**-0.98 (-1.95 to -0.02)**	0.046
problem solving	-0.31 (-0.82 to 0.19)	0.2213	-0.35 (-0.95 to 0.24)	0.2456	-0.35 (-1.00 to 0.30)	0.287	-0.32 (-0.87 to 0.23)	0.2519	**-0.59 (-0.90 to -0.27)**	0.0002	-0.31 (-0.82 to 0.20)	0.2292
psychoeducation	-0.11 (-0.94 to 0.72)	0.7933	-0.10 (-1.02 to 0.81)	0.8251	0.18 (-1.16 to 1.51)	0.7935	-0.11 (-1.02 to 0.79)	0.8045	-0.14 (-0.88 to 0.60)	0.707	-0.14 (-0.99 to 0.70)	0.7401
remote	0.07 (-0.51 to 0.65)	0.8089	0.01 (-0.67 to 0.69)	0.9697	0.25 (-0.53 to 1.04)	0.5291	0.16 (-0.48 to 0.80)	0.628	0.08 (-0.31 to 0.46)	0.6997	0.03 (-0.58 to 0.63)	0.9284
reinforcement	**-0.53 (-1.03 to -0.03)**	0.0363	-0.48 (-1.08 to 0.13)	0.1222	-0.42 (-1.31 to 0.46)	0.3504	-0.49 (-1.05 to 0.07)	0.0843	**-0.53 (-1.04 to -0.03)**	0.0367	**-0.55 (-1.06 to -0.04)**	0.035
reminders	-0.01 (-0.62 to 0.59)	0.9619	0.01 (-0.72 to 0.74)	0.9831	0.08 (-0.65 to 0.82)	0.8246	0.07 (-0.62 to 0.77)	0.8375	-0.07 (-0.50 to 0.36)	0.7378	-0.01 (-0.62 to 0.61)	0.9842
role playing	0.51 (-1.06 to 2.08)	0.5235	0.59 (-1.46 to 2.64)	0.5733	1.20 (-1.57 to 3.96)	0.3954	0.49 (-1.18 to 2.15)	0.5653	0.88 (-0.17 to 1.93)	0.1017	0.58 (-1.03 to 2.19)	0.4812
relaxation training	0.09 (-1.78 to 1.96)	0.9260	0.35 (-1.93 to 2.63)	0.7635	—	—	-0.09 (-2.18 to 2.01)	0.9352	0.99 (-1.33 to 3.30)	0.4031	0.09 (-1.80 to 1.98)	0.9238
rule-setting	0.97 (-0.08 to 2.02)	0.0689	0.54 (-0.67 to 1.75)	0.378	0.70 (-0.87 to 2.27)	0.3809	0.77 (-0.48 to 2.02)	0.226	**1.05 (0.23 to 1.88)**	0.0124	0.97 (-0.09 to 2.03)	0.0717
stimulus control	-0.02 (-0.56 to 0.52)	0.9524	0.12 (-0.58 to 0.81)	0.7425	-0.28 (-1.23 to 0.68)	0.5712	-0.04 (-0.68 to 0.61)	0.9121	**-0.60 (-1.13 to -0.08)**	0.0249	-0.03 (-0.58 to 0.52)	0.9194
self-concept improvement	0.39 (-0.24 to 1.02)	0.2228	0.49 (-0.28 to 1.27)	0.2082	-0.05 (-1.19 to 1.08)	0.9266	0.35 (-0.33 to 1.04)	0.3129	0.15 (-0.33 to 0.63)	0.5372	0.41 (-0.23 to 1.06)	0.208
serious games	-0.30 (-0.81 to 0.20)	0.2345	-0.63 (-1.25 to -0.01)	0.0453	-0.47 (-1.36 to 0.41)	0.292	-0.31 (-0.87 to 0.25)	0.2837	0.02 (-0.96 to 1.00)	0.9721	-0.29 (-0.80 to 0.22)	0.2658
self-monitoring	**-0.59 (-1.15 to -0.03)**	0.0376	-0.61 (-1.31 to 0.09)	0.088	-0.68 (-1.44 to 0.08)	0.0801	**-0.65 (-1.28 to -0.03)**	0.0412	**-0.45 (-0.82 to -0.08)**	0.0174	**-0.63 (-1.21 to -0.05)**	0.034
social support	-0.12 (-0.73 to 0.48)	0.6862	0.01 (-0.73 to 0.75)	0.9821	-0.64 (-1.73 to 0.45)	0.2486	-0.12 (-0.79 to 0.56)	0.7372	-0.20 (-0.59 to 0.20)	0.3266	-0.17 (-0.80 to 0.47)	0.6019
stoplight approach	-0.18 (-0.83 to 0.46)	0.5730	-0.09 (-0.90 to 0.71)	0.8182	-0.11 (-0.91 to 0.70)	0.7972	-0.22 (-0.91 to 0.47)	0.529	0.34 (-0.61 to 1.29)	0.4841	-0.24 (-0.92 to 0.44)	0.4944
stress management	0.89 (-0.03 to 1.80)	0.0584	0.62 (-0.54 to 1.78)	0.2944	0.78 (-0.49 to 2.04)	0.2298	0.89 (-0.10 to 1.87)	0.077	0.64 (-0.04 to 1.32)	0.0652	0.90 (-0.03 to 1.83)	0.0574
task-setting	-0.32 (-0.86 to 0.22)	0.2465	-0.44 (-1.11 to 0.23)	0.2012	-0.20 (-1.04 to 0.65)	0.6463	-0.26 (-0.84 to 0.31)	0.3707	-0.47 (-0.98 to 0.03)	0.0681	-0.33 (-0.89 to 0.22)	0.2358
third-wave components	-0.45 (-2.06 to 1.16)	0.5873	-0.48 (-2.40 to 1.44)	0.6253	0.47 (-2.59 to 3.52)	0.7658	-0.32 (-2.07 to 1.43)	0.7188	-0.42 (-1.82 to 0.99)	0.5622	-0.41 (-2.04 to 1.22)	0.6212
wait-list	—	—	—	—	—	—	—	—	—	—	-0.22 (-1.07 to 0.63)	0.6056

Appendix 9 Reference list for included studies

1. Hofsteenge GH, Chinapaw MJM, Delemarre-van de Waal HA, et al. Long-term effect of the Go4it group treatment for obese adolescents: A randomised controlled trial. Clinical Nutrition 2014;33(3):385-91. doi: https://dx.doi.org/10.1016/j.clnu.2013.06.002

2. Abraham AA, Chow WC, So HK, et al. Lifestyle intervention using an internet-based curriculum with cell phone reminders for obese Chinese teens: a randomized controlled study. PLoS One 2015;10(5):e0125673. doi: 10.1371/journal.pone.0125673

3. Ahmad N, Shariff ZM, Mukhtar F, et al. Family-based intervention using face-to-face sessions and social media to improve Malay primary school children's adiposity: a randomized controlled field trial of the Malaysian REDUCE programme. Nutr J 2018;17(1):74. doi: 10.1186/s12937-018-0379-1

4. Alustiza E, Perales A, Mateo-Abad M, et al. Tackling risk factors for type 2 diabetes in adolescents: PRE-STARt study in Euskadi. An Pediatr (Engl Ed) 2021;95(3):186-96. doi: 10.1016/j.anpede.2020.11.005

5. Anderson YC, Leung W, Grant CC, et al. Economic evaluation of a multi-disciplinary community-based intervention programme for New Zealand children and adolescents with obesity. Obes Res Clin Pract 2018;12(3):293-98. doi: 10.1016/j.orcp.2018.04.001

6. Anderson YC, Wynter LE, Grant CC, et al. A Novel Home-Based Intervention for Child and Adolescent Obesity: The Results of the Whanau Pakari Randomized Controlled Trial. Obesity (Silver Spring) 2017;25(11):1965-73. doi: 10.1002/oby.21967

7. Arauz Boudreau AD, Kurowski DS, Gonzalez WI, et al. Latino families, primary care, and childhood obesity: a randomized controlled trial. Am J Prev Med 2013;44(3 Suppl 3):S247-57. doi: 10.1016/j.amepre.2012.11.026

8. Arlinghaus KR, O'Connor DP, Johnston CA. Frequency of school-based intervention needed to improve weight outcomes of Mexican-American adolescents with overweight or obesity: a randomized controlled trial. Pediatr Obes 2019;14(12):e12568. doi: 10.1111/ijpo.12568

9. Arlinghaus KR, O'Connor DP, Ledoux TA, et al. A staged approach to address youth unresponsive to initial obesity intervention: a randomized clinical trial. Int J Obes (Lond) 2021;45(12):2585-90. doi: 10.1038/s41366-021-00940-0

10. aSaelens BE, Sallis JF, Wilfley DE, et al. Behavioral weight control for overweight adolescents initiated in primary care. Obes Res 2002;10(1):22-32. doi: 10.1038/oby.2002.4

11. Bagherniya M, Mostafavi Darani F, Sharma M, et al. Assessment of the Efficacy of Physical Activity Level and Lifestyle Behavior Interventions Applying Social Cognitive Theory for Overweight and Obese Girl Adolescents. J Res Health Sci 2018;18(2):e00409.

12. Ball GD, Mackenzie-Rife KA, Newton MS, et al. One-on-one lifestyle coaching for managing adolescent obesity: Findings from a pilot, randomized controlled trial in a real-world, clinical setting. Paediatr Child Health 2011;16(6):345-50. doi: 10.1093/pch/16.6.345

13. Bean MK, Ingersoll KS, Powell P, et al. Impact of motivational interviewing on outcomes of an adolescent obesity treatment: results from the MI Values randomized controlled pilot trial. Clin Obes 2018;8(5):323-26. doi: 10.1111/cob.12257

14. Berkowitz RI, Rukstalis MR, Bishop-Gilyard CT, et al. Treatment of adolescent obesity comparing self-guided and group lifestyle modification programs: a potential model for primary care. J Pediatr Psychol 2013;38(9):978-86. doi: 10.1093/jpepsy/jst035

15. Berry DC, Schwartz TA, McMurray RG, et al. The family partners for health study: a cluster randomized controlled trial for child and parent weight management. Nutr Diabetes 2014;4(1):e101. doi: 10.1038/nutd.2013.42

16. Bocca G, Kuitert MW, Sauer PJ, et al. A multidisciplinary intervention programme has positive effects on quality of life in overweight and obese preschool children. Acta Paediatr 2014;103(9):962-7. doi: 10.1111/apa.12701

17. Bohlin A, Hagman E, Klaesson S, et al. Childhood obesity treatment: telephone coaching is as good as usual care in maintaining weight loss - a randomized controlled trial. Clin Obes 2017;7(4):199-205. doi: 10.1111/cob.12194

18. Boutelle KN, Rhee KE, Liang J, et al. Effect of Attendance of the Child on Body Weight, Energy Intake, and Physical Activity in Childhood Obesity Treatment: A Randomized Clinical Trial. JAMA Pediatr 2017;171(7):622-28. doi: 10.1001/jamapediatrics.2017.0651

19. Boutelle KN, Zucker N, Peterson CB, et al. An intervention based on Schachter's externality theory for overweight children: the regulation of cues pilot. J Pediatr Psychol 2014;39(4):405-17. doi: 10.1093/jpepsy/jst142

20. Broccoli S, Davoli AM, Bonvicini L, et al. Motivational Interviewing to Treat Overweight Children: 24-Month Follow-Up of a Randomized Controlled Trial. Pediatrics 2016;137(1) doi: 10.1542/peds.2015-1979

21. Chew CSE, Oh JY, Rajasegaran K, et al. Evaluation of a group family-based intervention programme for adolescent obesity: the LITE randomised controlled pilot trial. Singapore Med J 2021;62(1):39-47. doi: 10.11622/smedj.2019122

22. Christie D, Hudson LD, Kinra S, et al. A community-based motivational personalised lifestyle intervention to reduce BMI in obese adolescents: results from the Healthy Eating and Lifestyle Programme (HELP) randomised controlled trial. Arch Dis Child 2017;102(8):695-701. doi: 10.1136/archdischild-2016-311586

23. Cohen TR, Hazell TJ, Vanstone CA, et al. A family-centered lifestyle intervention for obese six- to eight-year-old children: Results from a one-year randomized controlled trial conducted in Montreal, Canada. Can J Public Health 2016;107(4-5):e453-e60. doi: 10.17269/cjph.107.5470

24. Cohen TR, Mak IL, Loiselle SE, et al. Changes in Adiposity without Impacting Bone Health in Nine- to Twelve-Year-Old Children with Overweight and Obesity after a One-Year Family-Centered Lifestyle Behavior Intervention. Child Obes 2023;19(1):46-56. doi: 10.1089/chi.2022.0008

25. Collins CE, Okely AD, Morgan PJ, et al. Parent diet modification, child activity, or both in obese children: An RCT. Pediatrics 2011;127(4):619-27. doi: https://dx.doi.org/10.1542/peds.2010-1518

26. Crespo NC, Talavera GA, Campbell NR, et al. A randomized controlled trial to prevent obesity among Latino paediatric patients. Pediatr Obes 2018;13(11):697-704. doi: 10.1111/ijpo.12466

27. Davis A, Lancaster B, Fleming K, et al. Effectiveness of a paediatric weight management intervention for rural youth (iAmHealthy): Primary outcomes of a cluster randomised control trial. Pediatr Obes 2024;19(3):e13094. doi: 10.1111/ijpo.13094

28. Davis AM, Sampilo M, Gallagher KS, et al. Treating rural pediatric obesity through telemedicine: outcomes from a small randomized controlled trial. J Pediatr Psychol 2013;38(9):932-43. doi: 10.1093/jpepsy/jst005

29. de Niet J, Timman R, Bauer S, et al. The effect of a short message service maintenance treatment on body mass index and psychological well-being in overweight and obese children: a randomized controlled trial. Pediatr Obes 2012;7(3):205-19. doi: 10.1111/j.2047-6310.2012.00048.x

30. Díaz RG, Esparza-Romero J, Moya-Camarena SY, et al. Lifestyle intervention in primary care settings improves obesity parameters among Mexican youth. J Am Diet Assoc 2010;110(2):285-90. doi: 10.1016/j.jada.2009.10.042

31. Doyle AC, Goldschmidt A, Huang C, et al. Reduction of Overweight and Eating Disorder Symptoms via the Internet in Adolescents: A Randomized Controlled Trial. Journal of Adolescent Health 2008;43(2):172-79. doi: https://dx.doi.org/10.1016/j.jadohealth.2008.01.011

32. Ek A, Lewis Chamberlain K, Sorjonen K, et al. A Parent Treatment Program for Preschoolers With Obesity: A Randomized Controlled Trial. Pediatrics 2019;144(2) doi: 10.1542/peds.2018-3457
33. Epstein LH, Paluch RA, Gordy CC, et al. Problem solving in the treatment of childhood obesity. J Consult Clin Psychol 2000;68(4):717-21
34. Epstein LH, Paluch RA, Kilanowski CK, et al. The effect of reinforcement or stimulus control to reduce sedentary behavior in the treatment of pediatric obesity. Health Psychol 2004;23(4):371-80. doi: 10.1037/0278-6133.23.4.371
35. Faith MS, Berman N, Heo M, et al. Effects of contingent television on physical activity and television viewing in obese children. Pediatrics 2001;107(5):1043-8. doi: 10.1542/peds.107.5.1043
36. Fedele DA, Janicke DM, McQuaid EL, et al. A Behavioral Family Intervention for Children with Overweight and Asthma. Clin Pract Pediatr Psychol 2018;6(3):259-69. doi: 10.1037/cpp0000237
37. Ford AL, Bergh C, Södersten P, et al. Treatment of childhood obesity by retraining eating behaviour: randomised controlled trial. BMJ 2009;340:b5388. doi: 10.1136/bmj.b5388
38. Freira S, Lemos MS, Fonseca H, et al. Anthropometric outcomes of a motivational interviewing school-based randomized trial involving adolescents with overweight. Eur J Pediatr 2018;177(7):1121-30. doi: 10.1007/s00431-018-3158-2
39. Fullerton G, Tyler C, Johnston CA, et al. Quality of life in Mexican-American children following a weight management program. Obesity (Silver Spring) 2007;15(11):2553-6. doi: 10.1038/oby.2007.306
40. Garipağaoğlu M, Sahip Y, Darendeliler F, et al. Family-based group treatment versus individual treatment in the management of childhood obesity: randomized, prospective clinical trial. Eur J Pediatr 2009;168(9):1091-9. doi: 10.1007/s00431-008-0894-8
41. Gerards SMPL, Dagnelie PC, Gubbels JS, et al. The effectiveness of lifestyle triple P in the Netherlands: A randomized controlled trial. PLoS ONE 2015;10(4):e0122240. doi: https://dx.doi.org/10.1371/journal.pone.0122240
42. Gillis D, Brauner M, Granot E. A community-based behavior modification intervention for childhood obesity. J Pediatr Endocrinol Metab 2007;20(2):197-203. doi: 10.1515/jpem.2007.20.2.197
43. Golan M, Kaufman V, Shahar DR. Childhood obesity treatment: targeting parents exclusively v. parents and children. Br J Nutr 2006;95(5):1008-15. doi: 10.1079/bjn20061757
44. Golley RK, Magarey AM, Baur LA, et al. Twelve-month effectiveness of a parent-led, family-focused weight-management program for prepubertal children: a randomized, controlled trial. Pediatrics 2007;119(3):517-25. doi: 10.1542/peds.2006-1746
45. Gourlan M, Sarrazin P, Trouilloud D. Motivational interviewing as a way to promote physical activity in obese adolescents: a randomised-controlled trial using self-determination theory as an explanatory framework. Psychol Health 2013;28(11):1265-86. doi: 10.1080/08870446.2013.800518
46. Grey M, Jaser SS, Holl MG, et al. A multifaceted school-based intervention to reduce risk for type 2 diabetes in at-risk youth. Preventive Medicine 2009;49(2-3):122-8. doi: https://dx.doi.org/10.1016/j.ypmed.2009.07.014
47. Hadley W, McCullough MB, Rancourt D, et al. Shaking up the system: the role of change in maternal-adolescent communication quality and adolescent weight loss. J Pediatr Psychol 2015;40(1):121-31. doi: 10.1093/jpepsy/jsu073
48. Ham OK, Sung KM, Lee BG, et al. Transtheoretical Model Based Exercise Counseling Combined with Music Skipping Rope Exercise on Childhood Obesity. Asian Nurs Res (Korean Soc Nurs Sci) 2016;10(2):116-22. doi: 10.1016/j.anr.2016.03.003
49. Hidayanty H, Bardosono S, Khusun H, et al. A social cognitive theory-based programme for eating patterns and sedentary activity among overweight adolescents in Makassar, South Sulawesi : a cluster randomised

controlled trial. Asia Pacific journal of clinical nutrition 2016;25(Suppl 1):S83-S92. doi: https://doi.org/10.6133/apjcn.122016.s7
50. Hinton EC, Birch LA, Barton J, et al. Using neuroimaging to investigate the impact of Mandolean® training in young people with obesity: a pilot randomised controlled trial. BMC Pediatr 2018;18(1):366. doi: 10.1186/s12887-018-1342-1
51. Hughes AR, Stewart L, Chapple J, et al. Randomized, controlled trial of a best-practice individualized behavioral program for treatment of childhood overweight: Scottish Childhood Overweight Treatment Trial (SCOTT). Pediatrics 2008;121(3):e539-46. doi: 10.1542/peds.2007-1786
52. Hystad HT, Steinsbekk S, Ødegård R, et al. A randomised study on the effectiveness of therapist-led v. self-help parental intervention for treating childhood obesity. Br J Nutr 2013;110(6):1143-50. doi: 10.1017/s0007114513000056
53. Janicke DM, Lim CS, Perri MG, et al. Featured Article: Behavior Interventions Addressing Obesity in Rural Settings: The E-FLIP for Kids Trial. J Pediatr Psychol 2019;44(8):889-901. doi: 10.1093/jpepsy/jsz029
54. Janicke DM, Sallinen BJ, Perri MG, et al. Comparison of parent-only vs family-based interventions for overweight children in underserved rural settings: outcomes from project STORY. Arch Pediatr Adolesc Med 2008;162(12):1119-25. doi: 10.1001/archpedi.162.12.1119
55. Jelalian E, Mehlenbeck R, Lloyd-Richardson EE, et al. 'Adventure therapy' combined with cognitive-behavioral treatment for overweight adolescents. Int J Obes (Lond) 2006;30(1):31-9. doi: 10.1038/sj.ijo.0803069
56. Jelalian E, Sato A, Hart CN. The effect of group-based weight-control intervention on adolescent psychosocial outcomes: perceived peer rejection, social anxiety, and self-concept. Children's health care 2011;40(3):197-211. doi: https://doi.org/10.1080/02739615.2011.590391
57. Jiang JX, Xia XL, Greiner T, et al. A two year family based behaviour treatment for obese children. Arch Dis Child 2005;90(12):1235-8. doi: 10.1136/adc.2005.071753
58. Johnston CA, Moreno JP, Gallagher MR, et al. Achieving long-term weight maintenance in Mexican-American adolescents with a school-based intervention. J Adolesc Health 2013;53(3):335-41. doi: 10.1016/j.jadohealth.2013.04.001
59. Johnston CA, Tyler C, Fullerton G, et al. Effects of a school-based weight maintenance program for Mexican-American children: results at 2 years. Obesity (Silver Spring) 2010;18(3):542-7. doi: 10.1038/oby.2009.241
60. Kalarchian MA, Levine MD, Arslanian SA, et al. Family-based treatment of severe pediatric obesity: randomized, controlled trial. Pediatrics 2009;124(4):1060-8. doi: 10.1542/peds.2008-3727
61. Kalavainen MP, Korppi MO, Nuutinen OM. Clinical efficacy of group-based treatment for childhood obesity compared with routinely given individual counseling. Int J Obes (Lond) 2007;31(10):1500-8. doi: 10.1038/sj.ijo.0803628
62. Kitzman-Ulrich H, Hampson R, Wilson DK, et al. An adolescent weight-loss program integrating family variables reduces energy intake. J Am Diet Assoc 2009;109(3):491-6. doi: 10.1016/j.jada.2008.11.029
63. Kulendran M, King D, Schmidtke KA, et al. The use of commitment techniques to support weight loss maintenance in obese adolescents. Psychol Health 2016;31(11):1332-41. doi: 10.1080/08870446.2016.1204452
64. Looney SM, Raynor HA. Examining the effect of three low-intensity pediatric obesity interventions: a pilot randomized controlled trial. Clin Pediatr (Phila) 2014;53(14):1367-74. doi: 10.1177/0009922814541803
65. Love-Osborne K, Fortune R, Sheeder J, et al. School-based health center-based treatment for obese adolescents: feasibility and body mass index effects. Child Obes 2014;10(5):424-31. doi: 10.1089/chi.2013.0165

66. Luque V, Feliu A, Closa-Monasterolo R, et al. Impact of the motivational interviewing for childhood obesity treatment: The Obemat2.0 randomized clinical trial. Pediatr Obes 2024;19(7):e13125. doi: 10.1111/ijpo.13125
67. Macdonell K, Brogan K, Naar-King S, et al. A pilot study of motivational interviewing targeting weight-related behaviors in overweight or obese African American adolescents. J Adolesc Health 2012;50(2):201-3. doi: 10.1016/j.jadohealth.2011.04.018
68. Maddison R, Marsh S, Foley L, et al. Screen-Time Weight-loss Intervention Targeting Children at Home (SWITCH): a randomized controlled trial. Int J Behav Nutr Phys Act 2014;11:111. doi: 10.1186/s12966-014-0111-2
69. Magarey AM, Perry RA, Baur LA, et al. A parent-led family-focused treatment program for overweight children aged 5 to 9 years: the PEACH RCT. Pediatrics 2011;127(2):214-22. doi: 10.1542/peds.2009-1432
70. Markert J, Herget S, Petroff D, et al. Telephone-based adiposity prevention for families with overweight children (T.A.F.F.-Study): one year outcome of a randomized, controlled trial. Int J Environ Res Public Health 2014;11(10):10327-44. doi: 10.3390/ijerph111010327
71. Marques DCS, Ferreira WC, Santos IC, et al. Impacts of a Multi-Professional Family versus Isolated Intervention on Food Level Processing in Overweight Adolescents: A Randomized Trial. Nutrients 2023;15(4) doi: 10.3390/nu15040935
72. McCallum Z, Wake M, Gerner B, et al. Outcome data from the LEAP (Live, Eat and Play) trial: a randomized controlled trial of a primary care intervention for childhood overweight/mild obesity. Int J Obes (Lond) 2007;31(4):630-6. doi: 10.1038/sj.ijo.0803509
73. Miri SF, Javadi M, Lin CY, et al. Effectiveness of cognitive-behavioral therapy on nutrition improvement and weight of overweight and obese adolescents: A randomized controlled trial. Diabetes Metab Syndr 2019;13(3):2190-97. doi: 10.1016/j.dsx.2019.05.010
74. Moore SM, Borawski EA, Love TE, et al. Two Family Interventions to Reduce BMI in Low-Income Urban Youth: A Randomized Trial. Pediatrics 2019;143(6) doi: 10.1542/peds.2018-2185
75. Nemet D, Barkan S, Epstein Y, et al. Short- and long-term beneficial effects of a combined dietary-behavioral-physical activity intervention for the treatment of childhood obesity. Pediatrics 2005;115(4):e443-e49. doi: https://dx.doi.org/10.1542/peds.2004-2172
76. Nguyen B, Shrewsbury VA, O'Connor J, et al. Twelve-month outcomes of the loozit randomized controlled trial: a community-based healthy lifestyle program for overweight and obese adolescents. Arch Pediatr Adolesc Med 2012;166(2):170-7. doi: 10.1001/archpediatrics.2011.841
77. Njardvik U, Gunnarsdottir T, Olafsdottir AS, et al. Incorporating Appetite Awareness Training Within Family-Based Behavioral Treatment of Pediatric Obesity: A Randomized Controlled Pilot Study. J Pediatr Psychol 2018;43(9):1017-27. doi: 10.1093/jpepsy/jsy055
78. Norman G, Huang J, Davila EP, et al. Outcomes of a 1-year randomized controlled trial to evaluate a behavioral 'stepped-down' weight loss intervention for adolescent patients with obesity. Pediatr Obes 2016;11(1):18-25. doi: 10.1111/ijpo.12013
79. O'Connor TM, Hilmers A, Watson K, et al. Feasibility of an obesity intervention for paediatric primary care targeting parenting and children: Helping HAND. Child Care Health Dev 2013;39(1):141-9. doi: 10.1111/j.1365-2214.2011.01344.x
80. Pakpour AH, Gellert P, Dombrowski SU, et al. Motivational interviewing with parents for obesity: an RCT. Pediatrics 2015;135(3):e644-52. doi: 10.1542/peds.2014-1987
81. Patrick K, Norman GJ, Davila EP, et al. Outcomes of a 12-month technology-based intervention to promote weight loss in adolescents at risk for type 2 diabetes. J Diabetes Sci Technol 2013;7(3):759-70. doi: 10.1177/193229681300700322

82. Patsopoulou A, Tsimtsiou Z, Katsioulis A, et al. Evaluating the Efficacy of the Feeding Exercise Randomized Trial in Overweight and Obese Adolescents. Child Obes 2017;13(2):128-37. doi: 10.1089/chi.2016.0192
83. Pbert L, Druker S, Barton B, et al. A School-Based Program for Overweight and Obese Adolescents: A Randomized Controlled Trial. J Sch Health 2016;86(10):699-708. doi: 10.1111/josh.12428
84. Quattrin T, Roemmich JN, Paluch R, et al. Treatment outcomes of overweight children and parents in the medical home. Pediatrics 2014;134(2):290-7. doi: 10.1542/peds.2013-4084
85. Resnicow K, Taylor R, Baskin M, et al. Results of go girls: a weight control program for overweight African-American adolescent females. Obes Res 2005;13(10):1739-48. doi: 10.1038/oby.2005.212
86. Rhee KE, Herrera L, Strong D, et al. Guided Self-Help for Pediatric Obesity in Primary Care: A Randomized Clinical Trial. Pediatrics 2022;150(1) doi: 10.1542/peds.2021-055366
87. Robinson TN, Matheson D, Wilson DM, et al. A community-based, multi-level, multi-setting, multi-component intervention to reduce weight gain among low socioeconomic status Latinx children with overweight or obesity: The Stanford GOALS randomised controlled trial. Lancet Diabetes Endocrinol 2021;9(6):336-49. doi: 10.1016/s2213-8587(21)00084-x
88. Ruotsalainen H, Kyngäs H, Tammelin T, et al. Effectiveness of Facebook-Delivered Lifestyle Counselling and Physical Activity Self-Monitoring on Physical Activity and Body Mass Index in Overweight and Obese Adolescents: A Randomized Controlled Trial. Nurs Res Pract 2015;2015:159205. doi: 10.1155/2015/159205
89. Saelens BE, Lozano P, Scholz K. A randomized clinical trial comparing delivery of behavioral pediatric obesity treatment using standard and enhanced motivational approaches. J Pediatr Psychol 2013;38(9):954-64. doi: 10.1093/jpepsy/jst054
90. Sánchez-López AM, Menor-Rodríguez MJ, Sánchez-García JC, et al. Play as a Method to Reduce Overweight and Obesity in Children: An RCT. Int J Environ Res Public Health 2020;17(1) doi: 10.3390/ijerph17010346
91. Sauder KA, Dabelea D, Bailey-Callahan R, et al. Targeting risk factors for type 2 diabetes in American Indian youth: the Tribal Turning Point pilot study. Pediatr Obes 2018;13(5):321-29. doi: 10.1111/ijpo.12223
92. Savoye M, Nowicka P, Shaw M, et al. Long-term results of an obesity program in an ethnically diverse pediatric population. Pediatrics 2011;127(3):402-10. doi: 10.1542/peds.2010-0697
93. Sen M, Uzuner A, Akman M, et al. Examination of a board game approach to children's involvement in family-based weight management vs. traditional family-based behavioral counseling in primary care. Eur J Pediatr 2018;177(8):1231-38. doi: 10.1007/s00431-018-3177-z
94. Serra-Paya N, Ensenyat A, Castro-Viñuales I, et al. Effectiveness of a Multi-Component Intervention for Overweight and Obese Children (Nereu Program): A Randomized Controlled Trial. PLoS One 2015;10(12):e0144502. doi: 10.1371/journal.pone.0144502
95. Small L, Bonds-McClain D, Melnyk B, et al. The preliminary effects of a primary care-based randomized treatment trial with overweight and obese young children and their parents. J Pediatr Health Care 2014;28(3):198-207. doi: 10.1016/j.pedhc.2013.01.003
96. Smith JD, Berkel C, Carroll AJ, et al. Health behaviour outcomes of a family based intervention for paediatric obesity in primary care: A randomized type II hybrid effectiveness-implementation trial. Pediatr Obes 2021;16(9):e12780. doi: 10.1111/ijpo.12780
97. Spence ND, Newton AS, Keaschuk RA, et al. Parents as Agents of Change in Managing Pediatric Obesity: A Randomized Controlled Trial Comparing Cognitive Behavioral Therapy versus Psychoeducation Interventions. Child Obes 2023;19(2):71-87. doi: 10.1089/chi.2021.0194
98. Stark LJ, Filigno SS, Kichler JC, et al. Maintenance Following a Randomized Trial of a Clinic and Home-based Behavioral Intervention of Obesity in Preschoolers. J Pediatr 2019;213:128-36.e3. doi: 10.1016/j.jpeds.2019.05.004

99. Stasinaki A, Büchter D, Shih CI, et al. Effects of a novel mobile health intervention compared to a multi-component behaviour changing program on body mass index, physical capacities and stress parameters in adolescents with obesity: a randomized controlled trial. BMC Pediatr 2021;21(1):308. doi: 10.1186/s12887-021-02781-2
100. Taveras EM, Gortmaker SL, Hohman KH, et al. Randomized controlled trial to improve primary care to prevent and manage childhood obesity: the High Five for Kids study. Arch Pediatr Adolesc Med 2011;165(8):714-22. doi: 10.1001/archpediatrics.2011.44
101. Taveras EM, Marshall R, Kleinman KP, et al. Comparative effectiveness of childhood obesity interventions in pediatric primary care: a cluster-randomized clinical trial. JAMA Pediatr 2015;169(6):535-42. doi: 10.1001/jamapediatrics.2015.0182
102. Taveras EM, Marshall R, Sharifi M, et al. Comparative Effectiveness of Clinical-Community Childhood Obesity Interventions: A Randomized Clinical Trial. JAMA Pediatr 2017;171(8):e171325. doi: 10.1001/jamapediatrics.2017.1325
103. Taylor RW, Cox A, Knight L, et al. A Tailored Family-Based Obesity Intervention: A Randomized Trial. Pediatrics 2015;136(2):281-9. doi: 10.1542/peds.2015-0595
104. Trost SG, Sundal D, Foster GD, et al. Effects of a pediatric weight management program with and without active video games a randomized trial. JAMA Pediatr 2014;168(5):407-13. doi: 10.1001/jamapediatrics.2013.3436
105. Tsai SY, Tung YC, Huang CM, et al. A family-based and mobile-assisted intervention for lifestyle behaviors in youths: A randomized controlled trial. Res Nurs Health 2024;47(4):384-96. doi: 10.1002/nur.22374
106. Tsiros MD, Sinn N, Brennan L, et al. Cognitive behavioral therapy improves diet and body composition in overweight and obese adolescents. Am J Clin Nutr 2008;87(5):1134-40. doi: 10.1093/ajcn/87.5.1134
107. Tucker SJ, Ytterberg KL, Lenoch LM, et al. Reducing pediatric overweight: nurse-delivered motivational interviewing in primary care. J Pediatr Nurs 2013;28(6):536-47. doi: 10.1016/j.pedn.2013.02.031
108. Varagiannis P, Magriplis E, Risvas G, et al. Effects of Three Different Family-Based Interventions in Overweight and Obese Children: The "4 Your Family" Randomized Controlled Trial. Nutrients 2021;13(2) doi: 10.3390/nu13020341
109. Vermeiren E, Naets T, Van Eyck A, et al. Improving Treatment Outcome in Children With Obesity by an Online Self-Control Training: A Randomized Controlled Trial. Front Pediatr 2021;9:794256. doi: 10.3389/fped.2021.794256
110. Vidmar AP, Naguib M, Raymond JK, et al. Time-Limited Eating and Continuous Glucose Monitoring in Adolescents with Obesity: A Pilot Study. Nutrients 2021;13(11) doi: 10.3390/nu13113697
111. Vidmar AP, Salvy SJ, Wee CP, et al. An addiction-based digital weight loss intervention: A multi-centre randomized controlled trial. Pediatr Obes 2023;18(3):e12990. doi: 10.1111/ijpo.12990
112. Vos RC, Huisman SD, Houdijk EC, et al. The effect of family-based multidisciplinary cognitive behavioral treatment on health-related quality of life in childhood obesity. Qual Life Res 2012;21(9):1587-94. doi: 10.1007/s11136-011-0079-1
113. Wafa SW, Talib RA, Hamzaid NH, et al. Randomized controlled trial of a good practice approach to treatment of childhood obesity in Malaysia: Malaysian Childhood Obesity Treatment Trial (MASCOT). Int J Pediatr Obes 2011;6(2-2):e62-9. doi: 10.3109/17477166.2011.566340
114. Wake M, Baur LA, Gerner B, et al. Outcomes and costs of primary care surveillance and intervention for overweight or obese children: the LEAP 2 randomised controlled trial. BMJ 2009;339:b3308. doi: 10.1136/bmj.b3308
115. Wake M, Lycett K, Clifford SA, et al. Shared care obesity management in 3-10 year old children: 12 month outcomes of HopSCOTCH randomised trial. BMJ 2013;346:f3092. doi: 10.1136/bmj.f3092

116. Waling M, Bäcklund C, Lind T, et al. Effects on metabolic health after a 1-year-lifestyle intervention in overweight and obese children: a randomized controlled trial. J Nutr Metab 2012;2012:913965. doi: 10.1155/2012/913965
117. Walpole B, Dettmer E, Morrongiello BA, et al. Motivational interviewing to enhance self-efficacy and promote weight loss in overweight and obese adolescents: a randomized controlled trial. J Pediatr Psychol 2013;38(9):944-53. doi: 10.1093/jpepsy/jst023
118. Warschburger P, Kroeller K, Haerting J, et al. Empowering Parents of Obese Children (EPOC): A randomized controlled trial on additional long-term weight effects of parent training. Appetite 2016;103:148-56. doi: 10.1016/j.appet.2016.04.007
119. Weigensberg MJ, Lane CJ, Avila Q, et al. Imagine HEALTH: Results from a randomized pilot lifestyle intervention for obese Latino adolescents using Interactive Guided ImagerySM. BMC Complementary and Alternative Medicine 2014;17:14:28. doi: https://dx.doi.org/10.1186/1472-6882-14-28
120. Willeboordse M, Van De Kant KDG, Tan FES, et al. A Multifactorial Weight Reduction Programme for Children with Overweight and Asthma: A Randomized Controlled Trial. PLoS ONE 2016;11(6):e0157158. doi: https://dx.doi.org/10.1371/journal.pone.0157158
121. Williamson DA, Martin PD, White MA, et al. Efficacy of an internet-based behavioral weight loss program for overweight adolescent African-American girls. Eat Weight Disord 2005;10(3):193-203. doi: 10.1007/bf03327547
122. Wright JA, Phillips BD, Watson BL, et al. Randomized trial of a family-based, automated, conversational obesity treatment program for underserved populations. Obesity (Silver Spring) 2013;21(9):E369-78. doi: 10.1002/oby.20388
123. Wylie-Rosett J, Groisman-Perelstein AE, Diamantis PM, et al. Embedding weight management into safety-net pediatric primary care: randomized controlled trial. Int J Behav Nutr Phys Act 2018;15(1):12. doi: 10.1186/s12966-017-0639-z
124. Yackobovitch-Gavan M, Wolf Linhard D, Nagelberg N, et al. Intervention for childhood obesity based on parents only or parents and child compared with follow-up alone. Pediatr Obes 2018;13(11):647-55. doi: 10.1111/ijpo.12263
125. Zoellner JM, You W, Hill JL, et al. Comparing two different family-based childhood obesity treatment programmes in a medically underserved region: Effectiveness, engagement and implementation outcomes from a randomized controlled trial. Pediatr Obes 2022;17(1):e12840. doi: 10.1111/ijpo.12840
